# Supplementary material for: PlexinD1 is a driver and a therapeutic target in advanced prostate cancer
Source: EMBO Mol Med. 2025 Jan 2;17(2):336–64. doi: 10.1038/s44321-024-00186-z (PMC11822115; doi:10.1038/s44321-024-00186-z)
Supplement: Supplementary file 1 — Appendix [file 44321_2024_186_MOESM1_ESM.pdf]

Appendix

PlexinD1 Is a Driver and a Therapeutic Target in Advanced Prostate Cancer

Jing Wei, Jing Wang, Wen Guan, Jingjing Li, Tianjie Pu, Eva Corey, Tzu-Ping Lin, Allen C. Gao, and Boyang Jason Wu

Table of Contents

Appendix Figure S1……………………………………………………………………………………………...2
Appendix Figure S2……………………………………………………………………………………………...3
Appendix Figure S3……………………………………………………………………………………………...4
Appendix Figure S4……………………………………………………………………………………………...5
Appendix Figure S5……………………………………………………………………………………………...6
Appendix Figure S6……………………………………………………………………………………………...7
Appendix Figure S7……………………………………………………………………………………………...8
Appendix Figure S8……………………………………………………………………………………………...9
Appendix Figure S9……………………………………………………………………………………………10
Appendix Figure S10…………………………………………………………………………………………...11
Appendix Figure S11…………………………………………………………………………………………...12
Appendix Figure S12…………………………………………………………………………………………..13
Appendix Figure S13…………………………………………………………………………………………..14
Appendix Table S1……………………………………………………………………………………………..16
Appendix Table S2……………………………………………………………………………………………..17
Appendix Table S3……………………………………………………………………………………………..18
Appendix Table S4……………………………………………………………………………………………..19

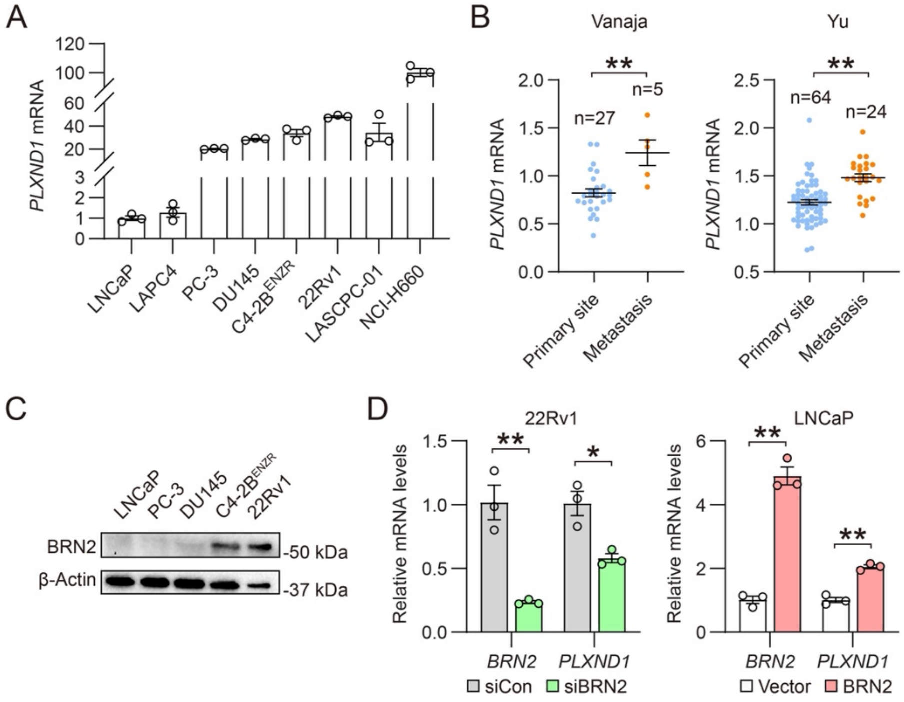

Appendix Figure S1. BRN2 is a potential transcriptional regulator of PlexinD1 in 22Rv1 cells.
(**A**) qPCR of *PLXND1* mRNA levels in a panel of human PCa cell lines (n=3 biological replicates). (**B**) Comparisons of *PLXND1* mRNA levels in metastatic vs. primary PCa from Vanaja and Yu datasets. (**C**) Western blot of BRN2 in multiple human PCa cell lines. (**D**) qPCR of *BRN2* and *PLXND1* in siRNA-mediated BRN2-knockdown 22Rv1, BRN2-overexpressing LNCaP, and their respective control cells (n=3 biological replicates). Data information: In (A, B, D), data are presented as mean ± SEM. In (B, D), P values were determined by unpaired two-tailed Student's t-test. *P<0.05, **P<0.01. Exact P values are listed in **Appendix Table S4**.

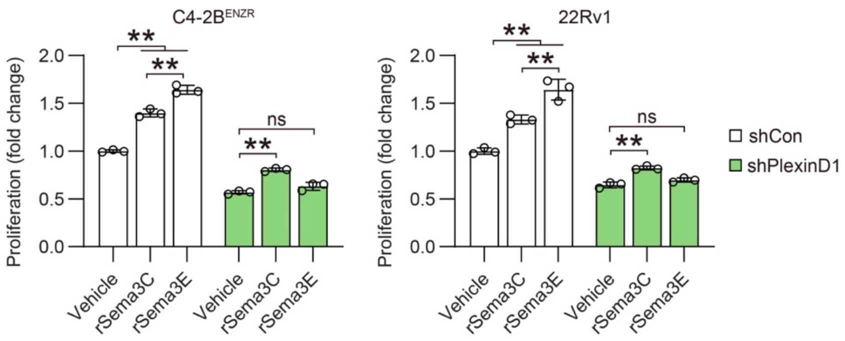

Appendix Figure S2. PlexinD1 promotes CRPC cell proliferation in a Sema3 ligand-dependent manner. Cell proliferation assays of control and PlexinD1-knockdown C4-2B^{ENZR} and 22Rv1 cells treated with recombinant Sema3C or Sema3E (200-500 ng/µl) proteins in serum-free medium for a 3-day observation period. Data represent the fold changes of cell proliferation on day 3 relative to treatment day (day 1) (*n*=3 biological replicates), with fold changes in the non-treated control cells set as 1 for normalization of other groups. Data information: Data are presented as mean ± SEM. *P* values were determined by one-way ANOVA with Tukey's multiple comparisons test. ***P*<0.01; ns, not significant. Exact *P* values are listed in **Appendix Table S4**.

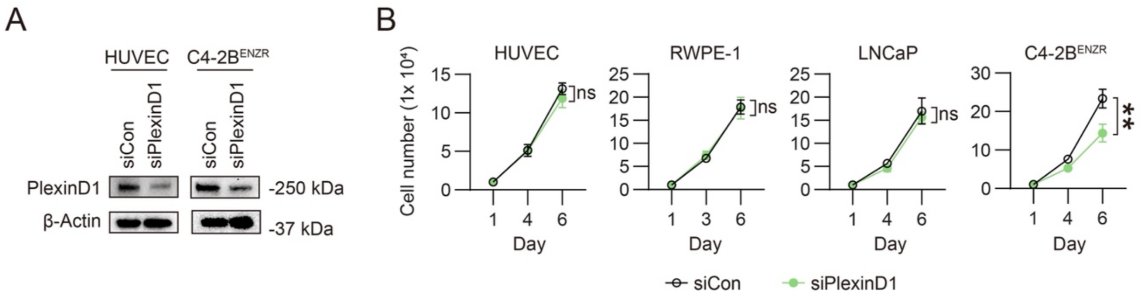

Appendix Figure S3. PlexinD1 knockdown does not affect the proliferation of normal and non-CRPC cells. (**A**) Western blot of PlexinD1 in control and siRNA-mediated PlexinD1-knockdown HUVEC and C4-2B^{ENZR} cells. (**B**) Cell proliferation assays of control and siRNA-mediated PlexinD1-knockdown HUVEC, RWPE-1, LNCaP and C4-2B^{ENZR} cells during a 6-day observation period (*n*=3 biological replicates). Upon siRNA transfection, cells were replated towards counting with the cell seeding day set as day 0. Data information: In (B), data are presented as mean ± SEM. *P* values were determined by unpaired two-tailed Student's *t*-test. ***P*<0.01; ns, not significant. Exact *P* values are listed in **Appendix Table S4**.

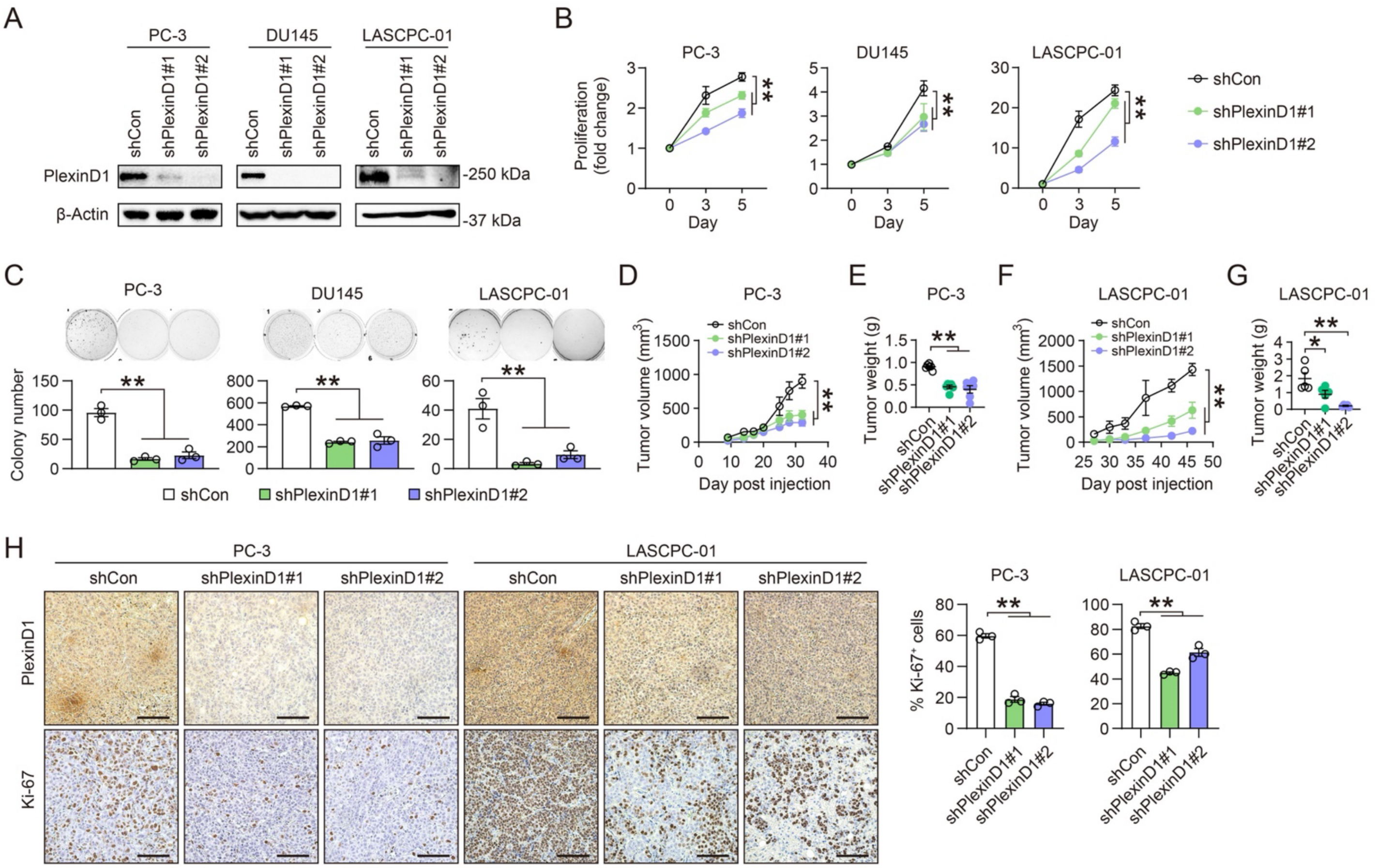

Appendix Figure S4. PlexinD1 silencing restricts CRPC growth *in vitro* and *in vivo*.
(**A**) Western blot of PlexinD1 in control and PlexinD1-knockdown CRPC cells as indicated. (**B**) Cell proliferation assays of control and PlexinD1-knockdown CRPC cells (*n*=4 biological replicates). Data represent the fold changes of cell proliferation during a 5-day observation period, with fold change on the day of cell seeding (day 0) in each group set as 1. (**C**) Representative images and quantification of colonies formed by control and PlexinD1-knockdown CRPC cells (*n*=3 biological replicates). (**D**-**G**) Tumor growth curves (D, F) and endpoint tumor weights (E, G) of control and PlexinD1-knockdown PC-3 and LASCPC-01 s.c. tumors grown in male NSG mice (*n*=5 tumors). (**H**) Representative IHC staining of PlexinD1 and Ki-67 and quantification of % of Ki-67+ cells in control and PlexinD1-knockdown PC-3 and LASCPC-01 tumors (*n*=3 tumors). Scale bars: 100 µm. Data information: In (B-H), data are presented as mean ± SEM (B-H). *P* values were determined by one-way ANOVA with Dunnett's multiple comparisons test. **P*<0.05, ***P*<0.01. Exact *P* values are listed in **Appendix Table S4**.

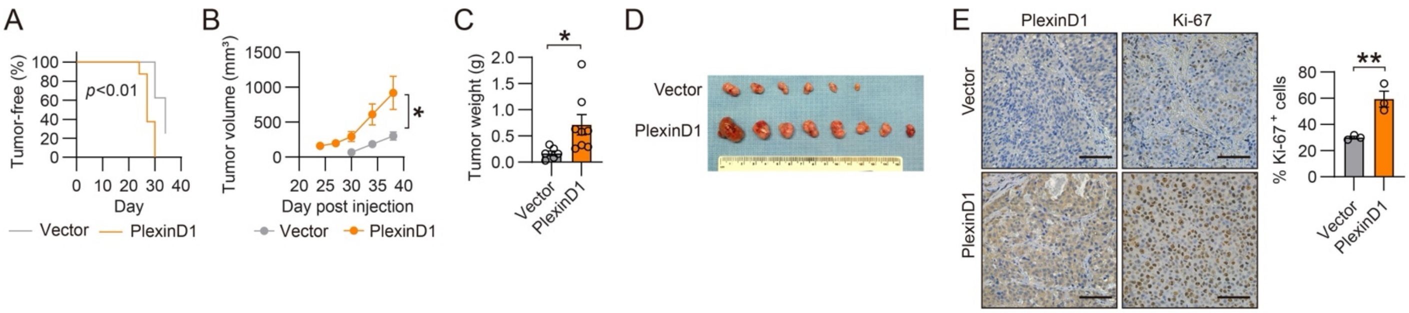

Appendix Figure S5. Overexpression of PlexinD1 promotes LNCaP prostate tumor growth.
(**A**) Kaplan-Meier tumor-free curves of mice s.c. inoculated with control and PlexinD1-overexpressing LNCaP cells (*n*=8 tumor inoculations). (**B**-**D**) Tumor growth curves (B), endpoint tumor weights (C), and anatomic tumor images (D) of mice bearing LNCaP tumors (*n*=6 and 8 tumors for Vector and PlexinD1 groups respectively) as described in (A). (**E**) Representative IHC staining of PlexinD1 and Ki-67 and quantification of % of Ki-67+ cells in LNCaP tumor samples (*n*=3 tumors). Scale bars: 100 μm. Data information: In (B, C, E), data are presented as mean ± SEM. In (A), the *P* value was determined by log-rank test. In (B, C, E), *P* values were determined by unpaired two-tailed Student's *t*-test. *P<0.05; **P<0.01. Exact *P* values are listed in **Appendix Table S4**.

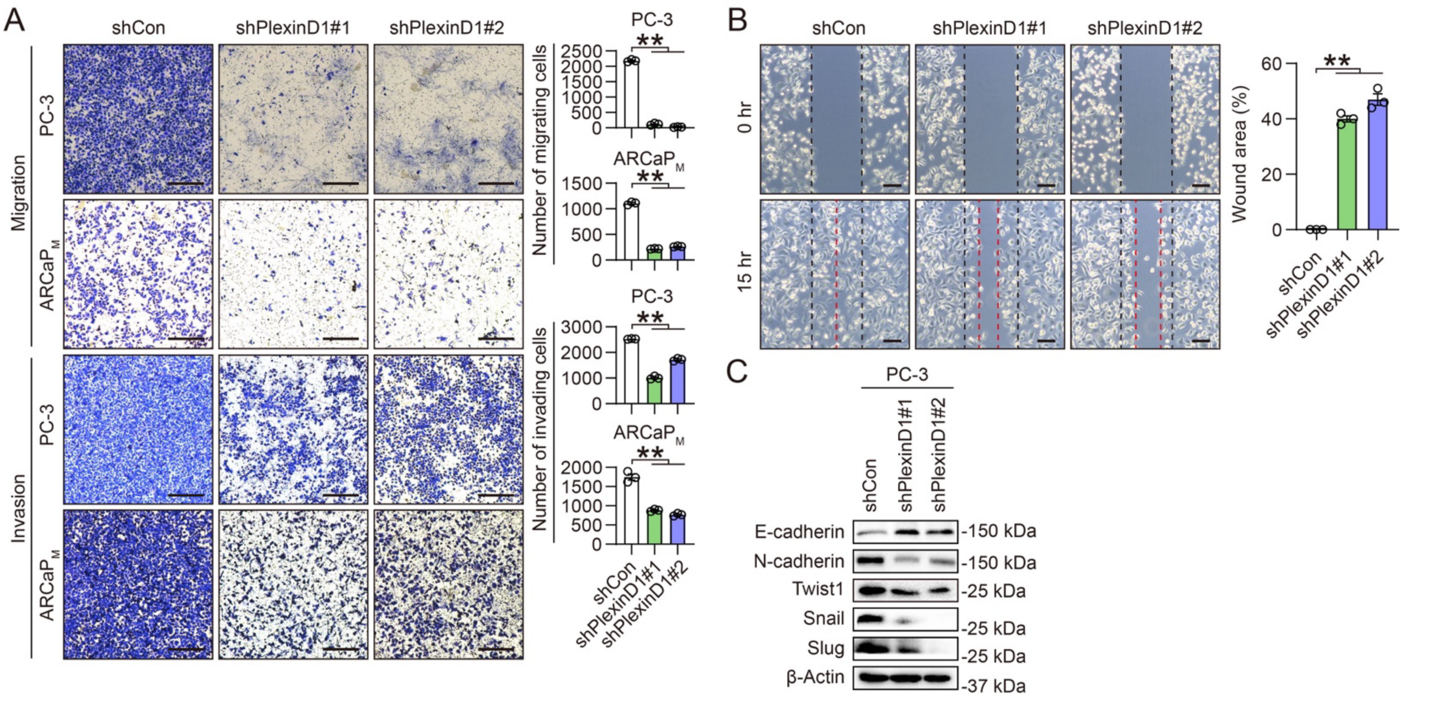

Appendix Figure S6. PlexinD1 knockdown impairs CRPC cell migration, invasion and EMT.
(**A**) Representative images and quantification of transwell-based cell migration and invasion by control and PlexinD1-knockdown PC-3 and ARCaP$_M$ cells (*n*=3 biological replicates). Scale bars: 400 μm. (**B**) Wound healing assays of control and PlexinD1-knockdown PC-3 cells (*n*=3 biological replicates). Scale bars: 100 μm. (**C**) Western blot of EMT markers as indicated in control and PlexinD1-knockdown PC-3 cells. Data information: In (A, B), data are presented as mean ± SEM. *P* values were determined by one-way ANOVA with Dunnett's multiple comparisons test. ***P*<0.01. Exact *P* values are listed in **Appendix Table S4**.

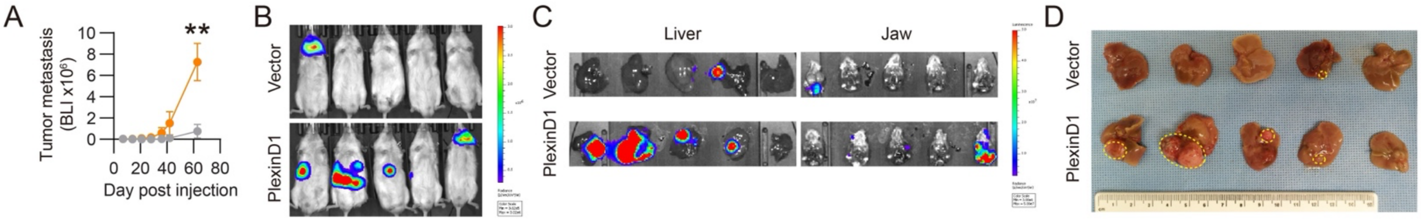

Appendix Figure S7. Overexpression of PlexinD1 promotes LNCaP prostate tumor metastasis.
(**A**, **B**) Bioluminescence (BLI)-based growth curves (A) and endpoint whole-body BLI images (B) of Luc-tagged control and PlexinD1-overexpressing LNCaP tumor metastasis developed in an intracardiac xenograft model (*n*=5 mice). (**C**) *Ex vivo* BLI images of tumor metastasis developed in the liver and jawbone of mice by control and PlexinD1-overexpressing LNCaP cells (*n*=5 mice). (**D**) Anatomic images of liver metastasis developed in mice inoculated with control and PlexinD1-overexpressing LNCaP cells, with yellow dashed lines circling liver metastatic tumors (*n*=5 mice). Data information: In (A), data are presented as mean ± SEM. *P* values were determined by unpaired two-tailed Student's *t*-test. ***P*<0.01. Exact *P* values are listed in **Appendix Table S4**.

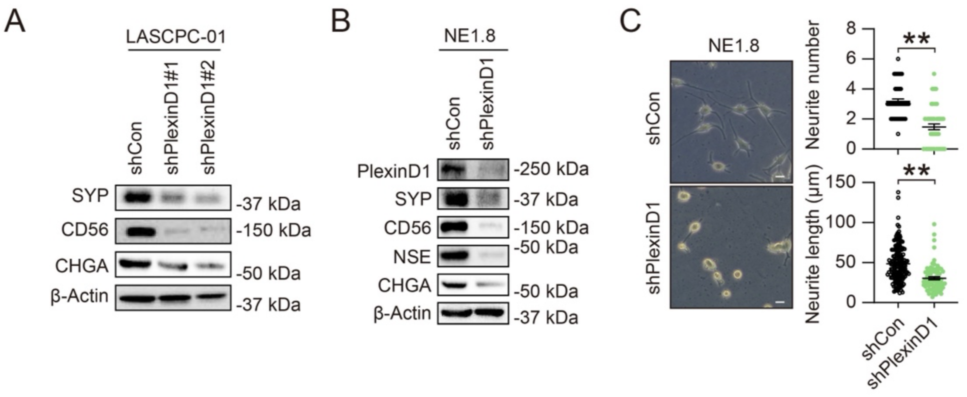

Appendix Figure S8. PlexinD1 silencing represses NE traits in CRPC cells.
(**A**) Western blot of NE markers as indicated in control and PlexinD1-knockdown LASCPC-01 cells. (**B**) Western blot of PlexinD1 and NE markers as indicated in control and PlexinD1-knockdown NE1.8 cells. (**C**) Representative images of control and PlexinD1-knockdown NE1.8 cell morphology and corresponding quantification of per-cell number of neurites and neurite length in each group (*n*=50 cells per group). A representative of three independent experiments was shown. Scale bars: 20 μm. Data information: In (C), data are presented as mean ± SEM (C). The *P* value was determined by unpaired two-tailed Student's *t*-test. ***P*<0.01. Exact *P* values are listed in **Appendix Table S4**.

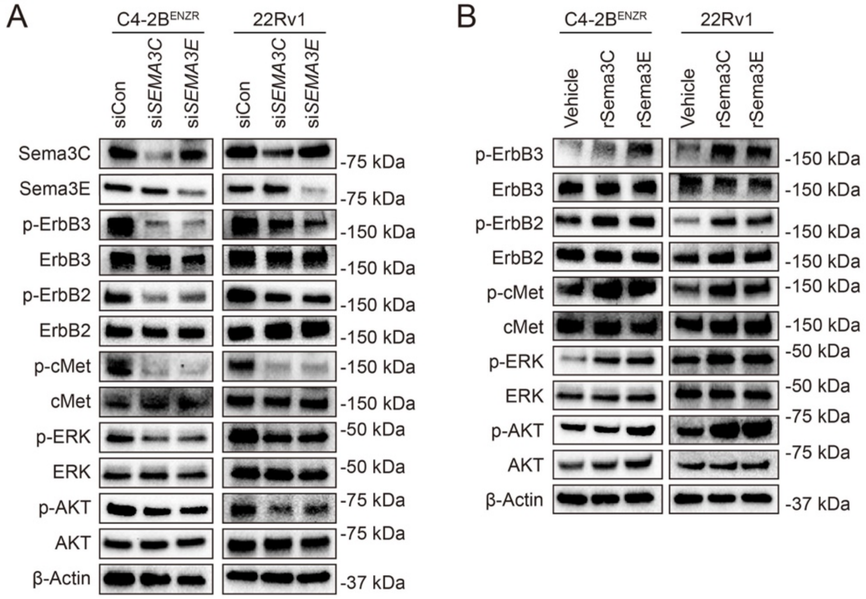

Appendix Figure S9. Sema3C and Sema3E activate PlexinD1-elicited ErbB3/cMet-ERK/AKT signaling.
(**A**) Western blot of Sema3C, Sema3E, p-ErbB3, p-ErbB2, p-cMet, p-ERK and p-AKT in C4-2B^{ENZR} and 22Rv1 cells subjected to *SEMA3C* or *SEMA3E* siRNA treatment (10 μM, 48 hrs). (**B**) Western blot of p-ErbB3, p-ErbB2, p-cMet, p-ERK and p-AKT in C4-2B^{ENZR} and 22Rv1 cells upon on treatment with recombinant Sema3C or Sema3E (200-500 ng/μl, 48 hrs) proteins.

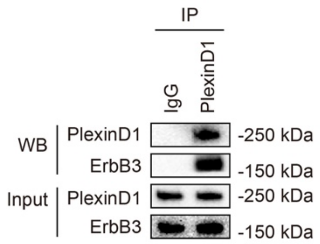

Appendix Figure S10. PlexinD1 directly interacts with ErbB3.
A co-IP assay of PlexinD1-ErbB3 interaction with both recombinant proteins co-incubated in solution. IgG was used in the IP step as negative control.

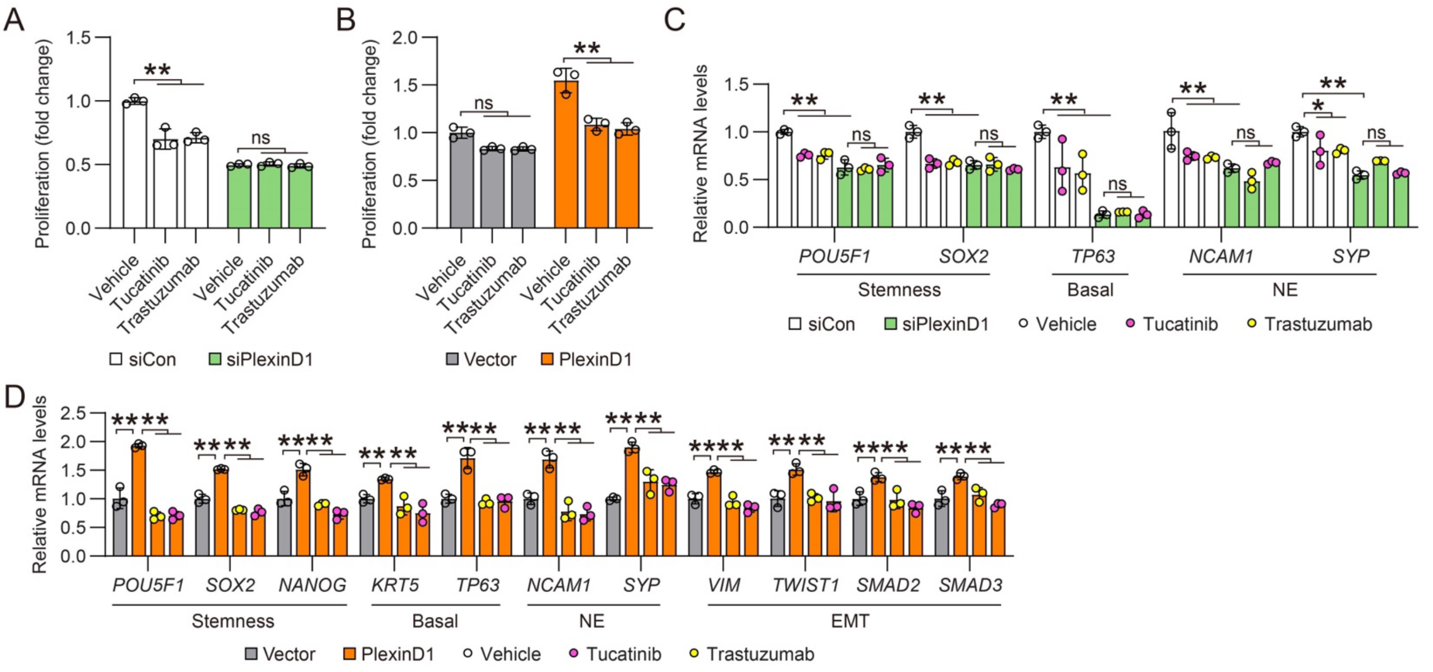

Appendix Figure S11. ErbB2 mediates PlexinD1's effects on CRPC cellular proliferation and plasticity. (**A**, **B**) Cell proliferation assays of 22Rv1 cells pre-treated with 100 nM tucatinib or 10 μg/ml trastuzumab for 24 hrs and then subjected to *PLXND1* siRNA addition followed by a 3-day observation period (A), and control and PlexinD1-overexpressing LNCaP cells upon treatment with tucatinib or trastuzumab during a 3-day observation period (B). Data represent the fold changes of cell proliferation on day 3 relative to treatment day (day 1) for both 22Rv1 and LNCaP cells (*n*=3 biological replicates), with fold changes in non-treated control cells set as 1 for normalization of other groups. (**C**, **D**) qPCR of stemness, basal and NE markers as indicated in 22Rv1 cells receiving 100 nM tucatinib or 10 μg/ml trastuzumab for 24 hrs followed by *PLXND1* siRNA treatment for another 48 hrs (C, *n*=3 biological replicates), and in control and PlexinD1-overexpressing LNCaP cells upon treatment with 100 nM tucatinib or 10 μg/ml trastuzumab for 48 hrs (D, *n*=3 biological replicates). Data information: In (A-D), data are presented as mean ± SEM. *P* values were determined by one-way ANOVA with Tukey's multiple comparisons test. *$P<0.05$, **$P<0.01$; ns, not significant. Exact *P* values are listed in **Appendix Table S4**.

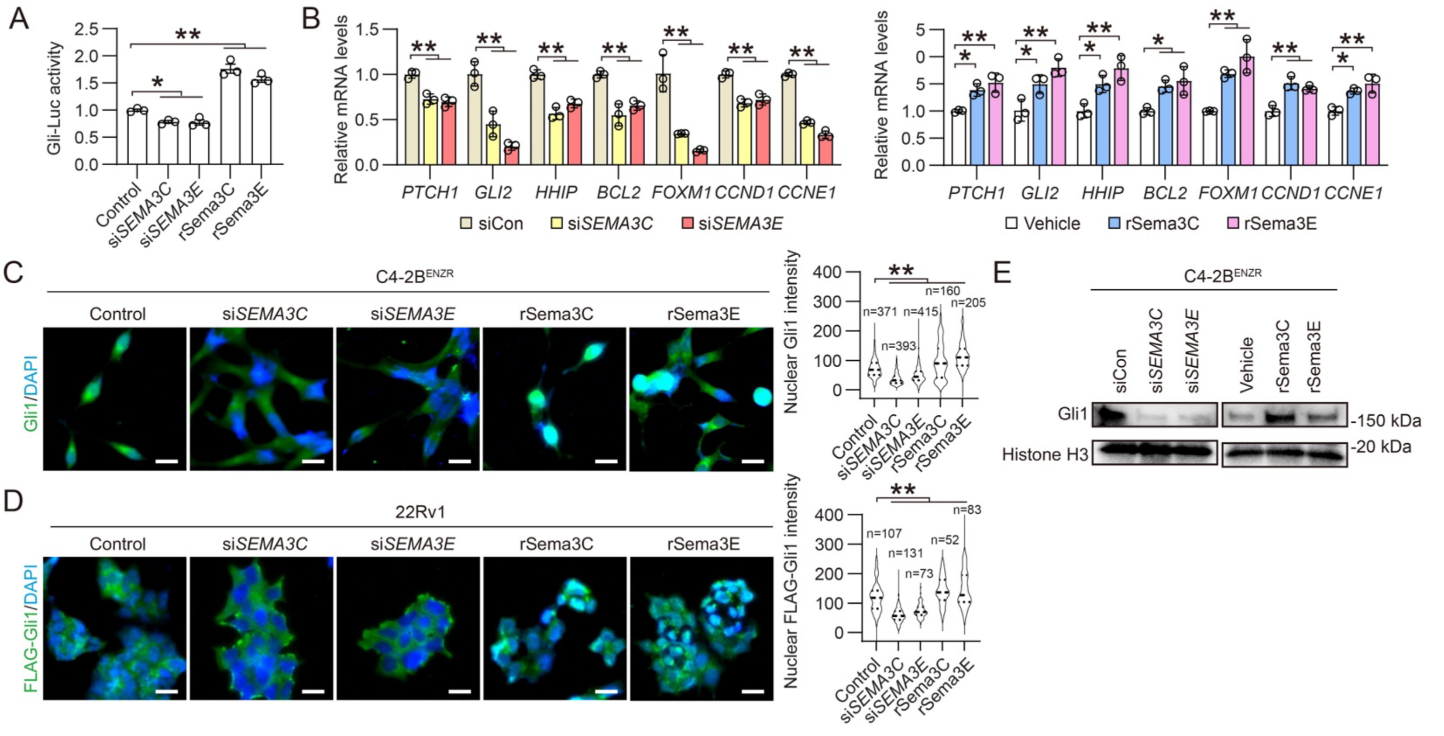

Appendix Figure S12. Sema3 ligands activate Gli1 transcriptional activity and nuclear transloction in PCa cells.

(**A**) Determination of Gli-Luc reporter activity in C4-2B^{ENZR} cells upon treatment with *SEMA3C*/*SEMA3E* siRNA (10 μM, 48 hrs) or recombinant Sema3C/Sema3E proteins (200-500 ng/µl, 48 hrs) (n=3 biological replicates). (**B**) qPCR of Gli1 target genes in C4-2B^{ENZR} cells upon treatment with *SEMA3C*/*SEMA3E* siRNA (10 μM, 48 hrs) or recombinant Sema3C/Sema3E proteins (200-500 ng/µl, 48hr) (n=3 biological replicates). (**C**) Representative images of Gli1 IF staining and quantification of nuclear Gli1 staining intensity in C4-2B^{ENZR} cells upon treatment with *SEMA3C*/*SEMA3E* siRNA (10 μM, 48 hrs) or recombinant Sema3C/Sema3E proteins (200-500 ng/µl, 48 hrs) (n=3 biological replicates). (**D**) Representative images of FLAG-tagged Gli1 IF staining with an anti-FLAG antibody and quantification of nuclear FLAG-Gli1 staining intensity in 22Rv1 cells upon treatment with *SEMA3C*/*SEMA3E* siRNA (10 μM, 48 hrs) or recombinant Sema3C/Sema3E proteins (200-500 ng/µl, 48 hrs) (n=3 biological replicates). (**E**) Western blot of Gli1 in the nuclear fraction of C4-2B^{ENZR} cells upon treatment with *SEMA3C*/*SEMA3E* siRNA (10 μM, 48 hrs) or recombinant Sema3C/Sema3E proteins (200-500 ng/µl, 48 hrs). Data information: In (A-D), data are represented as mean $\pm$ SEM. P values were determined by one-way ANOVA with Dunnett's multiple comparisons test. *P<0.05, **P<0.01. Exact P values are listed in **Appendix Table S4**.

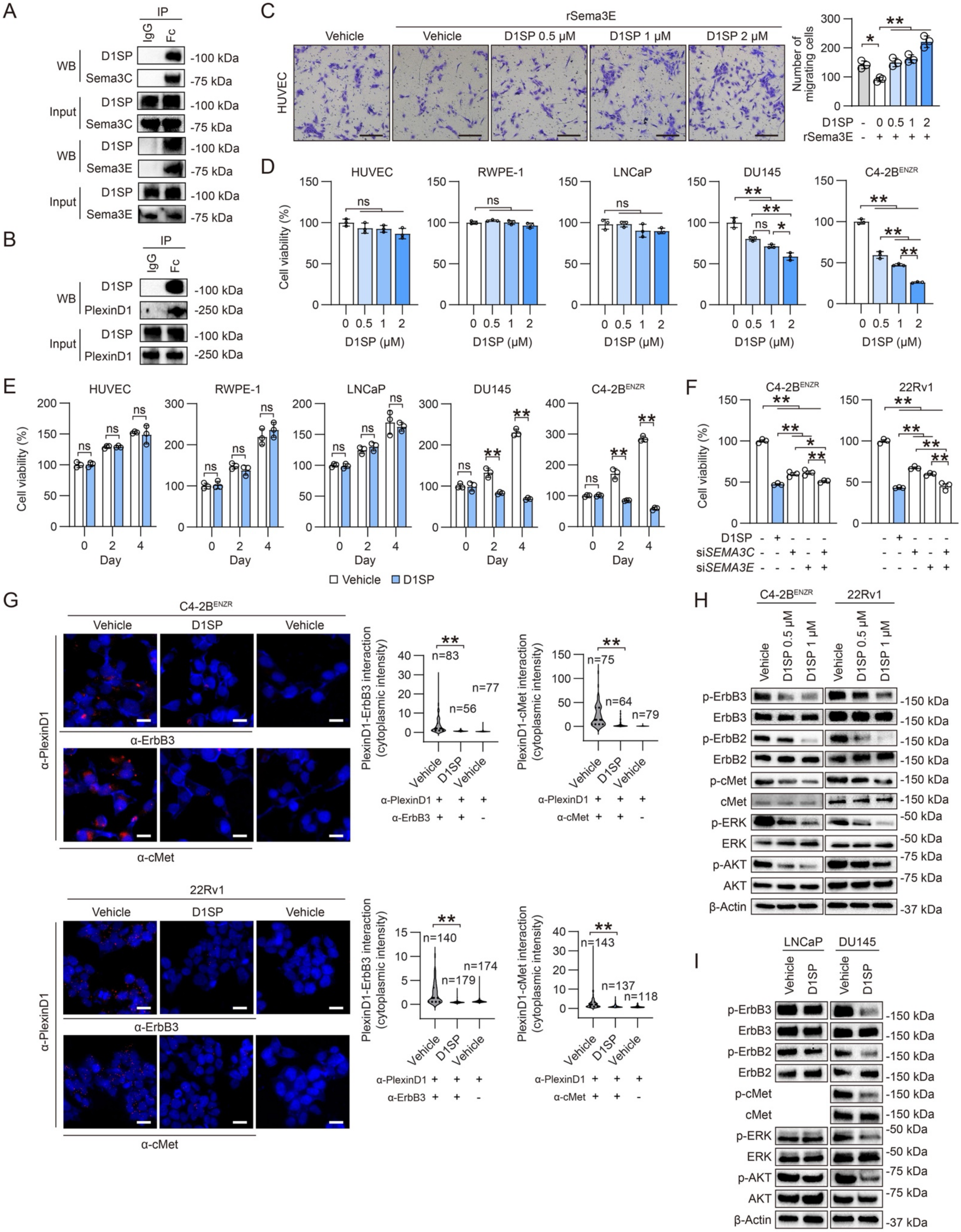
A
IP
IgG
Fc
WB
D1SP
Sema3C
Input
D1SP
Sema3C
WB
D1SP
Sema3E
Input
D1SP
Sema3E
-100 kDa
-75 kDa
B
IP
IgG
Fc
WB
D1SP
PlexinD1
Input
D1SP
PlexinD1
-100 kDa
-250 kDa
C
rSema3E
Vehicle
Vehicle
D1SP 0.5 μM
D1SP 1 μM
D1SP 2 μM
HUVEC
Number of migrating cells
D1SP - 0 0.5 1 2
rSema3E - + + + +
D
HUVEC
RWPE-1
LNCaP
DU145
C4-2B^ENZR
Cell viability (%)
D1SP (μM)
ns
E
HUVEC
RWPE-1
LNCaP
DU145
C4-2B^ENZR
Cell viability (%)
Day
Vehicle
D1SP
F
C4-2B^ENZR
22Rv1
Cell viability (%)
D1SP
siSEMA3C
siSEMA3E
G
C4-2B^ENZR
Vehicle
D1SP
Vehicle
α-PlexinD1
α-ErbB3
α-cMet
PlexinD1-ErbB3 interaction (cytoplasmic intensity)
PlexinD1-cMet interaction (cytoplasmic intensity)
n=83
n=56
n=77
n=75
n=64
n=79
22Rv1
n=140
n=179
n=174
n=143
n=137
n=118
H
C4-2B^ENZR
22Rv1
Vehicle
D1SP 0.5 μM
D1SP 1 μM
p-ErbB3
ErbB3
p-ErbB2
ErbB2
p-cMet
cMet
p-ERK
ERK
p-AKT
AKT
β-Actin
-150 kDa
-50 kDa
-75 kDa
-37 kDa
I
LNCaP
DU145
Vehicle
D1SP

Appendix Figure S13. D1SP interacts with PlexinD1 and its ligands and inhibits PlexinD1-driven CRPC cell proliferation and signaling.

(**A**, **B**) Co-IP assays of D1SP-Sema3C, D1SP-Sema3E (A), and D1SP-PlexinD1 (B) interaction with individual pairs of recombinant proteins co-incubated in solution. An IgGFc-specific antibody was used to precipitate D1SP out of solution. IgG was used in the IP step as negative control. (**C**) Representative images and quantification of transwell-based cell migration by HUVEC cells upon stimulation with 200 ng/μl recombinant Sema3E protein in the absence or presence of D1SP at various concentrations (*n*=3 biological replicates). Scale bars: 20 μm. (**D**, **E**) Cell viability assays of HUVEC, RWPE-1, LNCaP, DU145 and C4-2B^{ENZR} cells upon treatment with 0.5-2 μM D1SP for 3 days (D) or 1 μM D1SP for various days (E) (*n*=3 biological replicates). (**F**) Cell viability assays of C4-2B^{ENZR} and 22Rv1 cells upon treatment with 1 μM D1SP, 10 μM *SEMA3C* siRNA, 10 μM *SEMA3E* siRNA, or 10 μM *SEMA3C*/*SEMA3E* siRNA in combination for 3 days (*n*=3 biological replicates). (**G**) Representative PLA staining and quantification of PlexinD1-ErbB3/cMet interaction by per-cell cytoplasmic fluorescence intensity in C4-2B^{ENZR} and 22Rv1 cells upon D1SP treatment (1 μM, 48 hrs). PlexinD1 antibody incubation alone served as negative controls. Scale bars: 20 μm. **(H)** Western blot of p-ErbB3, p-ErbB2, p-cMet, p-ERK and p-AKT in C4-2B^{ENZR} and 22Rv1 cells upon treatment with D1SP at various concentrations for 1 hr. (**I**) Western blot of PlexinD1, p-ErbB3, p-ErbB2, p-cMet, p-ERK and p-AKT in LNCaP and DU145 cells upon D1SP treatment (1 μM, 1 hr). Data information: In (C-G), data are presented as mean ± SEM. In (C, F, G), *P* values were determined by one-way ANOVA with Dunnett's multiple comparisons test. In (D), *P* values were determined by one-way ANOVA with Tukey's multiple comparisons test. In (E), *P* values were determined by two-way ANOVA with Tukey's multiple comparisons test. **P*<0.05, ***P*<0.01; ns, not significant. Exact *P* values are listed in **Appendix Table S4**.

Gene symbol	Fold change	*P* value
ROBO2	74.20187	1.23E-14
PLXND1	26.94594	1.29E-91
SEMA4G	11.38913	5.76E-43
SEMA6D	5.422579	3.35E-06
EFNB3	5.270347	5.05E-19
EPHB2	4.377016	6.78E-28
NTN3	3.193529	0.016974
ROBO1	2.904445	2.13E-33
UNC5B	2.836456	1.04E-13
SEMA6A	2.314408	4.33E-27
EPHA3	1.979325	5.35E-28
EPHA2	1.887607	1.59E-11
SEMA7A	1.876706	0.001679
EFNA5	1.864531	1.76E-57
SEMA3B	1.66699	1.76E-06
EFNA3	1.644447	1.51E-15
SEMA3F	1.631491	1.89E-18
SEMA6C	1.629772	0.007098
EPHA1	1.550638	2.71E-06
EFNA4	1.513765	5.89E-06
ROBO3	-1.60948	0.000558
EFNB2	-2.73412	4.73E-24
EPHB6	-5.07065	1.74E-06
NTN4	-5.89443	0.000156
PLXNA2	-7.76727	9.43E-05
EPHA6	-35.2058	1.3E-111
UNC5A	-104.793	2.81E-66
UNC5C	-121.613	9.94E-17
SEMA3D	-414.34	7E-100

Appendix Table S1. List of axon guidance pathway genes differentially expressed in C4-2B^{ENZR} vs. LNCaP cells with statistical significance.

Ranking	Protein
1	Tie-1
2	Tyk2
3	ROS
4	RYK
5	PDGFR-α
6	LTK
7	PDGFR-β
8	Lyn
9	TNK1
10	ErbB3

Appendix Table S2. List of top 10 RTKs downregulated in PlexinD1-knockdown vs. control C4-2B^{ENZR} cells as revealed by a RTK phosphorylation antibody array.

Gene name	Forward	Reverse
PLXND1	CCCCAACCCACAGTTCTCTA	CGCAGCTTACTTGGCCTATC
SEMA3C	ATCGAGTGAACGCTGCTGATG	GCTCGCCACTGACAGAGTTGTT
SEMA3E	CAACAGGCACACATGCAA	GTCTTATCCAAAGCATCCC
KLK3	CCAGAGGAGTTCTTGACCCCAAA	CCCCAGAATCACCCGAGCAG
NCAM1	GTCCTGCTCCTGGTGGTTGT	TGACCGCAATGCACATGAA
NRG1	CGGTGTCCATGCCTTCCAT	GTGTCACGAGAAGTAGAGGTCT
NRG2	CCAGAAGAGGGTCCTGACCAT	GCATCTGCTTCCGCTGTTTT
HGF	CTCACACCCGCTGGGAGTAC	TCCTTGACCTTGGATGCATTC
POU5F1	TGTACTCCTCGGTCCCTTTC	TCCAGGTTTTCTTTCCCTAGC
SOX2	GCTAGTCTCCAAGCGACGAA	GCAAGAAGCCTCTCCTTGAA
NANOG	ATGCCTCACACGGAGACTGT	AAGTGGGTTGTTTGCCTTTG
KRT5	TGGAGCTGGTGGTGGCTTTG	GGTCTTGATCTGCTCGCGCT
TP63	GCTCCCCACCTCTGAACAAA	AGTCCATTCATGTCTCCAGCC
SYP	CTACCAGCCTGACTATGG	GGGCTTCACTGACCAGAC
VIM	GAGAACTTTGCCGTTGAAGC	GCTTCCTGTAGGTGGCAATC
TWIST1	GGAGTCCGCAGTCTTACGAG	TCTGGAGGACCTGGTAGAGG
SMAD2	CCGACACACCGAGATCCTAAC	GAGGTGGCGTTTCTGGAATATAA
SMAD3	TGGACGCAGGTTCTCCAAAC	CCGGCTCGCAGTAGGTAAC
PTCH1	AACTCGTCAGCGCCCATGCC	TGGCTGCCGCTTTGTCCTCG
GLI2	AAAGGCCTCTCCTTTGGTGG	CTTCCTTCCTGGTGTCGCAT
HHIP	ACGGTGGGCTATCGGGCCTC	GCCGTCAGGGCCAAAGAGCA
BCL2	TTGCTTTACGTGGCCTGTTTC	GAAGACCCTGAAGGACAGCCAT
FOXM1	GTTTAAGCAGCAGCAGAAACGA	TCACCGGGAACTGGATAGGT
IGFBP6	GAATCCAGGCACCTCTACCA	TGGGCACGTAGAGTGTTTGA
CCND1	TGCCCTCTGTGCCACAGATG	TCTGGAGAGGAAGCGTGTGA
CCNE1	GTTATAAGGGAGACGGGGAG	TGCTCTGCTTCTTACCGCTC
SHH	GGACAGGCTGATGACTCAGA	GCCCTCGTAGTGCAGAGACT
DHH	TGATGACCGAGCGTTGTAAG	GCCAGCAACCCATACTTGTT
WINT5A	GGACCACATGCAGTACATCG	CCTGCCAAAAACAGAGGTGT
ENO2	GCCAAAGGTCTTTTCCGG	CCTTCAGGACACCTTTGC
EZH2	CCCTGACCTCTGTCTTACTTGTGGA	ACGTCAGATGGTGCCAGCAATA
BRN2	TGGGATTTACCCAAGCGGAC	CGCCTGCAAAGGTCAAAACA

Appendix Table S3. Sequences of qPCR primers.

Figure 1

Panel	Lable	Comparison	Symbol	*P* value
Fig. 1F	PlexinD1-Sema3E	LNCaP vs. C4-2B^{ENZR}	**	<0.0001
	PlexinD1-Sema3C	LNCaP vs. C4-2B^{ENZR}	**	<0.0001

Figure 2

Panel	Lable	Comparison	Symbol	*P* value
Fig. 2A		Cancer vs. Normal	*p*<0.01	<0.0001
Fig. 2B		High GS vs. Low GS	*p*<0.05	0.0430
Fig. 2C	GSE3325	Cancer vs. Normal	*	0.0431
	GSE3933	Cancer vs. Normal	*	0.0120
Fig. 2D		Post-hormone therapy vs. Pre-hormone therapy	*p*<0.05	0.0208
Fig. 2E	GSE21032	Recurrence vs. No recurrence	**	0.0020
	GSE6099	Hormone refractory vs. Hormone sensitive	*	0.0169
Fig. 2F		Bone metastasis vs. Primary tumor	*p*<0.01	<0.0001
Fig. 2G	GSE21032	Metastasis vs. Primary site	**	0.0024
	GSE35988	Metastasis vs. Primary site	**	0.0036

Figure 3

Panel	Lable	Comparison	Symbol	*P* value
Fig. 3A	LNCaP - PLXND1	FBS vs. CSS	*	0.0281
		CSS vs. CSS+R1881	*	0.0234
	LNCaP - SEMA3C	FBS vs. CSS	**	<0.0001
		CSS vs. CSS+R1881	**	<0.0001
	LNCaP - SEMA3E	FBS vs. CSS	**	0.0004
		CSS vs. CSS+R1881	**	0.0004
	LNCaP - KLK3	FBS vs. CSS	*	0.0195
		CSS vs. CSS+R1881	**	<0.0001
	LNCaP - NCAM1	FBS vs. CSS	**	<0.0001
		CSS vs. CSS+R1881	**	0.0005
	LAPC4 - PLXND1	FBS vs. CSS	**	<0.0001
		CSS vs. CSS+R1881	**	0.0037
	LAPC4 - SEMA3C	FBS vs. CSS	**	0.0002
		CSS vs. CSS+R1881	**	0.0002
	LAPC4 - SEMA3E	FBS vs. CSS	**	<0.0001
		CSS vs. CSS+R1881	**	0.0002
	LAPC4 - KLK3	FBS vs. CSS	*	0.0359
		CSS vs. CSS+R1881	**	<0.0001
	LAPC4 - NCAM1	FBS vs. CSS	**	<0.0001
		CSS vs. CSS+R1881	**	<0.0001
	VCaP - PLXND1	FBS vs. CSS	**	0.0028
		CSS vs. CSS+R1881	**	0.0079
	VCaP - SEMA3C	FBS vs. CSS	**	0.0002
		CSS vs. CSS+R1881	**	0.0029
	VCaP - SEMA3E	FBS vs. CSS	**	<0.0001
		CSS vs. CSS+R1881	**	<0.0001
	VCaP - KLK3	FBS vs. CSS	**	0.0059
		CSS vs. CSS+R1881	**	<0.0001

	VCaP - NCAM1	FBS vs. CSS	**	0.0017
		CSS vs. CSS+R1881	**	0.0038
Fig. 3E	PLXND1 promoter	AR - Veh vs. R1881	**	0.0010
		IgG - Veh vs. R1881	ns	0.9549
		H3K9ac - Veh vs. R1881	**	0.0007
		IgG - Veh vs. R1881	ns	0.9904
	KLK3 promoter	AR - Veh vs. R1881	**	<0.0001
		IgG - Veh vs. R1881	ns	0.9675
		H3K9ac - Veh vs. R1881	**	0.0005
		IgG - Veh vs. R1881	ns	0.9941
Fig. 3G	WT	Veh vs. R1881	*	0.0283
	Mut ARE 1	Veh vs. R1881	ns	0.8292
	Mut ARE 2	Veh vs. R1881	ns	0.3607
	Mut ARE 3	Veh vs. R1881	ns	0.5711
Fig. 3H	TCGA - PLXND1-AR correlation		*p*<0.01	<0.0001
	SU2C/PCF 2019 - PLXND1-AR correlation		*p*<0.01	<0.0001

Figure 4

Panel	Lable	Comparison	Symbol	*P* value
Fig. 4B	C4-2B^{ENZR}	shPlexinD1#1 vs. shCon	**	0.0007
		shPlexinD1#2 vs. shCon	**	0.0004
	22Rv1	shPlexinD1#1 vs. shCon	**	0.0067
		shPlexinD1#2 vs. shCon	**	0.0029
	LNCaP	PlexinD1 vs. Vector	*	0.0214
Fig. 4C	C4-2B^{ENZR}	shPlexinD1#1 vs. shCon	*	0.0348
		shPlexinD1#2 vs. shCon	*	0.0500
	22Rv1	shPlexinD1#1 vs. shCon	**	0.0004
		shPlexinD1#2 vs. shCon	**	0.0004
	LNCaP	PlexinD1 vs. Vector	**	0.0098
Fig. 4E		shPlexinD1#1 vs. shCon	*p*<0.001	<0.0001
		shPlexinD1#2 vs. shCon	*p*<0.001	<0.0001
Fig. 4I		shPlexinD1#1 vs. shCon	*	0.0142
		shPlexinD1#2 vs. shCon	*	0.0192
Fig. 4J		shPlexinD1#1 vs. shCon	**	0.0040
		shPlexinD1#2 vs. shCon	**	0.0099
Fig. 4L		shPlexinD1#1 vs. shCon	**	<0.0001
		shPlexinD1#2 vs. shCon	**	<0.0001

Figure 5

Panel	Lable	Comparison	Symbol	*P* value
Fig. 5A	C4-2B^{ENZR} - migration	shPlexinD1#1 vs. shCon	**	0.0011
		shPlexinD1#2 vs. shCon	**	0.0037
	22Rv1 - migration	shPlexinD1#1 vs. shCon	**	<0.0001
		shPlexinD1#2 vs. shCon	**	<0.0001
	C4-2B^{ENZR} - invasion	shPlexinD1#1 vs. shCon	**	0.0012
		shPlexinD1#2 vs. shCon	**	0.0020
	22Rv1 - invasion	shPlexinD1#1 vs. shCon	**	0.0086
		shPlexinD1#2 vs. shCon	**	0.0027
Fig. 5B	Migration	PlexinD1 vs. Vector	**	0.0030
	Invasion	PlexinD1 vs. Vector	**	0.0006

Fig. 5E		shPlexinD1 vs. shCon	**	0.0031
Fig. 5G	Liver	shPlexinD1 vs. shCon	**	0.0002
	Adrenal	shPlexinD1 vs. shCon	*	0.0282
	Bone	shPlexinD1 vs. shCon	**	<0.0001
Fig. 5H	Liver	shPlexinD1 vs. shCon	*	0.0119
	Adrenal	shPlexinD1 vs. shCon	*	0.0125

Figure 6

Panel	Lable	Comparison	Symbol	*P* value
Fig. 6B	C4-2B^{ENZR} - number	shPlexinD1#1 vs. shCon	**	0.0014
		shPlexinD1#2 vs. shCon	*	0.0190
	22Rv1 - number	shPlexinD1#1 vs. shCon	**	0.0004
		shPlexinD1#2 vs. shCon	**	<0.0001
	LNCaP - number	PlexinD1 vs. Vector	*	0.0452
	C4-2B^{ENZR} - diameter	shPlexinD1#1 vs. shCon	**	0.0003
		shPlexinD1#2 vs. shCon	**	<0.0001
	22Rv1 - diameter	shPlexinD1#1 vs. shCon	**	<0.0001
		shPlexinD1#2 vs. shCon	**	<0.0001
	LNCaP - diameter	PlexinD1 vs. Vector	**	<0.0001
Fig. 6C	C4-2B^{ENZR}	shPlexinD1 vs. shCon	*	0.0126
	22Rv1	shPlexinD1 vs. shCon	**	<0.0001
	LNCaP	PlexinD1 vs. Vector	**	<0.0001
Fig. 6E	Number	shPlexinD1#1 vs. shCon	**	<0.0001
		shPlexinD1#2 vs. shCon	**	<0.0001
	Length	shPlexinD1#1 vs. shCon	**	<0.0001
		shPlexinD1#2 vs. shCon	**	<0.0001
Fig. 6F	Vector	ENZ vs. Con	**	<0.0001
	PlexinD1	ENZ vs. Con	ns	0.9762
Fig. 6G	PlexinD1-SYP correlation		*p*<0.01	0.0012
Fig. 6H	Trento/Cornell/Beltran 2016	NEPC vs. CRPC Adeno	**	<0.0001
	SU2C/PCF 2019	NEPC vs. Adeno	*	0.0470
	Fred Hutch 2016	NEPC vs. Adeno	**	<0.0001
	GSE32967	NEPC vs. CRPC Adeno	**	<0.0001
	GSE66187	NEPC vs. CRPC Adeno	**	0.0017
	GSE41192	NEPC vs. Adeno	**	0.0023

Figure 7

Panel	Lable	Comparison	Symbol	*P* value
Fig. 7B		shPlexinD1 vs. shCon	*	0.0239
Fig. 7D	C4-2B^{ENZR} - NRG1	shPlexinD1 vs. shCon	ns	0.9146
	C4-2B^{ENZR} - NRG2	shPlexinD1 vs. shCon	ns	0.9520
	C4-2B^{ENZR} - HGF	shPlexinD1 vs. shCon	ns	0.9087
	22Rv1 - NRG1	shPlexinD1 vs. shCon	ns	0.6533
	22Rv1 - NRG2	shPlexinD1 vs. shCon	ns	0.5762
	22Rv1 - HGF	shPlexinD1 vs. shCon	ns	0.6932
Fig. 7E	C4-2B^{ENZR} - PlexinD1-ErbB3	Veh vs. rSema3E	**	<0.0001
		Veh vs. rSema3C	**	<0.0001
	C4-2B^{ENZR} - PlexinD1-cMet	Veh vs. rSema3E	**	<0.0001
		Veh vs. rSema3C	*	0.0178
	22Rv1 - PlexinD1-ErbB3	Veh vs. rSema3E	**	0.0001

		Veh vs. rSema3C	**	<0.0001
	22Rv1 - PlexinD1-cMet	Veh vs. rSema3E	**	<0.0001
		Veh vs. rSema3C	**	<0.0001
Fig. 7G	22Rv1	siCon vs. siCon+α-ErbB3	**	<0.0001
		siCon vs. siCon+cMeti	**	<0.0001
		siCon vs. siPlexinD1	**	<0.0001
		siPlexinD1 vs. siPlexinD1+α-ErbB3	ns	0.8080
		siPlexinD1 vs. siPlexinD1+cMeti	ns	0.9327
	LNCaP	siCon vs. siCon+α-ErbB3	**	0.0012
		siCon vs. siCon+cMeti	**	0.0024
		siCon vs. siPlexinD1	**	<0.0001
		siPlexinD1 vs. siPlexinD1+α-ErbB3	**	<0.0001
		siPlexinD1 vs. siPlexinD1+cMeti	**	<0.0001
Fig. 7H	POU5F1	siCon vs. siCon+a-ErbB3	**	0.0064
		siCon vs. siCon+cMeti	**	0.0059
		siCon vs. siPlexinD1	*	0.0184
		siPlexinD1 vs. siPlexinD1+a-ErbB3	ns	0.9986
		siPlexinD1 vs. siPlexinD1+cMeti	ns	0.8837
		siPlexinD1 vs. siPlexinD1+ErbB3 OE	**	0.0065
		siPlexinD1 vs. siPlexinD1+cMet OE	**	0.0091
	SOX2	siCon vs. siCon+a-ErbB3	*	0.0470
		siCon vs. siCon+cMeti	*	0.0473
		siCon vs. siPlexinD1	*	0.0359
		siPlexinD1 vs. siPlexinD1+a-ErbB3	ns	>0.9999
		siPlexinD1 vs. siPlexinD1+cMeti	ns	0.9062
		siPlexinD1 vs. siPlexinD1+ErbB3 OE	*	0.0175
		siPlexinD1 vs. siPlexinD1+cMet OE	*	0.0149
	NANOG	siCon vs. siCon+a-ErbB3	**	0.0099
		siCon vs. siCon+cMeti	*	0.0387
		siCon vs. siPlexinD1	*	0.0183
		siPlexinD1 vs. siPlexinD1+a-ErbB3	ns	0.9592
		siPlexinD1 vs. siPlexinD1+cMeti	ns	0.6227
		siPlexinD1 vs. siPlexinD1+ErbB3 OE	**	0.0002
		siPlexinD1 vs. siPlexinD1+cMet OE	**	0.0033
	KRT5	siCon vs. siCon+a-ErbB3	*	0.0256
		siCon vs. siCon+cMeti	*	0.0376
		siCon vs. siPlexinD1	*	0.0453
		siPlexinD1 vs. siPlexinD1+a-ErbB3	ns	0.9996
		siPlexinD1 vs. siPlexinD1+cMeti	ns	>0.9999
		siPlexinD1 vs. siPlexinD1+ErbB3 OE	**	0.0065
		siPlexinD1 vs. siPlexinD1+cMet OE	**	0.0019
	TP63	siCon vs. siCon+a-ErbB3	**	<0.0001
		siCon vs. siCon+cMeti	*	0.0318
		siCon vs. siPlexinD1	**	<0.0001
		siPlexinD1 vs. siPlexinD1+a-ErbB3	ns	0.4856
		siPlexinD1 vs. siPlexinD1+cMeti	ns	0.9810
		siPlexinD1 vs. siPlexinD1+ErbB3 OE	**	0.0018
		siPlexinD1 vs. siPlexinD1+cMet OE	**	0.0078
	NCAM1	siCon vs. siCon+a-ErbB3	*	0.0310
		siCon vs. siCon+cMeti	*	0.0479

		siCon vs. siPlexinD1	**	0.0043
		siPlexinD1 vs. siPlexinD1+a-ErbB3	ns	0.9242
		siPlexinD1 vs. siPlexinD1+cMeti	ns	>0.9999
		siPlexinD1 vs. siPlexinD1+ErbB3 OE	**	0.0001
		siPlexinD1 vs. siPlexinD1+cMet OE	**	0.0003
	SYP	siCon vs. siCon+a-ErbB3	*	0.0118
		siCon vs. siCon+cMeti	**	0.0094
		siCon vs. siPlexinD1	**	0.0019
		siPlexinD1 vs. siPlexinD1+a-ErbB3	ns	0.8307
		siPlexinD1 vs. siPlexinD1+cMeti	ns	0.9984
		siPlexinD1 vs. siPlexinD1+ErbB3 OE	**	<0.0001
		siPlexinD1 vs. siPlexinD1+cMet OE	*	0.0155
Fig. 7I	POU5F1	Vector vs. PlexinD1	**	<0.0001
		PlexinD1 vs. PlexinD1+a-ErbB3	**	<0.0001
		PlexinD1 vs. PlexinD1+cMeti	**	<0.0001
	SOX2	Vector vs. PlexinD1	**	0.0003
		PlexinD1 vs. PlexinD1+a-ErbB3	*	0.0124
		PlexinD1 vs. PlexinD1+cMeti	**	0.0008
	NANOG	Vector vs. PlexinD1	**	0.0002
		PlexinD1 vs. PlexinD1+a-ErbB3	**	0.0008
		PlexinD1 vs. PlexinD1+cMeti	**	0.0009
	KRT5	Vector vs. PlexinD1	**	0.0064
		PlexinD1 vs. PlexinD1+a-ErbB3	**	0.0008
		PlexinD1 vs. PlexinD1+cMeti	**	0.0011
	TP63	Vector vs. PlexinD1	**	<0.0001
		PlexinD1 vs. PlexinD1+a-ErbB3	**	<0.0001
		PlexinD1 vs. PlexinD1+cMeti	**	<0.0001
	NCAM1	Vector vs. PlexinD1	**	<0.0001
		PlexinD1 vs. PlexinD1+a-ErbB3	**	0.0008
		PlexinD1 vs. PlexinD1+cMeti	**	<0.0001
	SYP	Vector vs. PlexinD1	*	0.0326
		PlexinD1 vs. PlexinD1+a-ErbB3	**	0.0019
		PlexinD1 vs. PlexinD1+cMeti	**	0.0079
	VIM	Vector vs. PlexinD1	*	0.0265
		PlexinD1 vs. PlexinD1+a-ErbB3	*	0.0112
		PlexinD1 vs. PlexinD1+cMeti	*	0.0163
	TWIST1	Vector vs. PlexinD1	*	0.0120
		PlexinD1 vs. PlexinD1+a-ErbB3	*	0.0172
		PlexinD1 vs. PlexinD1+cMeti	*	0.0283
	SMAD2	Vector vs. PlexinD1	*	0.0331
		PlexinD1 vs. PlexinD1+a-ErbB3	**	0.0038
		PlexinD1 vs. PlexinD1+cMeti	**	0.0038
	SMAD3	Vector vs. PlexinD1	*	0.0200
		PlexinD1 vs. PlexinD1+a-ErbB3	**	0.0017
		PlexinD1 vs. PlexinD1+cMeti	**	0.0026

Figure 8

Panel	Lable	Comparison	Symbol	*P* value
Fig. 8D	C4-2B^{ENZR}	shPlexinD1#1 vs. shCon	**	0.0072
		shPlexinD1#2 vs. shCon	**	0.0068

	22Rv1	shPlexinD1#1 vs. shCon	*	0.0219
		shPlexinD1#2 vs. shCon	**	0.0010
	LNCaP	PlexinD1 vs. Vector	*	0.0284
Fig. 8E	PTCH1	siPlexinD1 vs. siCon	**	0.0010
	GLI2	siPlexinD1 vs. siCon	**	0.0094
	HHIP	siPlexinD1 vs. siCon	*	0.0195
	BCL2	siPlexinD1 vs. siCon	**	0.0071
	FOXM1	siPlexinD1 vs. siCon	*	0.0160
	IGFBP6	siPlexinD1 vs. siCon	**	0.0001
	CCND1	siPlexinD1 vs. siCon	**	0.0019
	CCNE1	siPlexinD1 vs. siCon	**	0.0030
Fig. 8F	C4-2B^{ENZR} - SHH	siPlexinD1 vs. siCon	ns	0.2269
	C4-2B^{ENZR} - DHH	siPlexinD1 vs. siCon	ns	0.8730
	22Rv1 - SHH	siPlexinD1 vs. siCon	ns	0.1554
	22Rv1 - DHH	siPlexinD1 vs. siCon	ns	0.3510
Fig. 8G	22Rv1	siCon vs. siCon+cMeti	**	<0.0001
		siCon vs. siCon+a-ErbB3	**	0.0017
		siCon vs. siPlexinD1	**	0.0006
		siPlexinD1 vs. siPlexinD1+cMeti	ns	0.0657
		siPlexinD1 vs. siPlexinD1+a-ErbB3	ns	>0.9999
		siPlexinD1 vs. siPlexinD1+cMet OE	**	<0.0001
		siPlexinD1 vs. siPlexinD1+ErbB3 OE	**	<0.0001
	LNCaP	PlexinD1 vs. Vector	**	<0.0001
		PlexinD1 vs. PlexinD1+a-ErbB3	**	<0.0001
		PlexinD1 vs. PlexinD1+cMeti	**	<0.0001
Fig. 8H	22Rv1	siCon vs. siPlexinD1	**	<0.0001
		siPlexinD1 vs. siPlexinD1+ErbB3 OE	**	0.0014
		siPlexinD1 vs. siPlexinD1+cMet OE	**	<0.0001
	LNCaP	Vector vs. PlexinD1	**	<0.0001
		PlexinD1 vs. PlexinD1+aHER3	**	<0.0001
		PlexinD1 vs. PlexinD1+cMeti	**	<0.0001
		PlexinD1 vs. PlexinD1+ERKi	**	<0.0001
		PlexinD1 vs. PlexinD1+AKTi	**	0.0005
Fig. 8J	LNCaP	Vector vs. Vector+Cyclopamine	ns	0.7169
		Vector vs. Vector+GANT61	ns	0.6599
		PlexinD1 vs. PlexinD1+Cyclopamine	ns	0.0546
		PlexinD1 vs. PlexinD1+GANT61	**	<0.0001
Fig. 8K	C4-2B^{ENZR}	Veh vs. Cyclopamine	ns	0.3164
		Veh vs. GANT61	**	<0.0001
	22Rv1	Veh vs. Cyclopamine	ns	0.0950
		Veh vs. GANT61	**	<0.0001
Fig. 8L	SOX2	Vector vs. PlexinD1	**	0.0009
		PlexinD1 vs. PlexinD1+GANT61	**	0.0019
	NANOG	Vector vs. PlexinD1	**	0.0002
		PlexinD1 vs. PlexinD1+GANT61	**	0.0004
	WNT5A	Vector vs. PlexinD1	**	0.0005
		PlexinD1 vs. PlexinD1+GANT61	**	0.0005
	NCAM1	Vector vs. PlexinD1	**	0.0042
		PlexinD1 vs. PlexinD1+GANT61	**	0.0042

	ENO2	Vector vs. PlexinD1	**	0.0003
		PlexinD1 vs. PlexinD1+GANT61	*	0.0131
	EZH2	Vector vs. PlexinD1	**	0.0005
		PlexinD1 vs. PlexinD1+GANT61	**	0.0028
	VIM	Vector vs. PlexinD1	**	<0.0001
		PlexinD1 vs. PlexinD1+GANT61	**	0.0010
	TWIST1	Vector vs. PlexinD1	*	0.0102
		PlexinD1 vs. PlexinD1+GANT61	*	0.0333
	SMAD2	Vector vs. PlexinD1	*	0.0126
		PlexinD1 vs. PlexinD1+GANT61	**	0.0085
	SMAD3	Vector vs. PlexinD1	**	0.0088
		PlexinD1 vs. PlexinD1+GANT61	**	0.0042
Fig. 8N		PTCH1 high vs. PTCH1 low	**	0.0005
		GLI2 high vs. GLI2 low	**	<0.0001
		HHIP high vs. HHIP low	**	<0.0001
		BCL2 high vs. BCL2 low	**	0.0018
		IGFBP6 high vs. IGFBP6 low	**	<0.0001

Figure 9

Panel	Lable	Comparison	Symbol	*P* value
Fig. 9C		Veh vs. 0.5 uM D1SP	**	<0.0001
		Veh vs. 1 uM D1SP	**	<0.0001
		Veh vs. 2 uM D1SP	**	<0.0001
Fig. 9D	PlexinD1-Sema3E	Veh vs. D1SP	**	0.0002
	PlexinD1-Sema3C	Veh vs. D1SP	**	<0.0001
Fig. 9E	C4-2B^{ENZR}	Veh vs. D1SP	**	<0.0001
		Veh vs. rSema3E	**	0.0010
		D1SP vs. D1SP+rSema3E	ns	0.1677
		rSema3E vs. D1SP+rSema3E	**	<0.0001
	22Rv1	Veh vs. D1SP	**	<0.0001
		Veh vs. rSema3E	*	0.0216
		D1SP vs. D1SP+rSema3E	ns	0.5352
		rSema3E vs. D1SP+rSema3E	**	<0.0001
Fig. 9F	C4-2B^{ENZR} - migration	Veh vs. D1SP	**	0.0013
	22Rv1 - migration	Veh vs. D1SP	**	0.0002
	C4-2B^{ENZR} - invasion	Veh vs. D1SP	**	0.0013
	22Rv1 - invasion	Veh vs. D1SP	**	0.0012
Fig. 9G	LuCaP 147CR	Veh vs. D1SP	**	<0.0001
	LuCaP 49	Veh vs. D1SP	**	0.0039
	LuCaP 173.1	Veh vs. D1SP	**	0.0014
Fig. 9H		Veh vs. D1SP	*	0.0371
Fig. 9I		Veh vs. D1SP	**	0.0082
Fig. 9L	Ki-67	Veh vs. D1SP	**	0.0058
	p-ErbB3	Veh vs. D1SP	*	0.0261
	p-ErbB2	Veh vs. D1SP	**	0.0005
	p-cMet	Veh vs. D1SP	**	<0.0001
Fig. 9M	PTCH1	Veh vs. D1SP	**	0.0028

	GLI2	Veh vs. D1SP	*	0.0200
	IGFBP6	Veh vs. D1SP	*	0.0130
	CCND1	Veh vs. D1SP	*	0.0369

Appendix Figures S1-S13

Panel	Lable	Comparison	Symbol	*P* value
Fig. S1B	Vanaja	Metastasis vs. Primary site	**	0.0008
	Yu	Metastasis vs. Primary site	**	<0.0001
Fig. S1D	22Rv1	siCon vs. siBRN2	**	0.0044
		siCon vs. siBRN2	*	0.0133
	LNCaP	Vector vs. BRN2	**	0.0002
		Vector vs. BRN2	**	0.0005
	C4-2B^{ENZR}	shCon - Con vs. rSema3C	**	<0.0001
		shCon - Con vs. rSema3E	**	<0.0001
		shCon - rSema3C vs. rSema3E	**	<0.0001
		shPlexinD1 - Con vs. rSema3C	**	<0.0001
		shPlexinD1 - Con vs. rSema3E	ns	0.2283
	22Rv1	shCon - Con vs. rSema3C	**	<0.0001
		shCon - Con vs. rSema3E	**	<0.0001
		shCon - rSema3C vs. rSema3E	**	<0.0001
		shPlexinD1 - Con vs. rSema3C	**	<0.0001
		shPlexinD1 - Con vs. rSema3E	ns	0.8449
Fig. S3B	LNCaP	siCon vs. siPlexinD1	ns	0.4800
	RWPE-1	siCon vs. siPlexinD1	ns	0.8841
	HUVEC	siCon vs. siPlexinD1	ns	0.2012
	C4-2B^{ENZR}	siCon vs. siPlexinD1	**	0.0094
Fig. S4B	PC-3	shPlexinD1#1 vs. shCon	**	0.0002
		shPlexinD1#2 vs. shCon	**	<0.0001
	DU145	shPlexinD1#1 vs. shCon	**	0.0046
		shPlexinD1#2 vs. shCon	**	0.0011
	LASCPC-01	shPlexinD1#1 vs. shCon	**	0.0073
		shPlexinD1#2 vs. shCon	**	<0.0001
Fig. S4C	PC-3	shPlexinD1#1 vs. shCon	**	<0.0001
		shPlexinD1#2 vs. shCon	**	0.0001
	DU145	shPlexinD1#1 vs. shCon	**	<0.0001
		shPlexinD1#2 vs. shCon	**	<0.0001
	LASCPC-01	shPlexinD1#1 vs. shCon	**	0.0016
		shPlexinD1#2 vs. shCon	**	0.0040
Fig. S4D	PC-3	shPlexinD1#1 vs. shCon	**	0.0007
		shPlexinD1#2 vs. shCon	**	0.0001
Fig. S4E	PC-3	shPlexinD1#1 vs. shCon	**	<0.0001
		shPlexinD1#2 vs. shCon	**	<0.0001
Fig. S4F	LASCPC-01	shPlexinD1#1 vs. shCon	**	0.0010
		shPlexinD1#2 vs. shCon	**	<0.0001
Fig. S4G	LASCPC-01	shPlexinD1#1 vs. shCon	*	0.0281
		shPlexinD1#2 vs. shCon	**	0.0008
Fig. S4H	PC-3	shPlexinD1#1 vs. shCon	**	<0.0001
		shPlexinD1#2 vs. shCon	**	<0.0001
	LASCPC-01	shPlexinD1#1 vs. shCon	**	<0.0001
		shPlexinD1#2 vs. shCon	**	0.0008

Fig. S5A		Vector vs. PlexinD1	$p<0.01$	0.0017
Fig. S5B		Vector vs. PlexinD1	*	0.0490
Fig. S5C		Vector vs. PlexinD1	*	0.0356
Fig. S5E		Vector vs. PlexinD1	**	0.0091
Fig. S6A	PC-3 - migration	shPlexinD1#1 vs. shCon	**	<0.0001
		shPlexinD1#2 vs. shCon	**	<0.0001
	$ARCaP_M$ - migration	shPlexinD1#1 vs. shCon	**	<0.0001
		shPlexinD1#2 vs. shCon	**	<0.0001
	PC-3 - invasion	shPlexinD1#1 vs. shCon	**	<0.0001
		shPlexinD1#2 vs. shCon	**	<0.0001
	$ARCaP_M$ - invasion	shPlexinD1#1 vs. shCon	**	<0.0001
		shPlexinD1#2 vs. shCon	**	<0.0001
Fig. S6B		shPlexinD1#1 vs. shCon	**	<0.0001
		shPlexinD1#2 vs. shCon	**	<0.0001
Fig. S7A		Vector vs. PlexinD1	**	0.0084
Fig. S8C	Number	shPlexinD1 vs. shCon	**	<0.0001
	Length	shPlexinD1 vs. shCon	**	<0.0001
Fig. S11A	siCon	Veh vs. Tucatinib	**	<0.0001
		Veh vs. Trastuzumab	**	<0.0001
	siPlexinD1	Veh vs. Tucatinib	ns	0.9999
		Veh vs. Trastuzumab	ns	>0.9999
Fig. S11B	Vector	Veh vs. Tucatinib	ns	0.0996
		Veh vs. Trastuzumab	ns	0.0933
	PlexinD1	Veh vs. Tucatinib	**	<0.0001
		Veh vs. Trastuzumab	**	<0.0001
Fig. S11C	POU5F1	siCon vs. siCon+Tucatinib	**	0.0067
		siCon vs. siCon+Trastuzumab	**	0.0070
		siCon vs. siPlexinD1	**	<0.0001
		siPlexinD1 vs. siPlexinD1+Tucatinib	ns	0.9997
		siPlexinD1 vs. siPlexinD1+Trastuzumab	ns	0.9989
	SOX2	siCon vs. siCon+Tucatinib	**	<0.0001
		siCon vs. siCon+Trastuzumab	**	0.0001
		siCon vs. siPlexinD1	**	<0.0001
		siPlexinD1 vs. siPlexinD1+Tucatinib	ns	>0.9999
		siPlexinD1 vs. siPlexinD1+Trastuzumab	ns	0.9810
	TP63	siCon vs. siCon+Tucatinib	**	<0.0001
		siCon vs. siCon+Trastuzumab	**	<0.0001
		siCon vs. siPlexinD1	**	<0.0001
		siPlexinD1 vs. siPlexinD1+Tucatinib	ns	0.9990
		siPlexinD1 vs. siPlexinD1+Trastuzumab	ns	>0.9999
	NCAM1	siCon vs. siCon+Tucatinib	**	0.0022
		siCon vs. siCon+Trastuzumab	**	0.0010
		siCon vs. siPlexinD1	**	<0.0001
		siPlexinD1 vs. siPlexinD1+Tucatinib	ns	0.3508
		siPlexinD1 vs. siPlexinD1+Trastuzumab	ns	0.9596
	SYP	siCon vs. siCon+Tucatinib	*	0.0498
		siCon vs. siCon+Trastuzumab	*	0.0468
		siCon vs. siPlexinD1	**	<0.0001
		siPlexinD1 vs. siPlexinD1+Tucatinib	ns	0.2526
		siPlexinD1 vs. siPlexinD1+Trastuzumab	ns	0.9996

Fig. S11D	POU5F1	Vector vs. PlexinD1	**	<0.0001
		PlexinD1 vs. PlexinD1+Tucatinib	**	<0.0001
		PlexinD1 vs. PlexinD1+Trastuzumab	**	<0.0001
	SOX2	Vector vs. PlexinD1	**	<0.0001
		PlexinD1 vs. PlexinD1+Tucatinib	**	<0.0001
		PlexinD1 vs. PlexinD1+Trastuzumab	**	<0.0001
	NANOG	Vector vs. PlexinD1	**	<0.0001
		PlexinD1 vs. PlexinD1+Tucatinib	**	<0.0001
		PlexinD1 vs. PlexinD1+Trastuzumab	**	<0.0001
	KRT5	Vector vs. PlexinD1	**	0.0011
		PlexinD1 vs. PlexinD1+Tucatinib	**	<0.0001
		PlexinD1 vs. PlexinD1+Trastuzumab	**	<0.0001
	TP63	Vector vs. PlexinD1	**	<0.0001
		PlexinD1 vs. PlexinD1+Tucatinib	**	<0.0001
		PlexinD1 vs. PlexinD1+Trastuzumab	**	<0.0001
	NCAM1	Vector vs. PlexinD1	**	<0.0001
		PlexinD1 vs. PlexinD1+Tucatinib	**	<0.0001
		PlexinD1 vs. PlexinD1+Trastuzumab	**	<0.0001
	SYP	Vector vs. PlexinD1	**	<0.0001
		PlexinD1 vs. PlexinD1+Tucatinib	**	<0.0001
		PlexinD1 vs. PlexinD1+Trastuzumab	**	<0.0001
	VIM	Vector vs. PlexinD1	**	<0.0001
		PlexinD1 vs. PlexinD1+Tucatinib	**	<0.0001
		PlexinD1 vs. PlexinD1+Trastuzumab	**	<0.0001
	TWIST1	Vector vs. PlexinD1	**	<0.0001
		PlexinD1 vs. PlexinD1+Tucatinib	**	<0.0001
		PlexinD1 vs. PlexinD1+Trastuzumab	**	<0.0001
	SMAD2	Vector vs. PlexinD1	**	0.0003
		PlexinD1 vs. PlexinD1+Tucatinib	**	0.0002
		PlexinD1 vs. PlexinD1+Trastuzumab	**	<0.0001
	SMAD3	Vector vs. PlexinD1	**	0.0002
		PlexinD1 vs. PlexinD1+Tucatinib	**	0.0031
		PlexinD1 vs. PlexinD1+Trastuzumab	**	<0.0001
Fig. S12A		Con vs. siSEMA3C	*	0.0407
		Con vs. siSEMA3E	*	0.0321
		Con vs. rSema3C	**	<0.0001
		Con vs. rSema3E	**	<0.0001
Fig. S12B	PTCH1	siCon vs. siSEMA3C	**	0.0011
		siCon vs. siSEMA3E	**	0.0006
	GLI2	siCon vs. siSEMA3C	**	0.0020
		siCon vs. siSEMA3E	**	0.0003
	HHIP	siCon vs. siSEMA3C	**	0.0002
		siCon vs. siSEMA3E	**	0.0007
	BCL2	siCon vs. siSEMA3C	**	0.0008
		siCon vs. siSEMA3E	**	0.0032
	FOXM1	siCon vs. siSEMA3C	**	0.0008
		siCon vs. siSEMA3E	**	0.0002
	CCND1	siCon vs. siSEMA3C	**	0.0004
		siCon vs. siSEMA3E	**	0.0008
	CCNE1	siCon vs. siSEMA3C	**	<0.0001

		siCon vs. siSEMA3E	**	<0.0001
	PTCH1	Veh vs. rSema3C	*	0.0144
		Veh vs. rSema3E	**	0.0035
	GLI2	Veh vs. rSema3C	*	0.0297
		Veh vs. rSema3E	**	0.0031
	HHIP	Veh vs. rSema3C	*	0.0253
		Veh vs. rSema3E	**	0.0029
	BCL2	Veh vs. rSema3C	*	0.0273
		Veh vs. rSema3E	*	0.0116
	FOXM1	Veh vs. rSema3C	**	0.0064
		Veh vs. rSema3E	**	0.0009
	CCND1	Veh vs. rSema3C	**	0.0011
		Veh vs. rSema3E	**	0.0034
	CCNE1	Veh vs. rSema3C	*	0.0131
		Veh vs. rSema3E	**	0.0028
Fig. S12C		Control vs. siSEMA3C	**	<0.0001
		Control vs. siSEMA3E	**	<0.0001
		Control vs. rSEMA3C	**	<0.0001
		Control vs. rSEMA3E	**	<0.0001
Fig. S12D		Control vs. siSEMA3C	**	<0.0001
		Control vs. siSEMA3E	**	<0.0001
		Control vs. rSema3C	**	0.0096
		Control vs. rSema3E	**	<0.0001
Fig. S13C		Veh vs. rSema3E	*	0.0106
		rSema3E vs. rSema3E+D1SP 0.5 uM	**	0.0038
		rSema3E vs. rSema3E+D1SP 1 uM	**	0.0009
		rSema3E vs. rSema3E+D1SP 2 uM	**	<0.0001
Fig. S13D	LNCaP	Veh vs. D1SP 0.5 uM	ns	>0.9999
		Veh vs. D1SP 1 uM	ns	0.3647
		Veh vs. D1SP 2 uM	ns	0.3315
	RWPE-1	Veh vs. D1SP 0.5 uM	ns	0.4288
		Veh vs. D1SP 1 uM	ns	0.9981
		Veh vs. D1SP 2 uM	ns	0.1920
	HUVEC	Veh vs. D1SP 0.5 uM	ns	>0.9999
		Veh vs. D1SP 1 uM	ns	0.3647
		Veh vs. D1SP 2 uM	ns	0.3315
	DU145	Veh vs. D1SP 0.5 uM	**	0.0016
		Veh vs. D1SP 1 uM	**	0.0001
		Veh vs. D1SP 2 uM	**	<0.0001
		D1SP 0.5 uM vs. D1SP 1 uM	ns	0.1047
		D1SP 0.5 uM vs. D1SP 2 uM	**	0.0009
		D1SP 1 uM vs. D1SP 2 uM	*	0.0242
	C4-2B^{ENZR}	Veh vs. D1SP 0.5 uM	**	<0.0001
		Veh vs. D1SP 1 uM	**	<0.0001
		Veh vs. D1SP 2 uM	**	<0.0001
		D1SP 0.5 uM vs. D1SP 1 uM	**	0.0024
		D1SP 0.5 uM vs. D1SP 2 uM	**	<0.0001
		D1SP 1 uM vs. D1SP 2 uM	**	<0.0001
Fig. S13E	LNCaP	Day 0 Veh vs. D1SP	ns	0.9973
		Day 2 Veh vs. D1SP	ns	0.8503

		Day 4 Veh vs. D1SP	ns	0.7532
	RWPE-1	Day 0 Veh vs. D1SP	ns	0.9939
		Day 2 Veh vs. D1SP	ns	0.7183
		Day 4 Veh vs. D1SP	ns	0.3614
	HUVEC	Day 0 Veh vs. D1SP	ns	0.9996
		Day 2 Veh vs. D1SP	ns	>0.9999
		Day 4 Veh vs. D1SP	ns	0.8938
	DU145	Day 0 Veh vs. D1SP	ns	0.9967
		Day 2 Veh vs. D1SP	**	<0.0001
		Day 4 Veh vs. D1SP	**	<0.0001
	C4-2B^{ENZR}	Day 0 Veh vs. D1SP	ns	0.9994
		Day 2 Veh vs. D1SP	**	<0.0001
		Day 4 Veh vs. D1SP	**	<0.0001
Fig. S13F	C4-2B^{ENZR}	Con vs. D1SP	**	<0.0001
		Con vs. siSEMA3C	**	<0.0001
		Con vs. siSEMA3E	**	<0.0001
		Con vs. siSEMA3C+siSEMA3E	**	<0.0001
		D1SP vs. siSEMA3C	**	0.0014
		D1SP vs. siSEMA3E	**	0.0005
		siSEMA3C vs. siSEMA3C+siSEMA3E	*	0.0159
		siSEMA3E vs. siSEMA3C+siSEMA3E	**	0.0046
	22Rv1	Con vs. D1SP	**	<0.0001
		Con vs. siSEMA3C	**	<0.0001
		Con vs. siSEMA3E	**	<0.0001
		Con vs. siSEMA3C+siSEMA3E	**	<0.0001
		D1SP vs. siSEMA3C	**	<0.0001
		D1SP vs. siSEMA3E	**	0.0003
		siSEMA3C vs. siSEMA3C+siSEMA3E	**	<0.0001
		siSEMA3E vs. siSEMA3C+siSEMA3E	**	0.0008
Fig. S13G	C4-2B^{ENZR} PlexinD1-ErbB3	Veh vs. D1SP	**	<0.0001
	C4-2B^{ENZR} PlexinD1-cMet	Veh vs. D1SP	**	<0.0001
	22Rv1 PlexinD1-ErbB3	Veh vs. D1SP	**	<0.0001
	22Rv1 PlexinD1-cMet	Veh vs. D1SP	**	<0.0001

Appendix Table S4. Exact *P* values for statistical analyses.
