# Supplementary figures and images for: PlexinD1 is a driver and a therapeutic target in advanced prostate cancer (part 1 of 2)
Source: EMBO Mol Med. 2025 Jan 2;17(2):336–64. doi: 10.1038/s44321-024-00186-z (PMC11822115; doi:10.1038/s44321-024-00186-z)

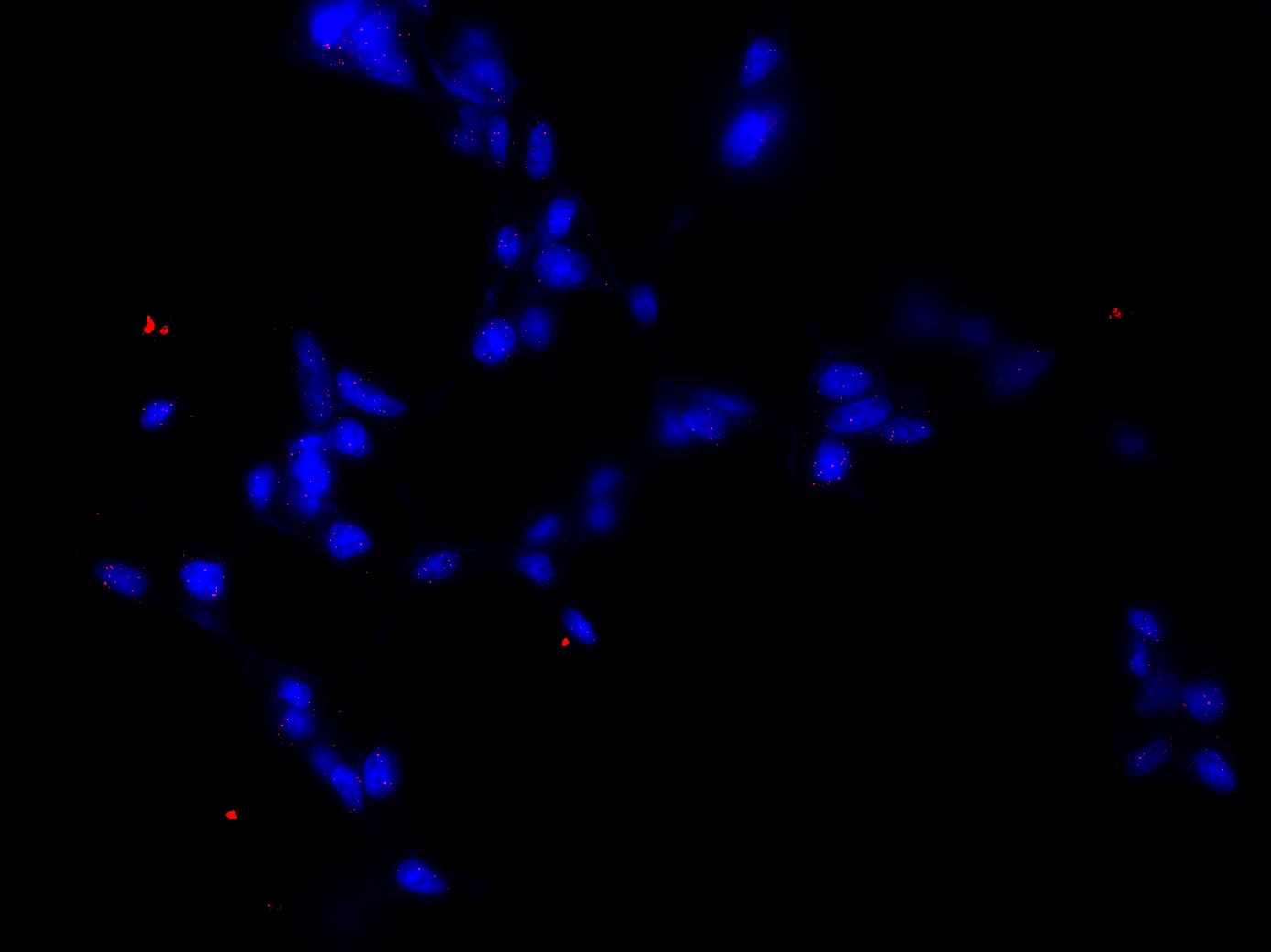

Supplement: Supplementary file 3 — Source data Fig. 1 [file 44321_2024_186_MOESM3_ESM.zip › Figure 1/1F/aPlexinD1-aSEMA3C_LNCaP.jpg]

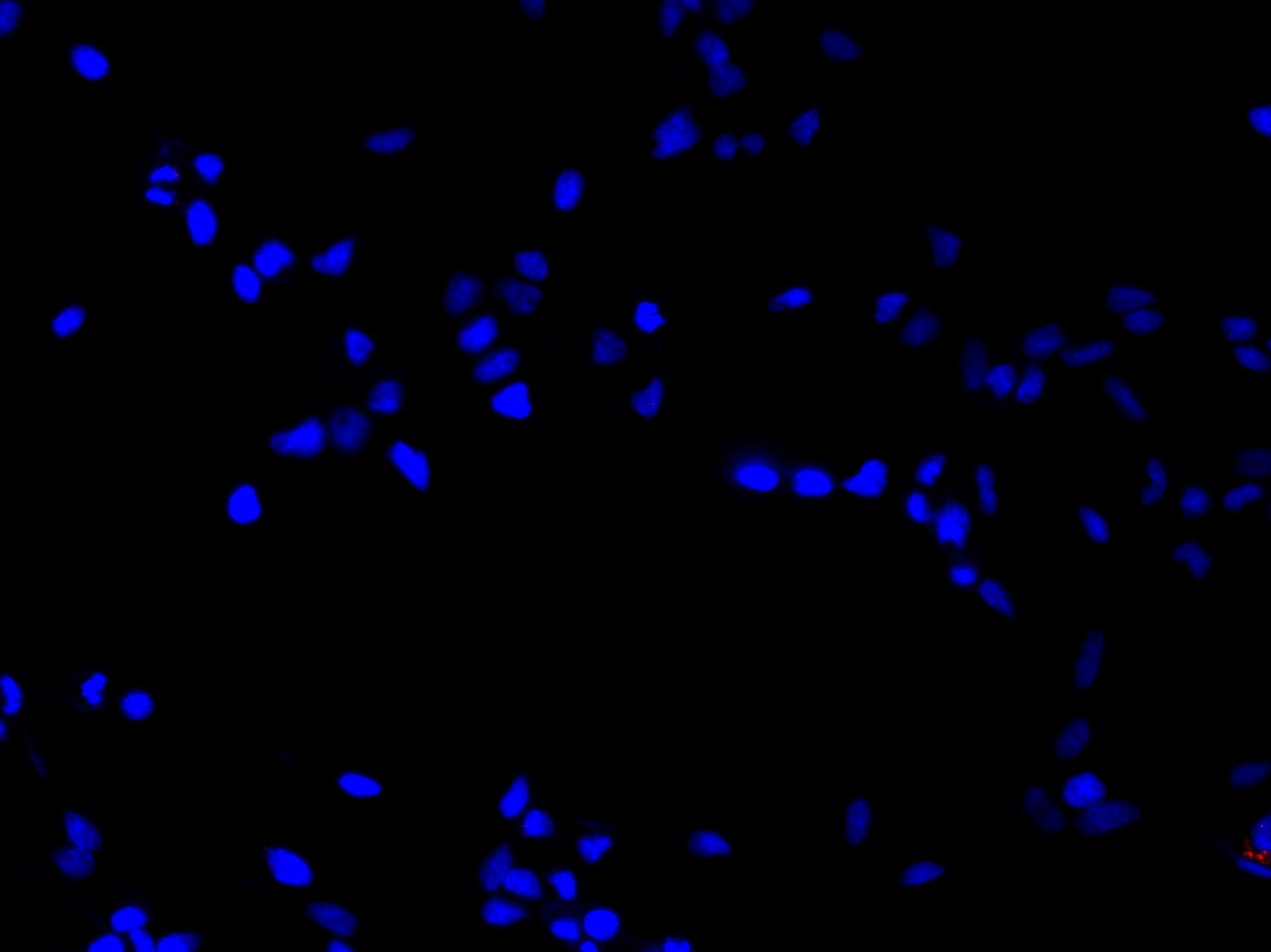

Supplement: Supplementary file 3 — Source data Fig. 1 [file 44321_2024_186_MOESM3_ESM.zip › Figure 1/1F/aPlexinD1-aSEMA3E_LNCaP.jpg]

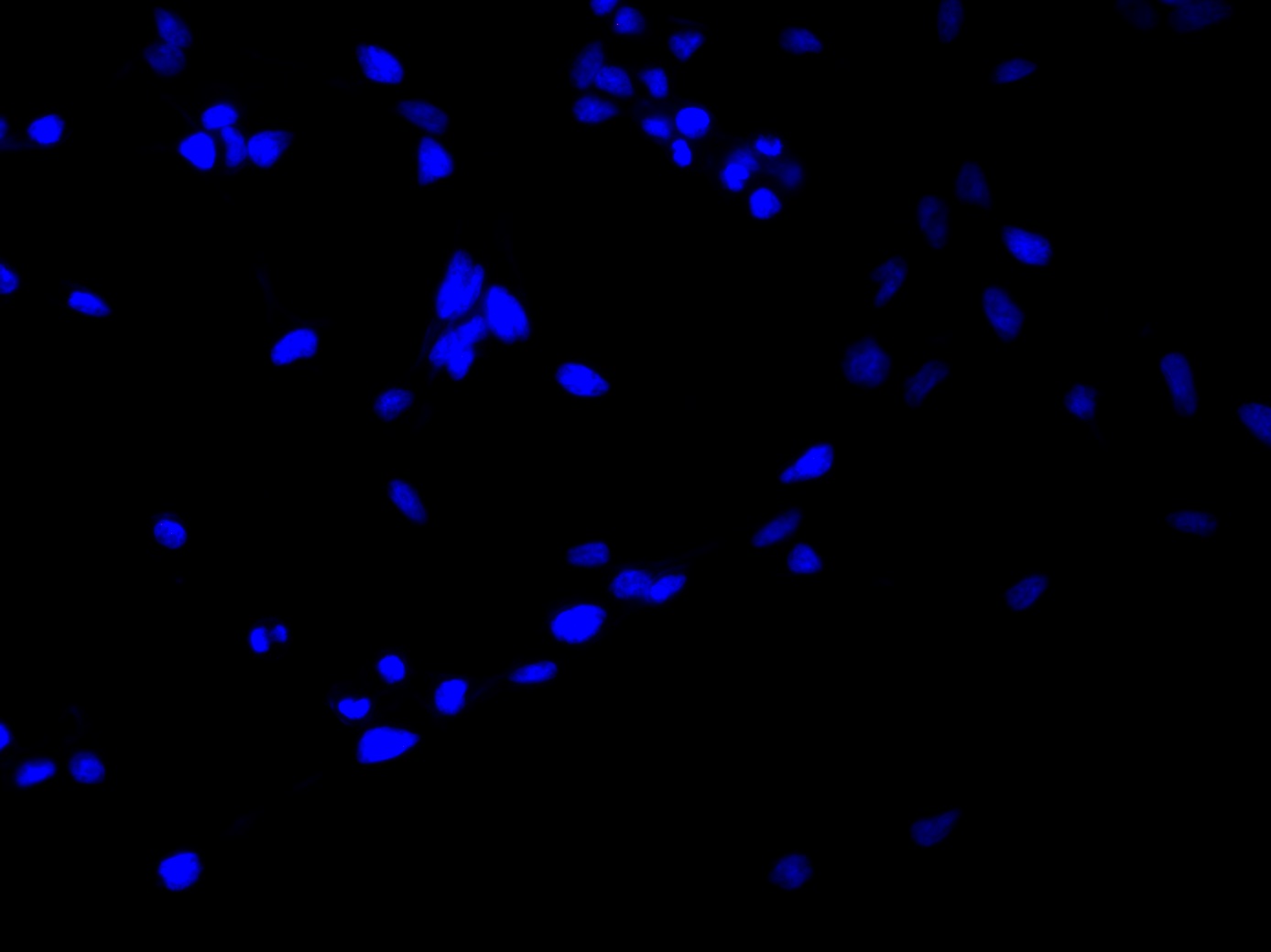

Supplement: Supplementary file 3 — Source data Fig. 1 [file 44321_2024_186_MOESM3_ESM.zip › Figure 1/1F/aPlexinD1-aSEMA3E_C42BENZR-neg.jpg]

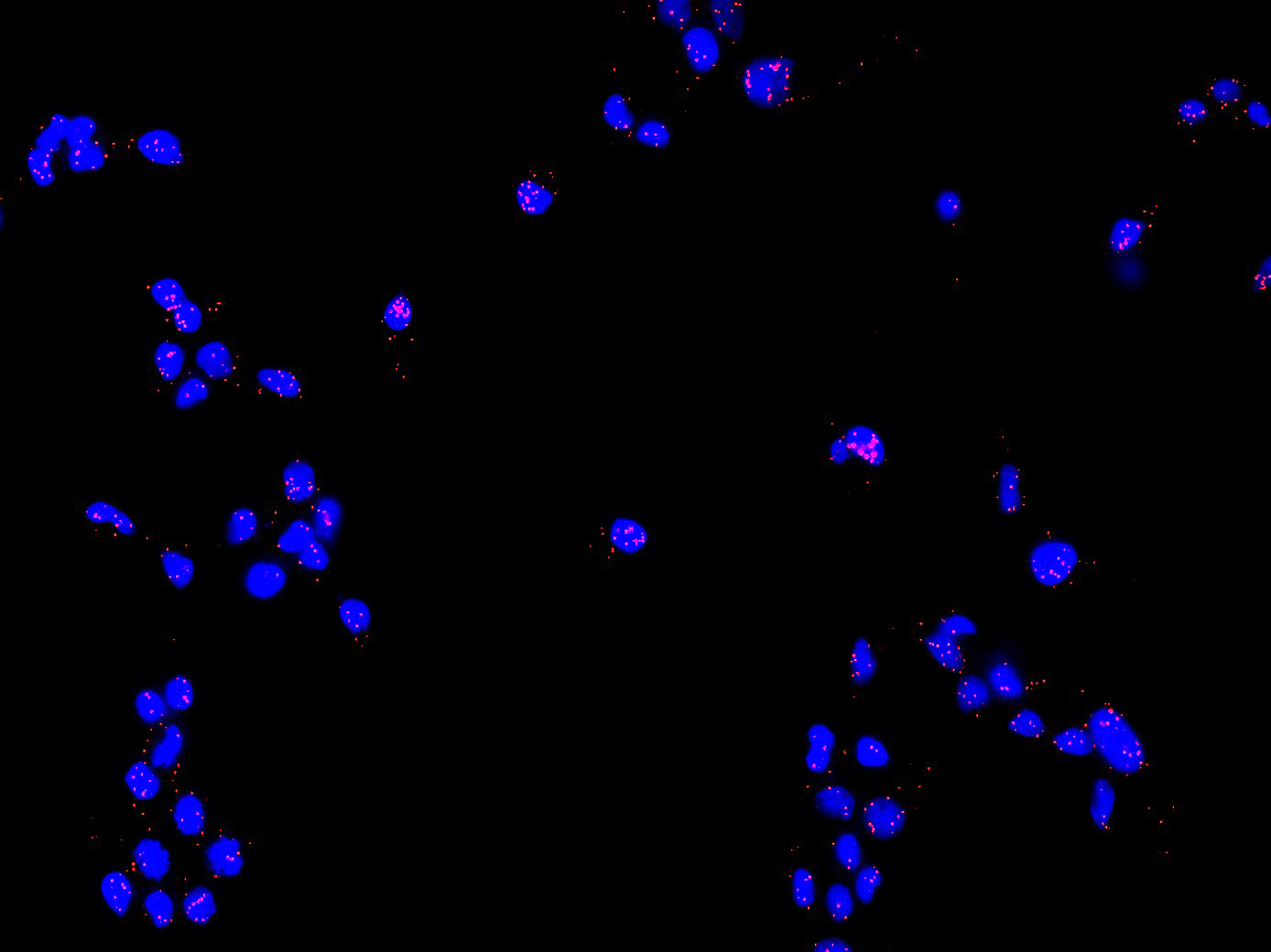

Supplement: Supplementary file 3 — Source data Fig. 1 [file 44321_2024_186_MOESM3_ESM.zip › Figure 1/1F/aPlexinD1-aSEMA3E_C42BENZR.jpg]

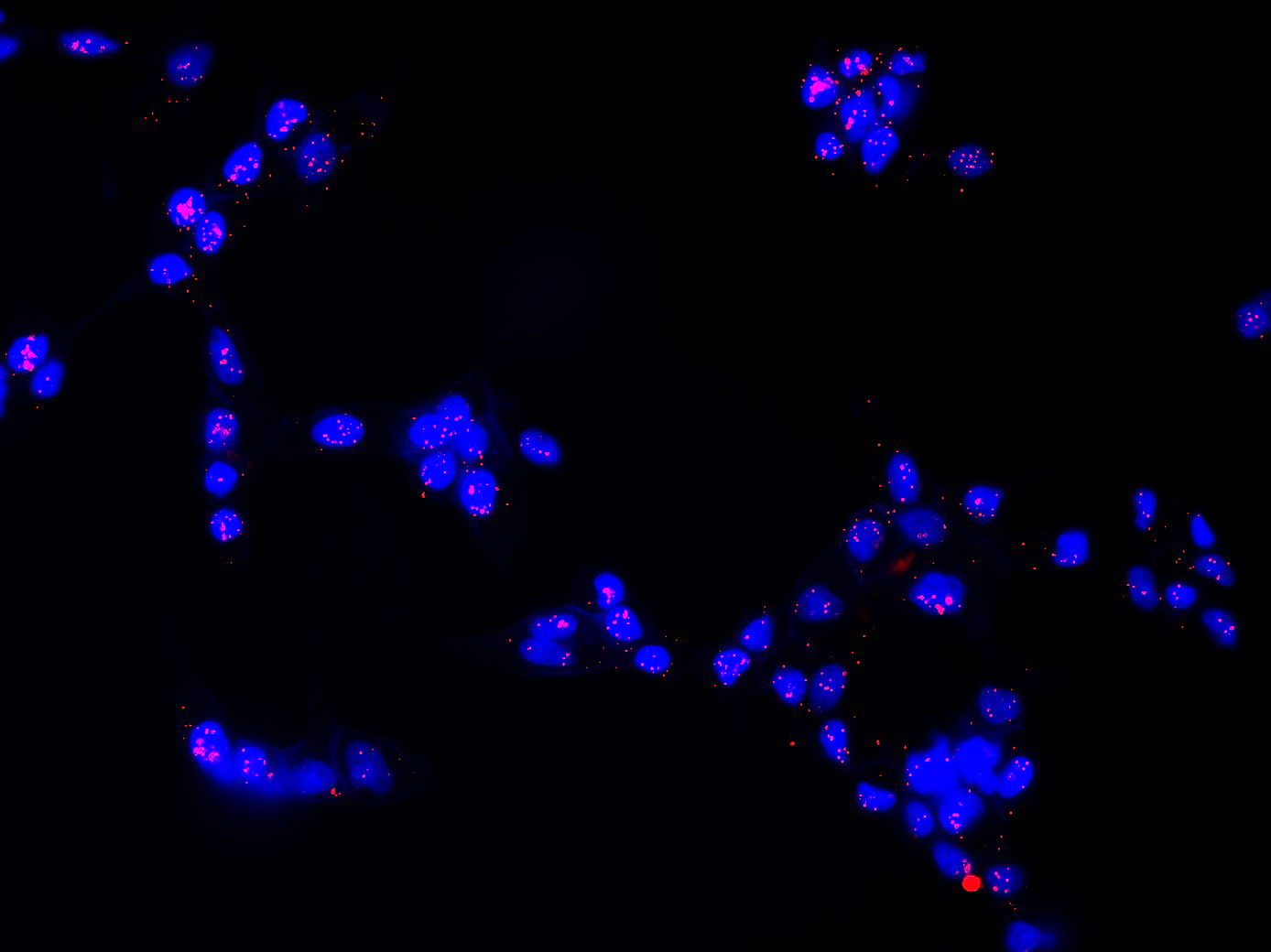

Supplement: Supplementary file 3 — Source data Fig. 1 [file 44321_2024_186_MOESM3_ESM.zip › Figure 1/1F/aPlexinD1-aSEMA3C_C42BENZR.jpg]

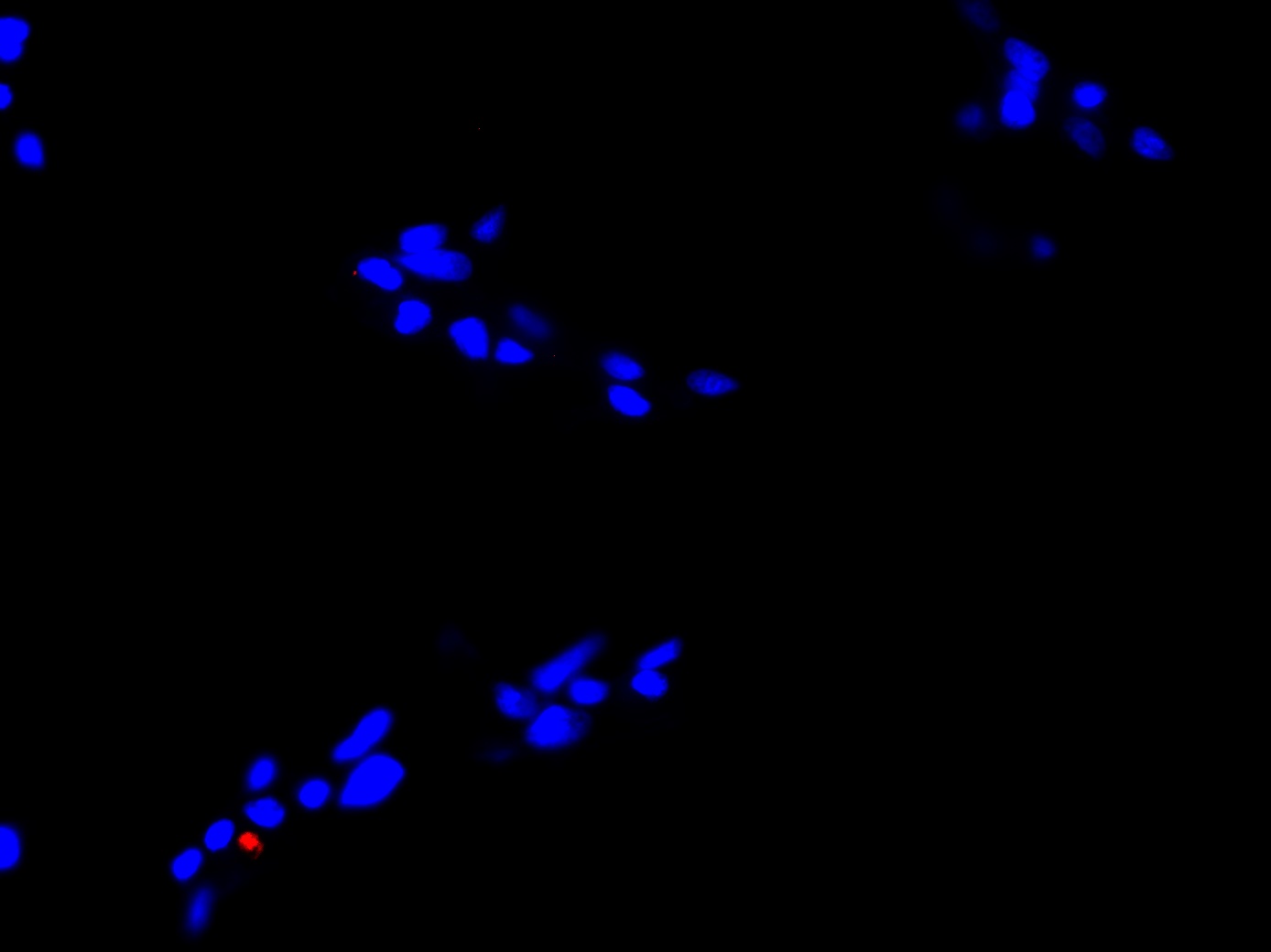

Supplement: Supplementary file 3 — Source data Fig. 1 [file 44321_2024_186_MOESM3_ESM.zip › Figure 1/1F/aPlexinD1-aSEMA3C_C42BENZR-neg.jpg]

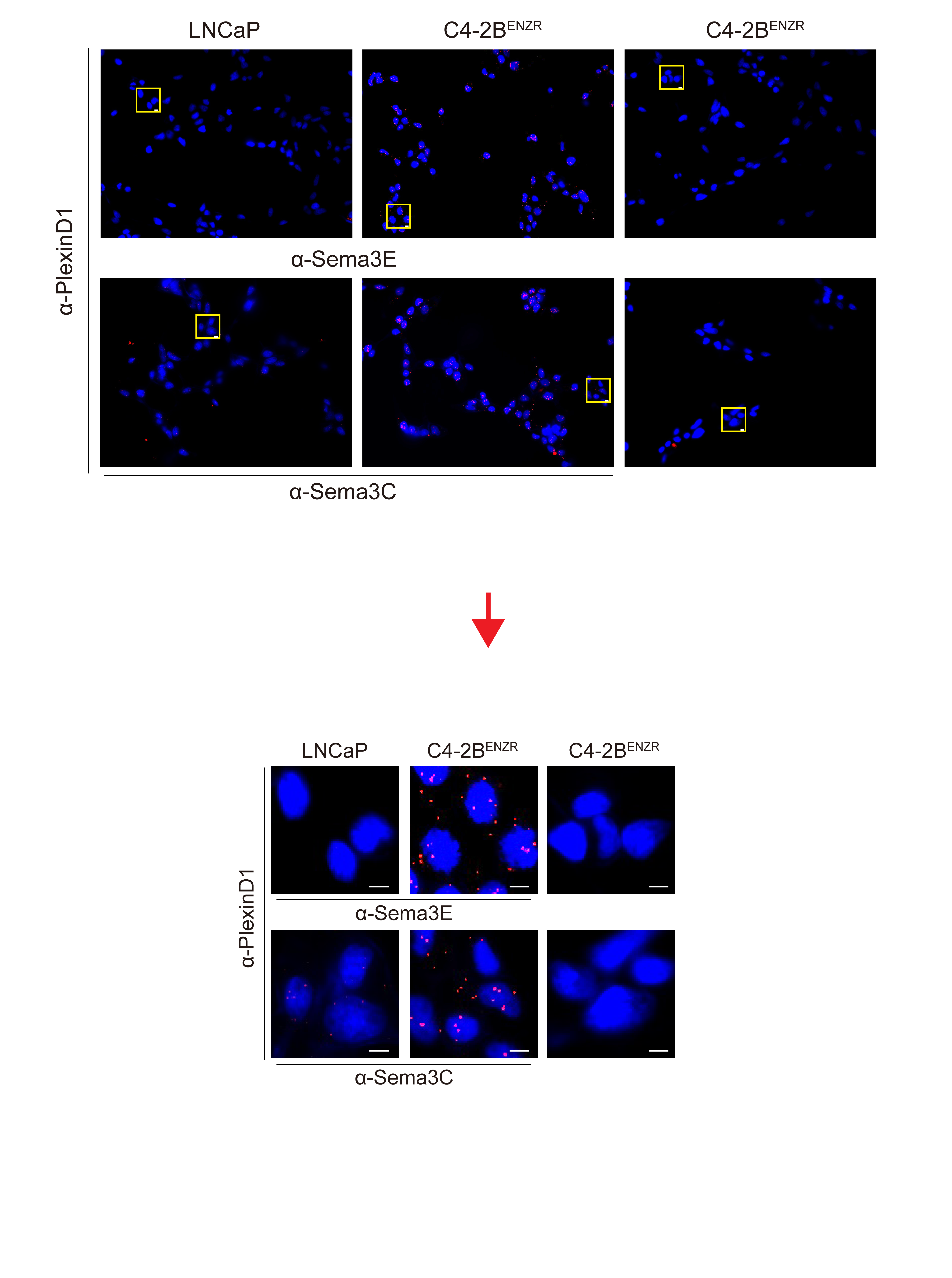

Supplement: Supplementary file 3 — Source data Fig. 1 [file 44321_2024_186_MOESM3_ESM.zip › Figure 1/1F/README.tif]

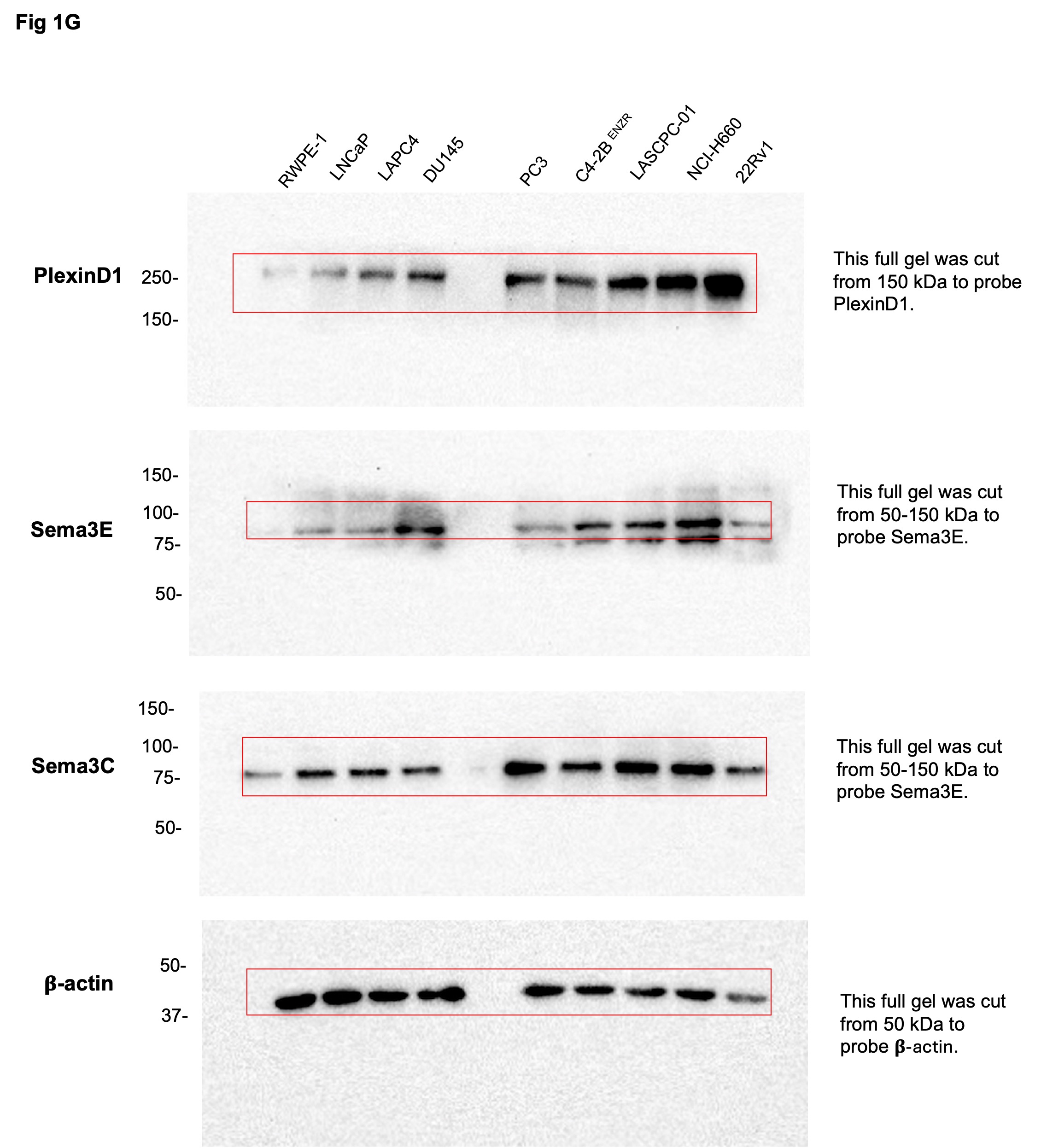

Supplement: Supplementary file 3 — Source data Fig. 1 [file 44321_2024_186_MOESM3_ESM.zip › Figure 1/1G/WB-1G.jpg]

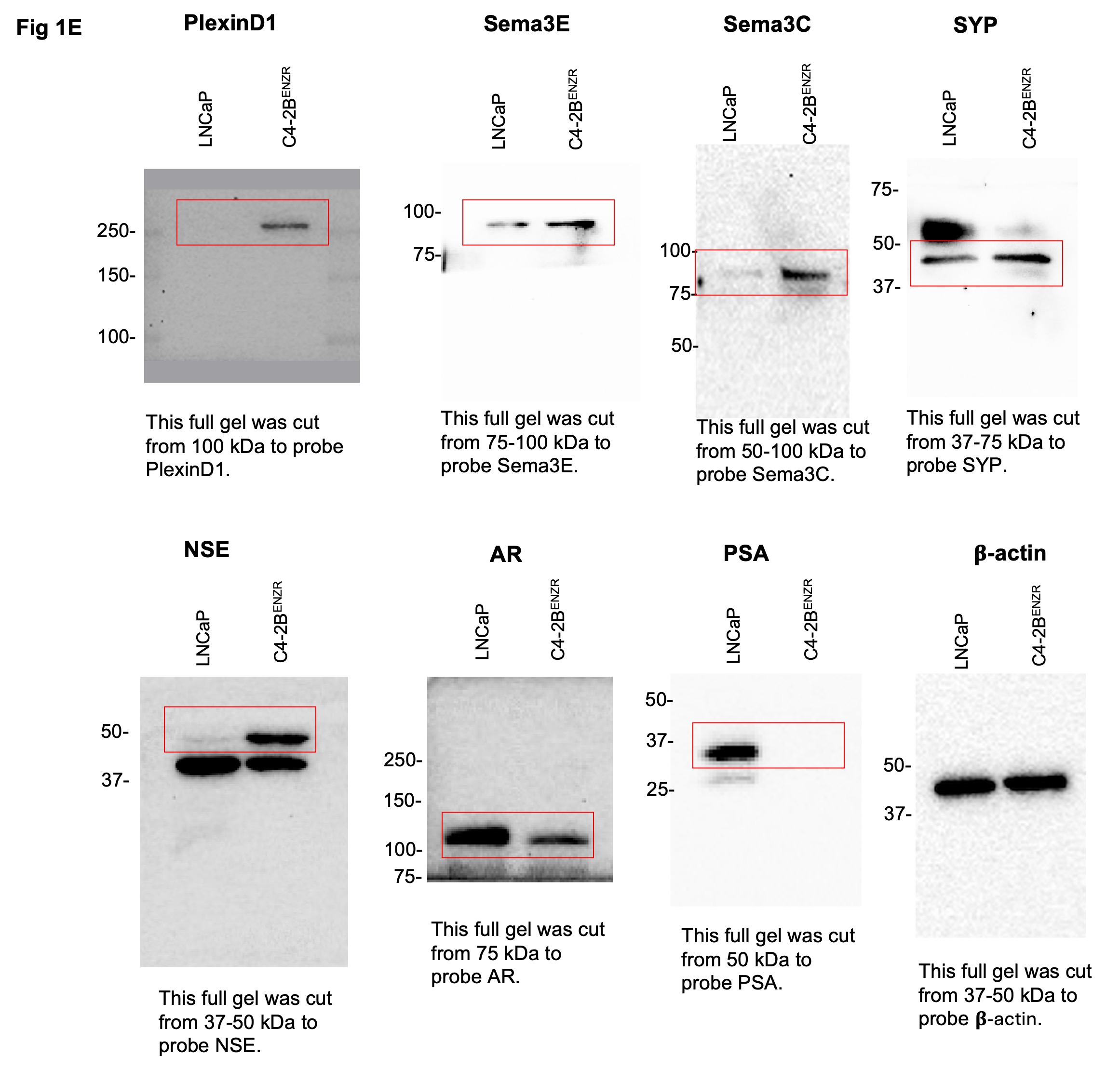

Supplement: Supplementary file 3 — Source data Fig. 1 [file 44321_2024_186_MOESM3_ESM.zip › Figure 1/1E/WB-1E.jpg]

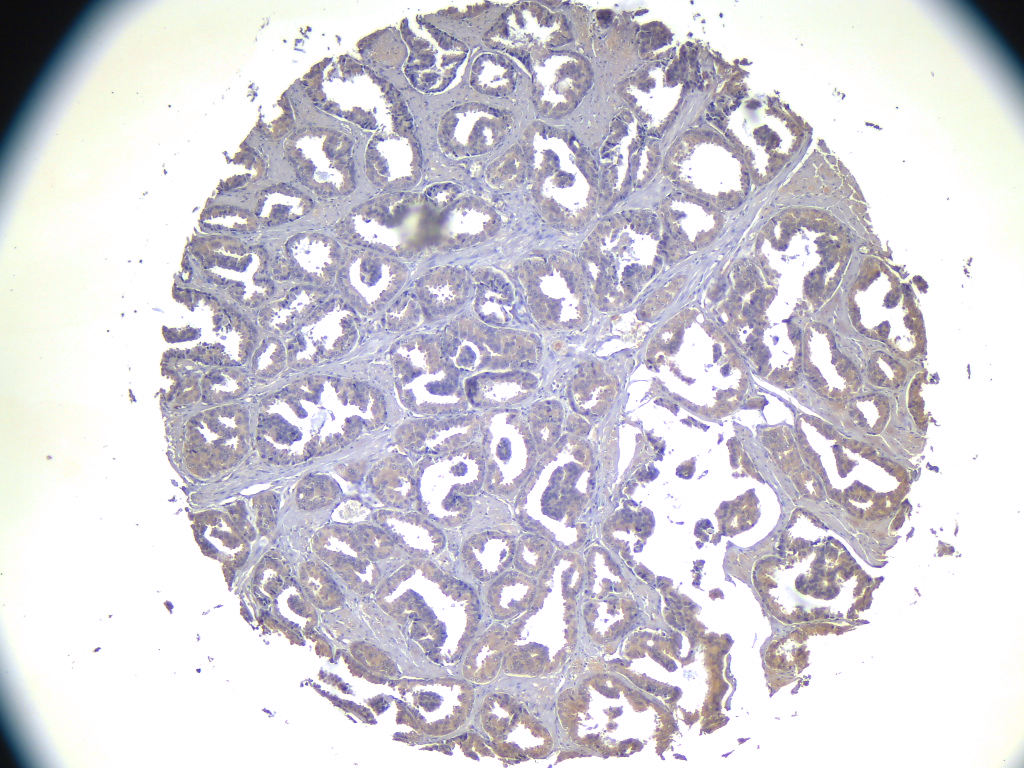

Supplement: Supplementary file 4 — Source data Fig. 2 [file 44321_2024_186_MOESM4_ESM.zip › Figure 2/2A/Cancer_5X.tif]

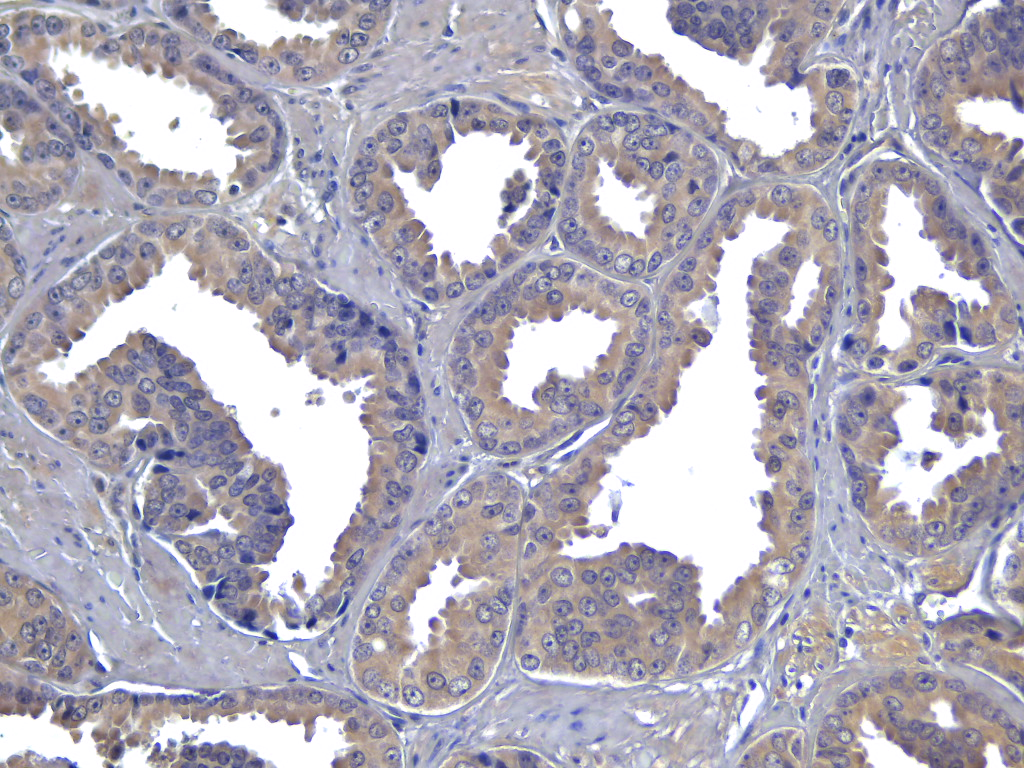

Supplement: Supplementary file 4 — Source data Fig. 2 [file 44321_2024_186_MOESM4_ESM.zip › Figure 2/2A/Cancer_20X.tif]

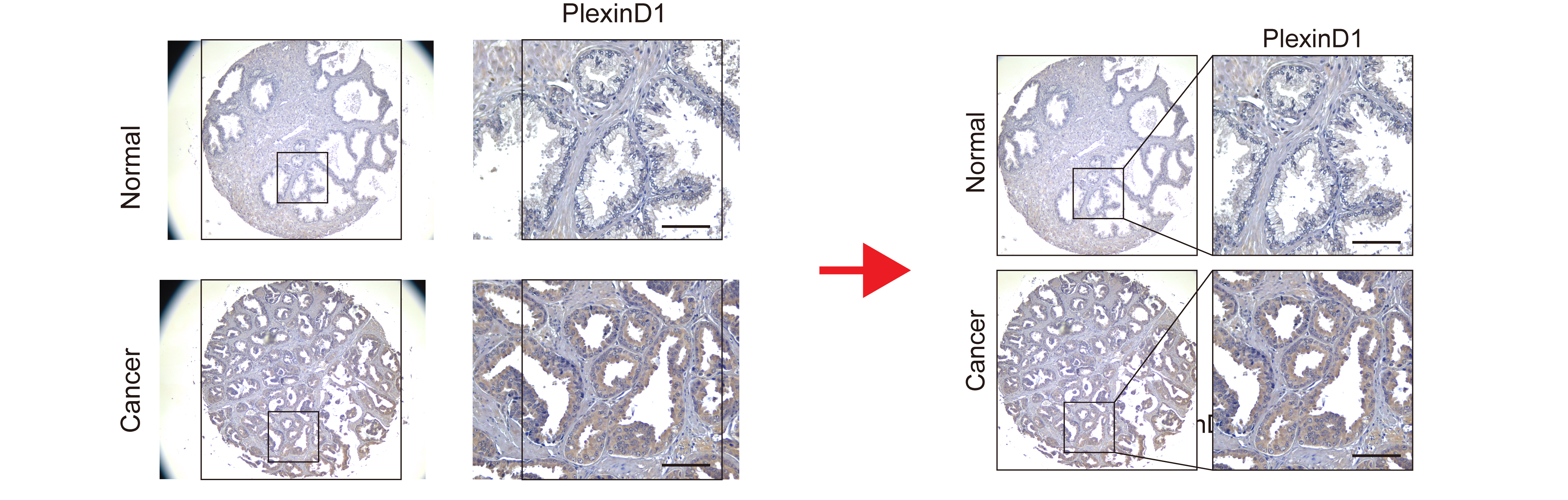

Supplement: Supplementary file 4 — Source data Fig. 2 [file 44321_2024_186_MOESM4_ESM.zip › Figure 2/2A/README.tif]

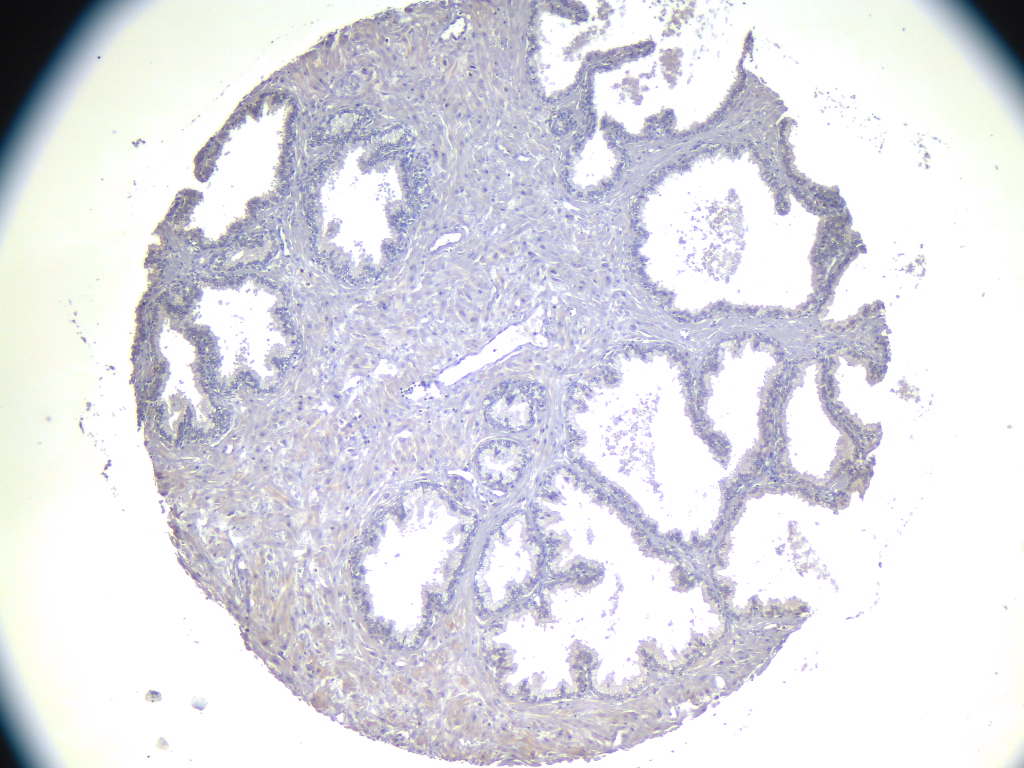

Supplement: Supplementary file 4 — Source data Fig. 2 [file 44321_2024_186_MOESM4_ESM.zip › Figure 2/2A/Normal_5X.tif]

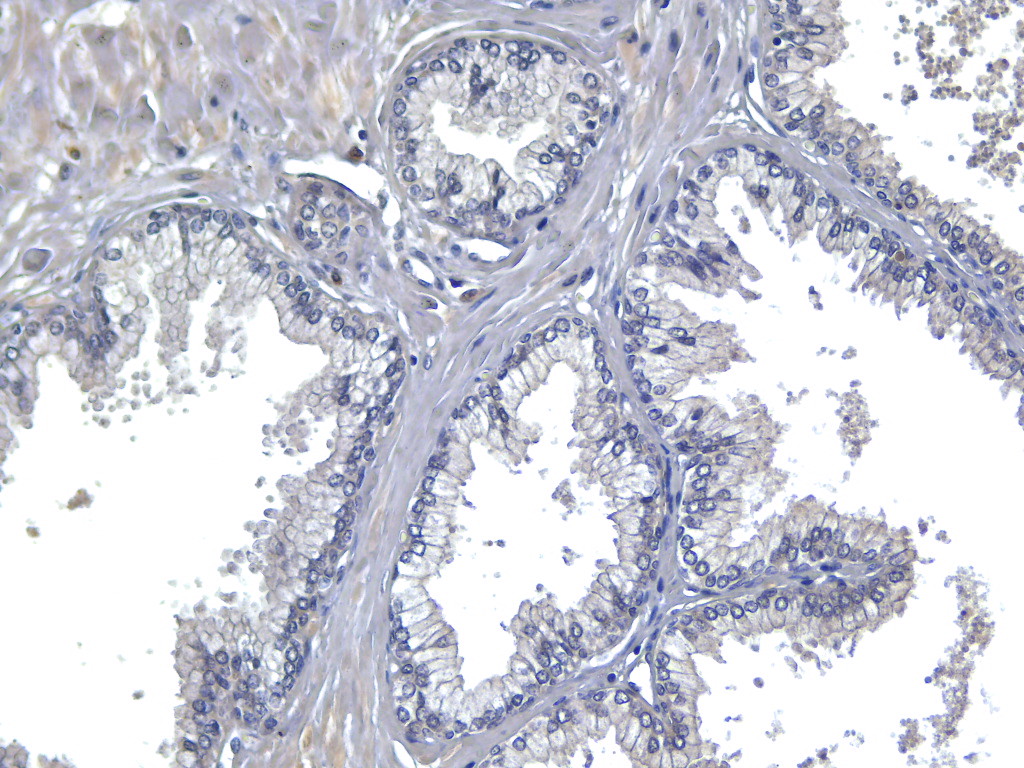

Supplement: Supplementary file 4 — Source data Fig. 2 [file 44321_2024_186_MOESM4_ESM.zip › Figure 2/2A/Normal_20X.tif]

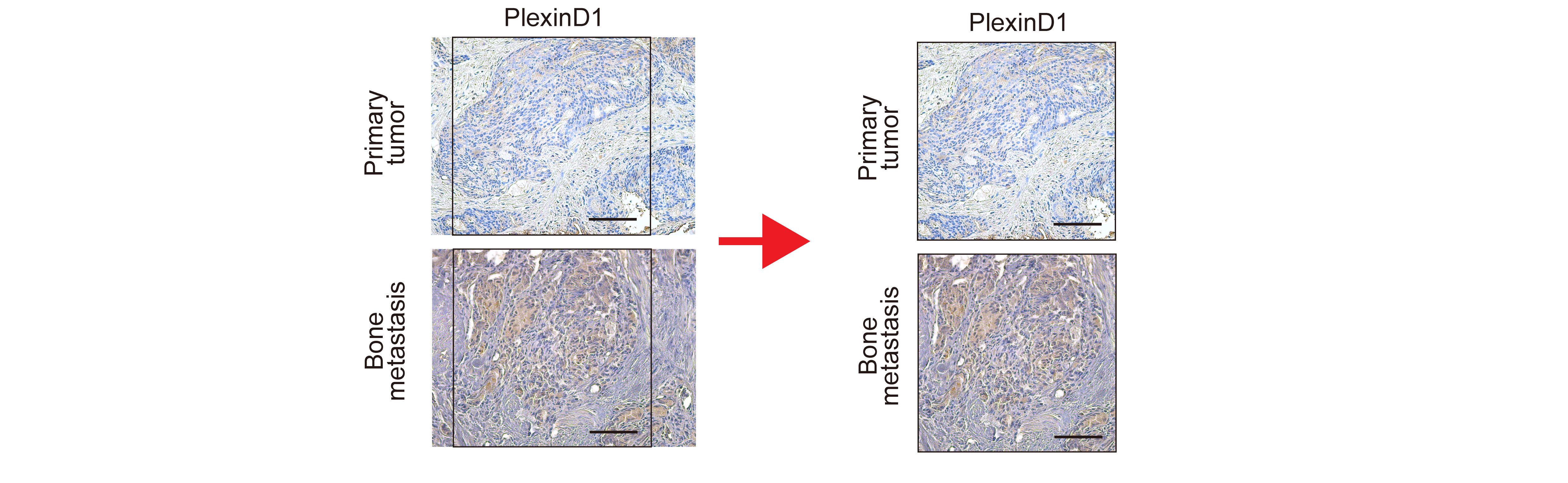

Supplement: Supplementary file 4 — Source data Fig. 2 [file 44321_2024_186_MOESM4_ESM.zip › Figure 2/2F/README.tif]

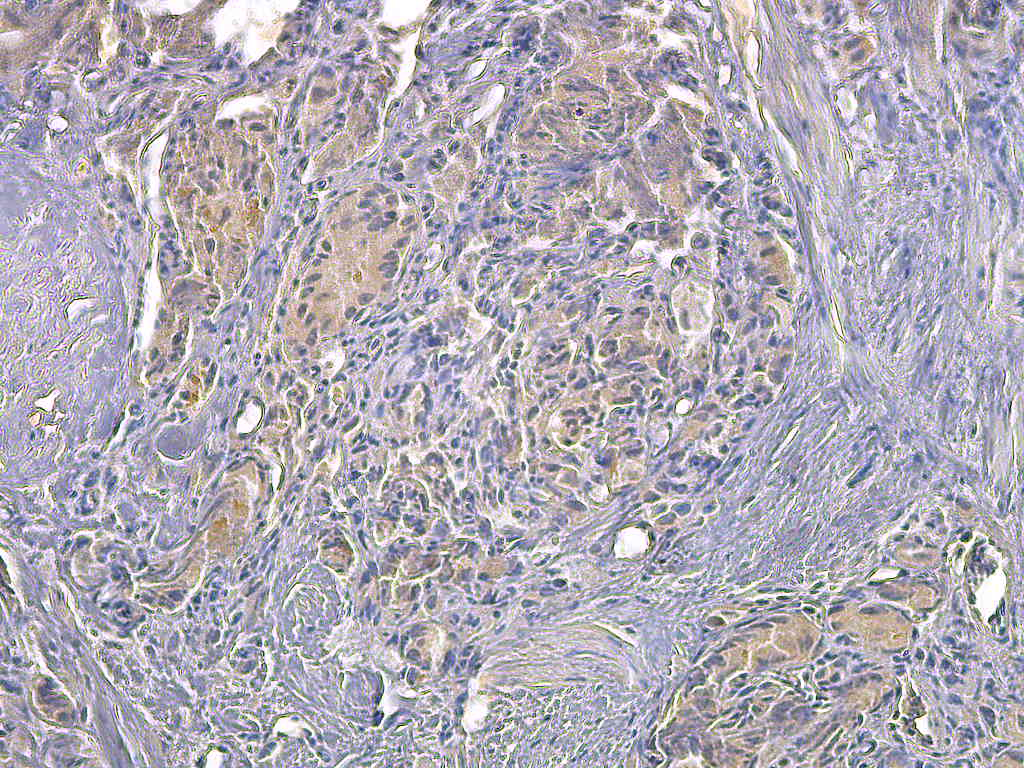

Supplement: Supplementary file 4 — Source data Fig. 2 [file 44321_2024_186_MOESM4_ESM.zip › Figure 2/2F/Bone metastasis.tif]

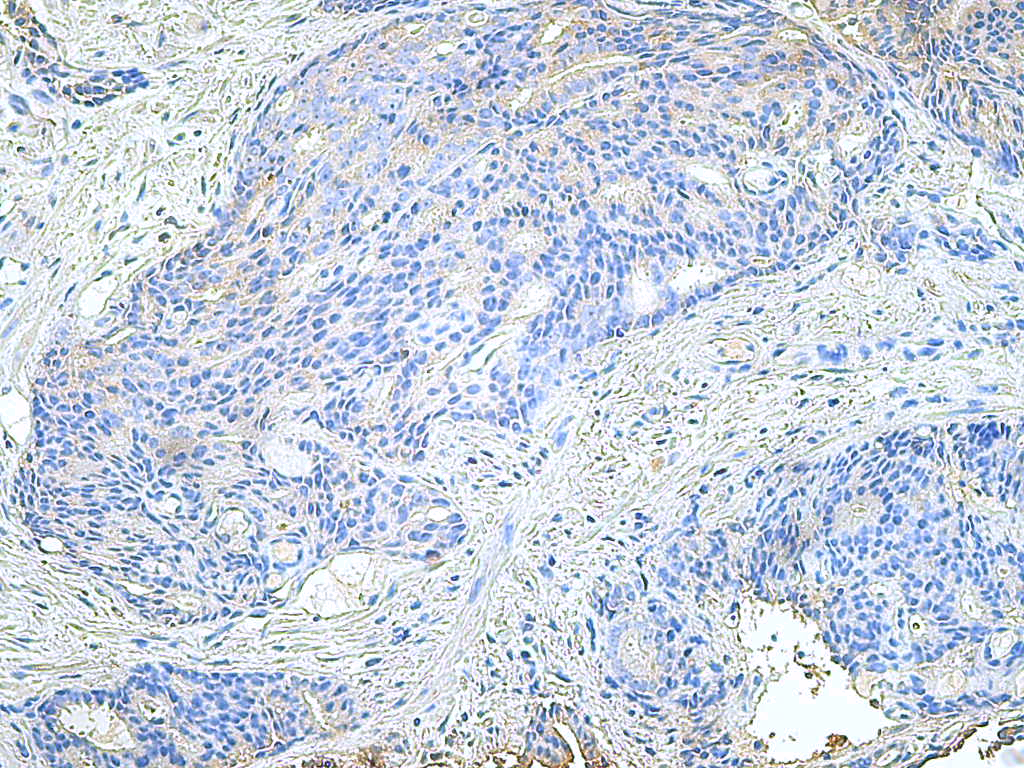

Supplement: Supplementary file 4 — Source data Fig. 2 [file 44321_2024_186_MOESM4_ESM.zip › Figure 2/2F/Primary tumor.tif]

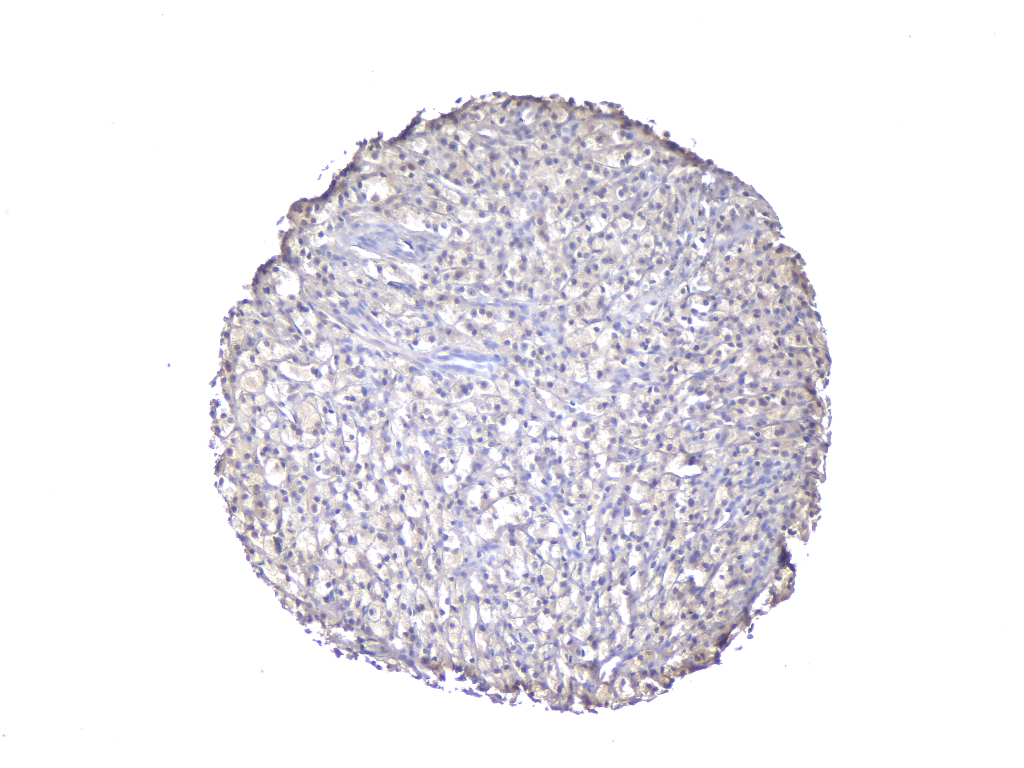

Supplement: Supplementary file 4 — Source data Fig. 2 [file 44321_2024_186_MOESM4_ESM.zip › Figure 2/2D/Pre-hormone therapy_10X.tif]

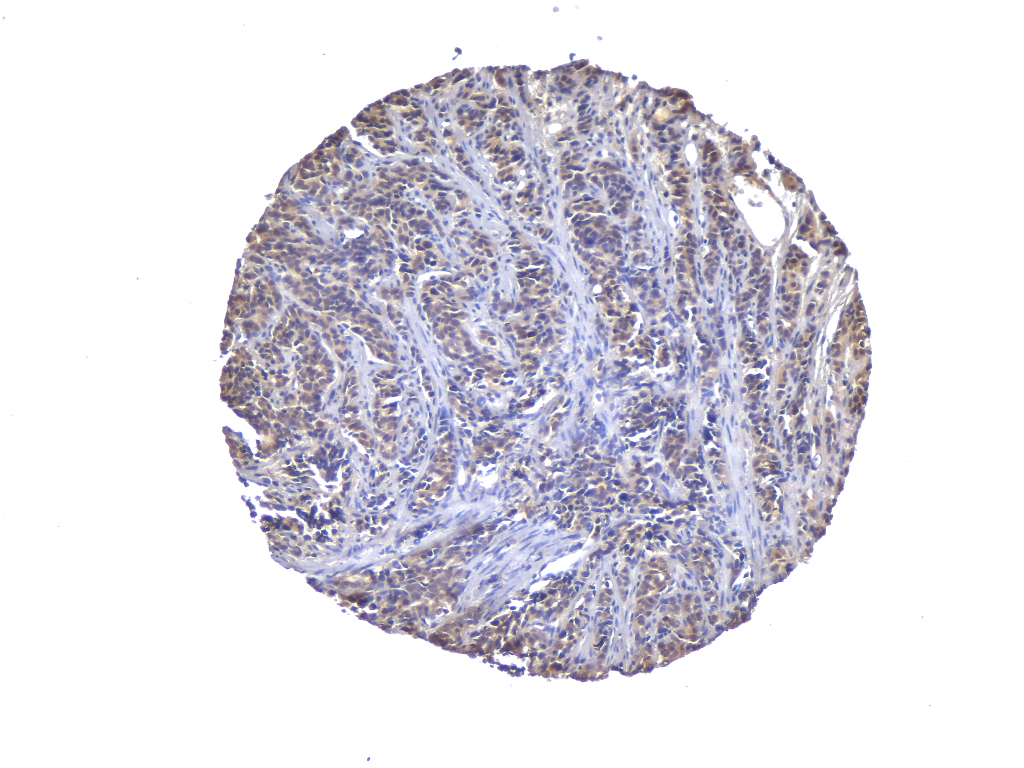

Supplement: Supplementary file 4 — Source data Fig. 2 [file 44321_2024_186_MOESM4_ESM.zip › Figure 2/2D/Post-hormone therapy_10X.tif]

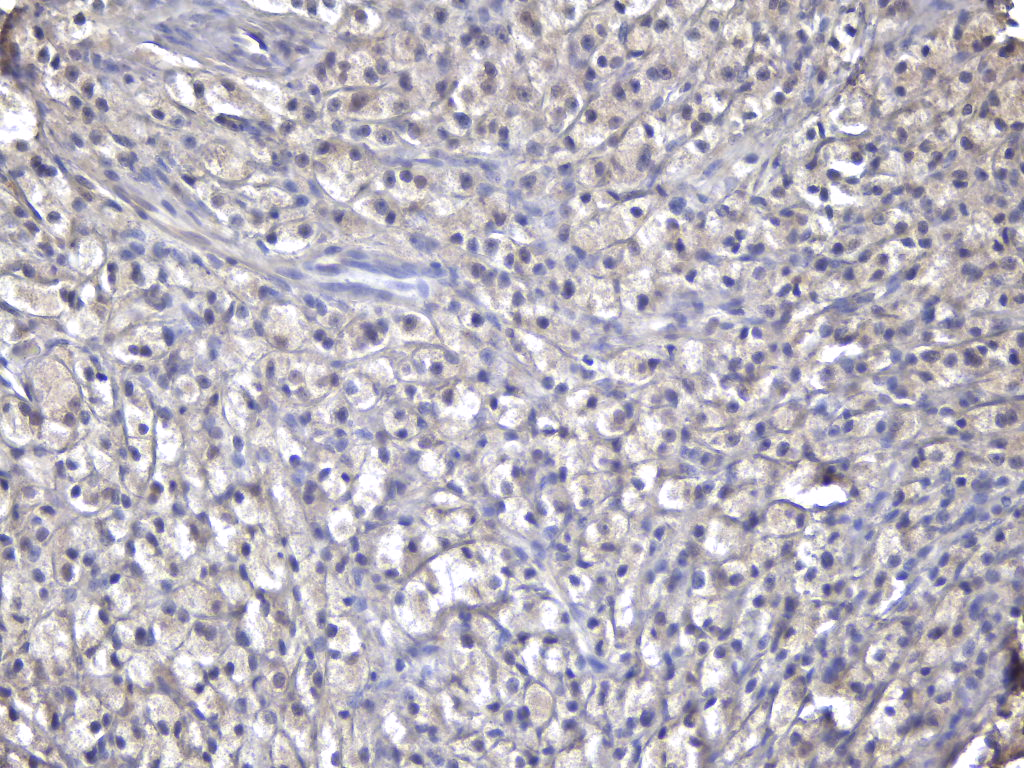

Supplement: Supplementary file 4 — Source data Fig. 2 [file 44321_2024_186_MOESM4_ESM.zip › Figure 2/2D/Pre-hormone therapy_20X.tif]

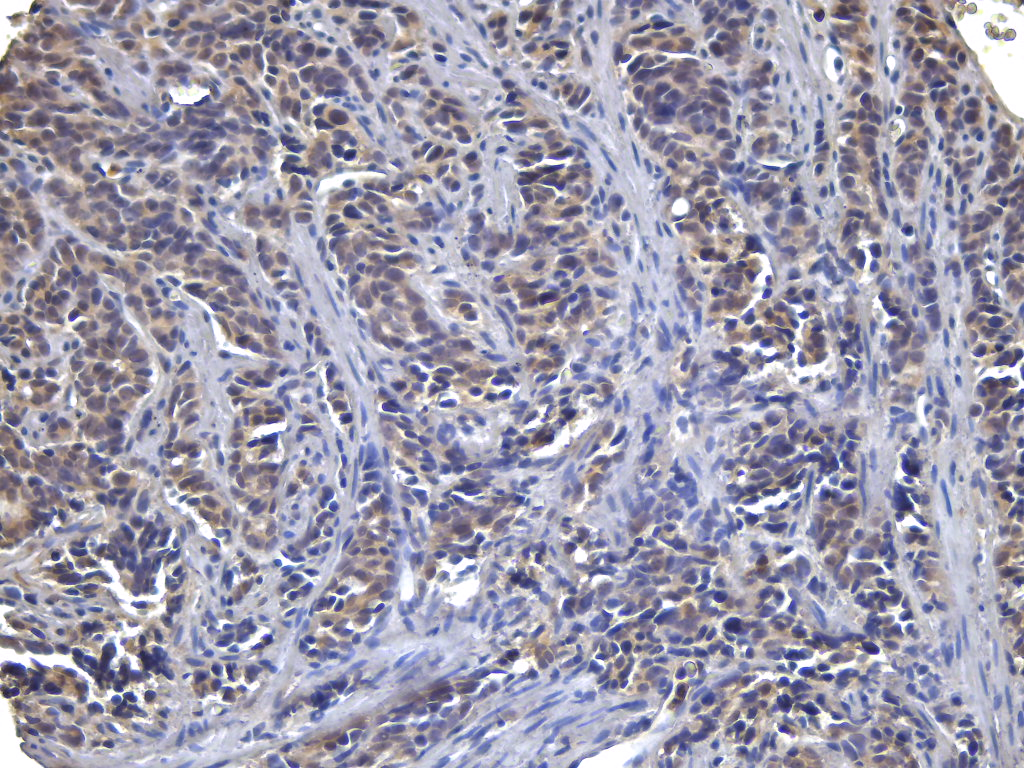

Supplement: Supplementary file 4 — Source data Fig. 2 [file 44321_2024_186_MOESM4_ESM.zip › Figure 2/2D/Post-hormone therapy_20X.tif]

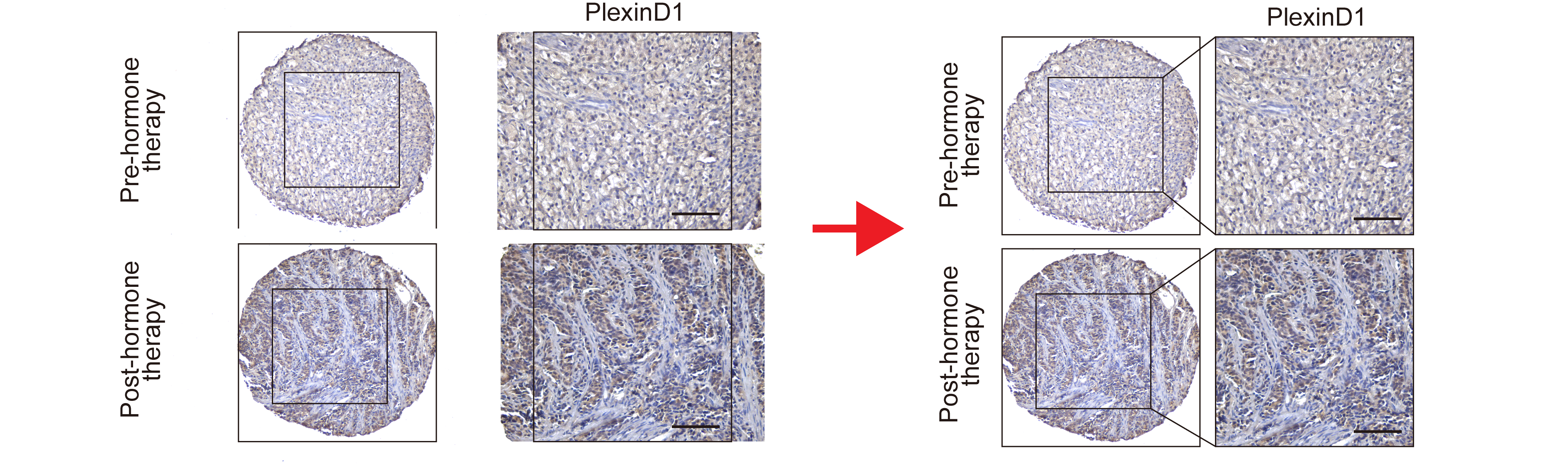

Supplement: Supplementary file 4 — Source data Fig. 2 [file 44321_2024_186_MOESM4_ESM.zip › Figure 2/2D/README.tif]

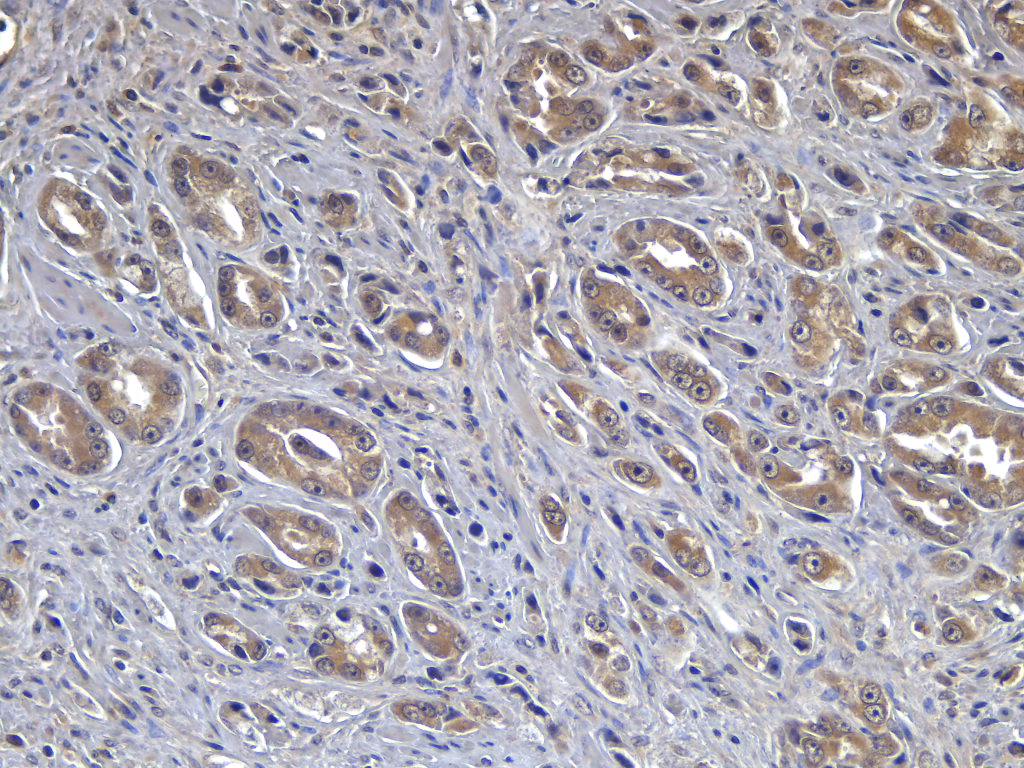

Supplement: Supplementary file 4 — Source data Fig. 2 [file 44321_2024_186_MOESM4_ESM.zip › Figure 2/2B/High GS_20X.tif]

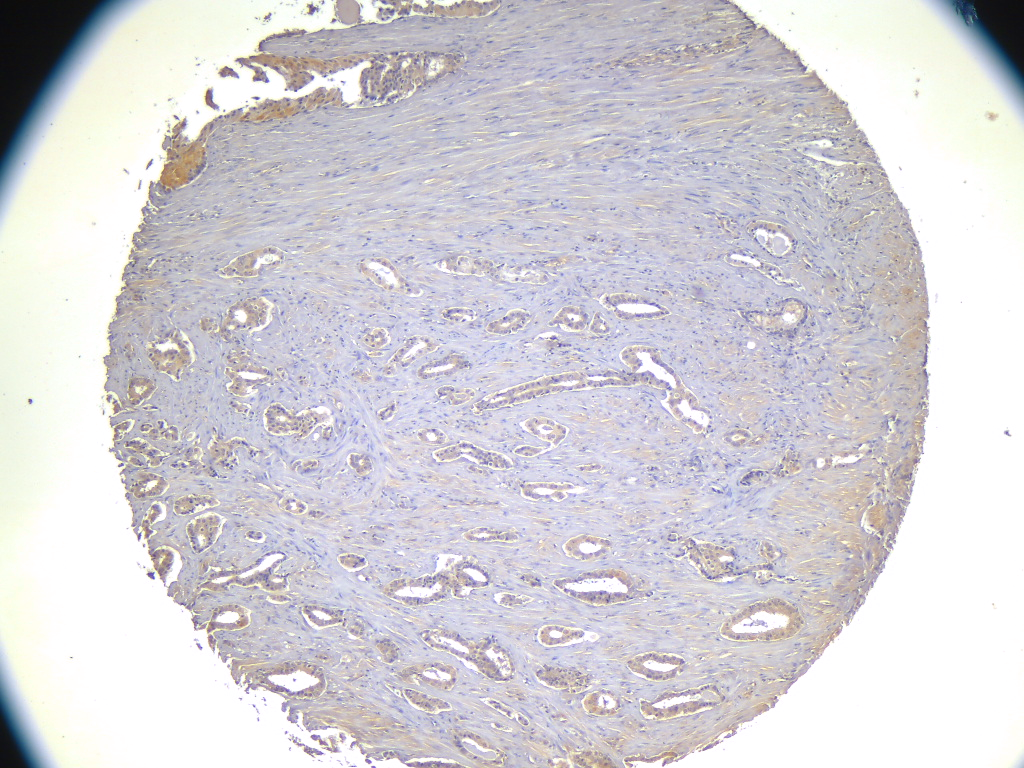

Supplement: Supplementary file 4 — Source data Fig. 2 [file 44321_2024_186_MOESM4_ESM.zip › Figure 2/2B/Low GS_5X.tif]

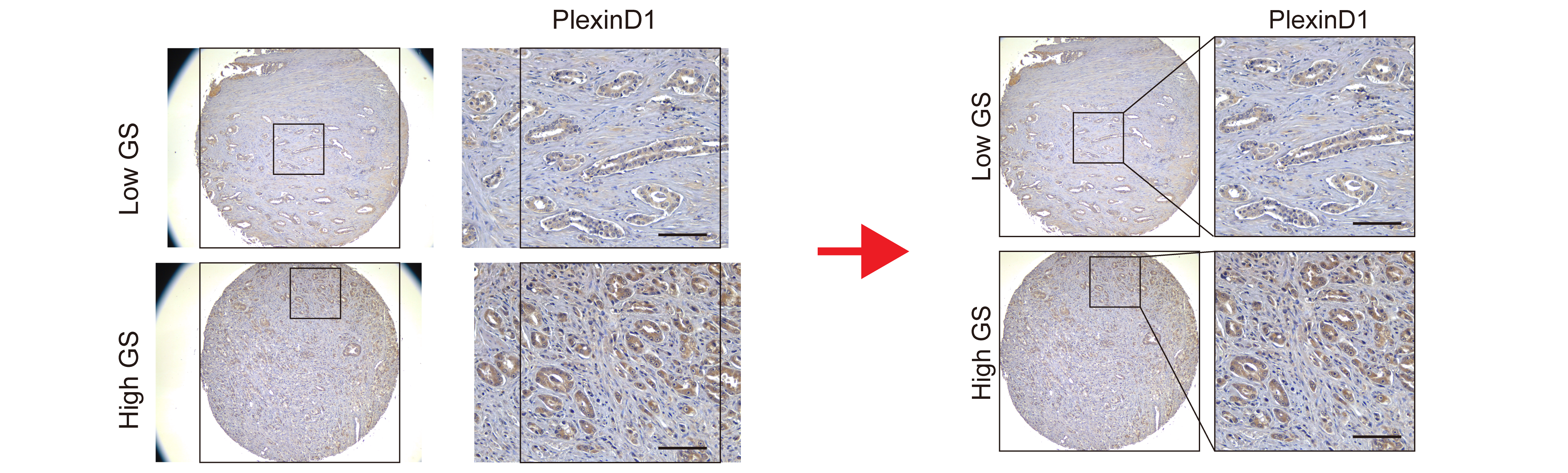

Supplement: Supplementary file 4 — Source data Fig. 2 [file 44321_2024_186_MOESM4_ESM.zip › Figure 2/2B/README.tif]

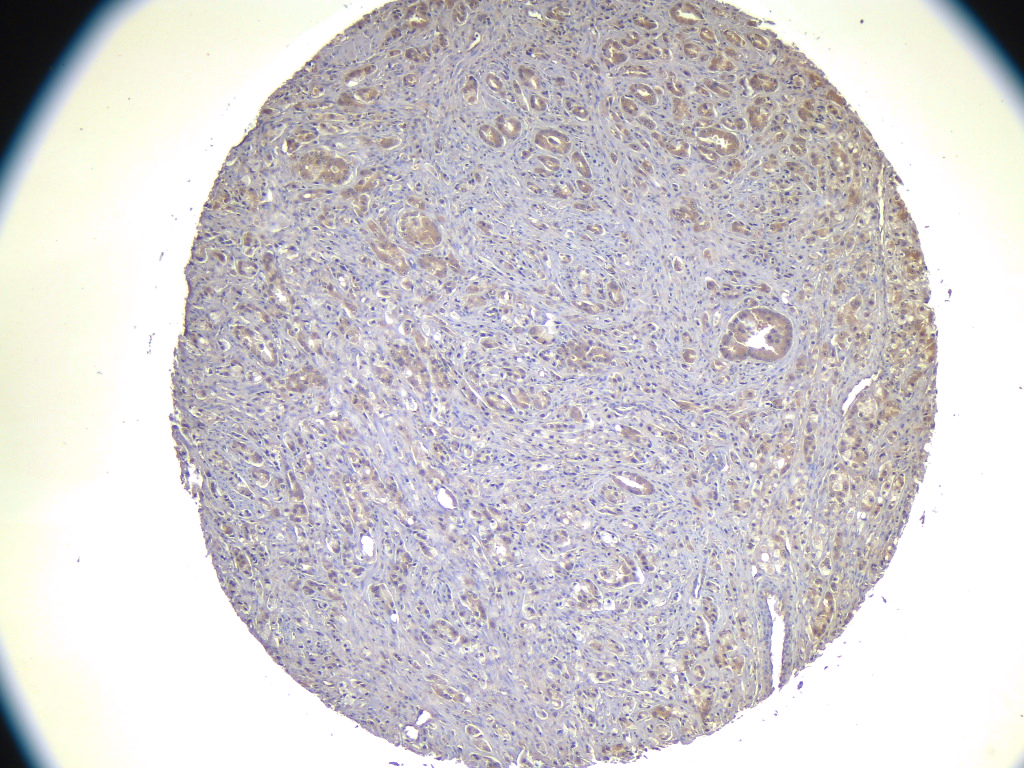

Supplement: Supplementary file 4 — Source data Fig. 2 [file 44321_2024_186_MOESM4_ESM.zip › Figure 2/2B/High GS_5X.tif]

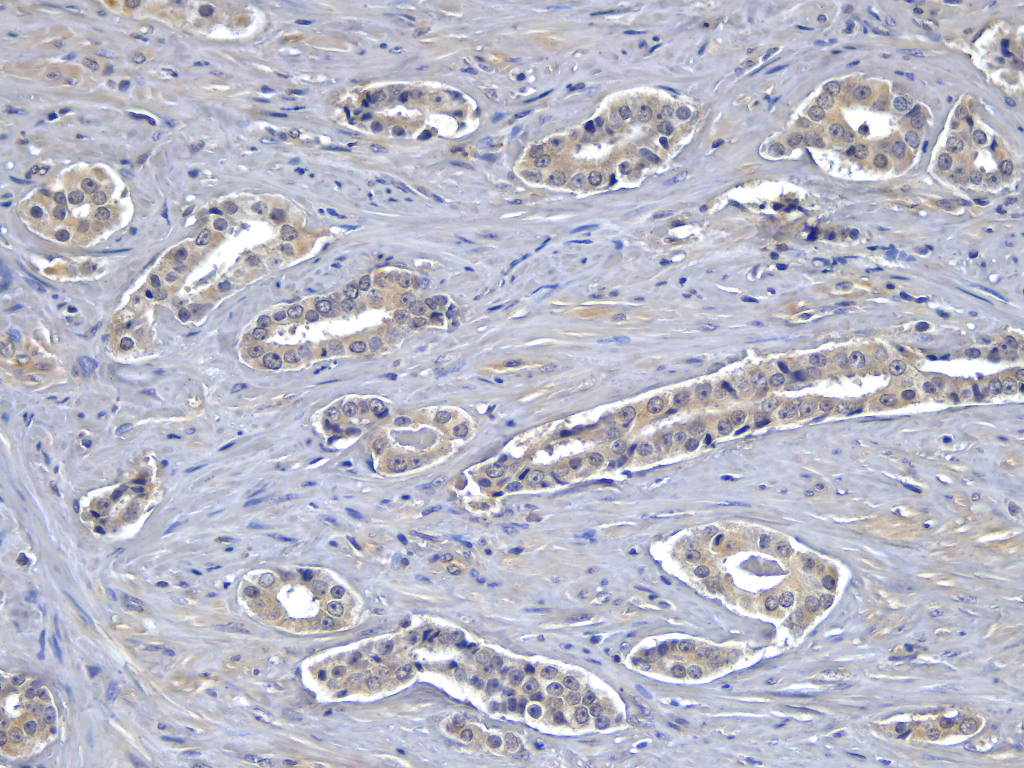

Supplement: Supplementary file 4 — Source data Fig. 2 [file 44321_2024_186_MOESM4_ESM.zip › Figure 2/2B/Low GS_20X.tif]

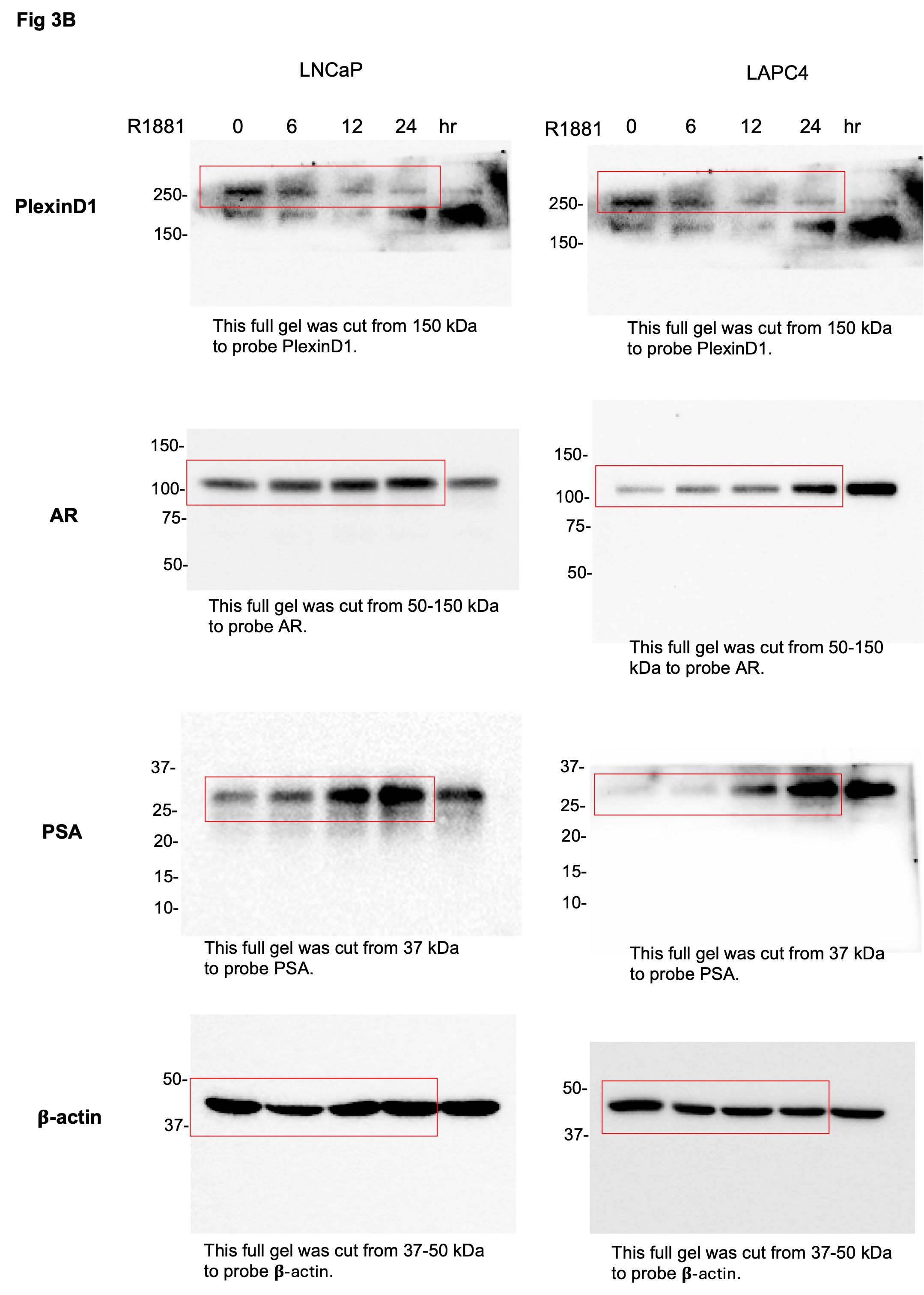

Supplement: Supplementary file 5 — Source data Fig. 3 [file 44321_2024_186_MOESM5_ESM.zip › Figure 3/3B/WB-3B.jpg]

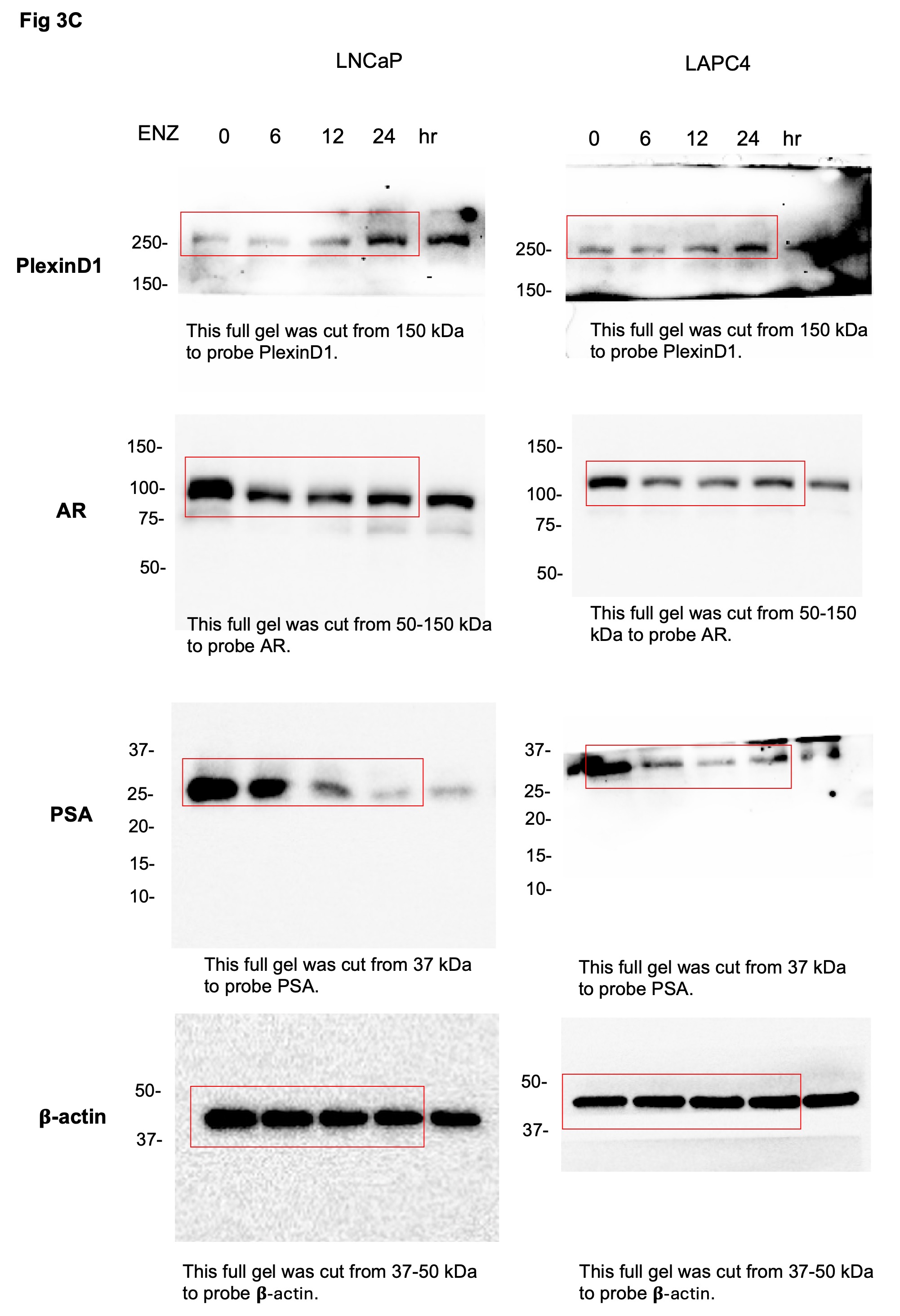

Supplement: Supplementary file 5 — Source data Fig. 3 [file 44321_2024_186_MOESM5_ESM.zip › Figure 3/3C/WB-3C.jpg]

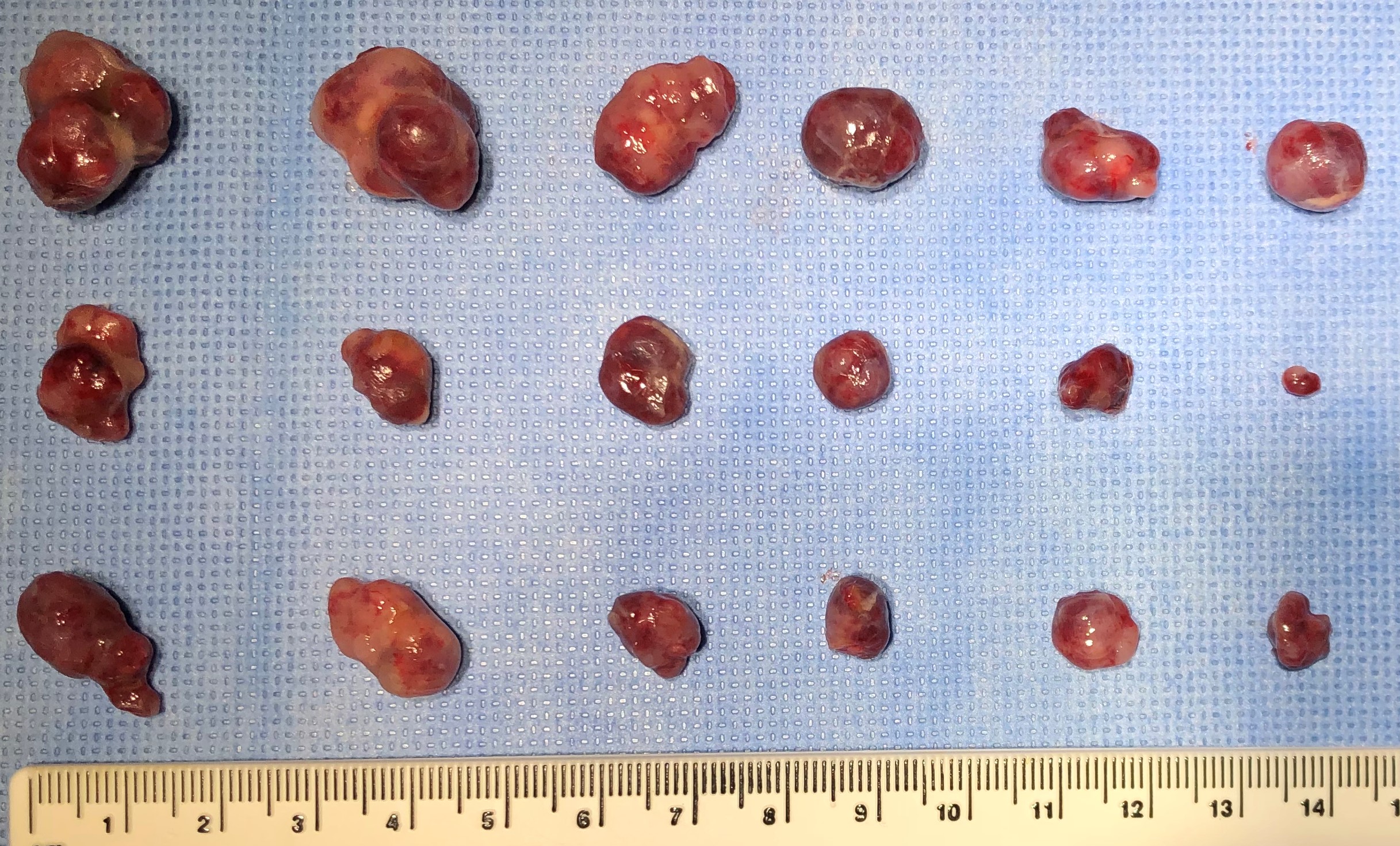

Supplement: Supplementary file 6 — Source data Fig. 4 [file 44321_2024_186_MOESM6_ESM.zip › Figure 4/4K/22Rv1 tumor.jpg]

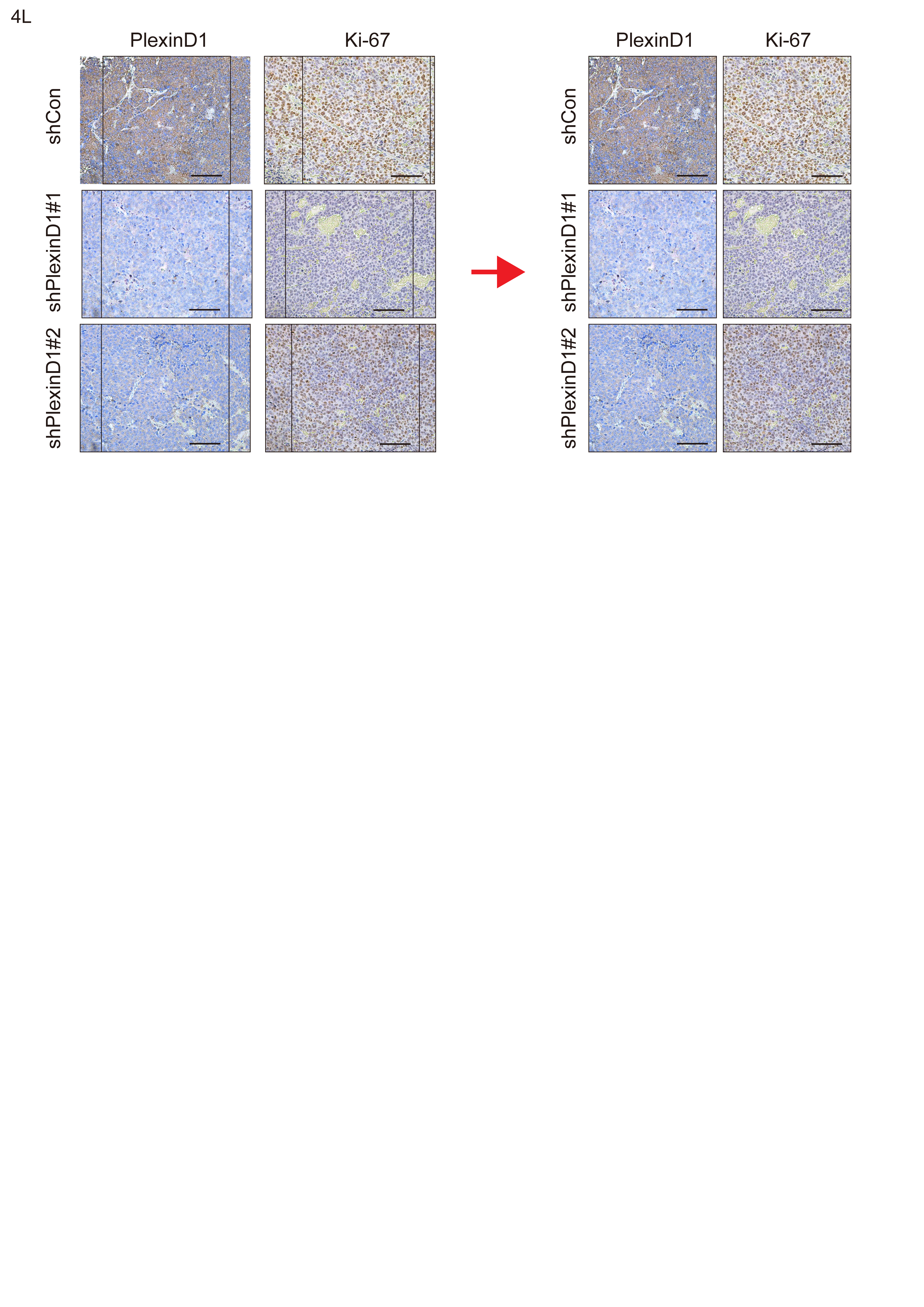

Supplement: Supplementary file 6 — Source data Fig. 4 [file 44321_2024_186_MOESM6_ESM.zip › Figure 4/4L/README.tif]

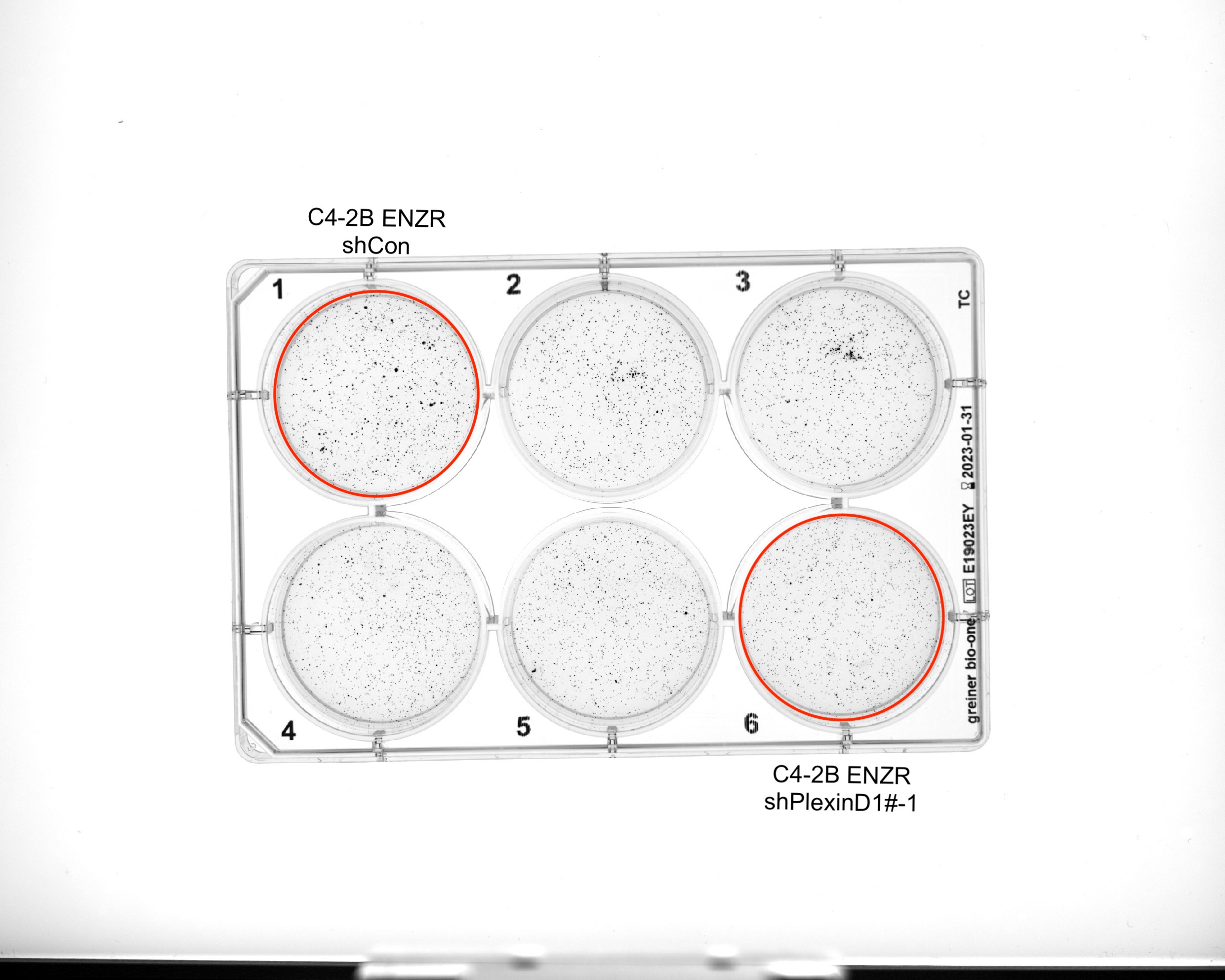

Supplement: Supplementary file 6 — Source data Fig. 4 [file 44321_2024_186_MOESM6_ESM.zip › Figure 4/4C/colony_C42B ENZR_ shC and shP#1 .jpg]

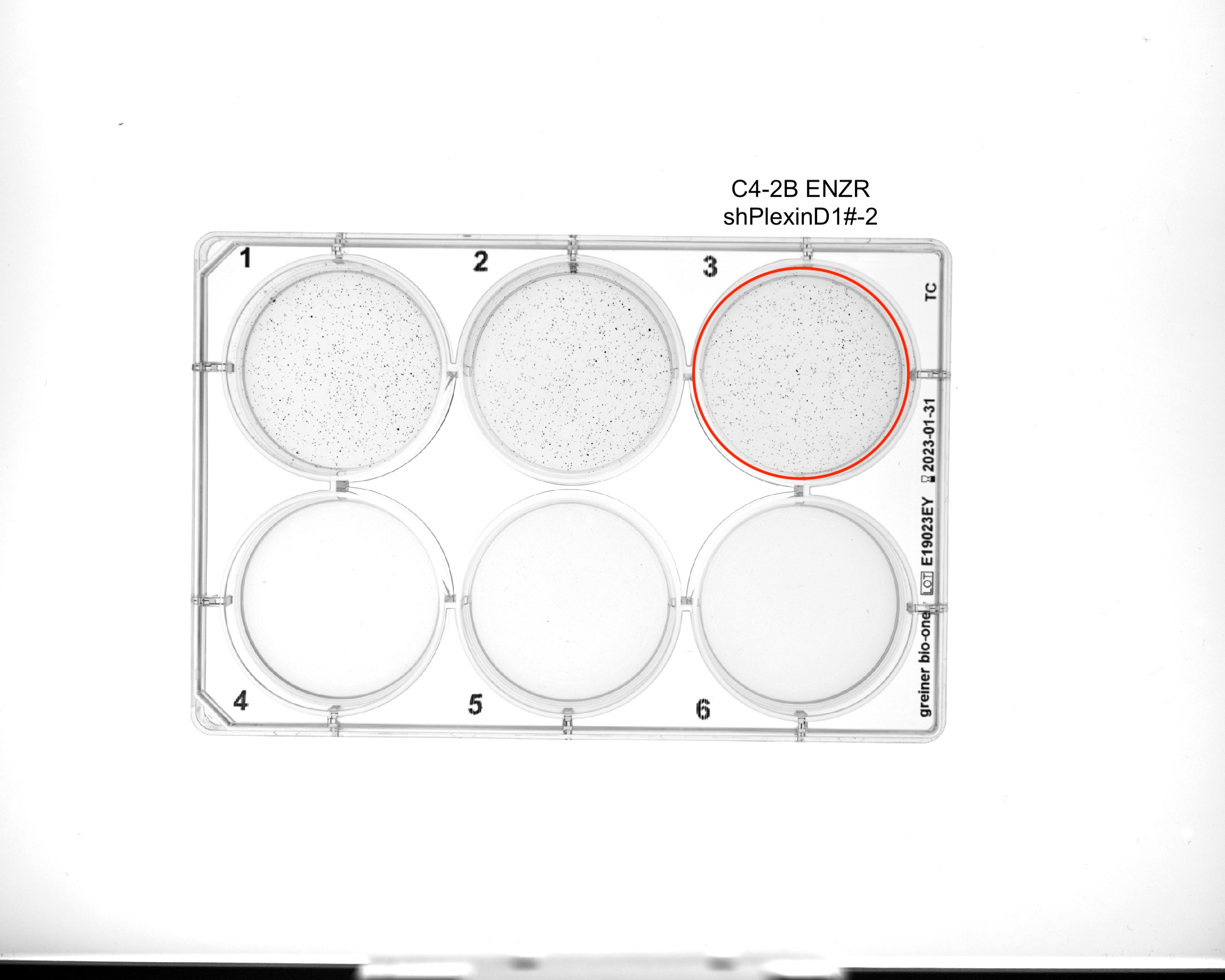

Supplement: Supplementary file 6 — Source data Fig. 4 [file 44321_2024_186_MOESM6_ESM.zip › Figure 4/4C/colony_C42B ENZR_ shP#2.jpg]

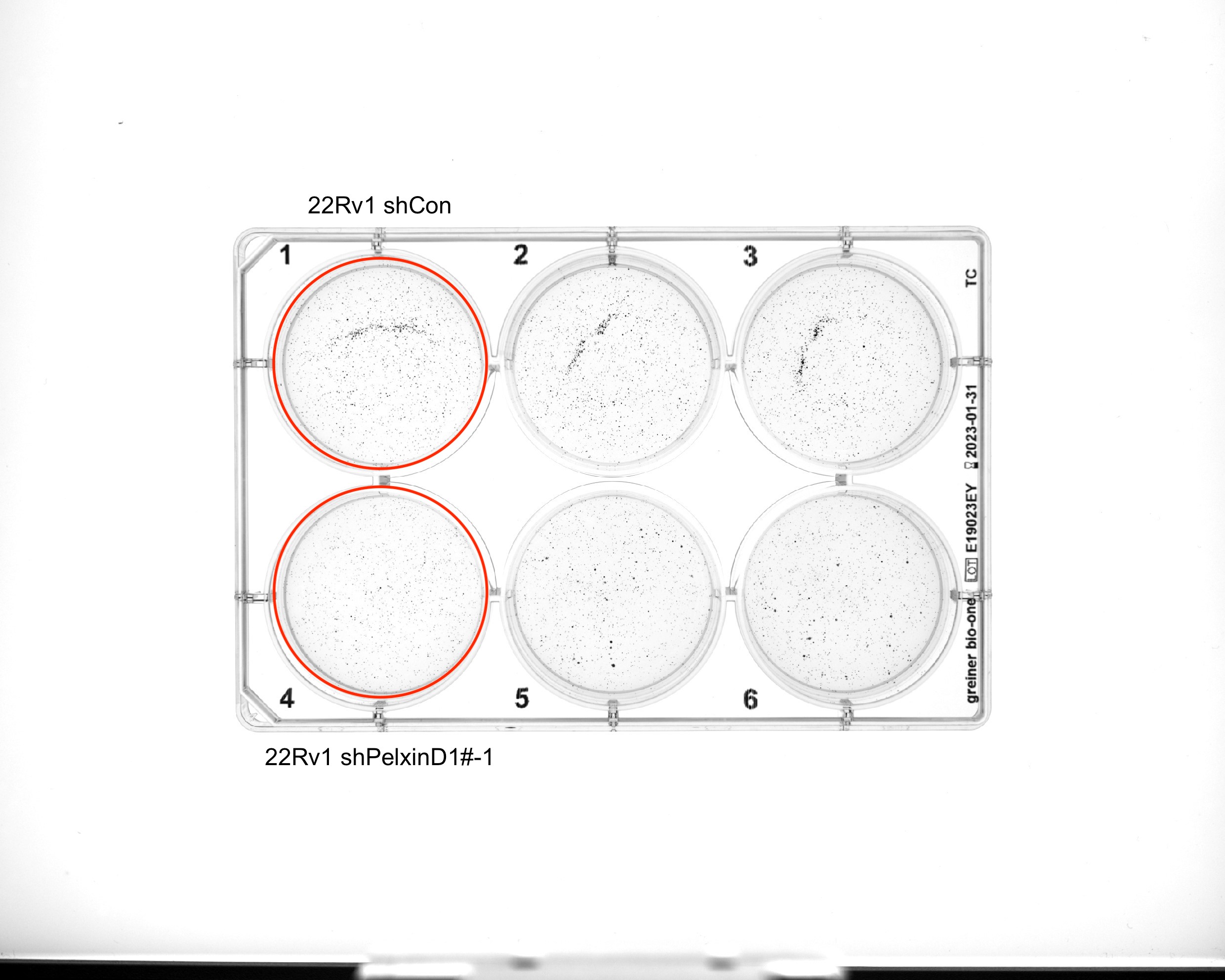

Supplement: Supplementary file 6 — Source data Fig. 4 [file 44321_2024_186_MOESM6_ESM.zip › Figure 4/4C/colony_22Rv1_ shC and shP#1.jpg]

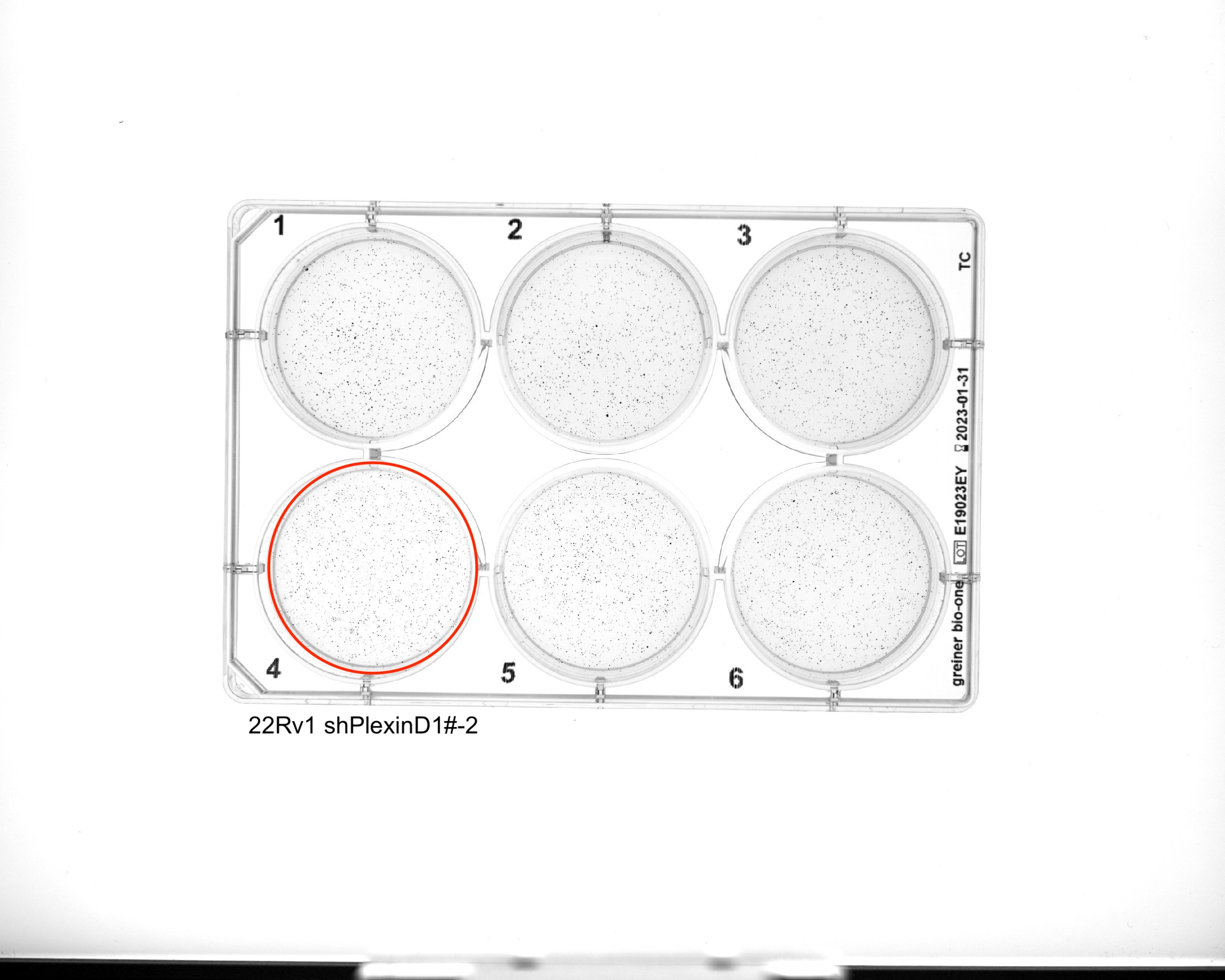

Supplement: Supplementary file 6 — Source data Fig. 4 [file 44321_2024_186_MOESM6_ESM.zip › Figure 4/4C/colony_22Rv1 shP#2.jpg]

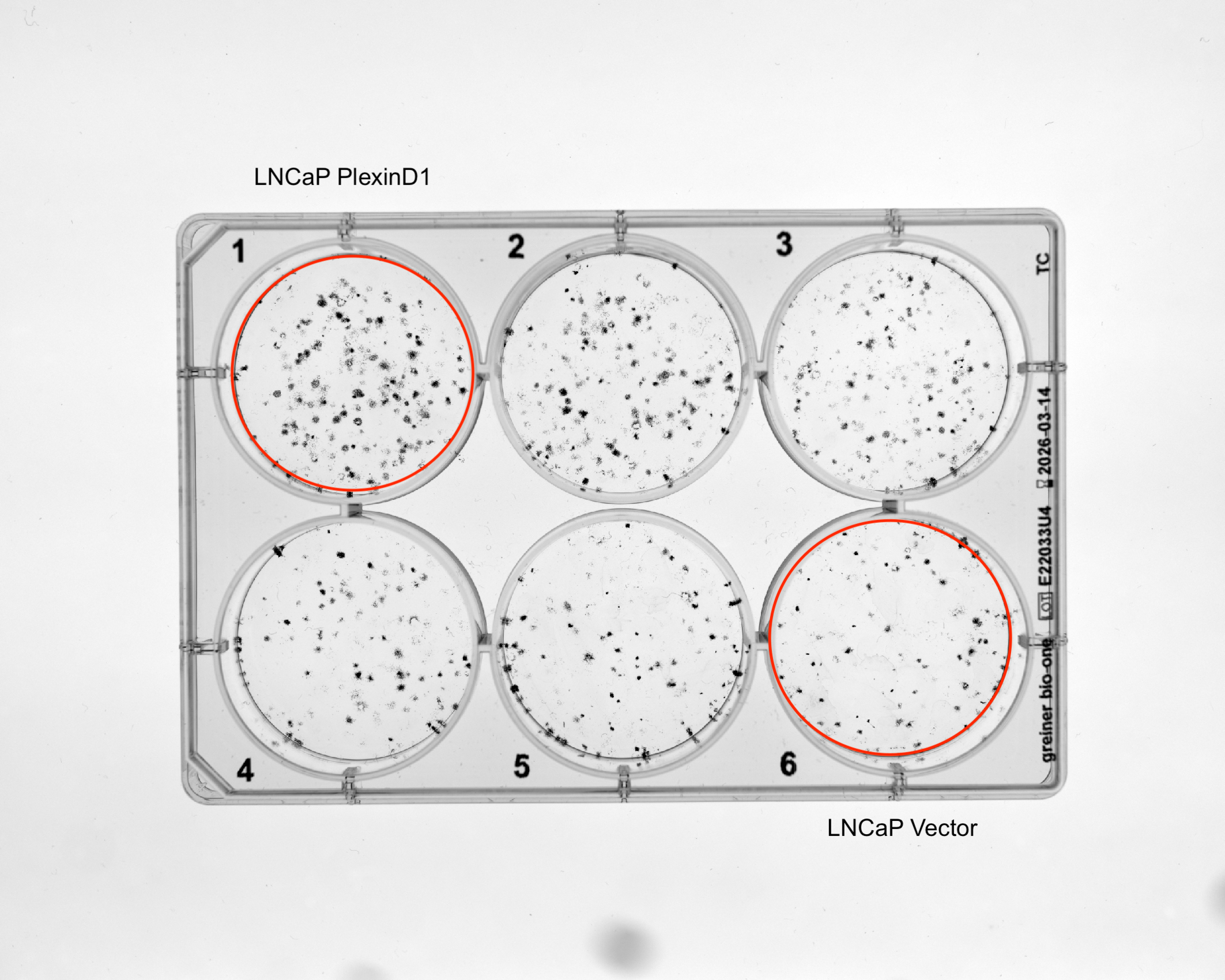

Supplement: Supplementary file 6 — Source data Fig. 4 [file 44321_2024_186_MOESM6_ESM.zip › Figure 4/4C/LNCaP.jpg]

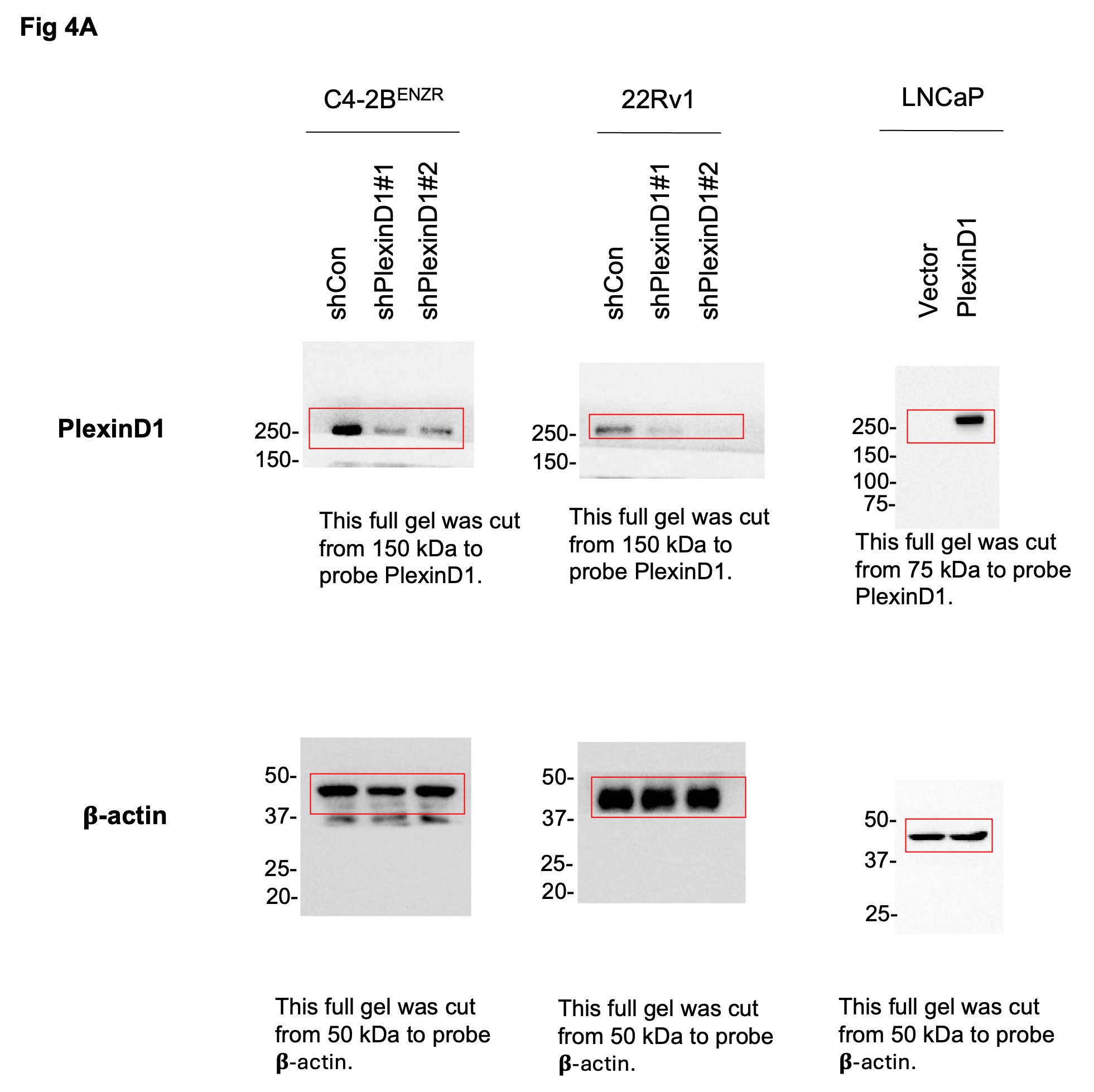

Supplement: Supplementary file 6 — Source data Fig. 4 [file 44321_2024_186_MOESM6_ESM.zip › Figure 4/4A/WB-4A.jpg]

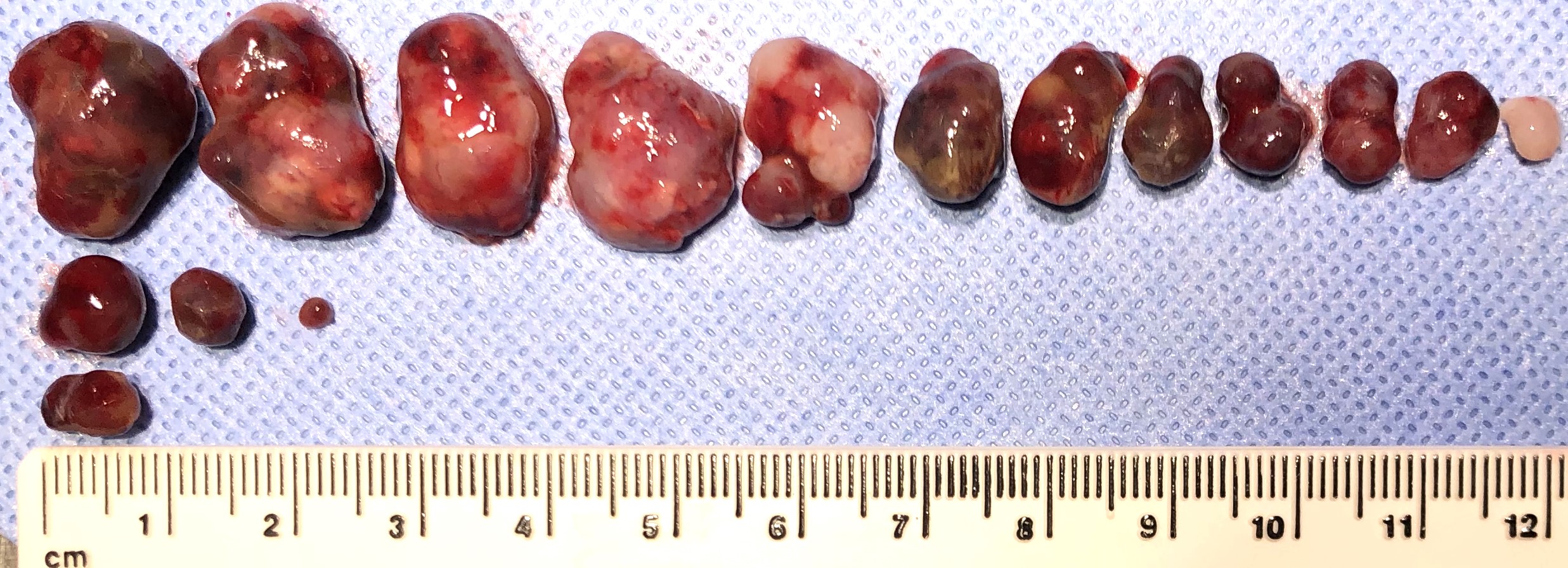

Supplement: Supplementary file 6 — Source data Fig. 4 [file 44321_2024_186_MOESM6_ESM.zip › Figure 4/4H/C4-2B ENZR tumor.jpg]

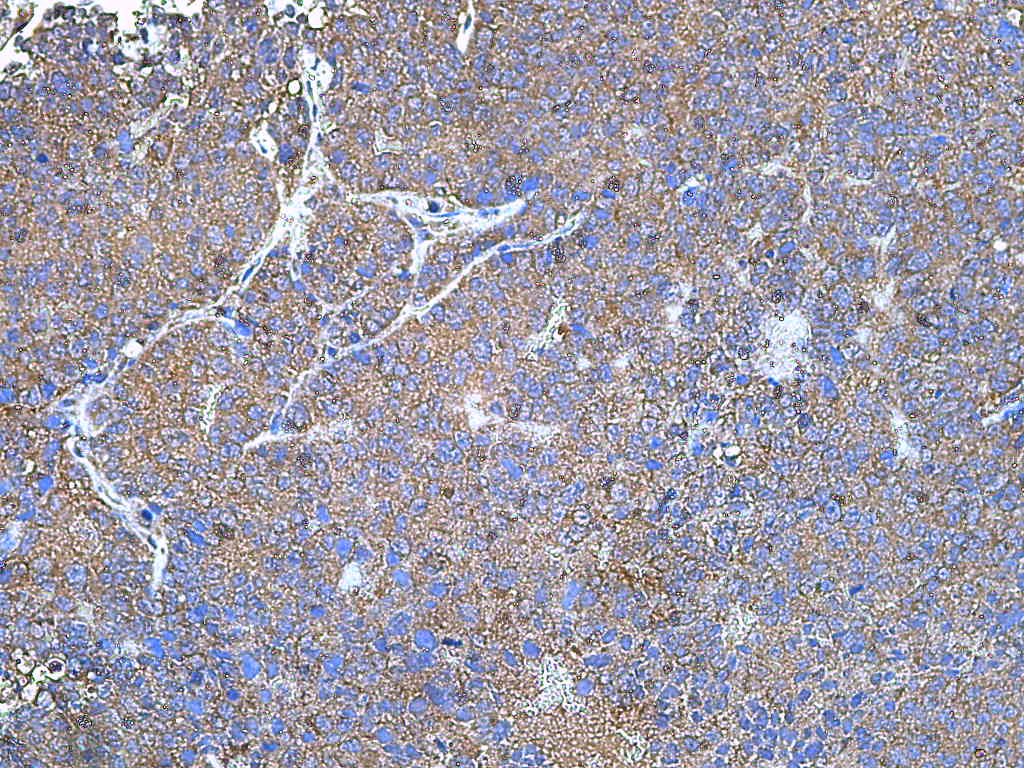

Supplement: Supplementary file 6 — Source data Fig. 4 [file 44321_2024_186_MOESM6_ESM.zip › Figure 4/4L/PlexinD1/shCon.tif]

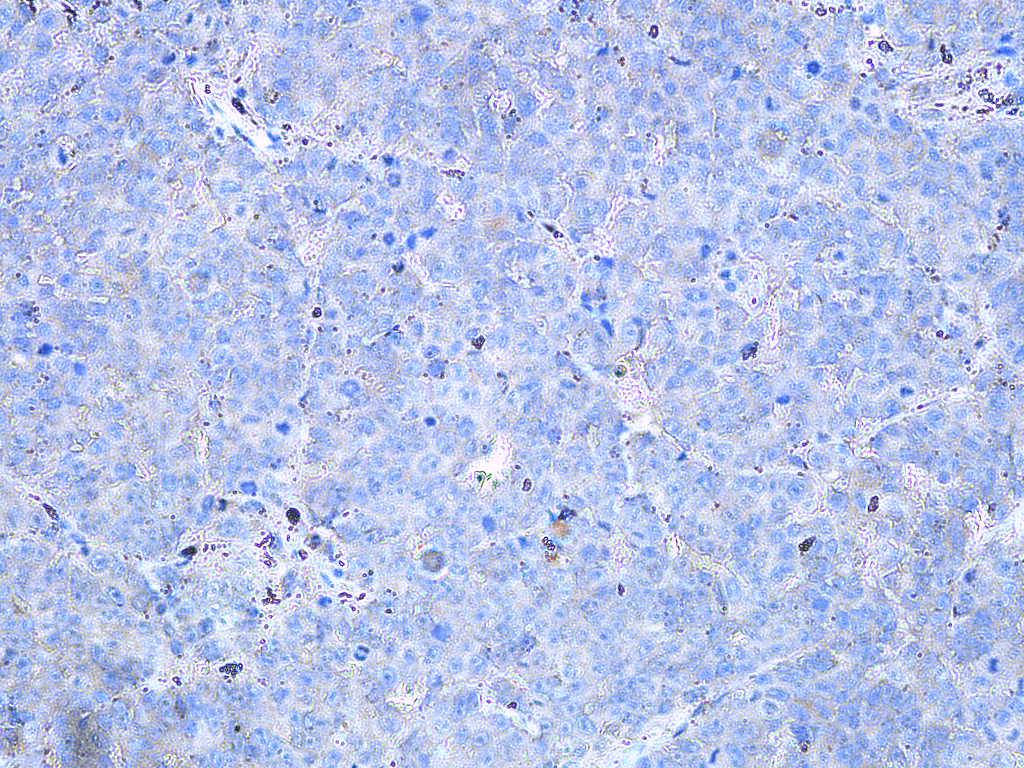

Supplement: Supplementary file 6 — Source data Fig. 4 [file 44321_2024_186_MOESM6_ESM.zip › Figure 4/4L/PlexinD1/shPlexinD1-1.tif]

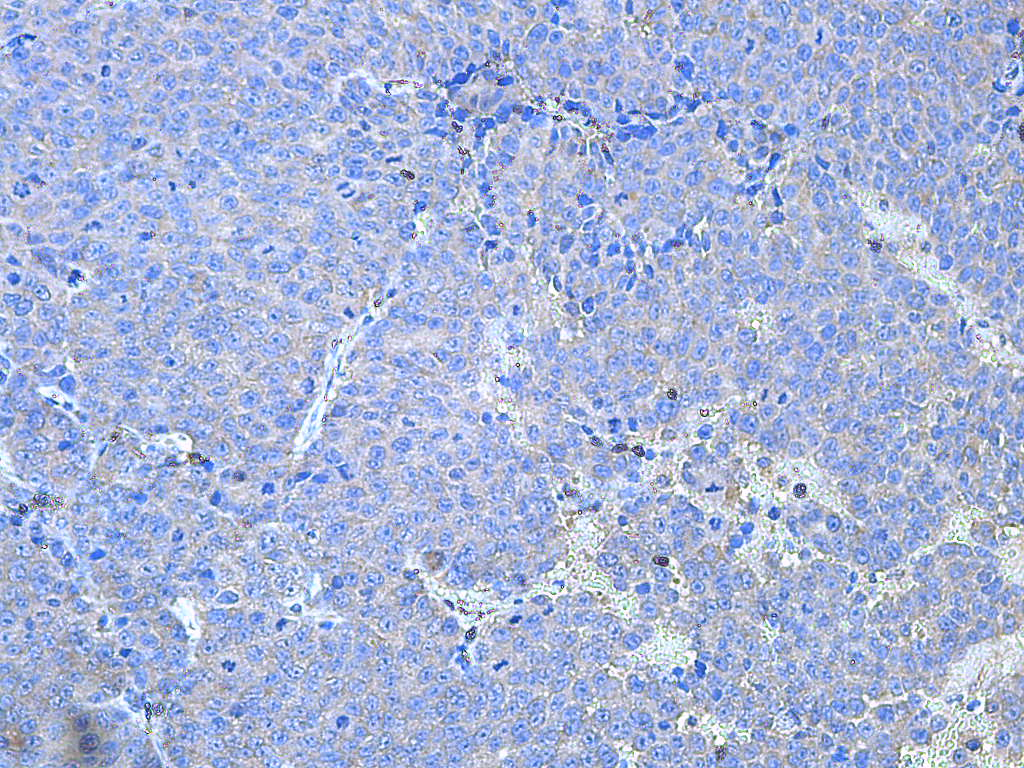

Supplement: Supplementary file 6 — Source data Fig. 4 [file 44321_2024_186_MOESM6_ESM.zip › Figure 4/4L/PlexinD1/shPlexinD1-2.tif]

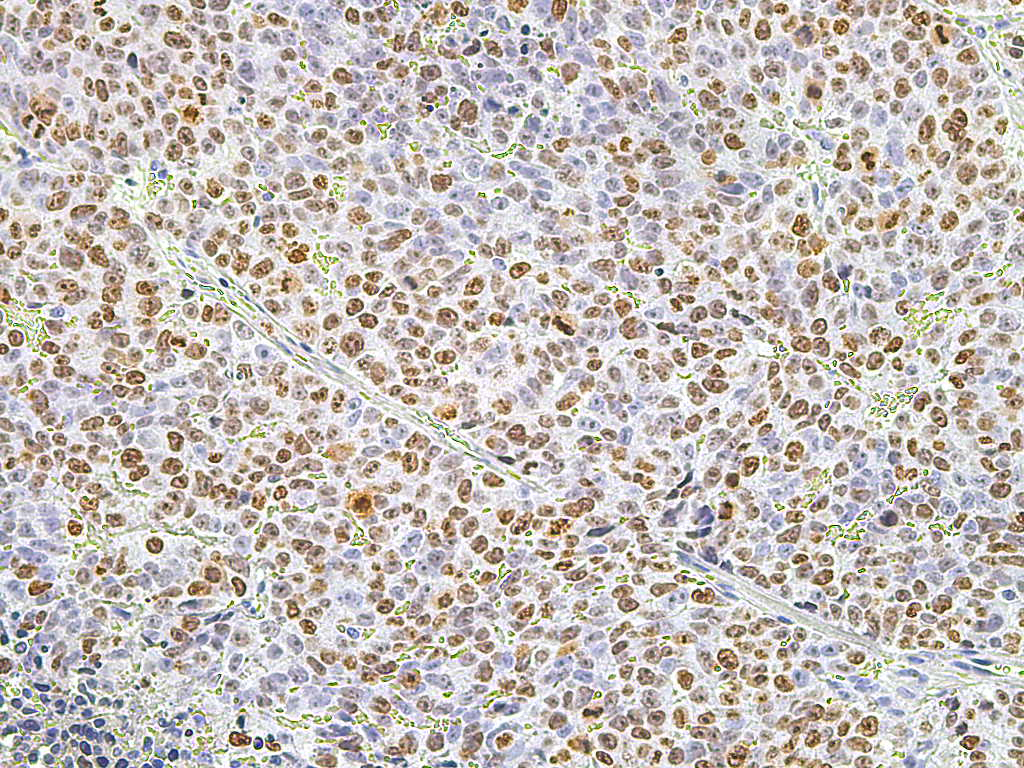

Supplement: Supplementary file 6 — Source data Fig. 4 [file 44321_2024_186_MOESM6_ESM.zip › Figure 4/4L/Ki-67/shCon.tif]

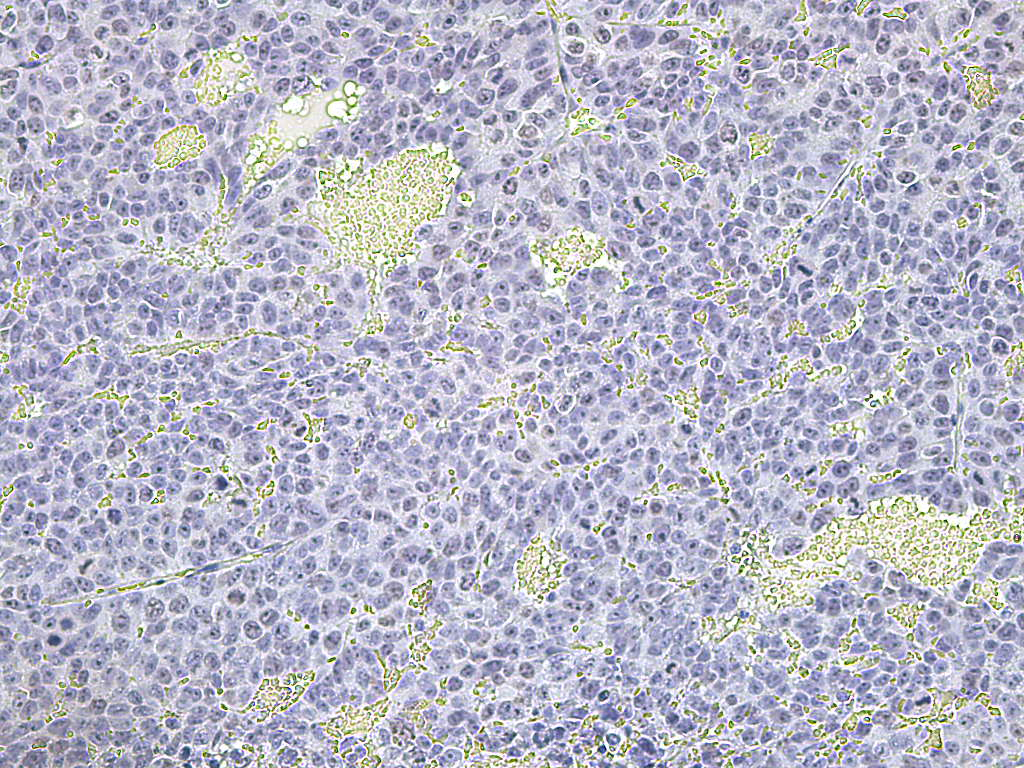

Supplement: Supplementary file 6 — Source data Fig. 4 [file 44321_2024_186_MOESM6_ESM.zip › Figure 4/4L/Ki-67/shPlexinD1-1.tif]

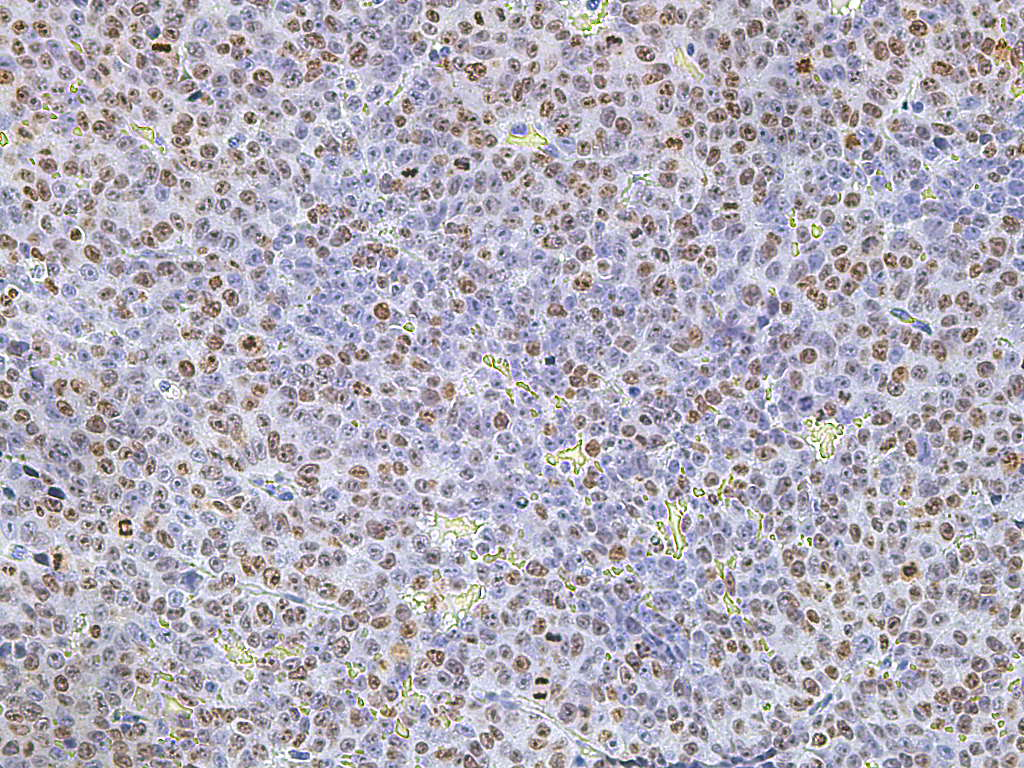

Supplement: Supplementary file 6 — Source data Fig. 4 [file 44321_2024_186_MOESM6_ESM.zip › Figure 4/4L/Ki-67/shPlexinD1-2.tif]

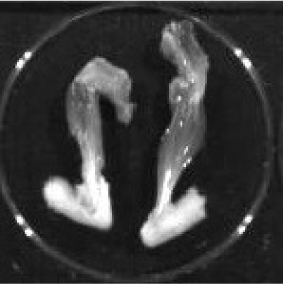

Supplement: Supplementary file 7 — Source data Fig. 5 [file 44321_2024_186_MOESM7_ESM.zip › Figure 5/5G/shPlexinD1-limb.tif]

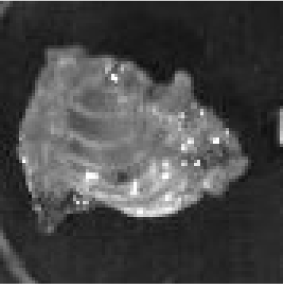

Supplement: Supplementary file 7 — Source data Fig. 5 [file 44321_2024_186_MOESM7_ESM.zip › Figure 5/5G/shPlexinD1-rib.tif]

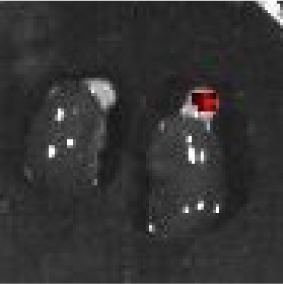

Supplement: Supplementary file 7 — Source data Fig. 5 [file 44321_2024_186_MOESM7_ESM.zip › Figure 5/5G/shPlexinD1-adrenal.tif]

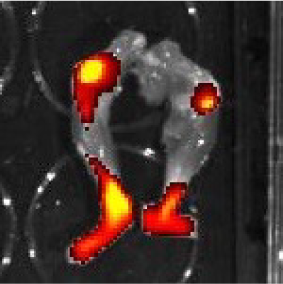

Supplement: Supplementary file 7 — Source data Fig. 5 [file 44321_2024_186_MOESM7_ESM.zip › Figure 5/5G/shCon-limb.tif]

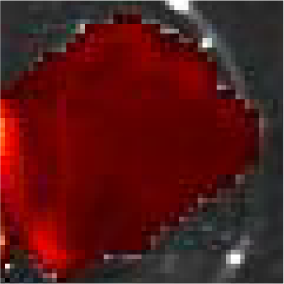

Supplement: Supplementary file 7 — Source data Fig. 5 [file 44321_2024_186_MOESM7_ESM.zip › Figure 5/5G/shCon-rib.tif]

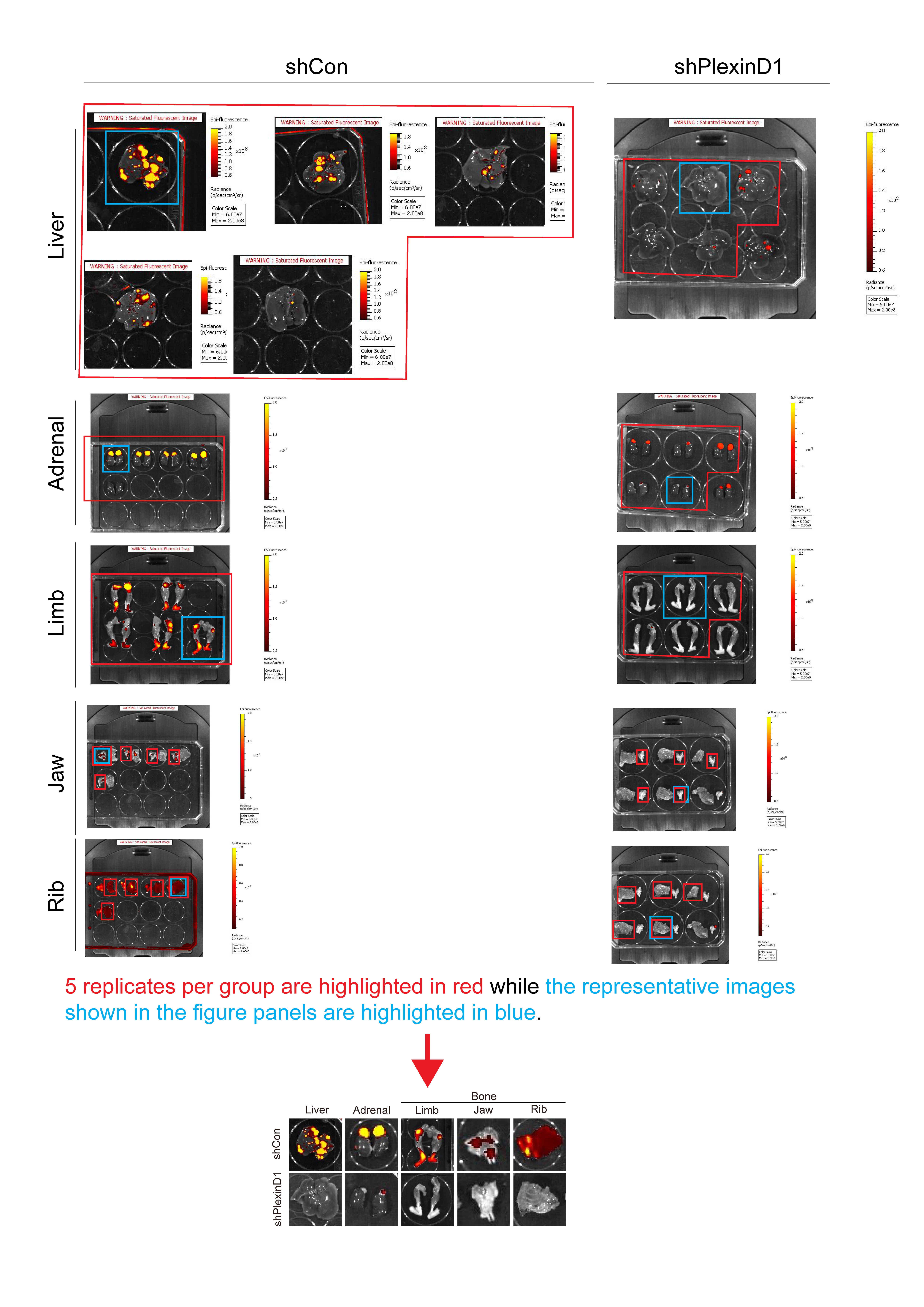

Supplement: Supplementary file 7 — Source data Fig. 5 [file 44321_2024_186_MOESM7_ESM.zip › Figure 5/5G/README.tif]

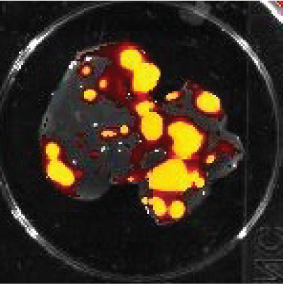

Supplement: Supplementary file 7 — Source data Fig. 5 [file 44321_2024_186_MOESM7_ESM.zip › Figure 5/5G/shCon-liver.tif]

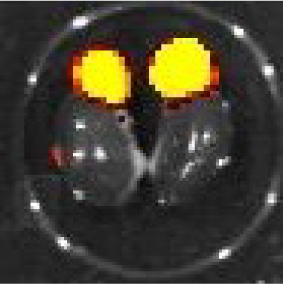

Supplement: Supplementary file 7 — Source data Fig. 5 [file 44321_2024_186_MOESM7_ESM.zip › Figure 5/5G/shCon-adrenal.tif]

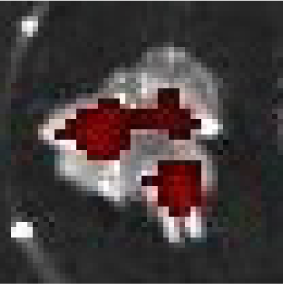

Supplement: Supplementary file 7 — Source data Fig. 5 [file 44321_2024_186_MOESM7_ESM.zip › Figure 5/5G/shCon-jaw.tif]

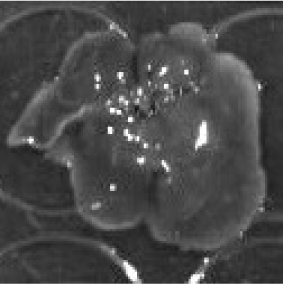

Supplement: Supplementary file 7 — Source data Fig. 5 [file 44321_2024_186_MOESM7_ESM.zip › Figure 5/5G/shPlexinD1-liver.tif]

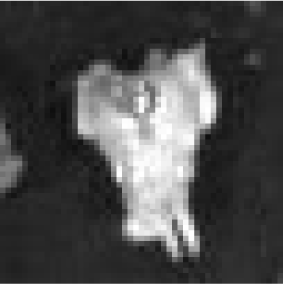

Supplement: Supplementary file 7 — Source data Fig. 5 [file 44321_2024_186_MOESM7_ESM.zip › Figure 5/5G/shPlexinD1-jaw.tif]

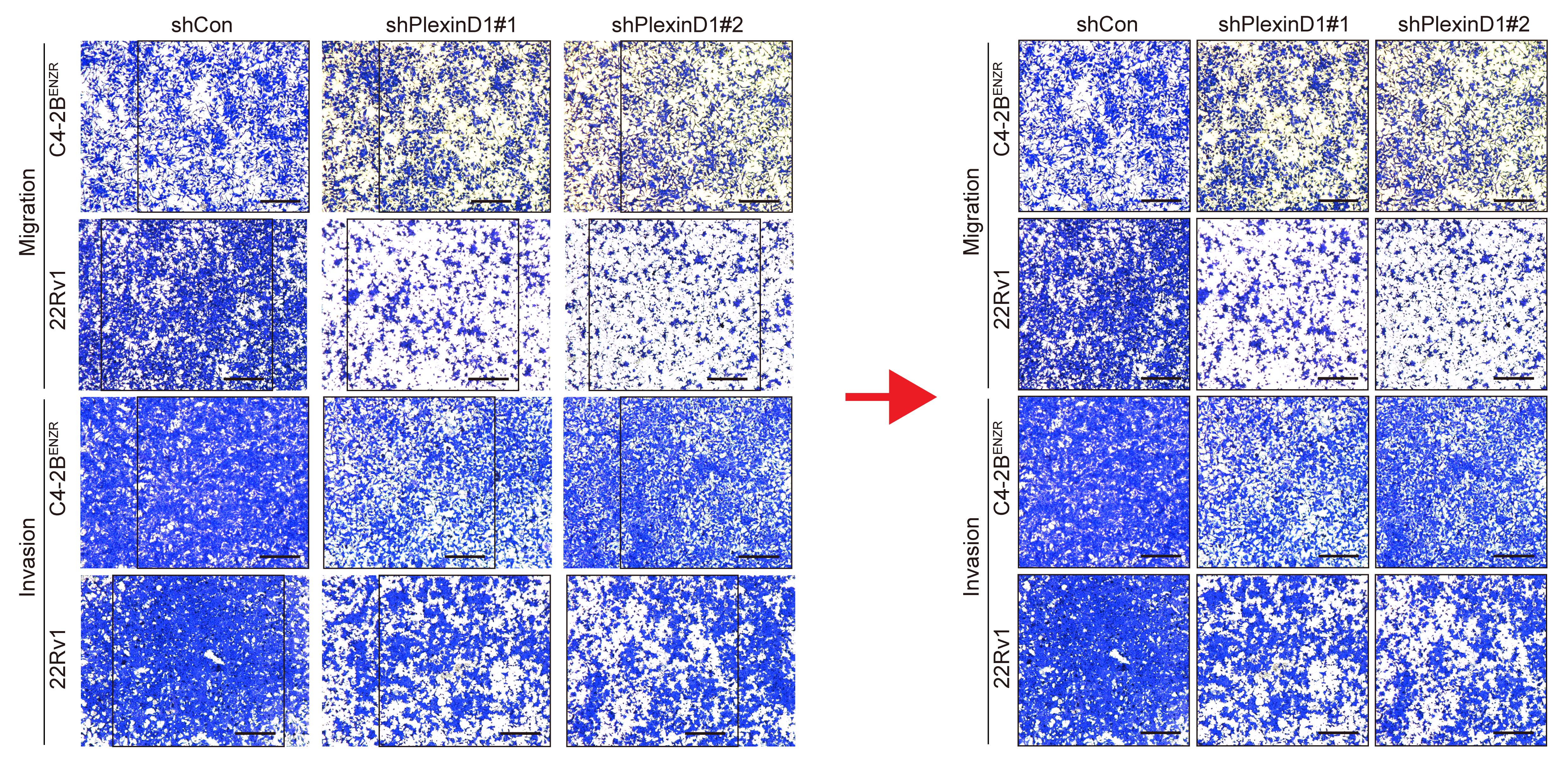

Supplement: Supplementary file 7 — Source data Fig. 5 [file 44321_2024_186_MOESM7_ESM.zip › Figure 5/5A/README.tif]

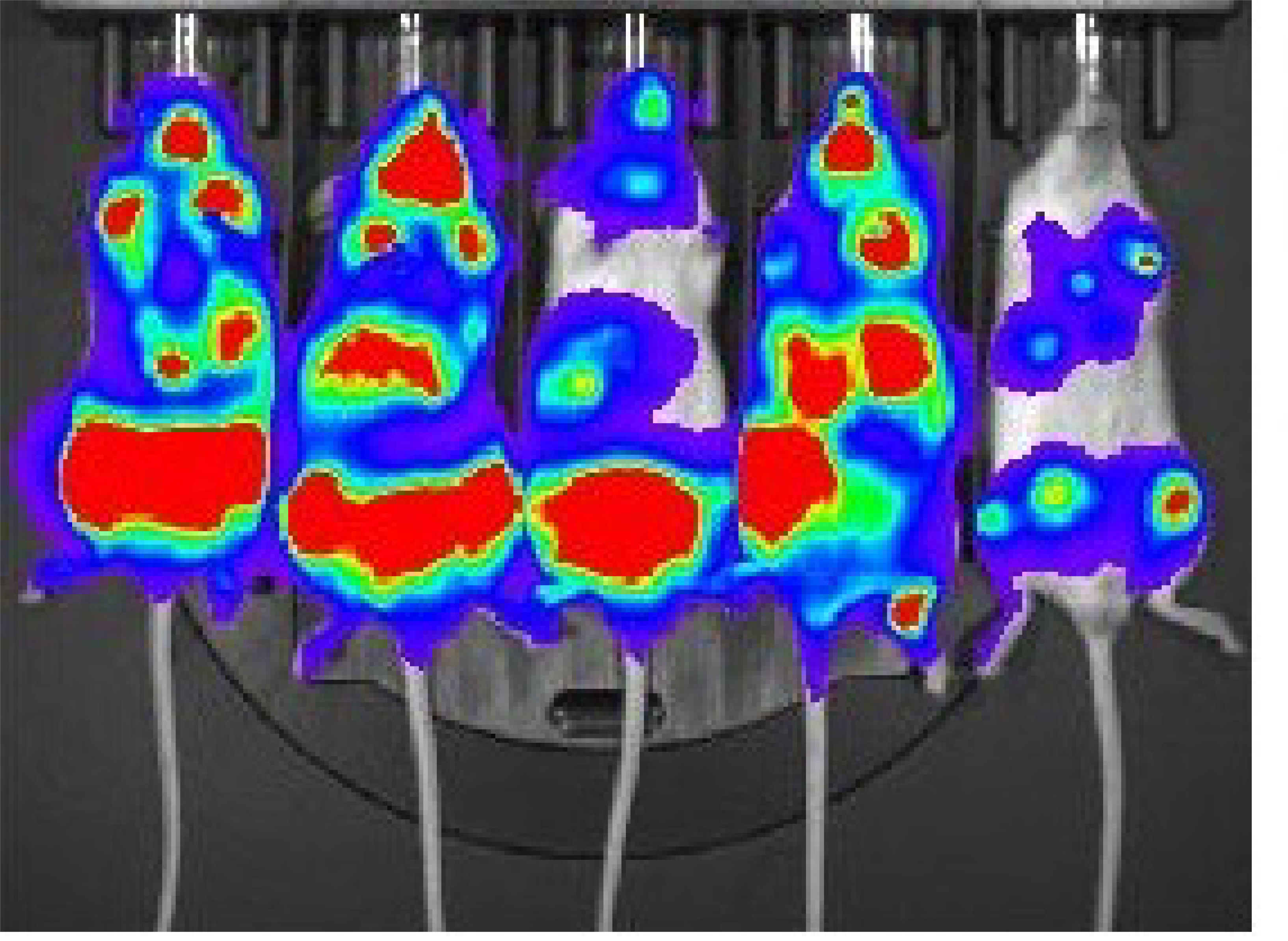

Supplement: Supplementary file 7 — Source data Fig. 5 [file 44321_2024_186_MOESM7_ESM.zip › Figure 5/5F/IVIS-shCon.tif]

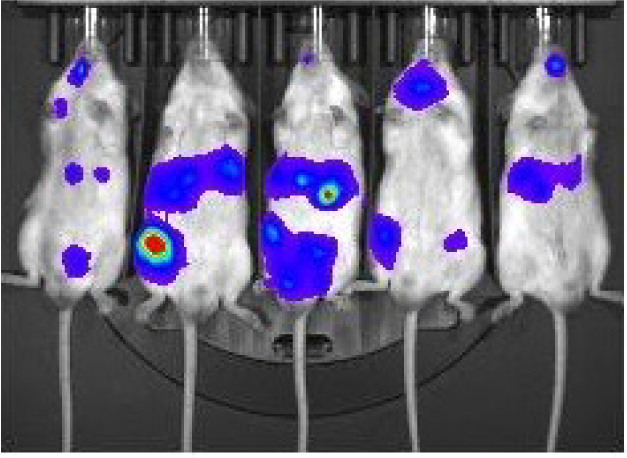

Supplement: Supplementary file 7 — Source data Fig. 5 [file 44321_2024_186_MOESM7_ESM.zip › Figure 5/5F/IVIS-shPlexinD1.tif]

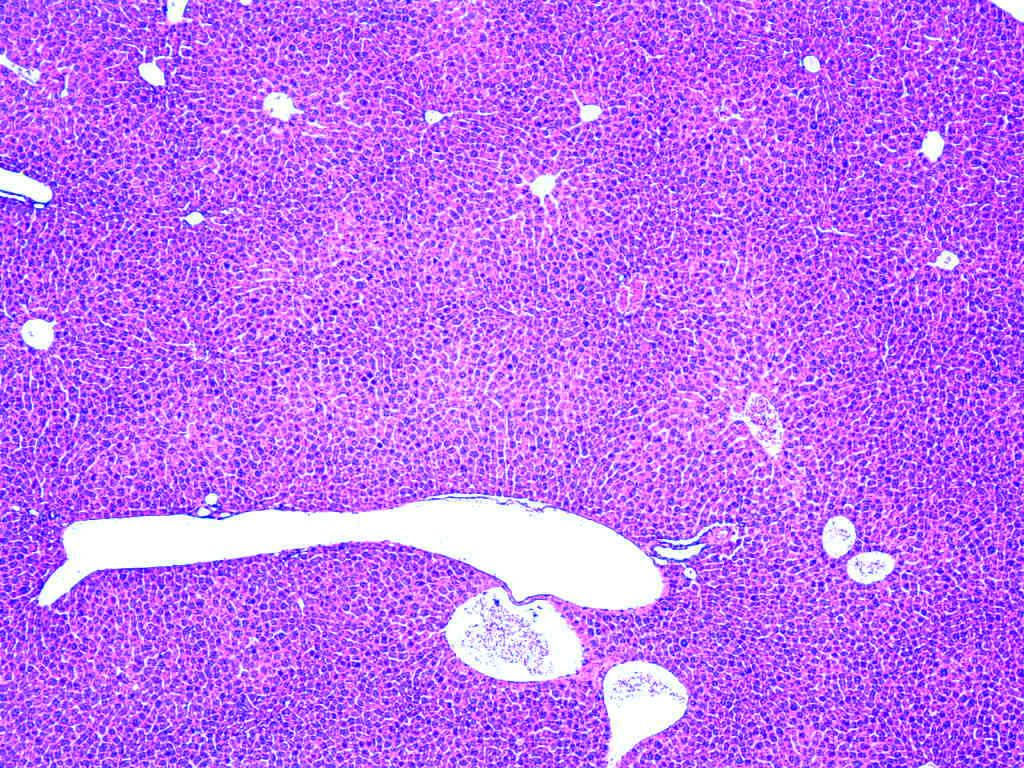

Supplement: Supplementary file 7 — Source data Fig. 5 [file 44321_2024_186_MOESM7_ESM.zip › Figure 5/5H/HE_liver_shPlexinD1.tif]

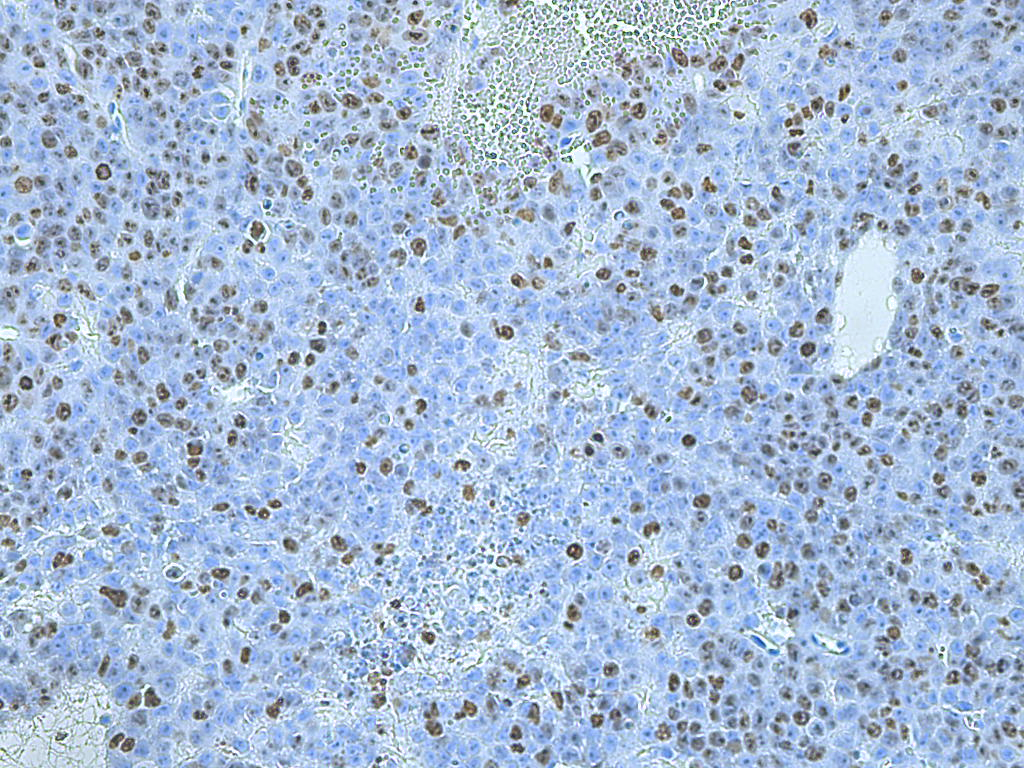

Supplement: Supplementary file 7 — Source data Fig. 5 [file 44321_2024_186_MOESM7_ESM.zip › Figure 5/5H/ki67_adrenal gland_shPlexinD1.tif]

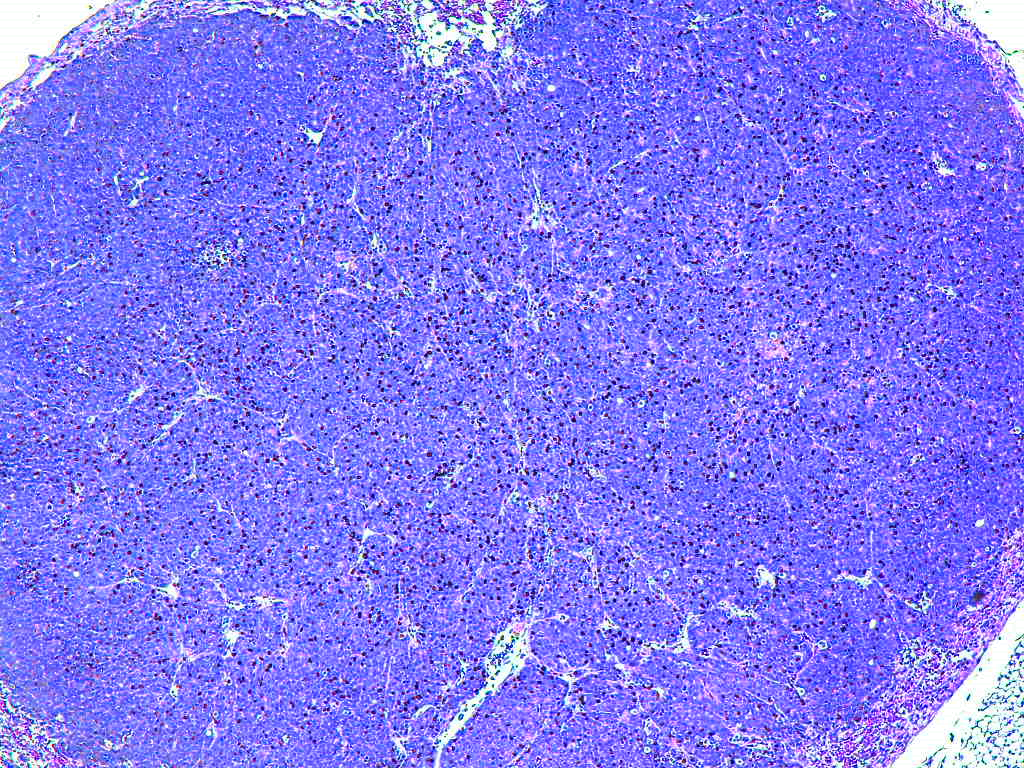

Supplement: Supplementary file 7 — Source data Fig. 5 [file 44321_2024_186_MOESM7_ESM.zip › Figure 5/5H/HE_Adrenal gland_shCon.tif]

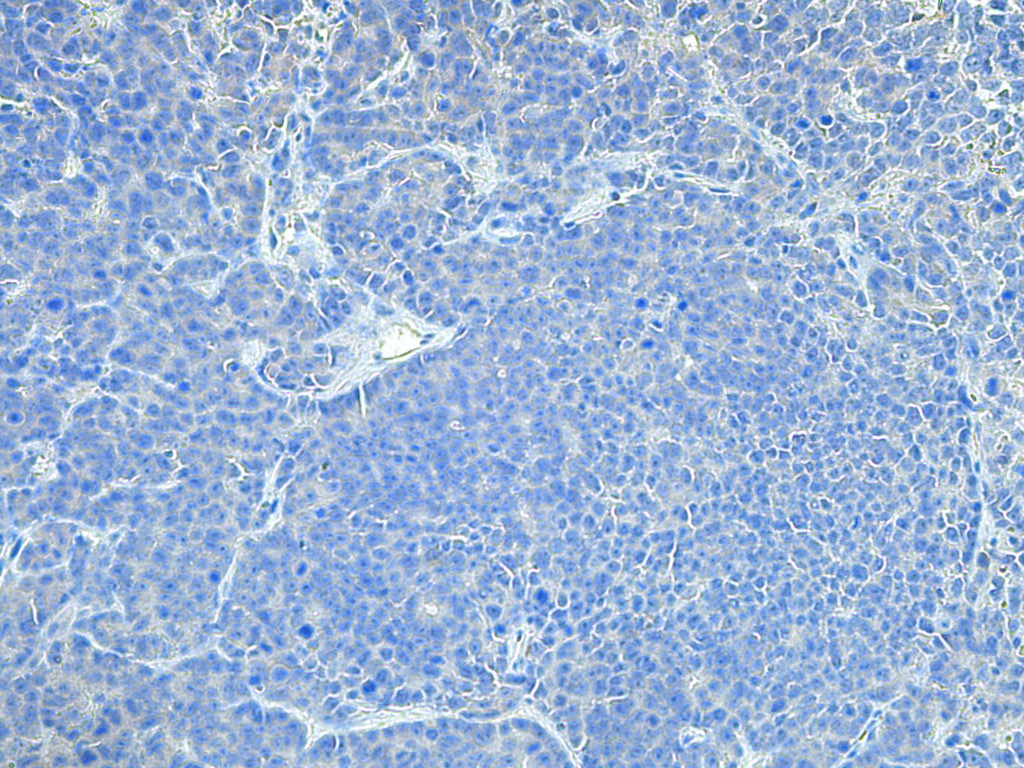

Supplement: Supplementary file 7 — Source data Fig. 5 [file 44321_2024_186_MOESM7_ESM.zip › Figure 5/5H/PlexinD1 IHC_liver_shPlexinD1.tif]

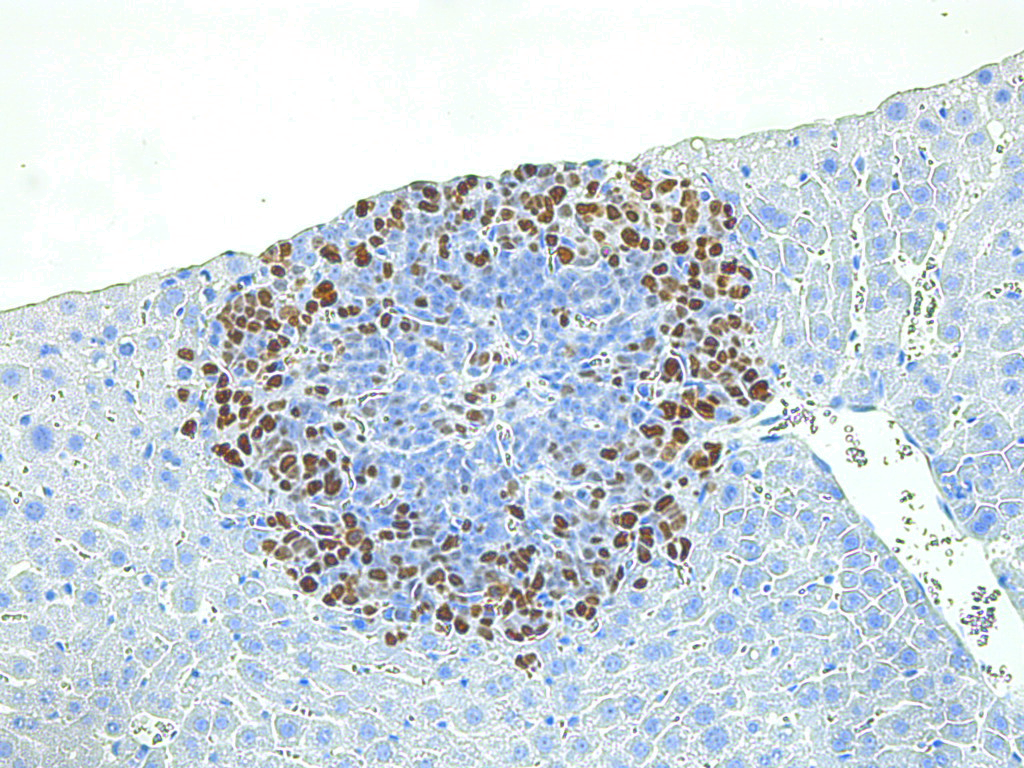

Supplement: Supplementary file 7 — Source data Fig. 5 [file 44321_2024_186_MOESM7_ESM.zip › Figure 5/5H/ki67_liver_shPlexinD1.tif]

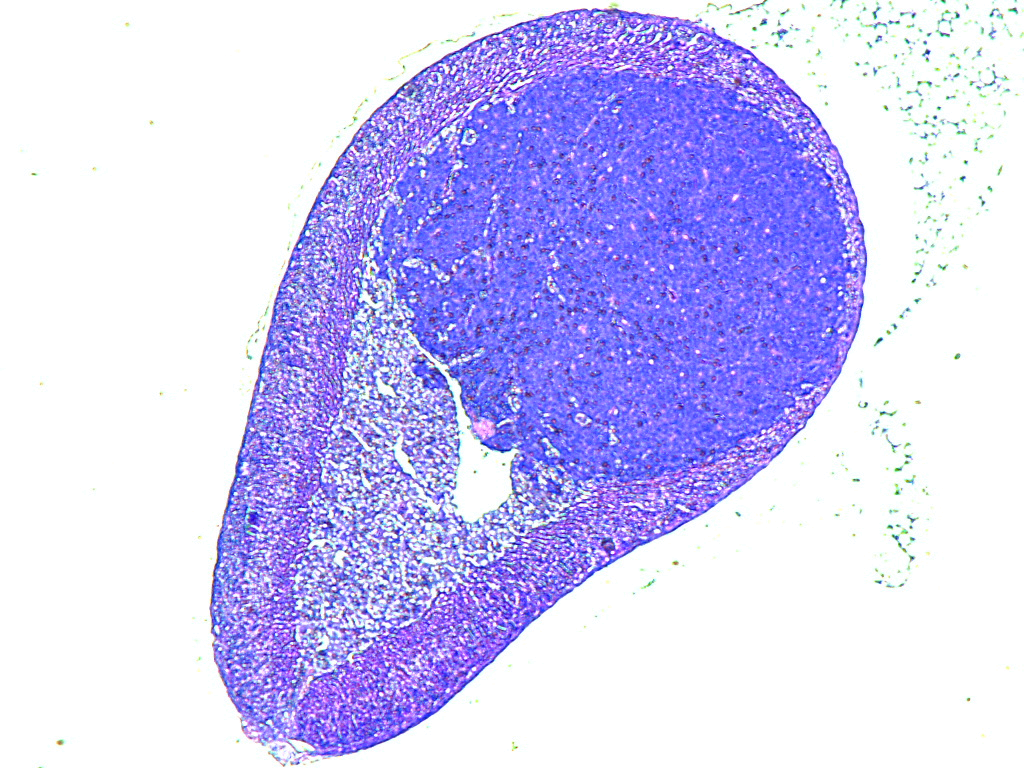

Supplement: Supplementary file 7 — Source data Fig. 5 [file 44321_2024_186_MOESM7_ESM.zip › Figure 5/5H/HE_Adrenal gland_shPlexinD1.tif]

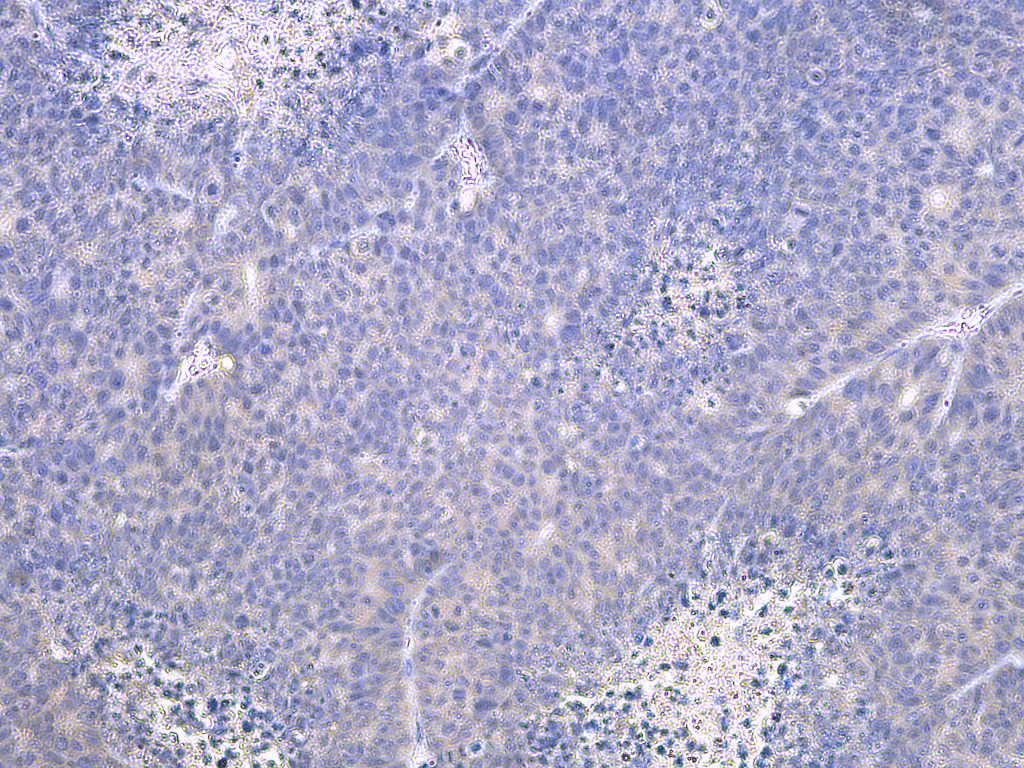

Supplement: Supplementary file 7 — Source data Fig. 5 [file 44321_2024_186_MOESM7_ESM.zip › Figure 5/5H/PlexinD1 IHC_adrenal gland_shPlexinD1.tif]

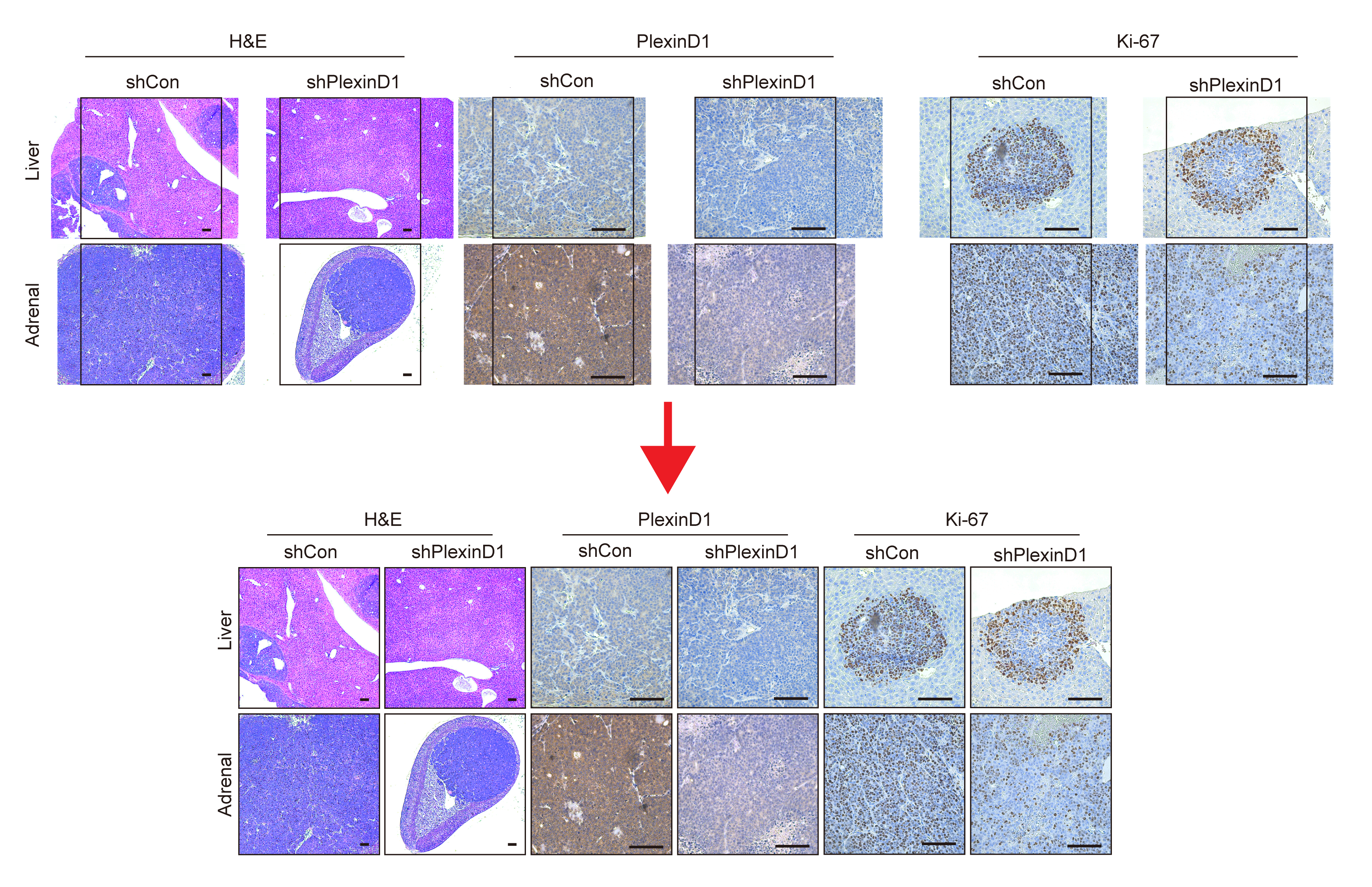

Supplement: Supplementary file 7 — Source data Fig. 5 [file 44321_2024_186_MOESM7_ESM.zip › Figure 5/5H/README.tif]

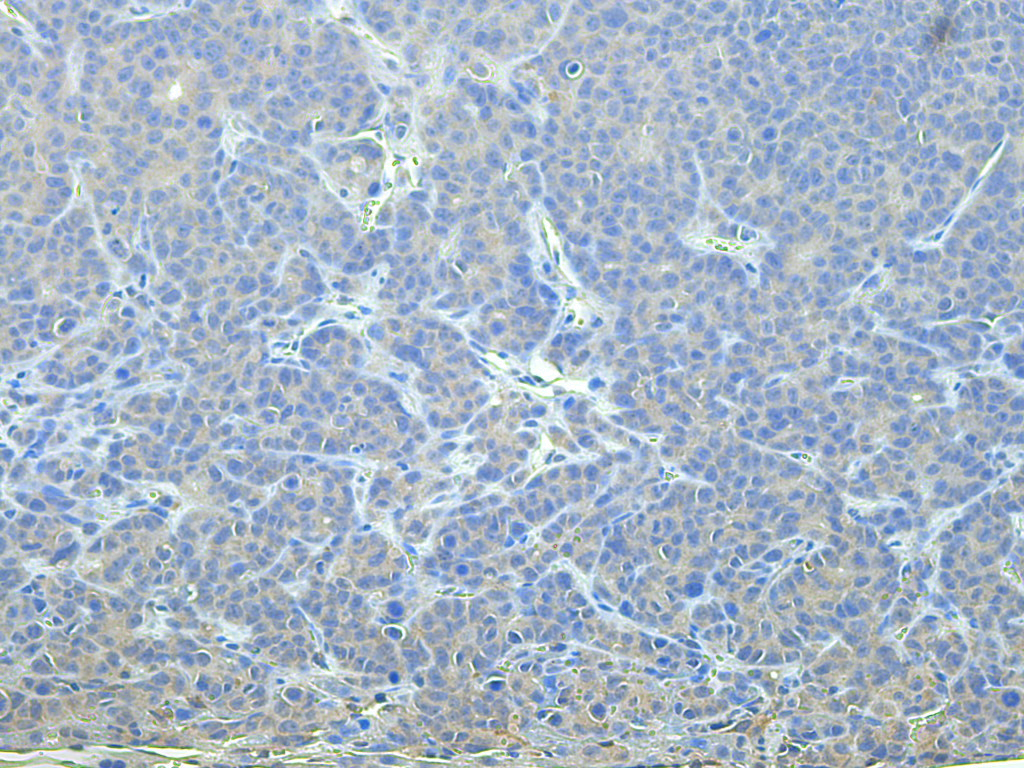

Supplement: Supplementary file 7 — Source data Fig. 5 [file 44321_2024_186_MOESM7_ESM.zip › Figure 5/5H/PlexinD1 IHC_liver_shCon.tif]

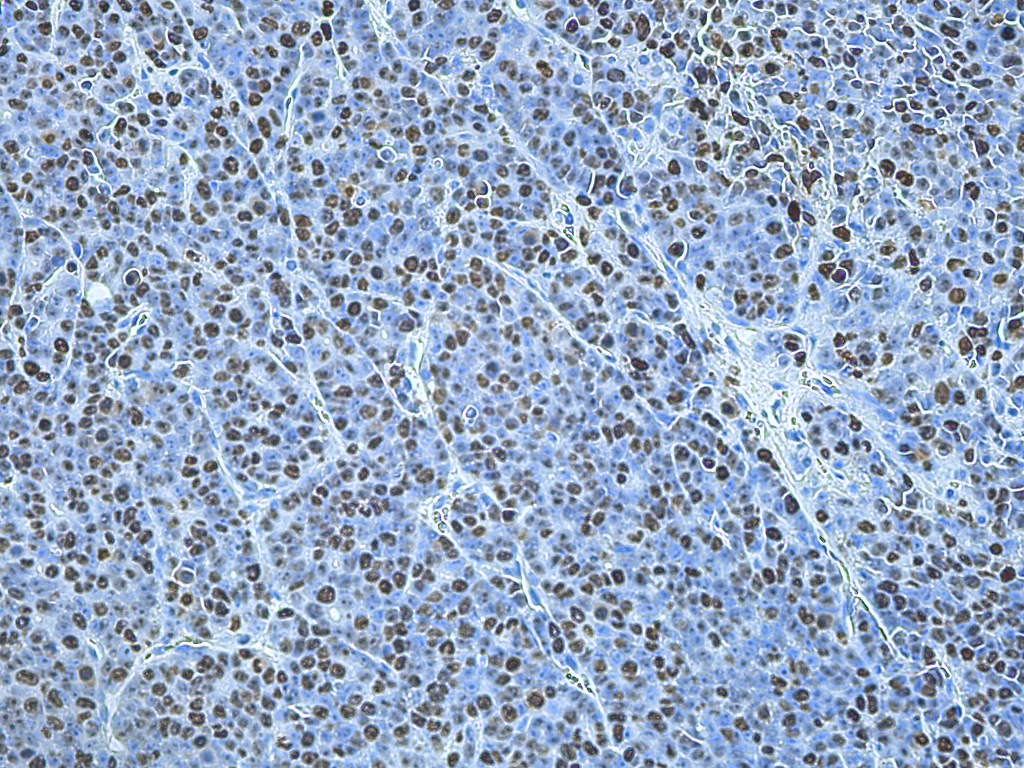

Supplement: Supplementary file 7 — Source data Fig. 5 [file 44321_2024_186_MOESM7_ESM.zip › Figure 5/5H/ki67_adrenal gland_shCon.tif]

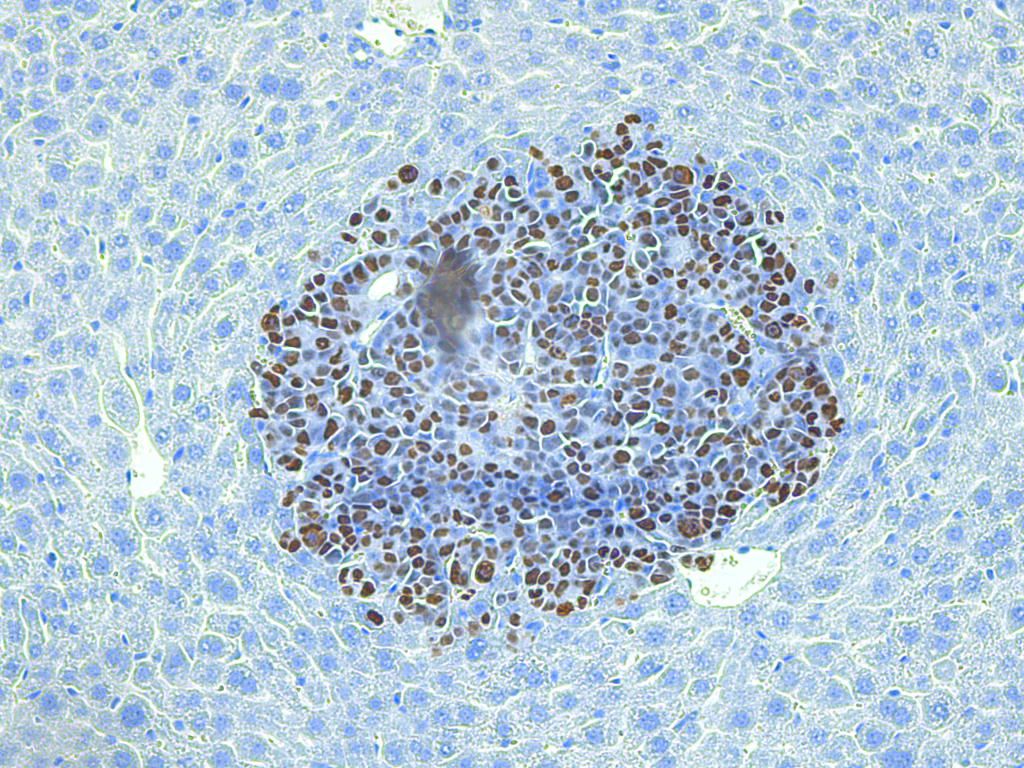

Supplement: Supplementary file 7 — Source data Fig. 5 [file 44321_2024_186_MOESM7_ESM.zip › Figure 5/5H/ki67_liver_shCon.tif]

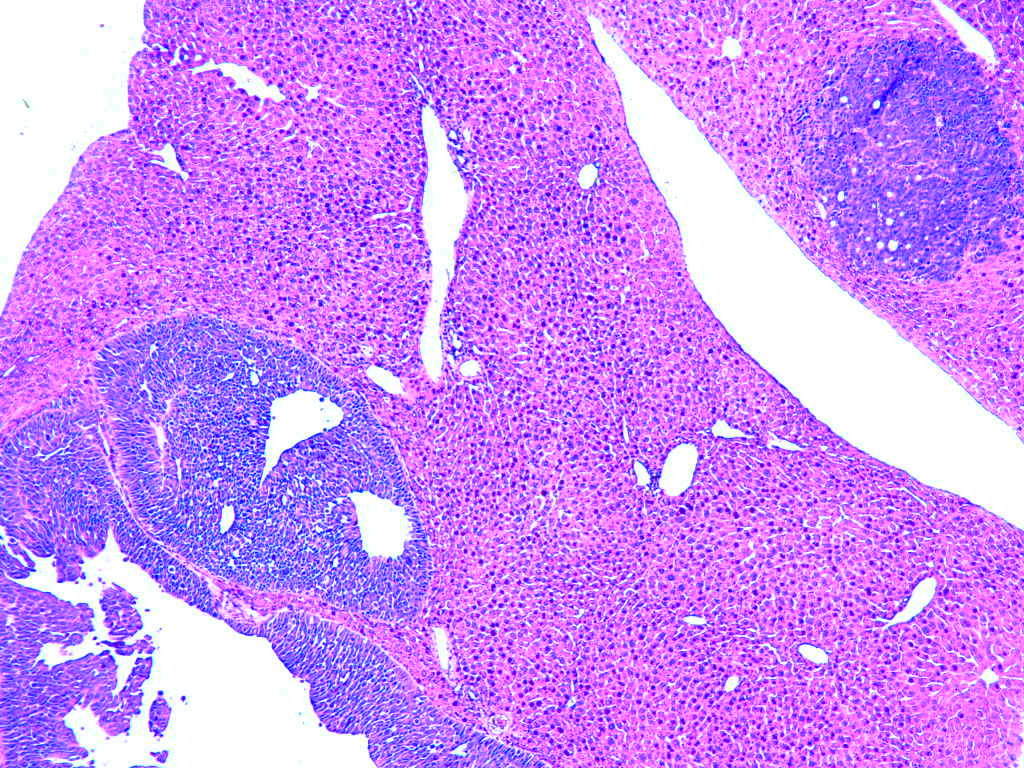

Supplement: Supplementary file 7 — Source data Fig. 5 [file 44321_2024_186_MOESM7_ESM.zip › Figure 5/5H/HE_liver_shCon.tif]

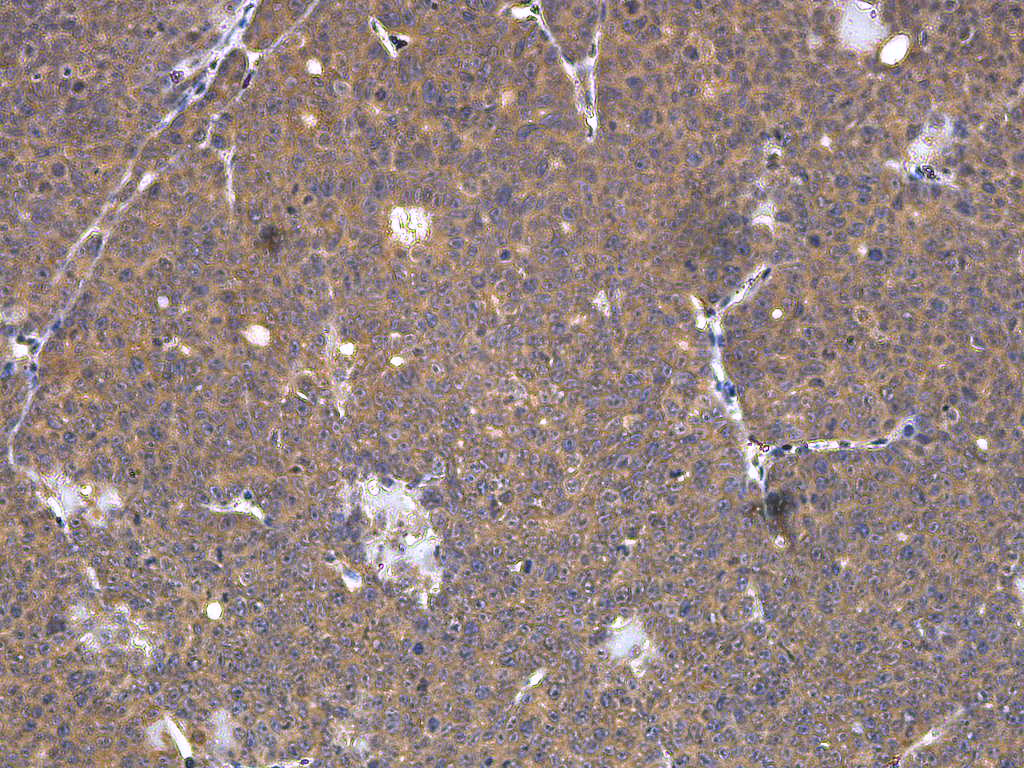

Supplement: Supplementary file 7 — Source data Fig. 5 [file 44321_2024_186_MOESM7_ESM.zip › Figure 5/5H/PlexinD1 IHC_adrenal gland_shCon.tif]

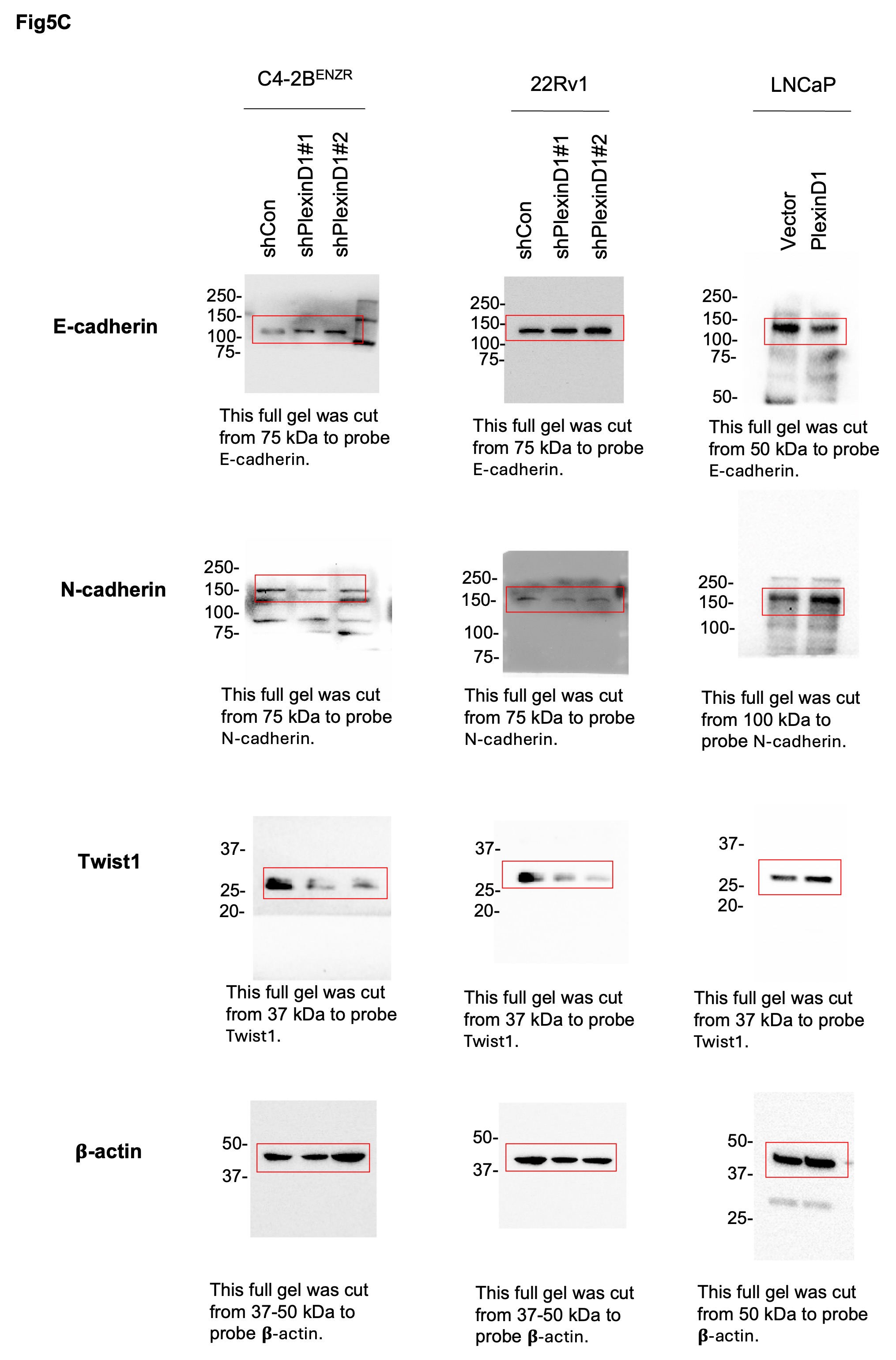

Supplement: Supplementary file 7 — Source data Fig. 5 [file 44321_2024_186_MOESM7_ESM.zip › Figure 5/5C/WB-5C.jpg]

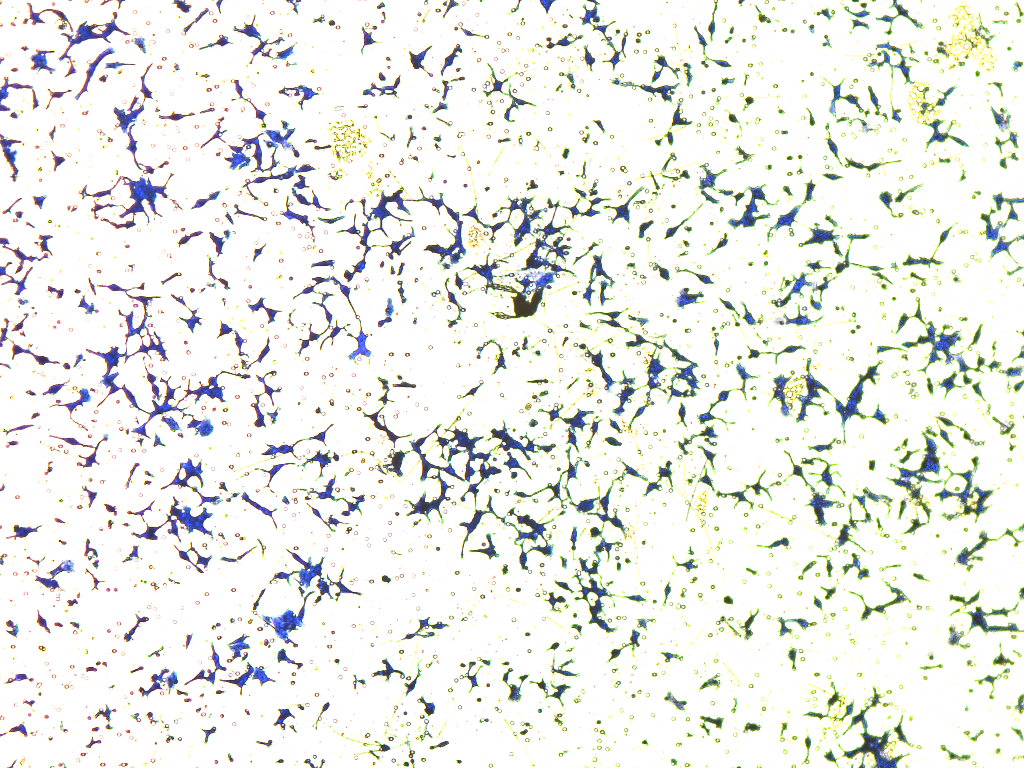

Supplement: Supplementary file 7 — Source data Fig. 5 [file 44321_2024_186_MOESM7_ESM.zip › Figure 5/5B/Migration_Vector.tif]

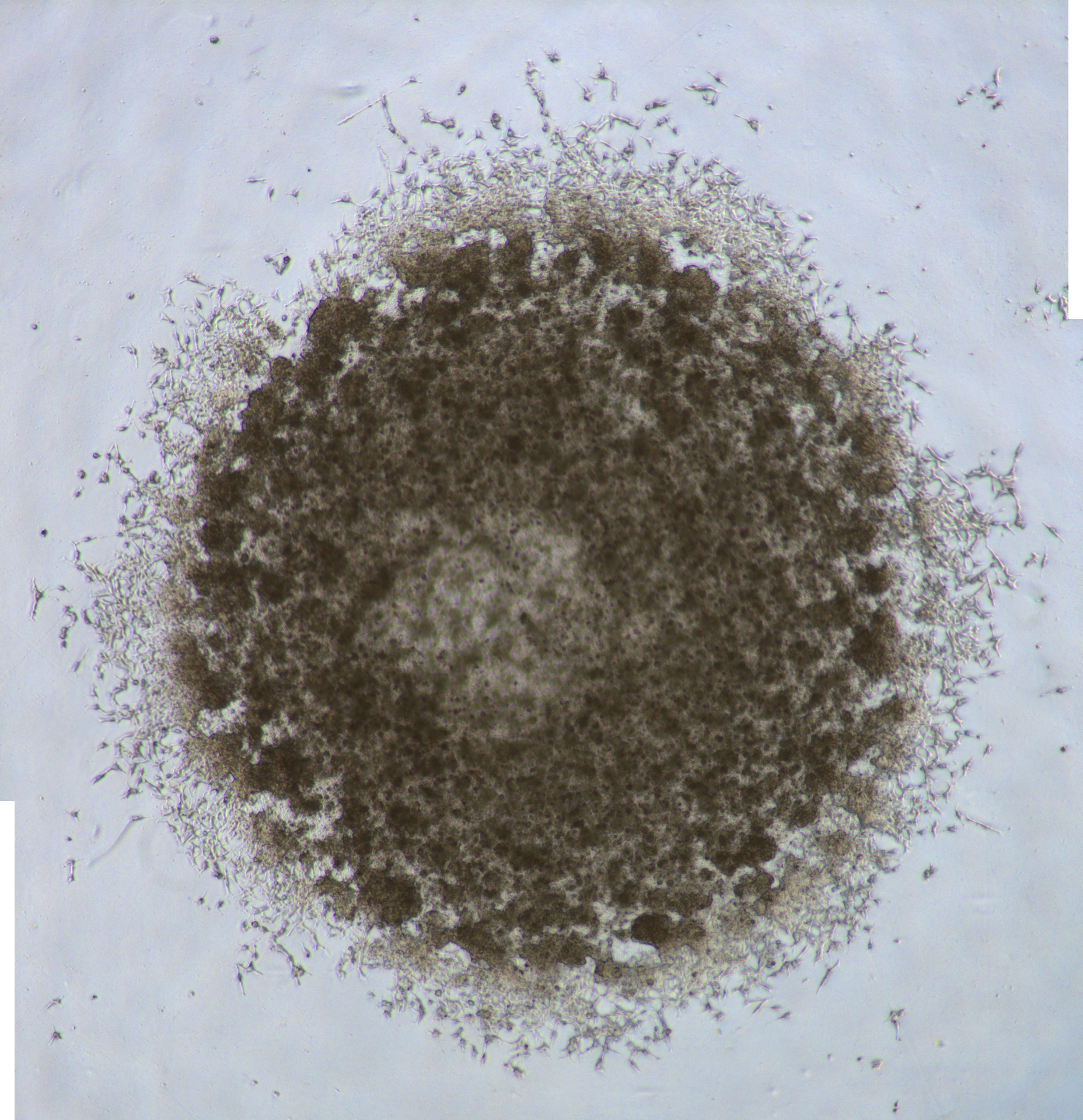

Supplement: Supplementary file 7 — Source data Fig. 5 [file 44321_2024_186_MOESM7_ESM.zip › Figure 5/5B/Invasion_PlexinD1.tif]

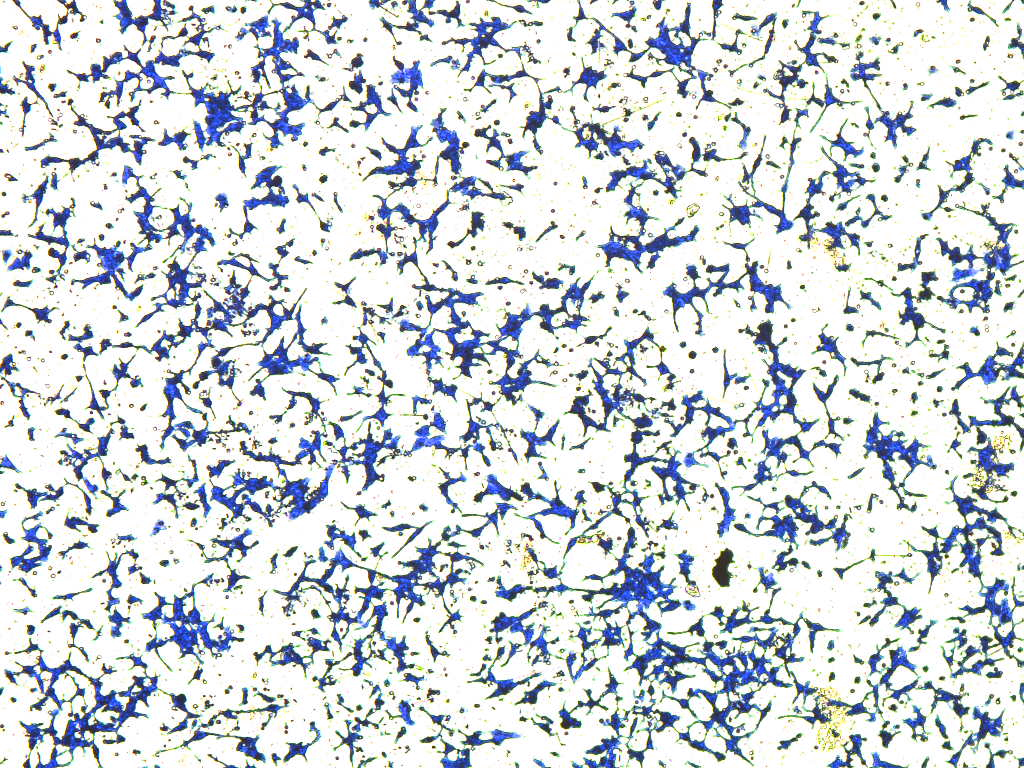

Supplement: Supplementary file 7 — Source data Fig. 5 [file 44321_2024_186_MOESM7_ESM.zip › Figure 5/5B/Migration_PlexinD1.tif]

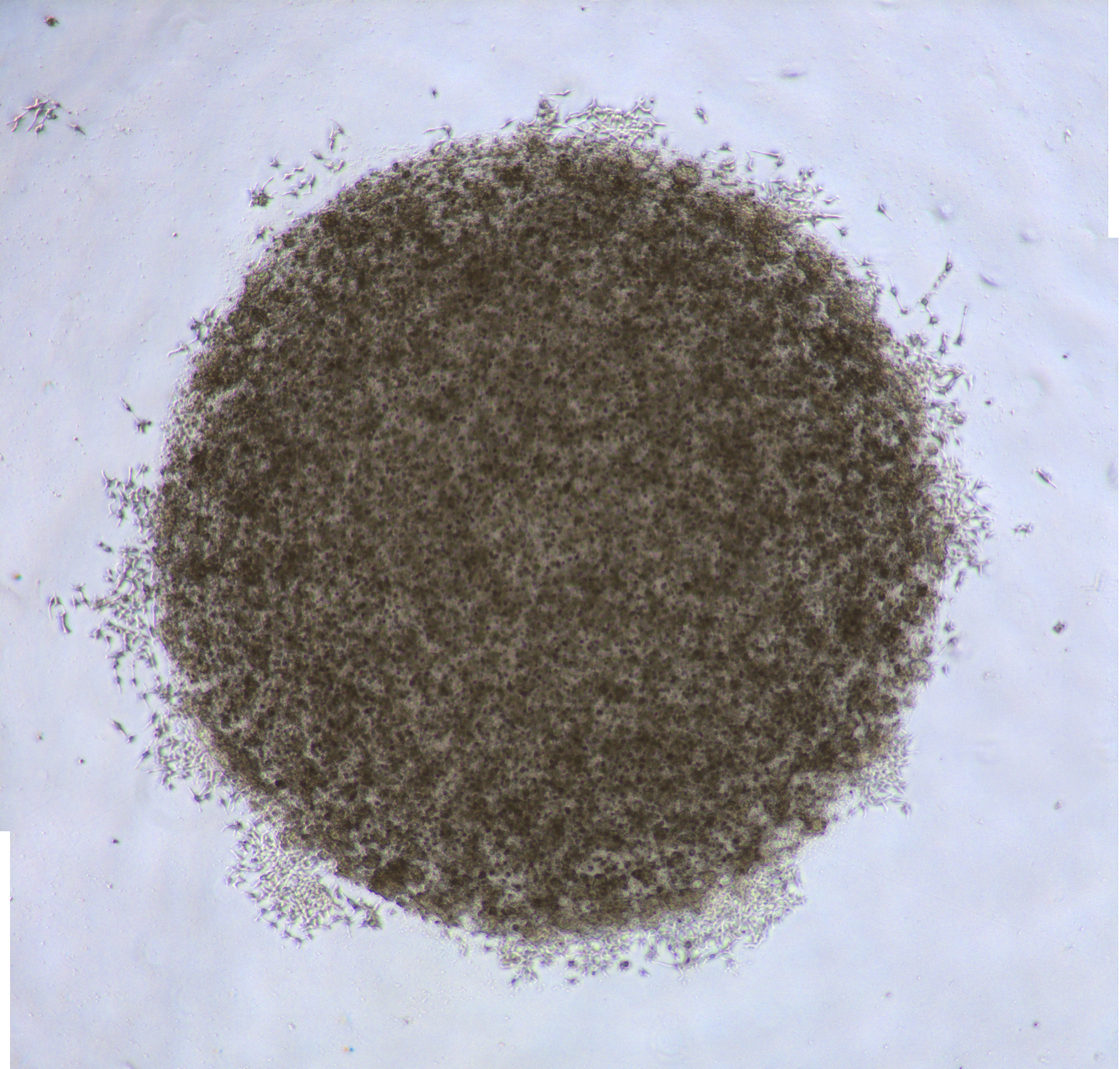

Supplement: Supplementary file 7 — Source data Fig. 5 [file 44321_2024_186_MOESM7_ESM.zip › Figure 5/5B/Invasion_Vector.tif]

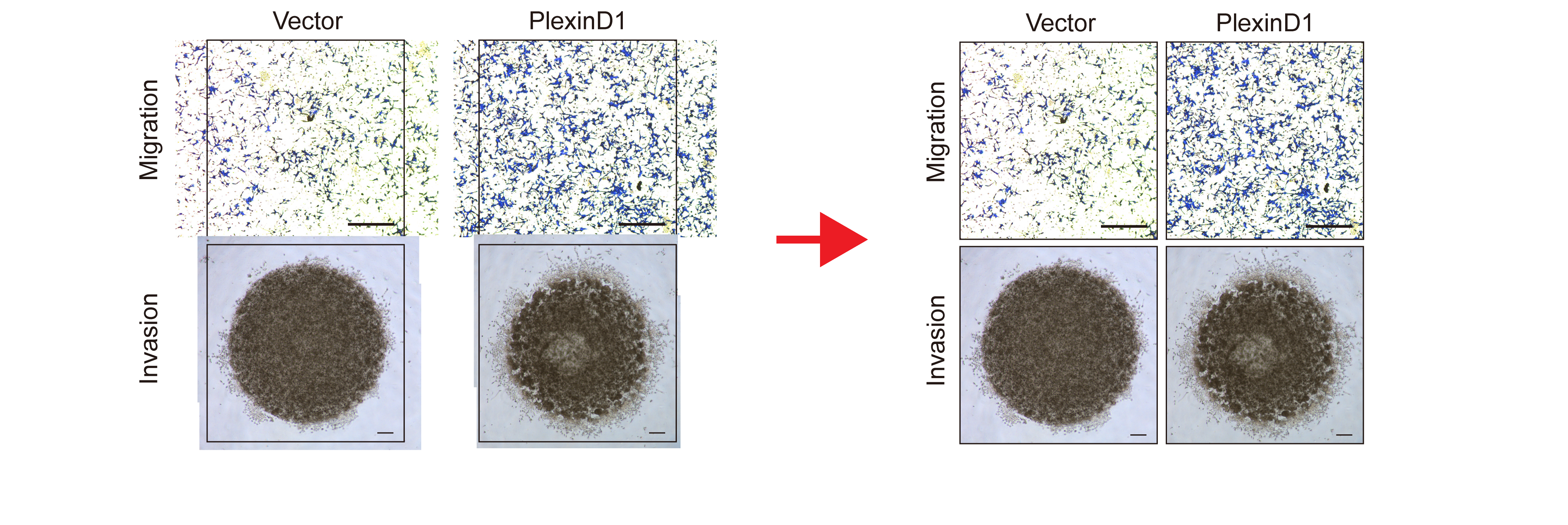

Supplement: Supplementary file 7 — Source data Fig. 5 [file 44321_2024_186_MOESM7_ESM.zip › Figure 5/5B/README.tif]

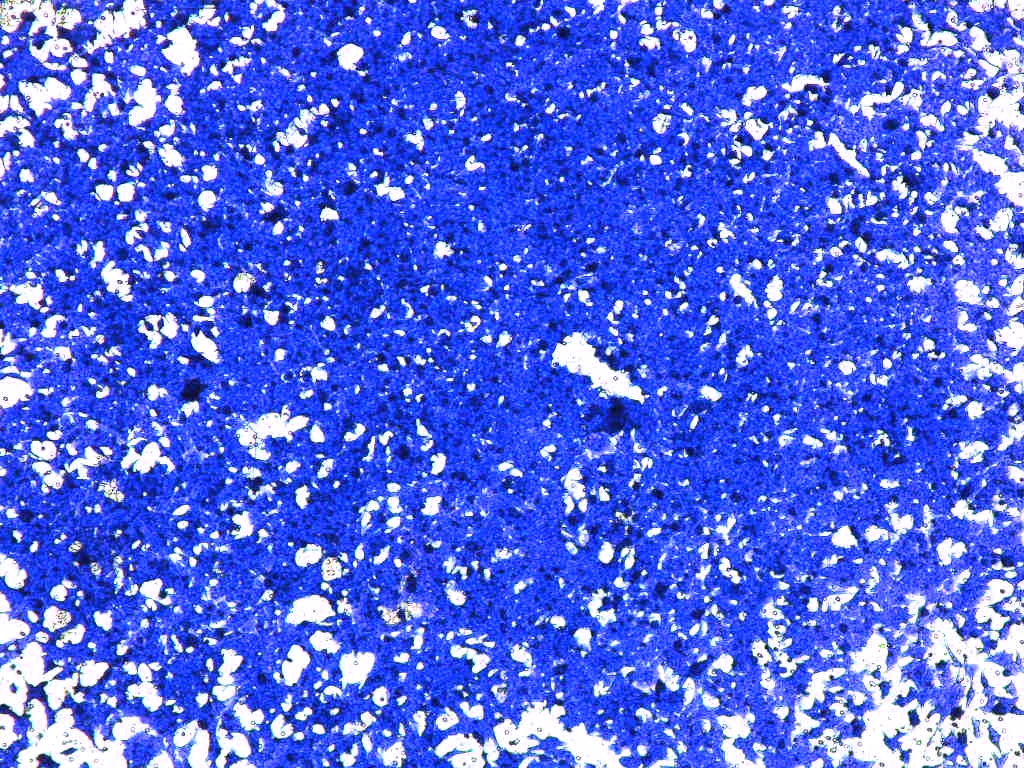

Supplement: Supplementary file 7 — Source data Fig. 5 [file 44321_2024_186_MOESM7_ESM.zip › Figure 5/5A/Invasion/22Rv1 shC.tif]

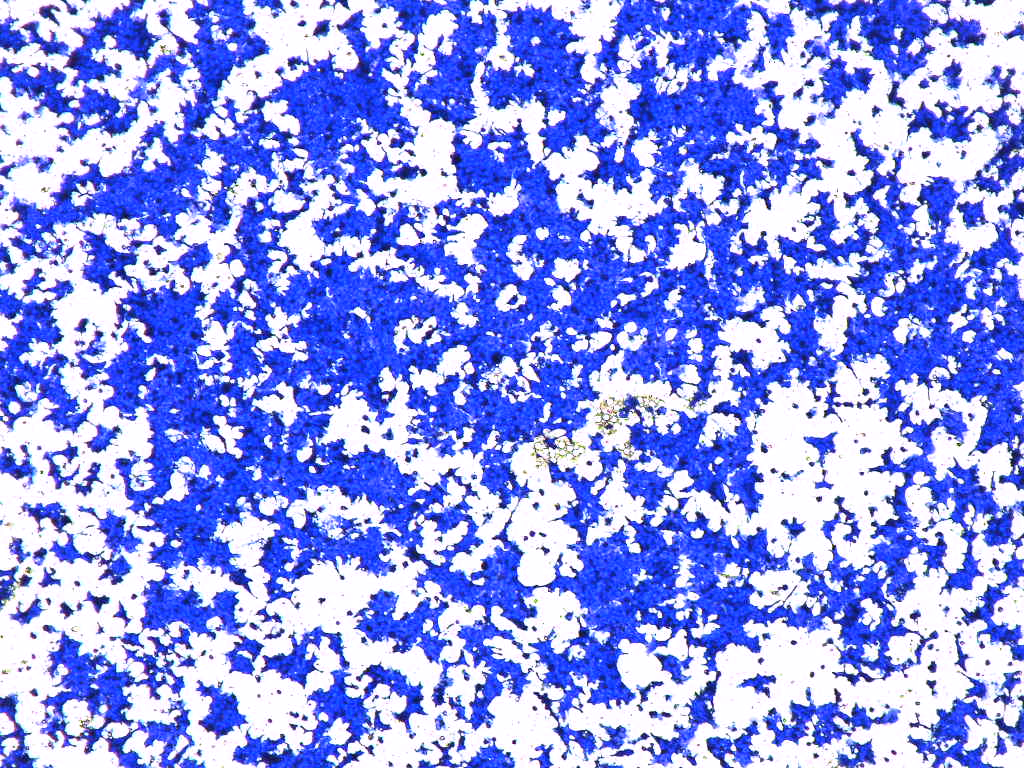

Supplement: Supplementary file 7 — Source data Fig. 5 [file 44321_2024_186_MOESM7_ESM.zip › Figure 5/5A/Invasion/22Rv1 shPlexinD1-1.tif]

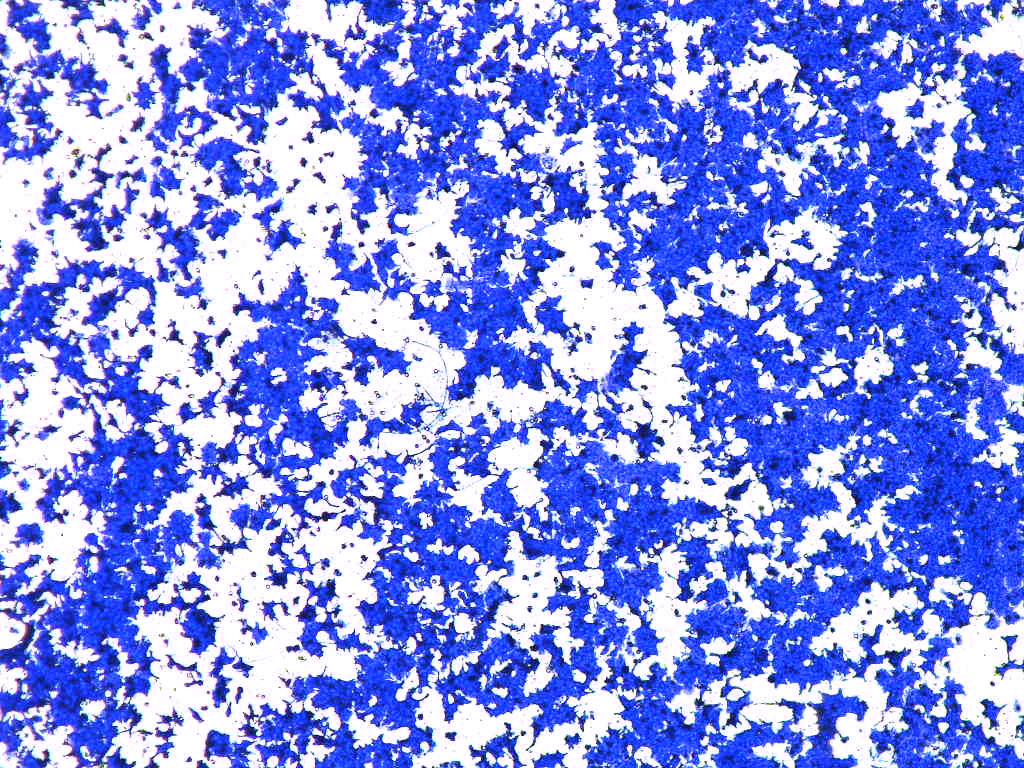

Supplement: Supplementary file 7 — Source data Fig. 5 [file 44321_2024_186_MOESM7_ESM.zip › Figure 5/5A/Invasion/22Rv1 shPlexinD1-2.tif]

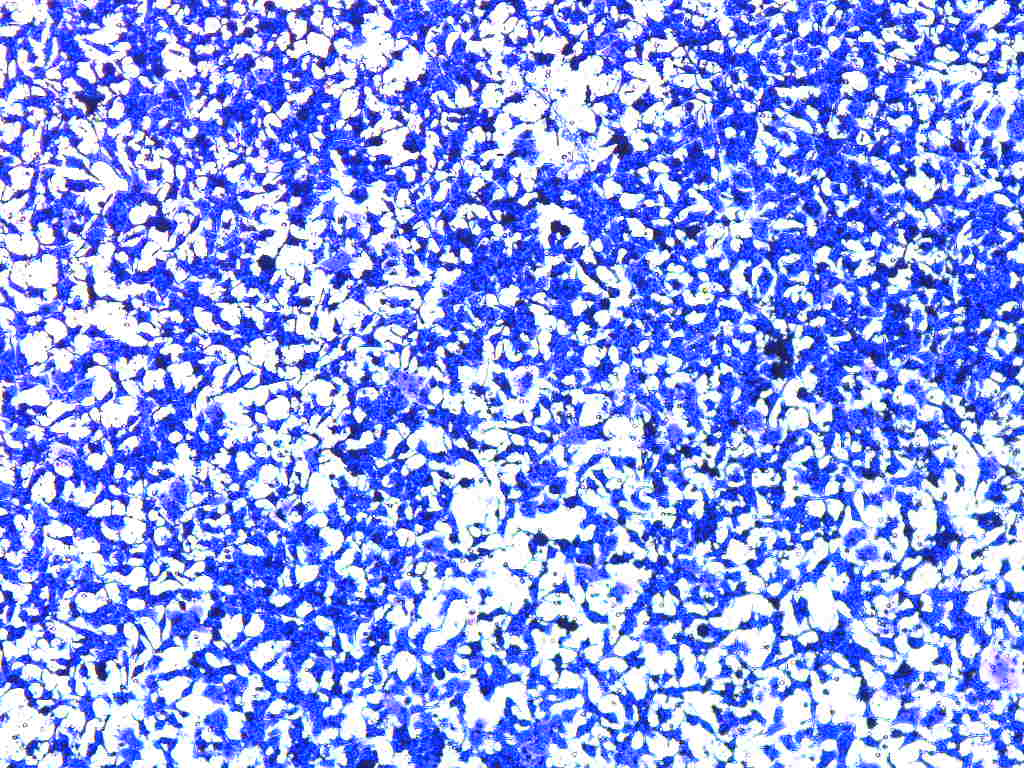

Supplement: Supplementary file 7 — Source data Fig. 5 [file 44321_2024_186_MOESM7_ESM.zip › Figure 5/5A/Invasion/C4-2BENZR shPlexinD1-1.tif]

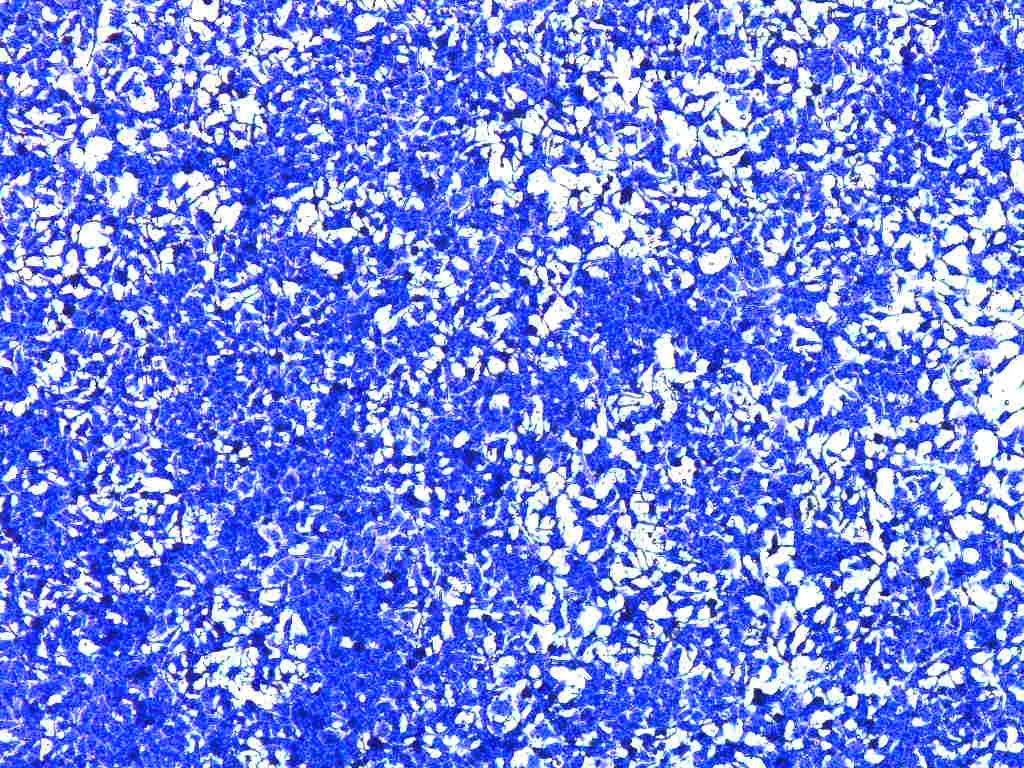

Supplement: Supplementary file 7 — Source data Fig. 5 [file 44321_2024_186_MOESM7_ESM.zip › Figure 5/5A/Invasion/C4-2BENZR shPlexinD1-2.tif]

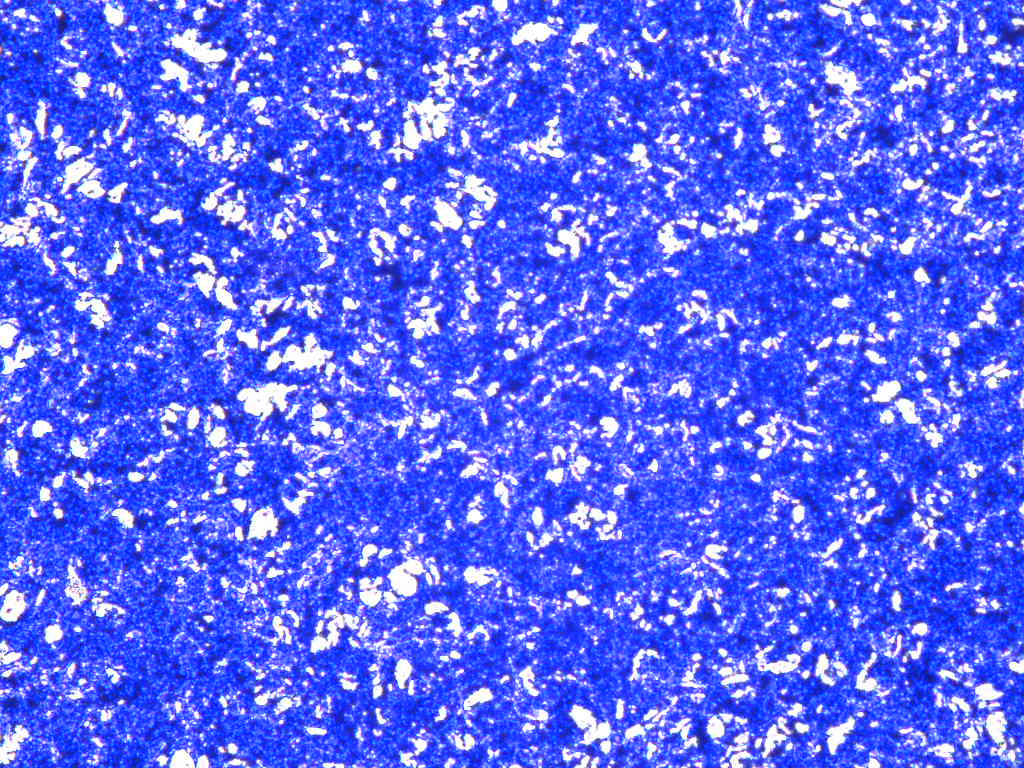

Supplement: Supplementary file 7 — Source data Fig. 5 [file 44321_2024_186_MOESM7_ESM.zip › Figure 5/5A/Invasion/C4-2BENZR shC.tif]

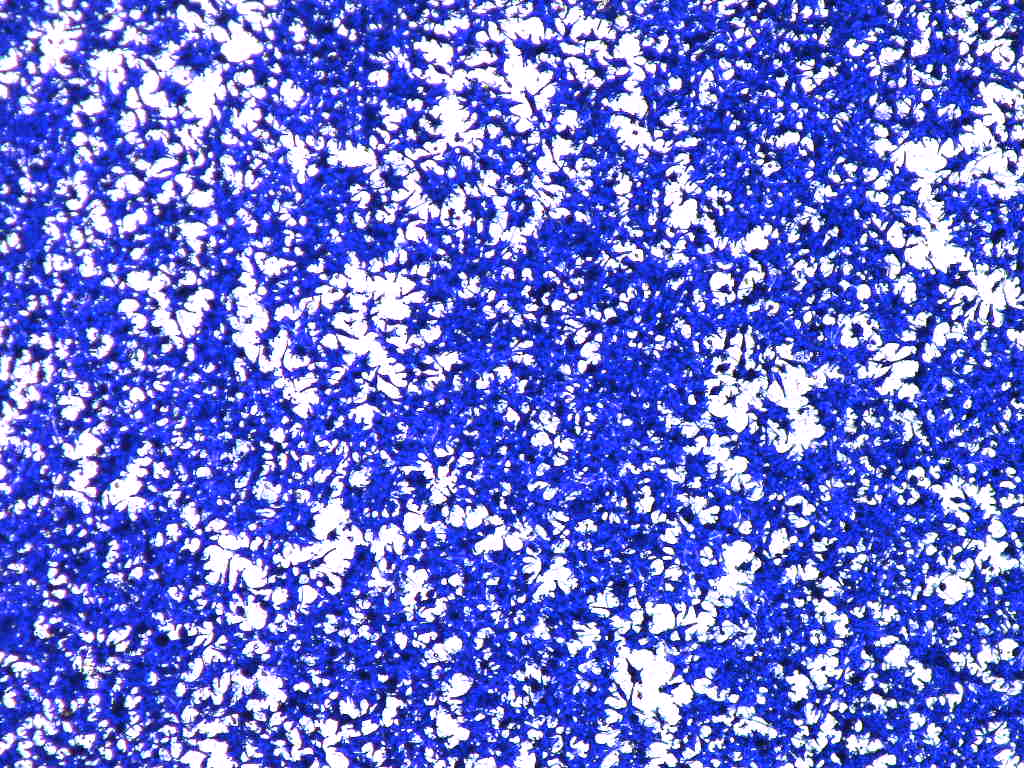

Supplement: Supplementary file 7 — Source data Fig. 5 [file 44321_2024_186_MOESM7_ESM.zip › Figure 5/5A/Migration/22Rv1 shC.tif]

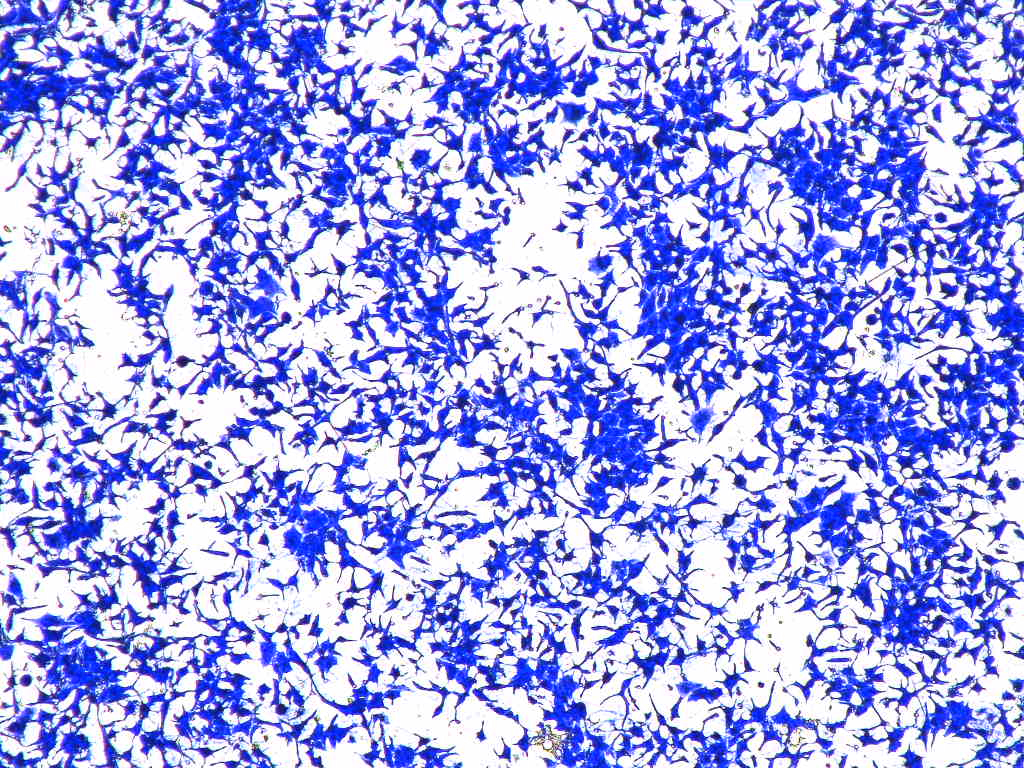

Supplement: Supplementary file 7 — Source data Fig. 5 [file 44321_2024_186_MOESM7_ESM.zip › Figure 5/5A/Migration/C4-2B ENZR shC.tif]

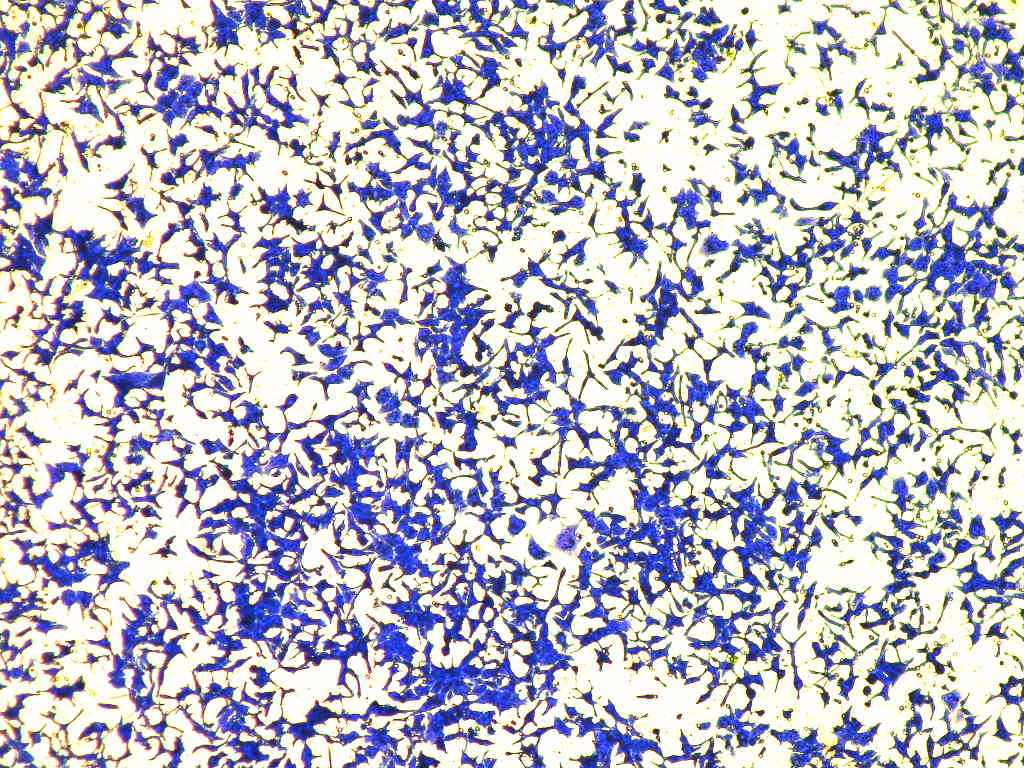

Supplement: Supplementary file 7 — Source data Fig. 5 [file 44321_2024_186_MOESM7_ESM.zip › Figure 5/5A/Migration/C4-2B ENZR shPlexinD1-2.tif]

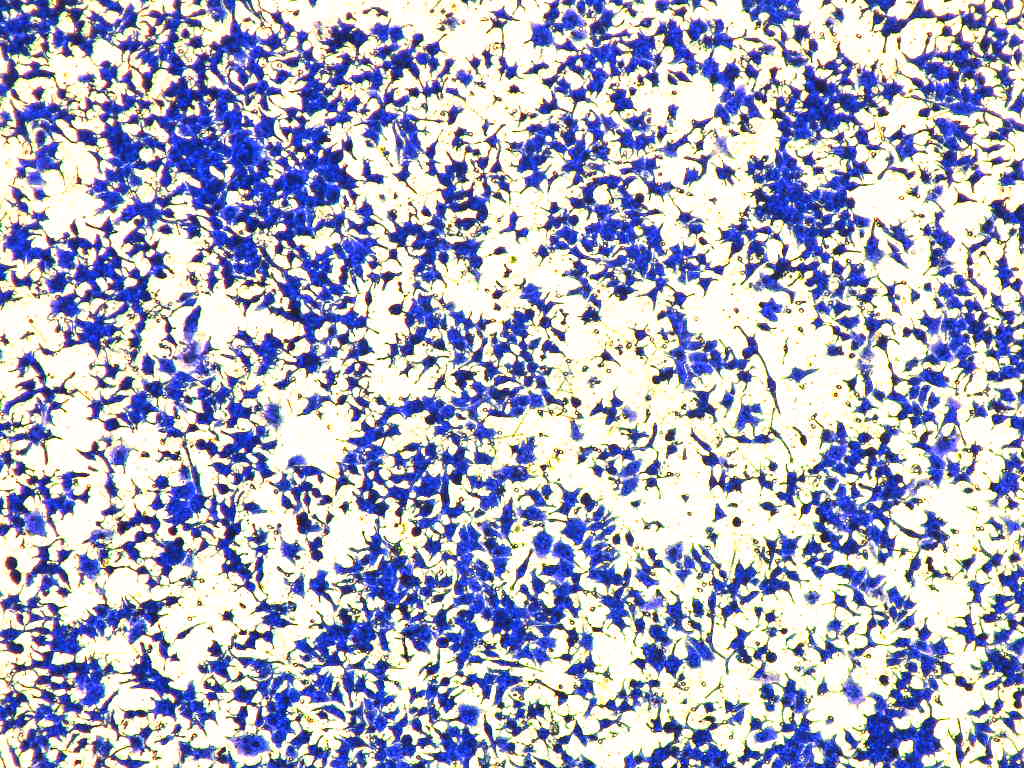

Supplement: Supplementary file 7 — Source data Fig. 5 [file 44321_2024_186_MOESM7_ESM.zip › Figure 5/5A/Migration/C4-2B ENZR shPlexinD1-1.tif]

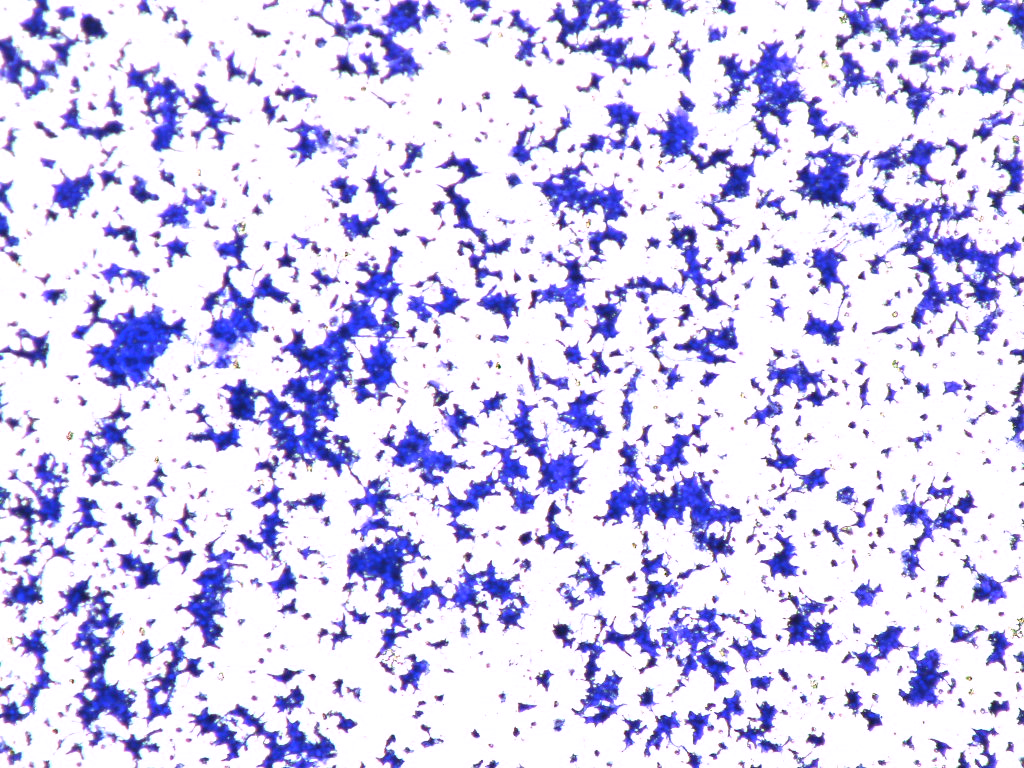

Supplement: Supplementary file 7 — Source data Fig. 5 [file 44321_2024_186_MOESM7_ESM.zip › Figure 5/5A/Migration/22Rv1 shPlexinD1-1.tif]

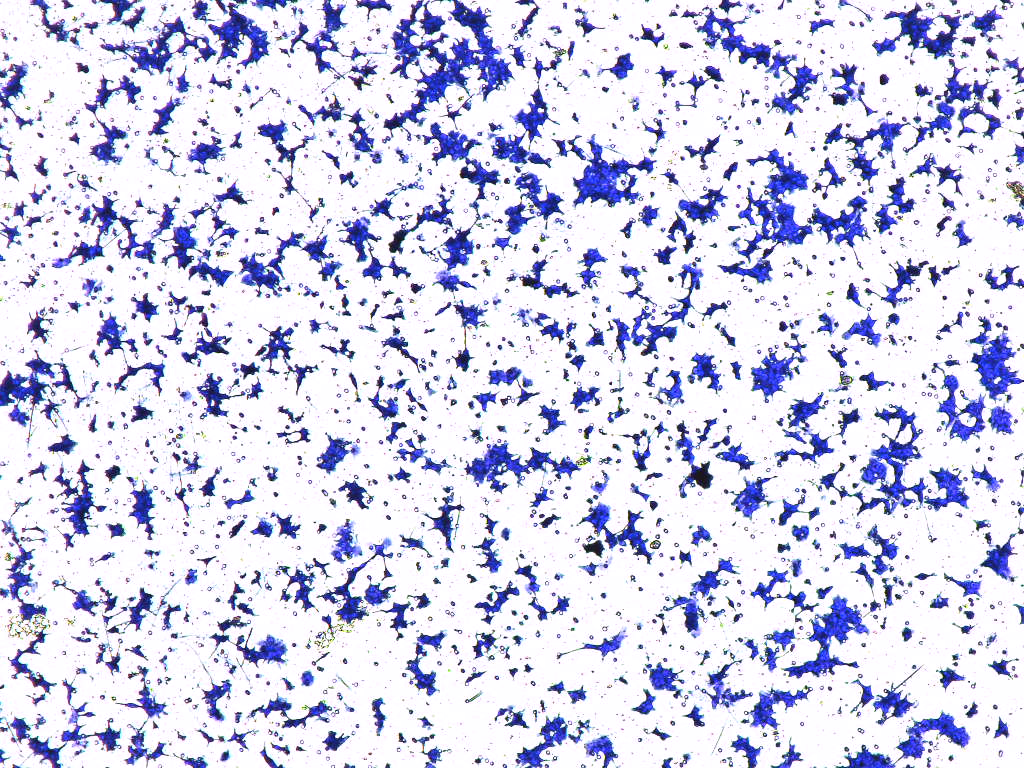

Supplement: Supplementary file 7 — Source data Fig. 5 [file 44321_2024_186_MOESM7_ESM.zip › Figure 5/5A/Migration/22Rv1 shPlexinD1-2.tif]

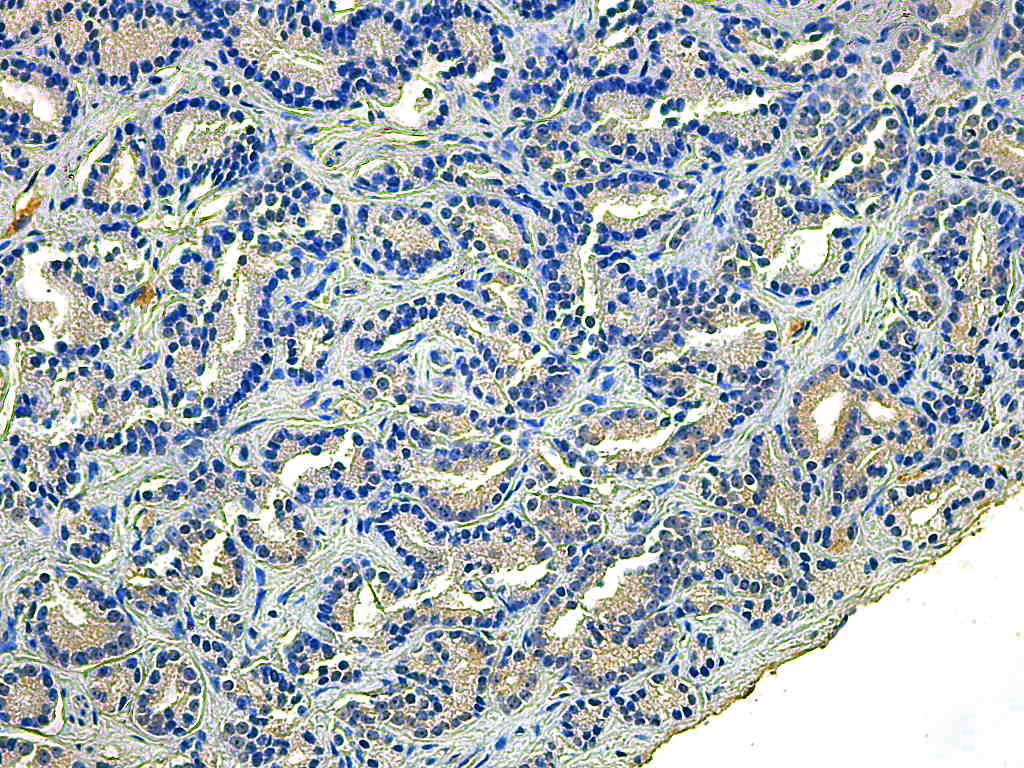

Supplement: Supplementary file 8 — Source data Fig. 6 [file 44321_2024_186_MOESM8_ESM.zip › Figure 6/6G/PlexinD1 high.tif]

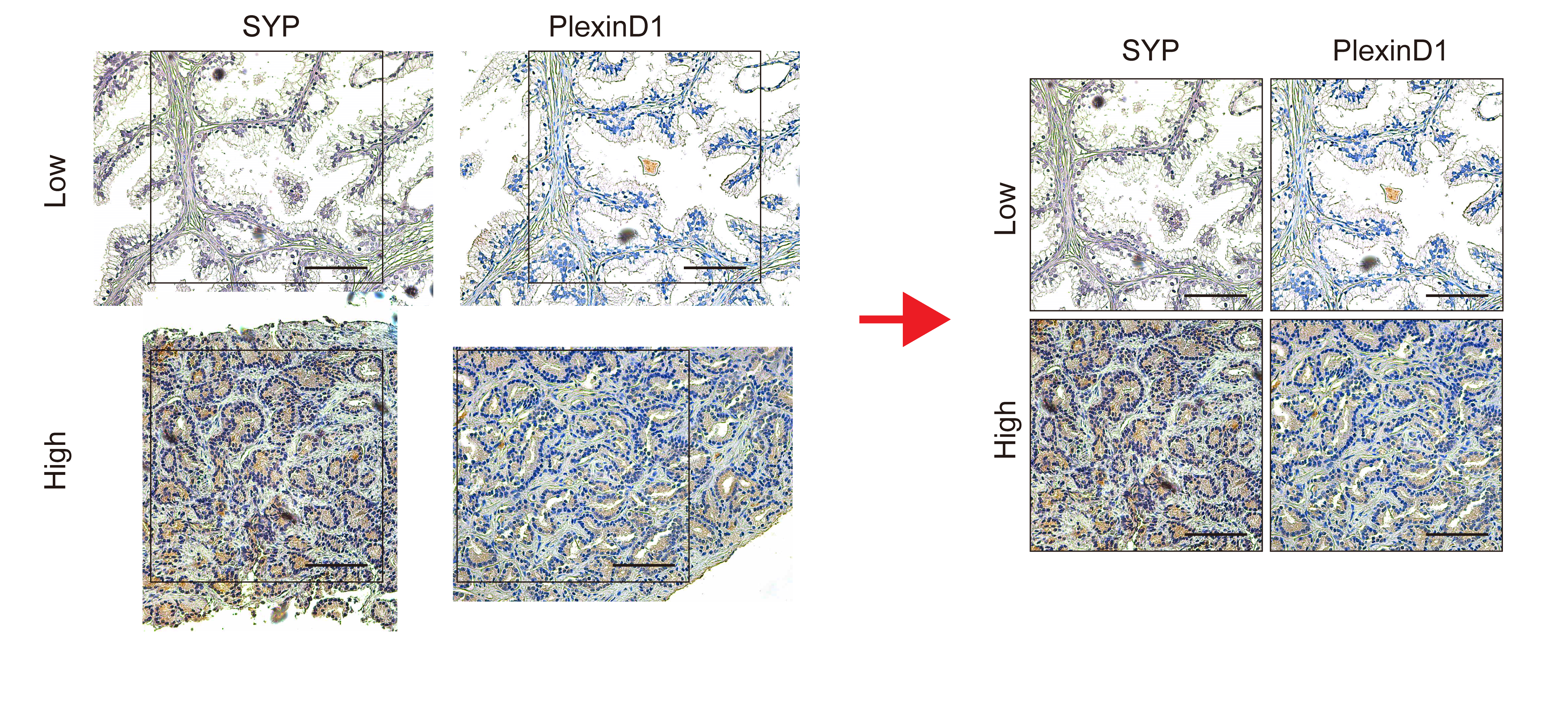

Supplement: Supplementary file 8 — Source data Fig. 6 [file 44321_2024_186_MOESM8_ESM.zip › Figure 6/6G/README.tif]

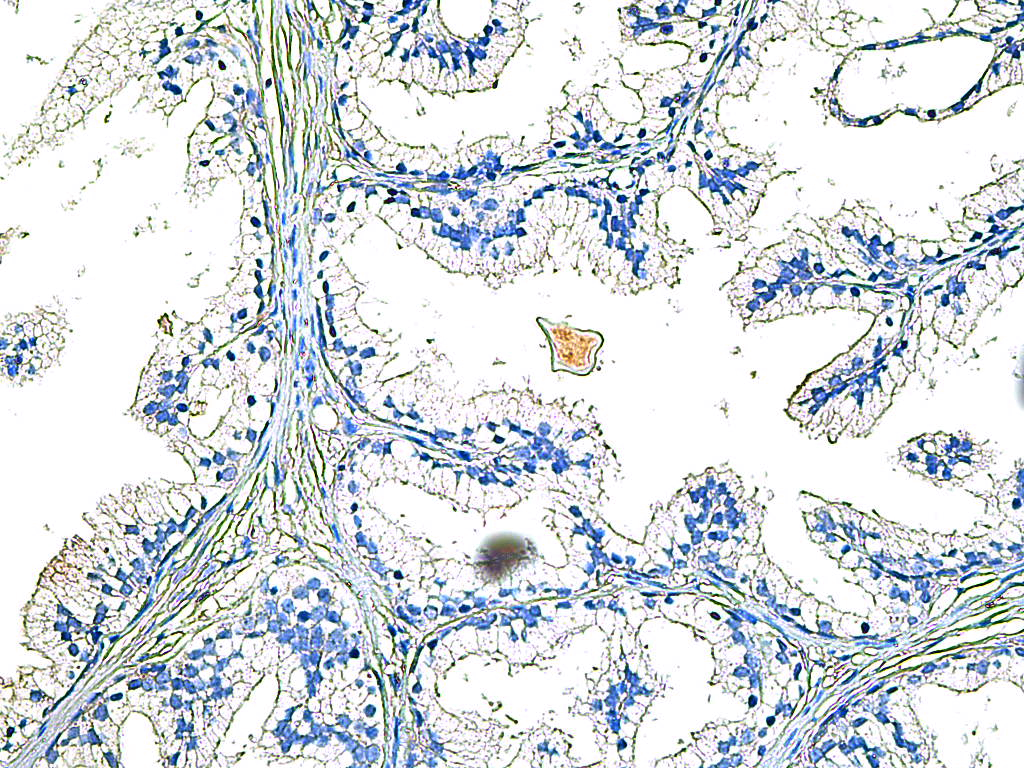

Supplement: Supplementary file 8 — Source data Fig. 6 [file 44321_2024_186_MOESM8_ESM.zip › Figure 6/6G/PlexinD1 low.tif]

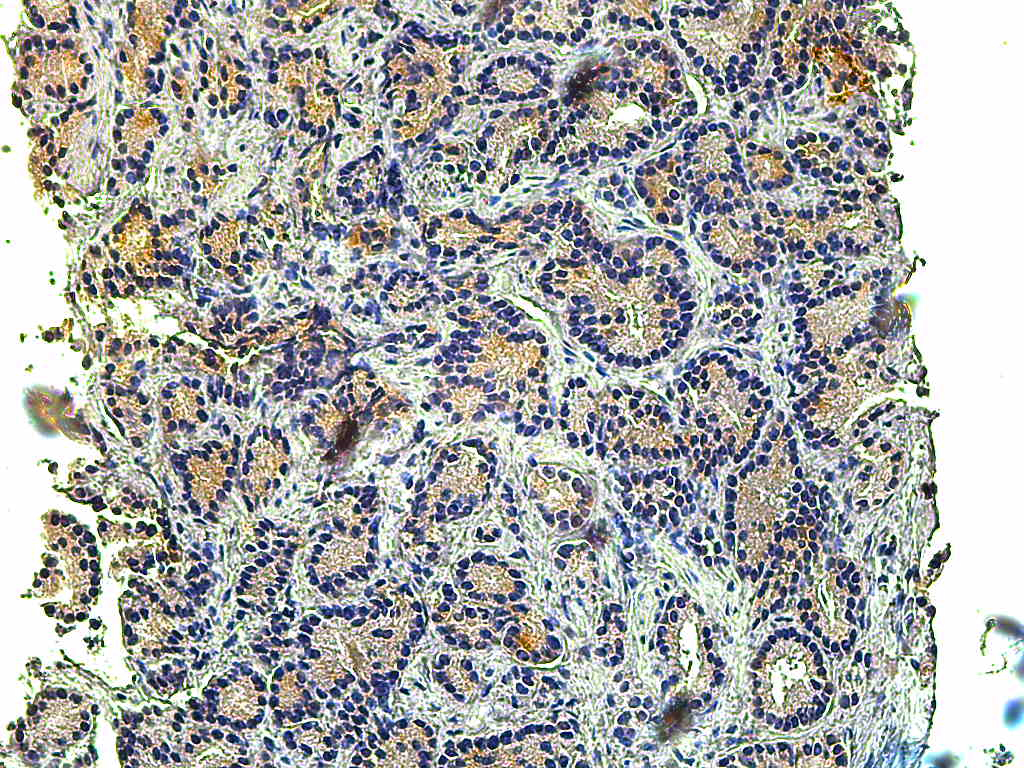

Supplement: Supplementary file 8 — Source data Fig. 6 [file 44321_2024_186_MOESM8_ESM.zip › Figure 6/6G/SYP high.tif]

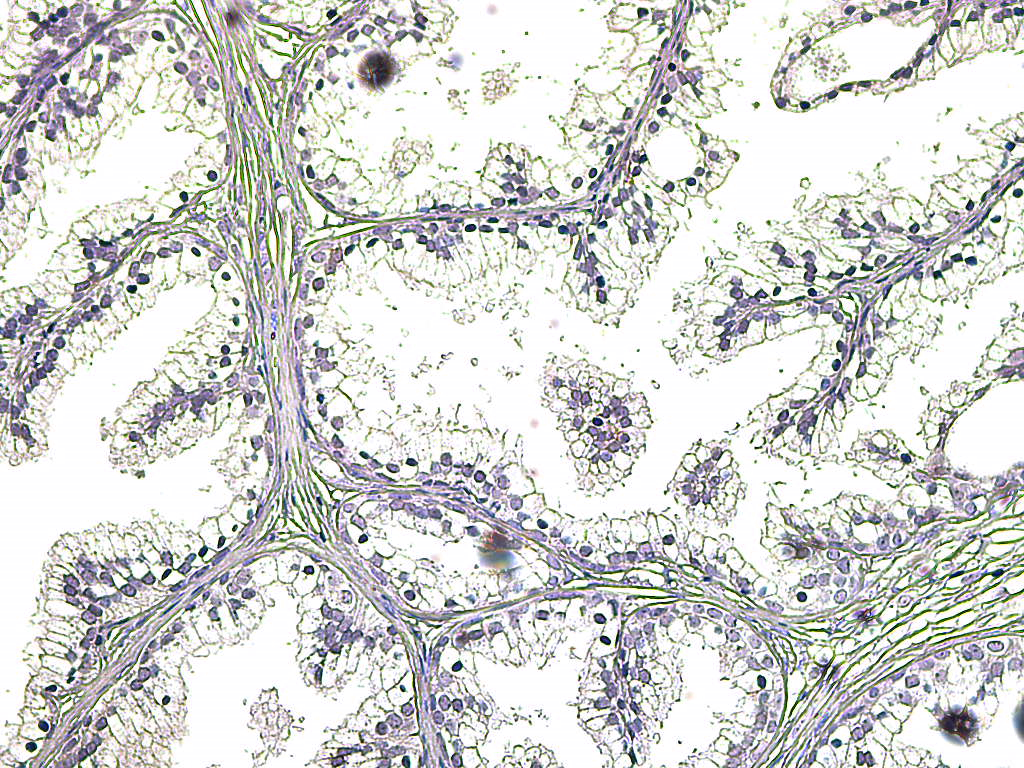

Supplement: Supplementary file 8 — Source data Fig. 6 [file 44321_2024_186_MOESM8_ESM.zip › Figure 6/6G/SYP low.tif]

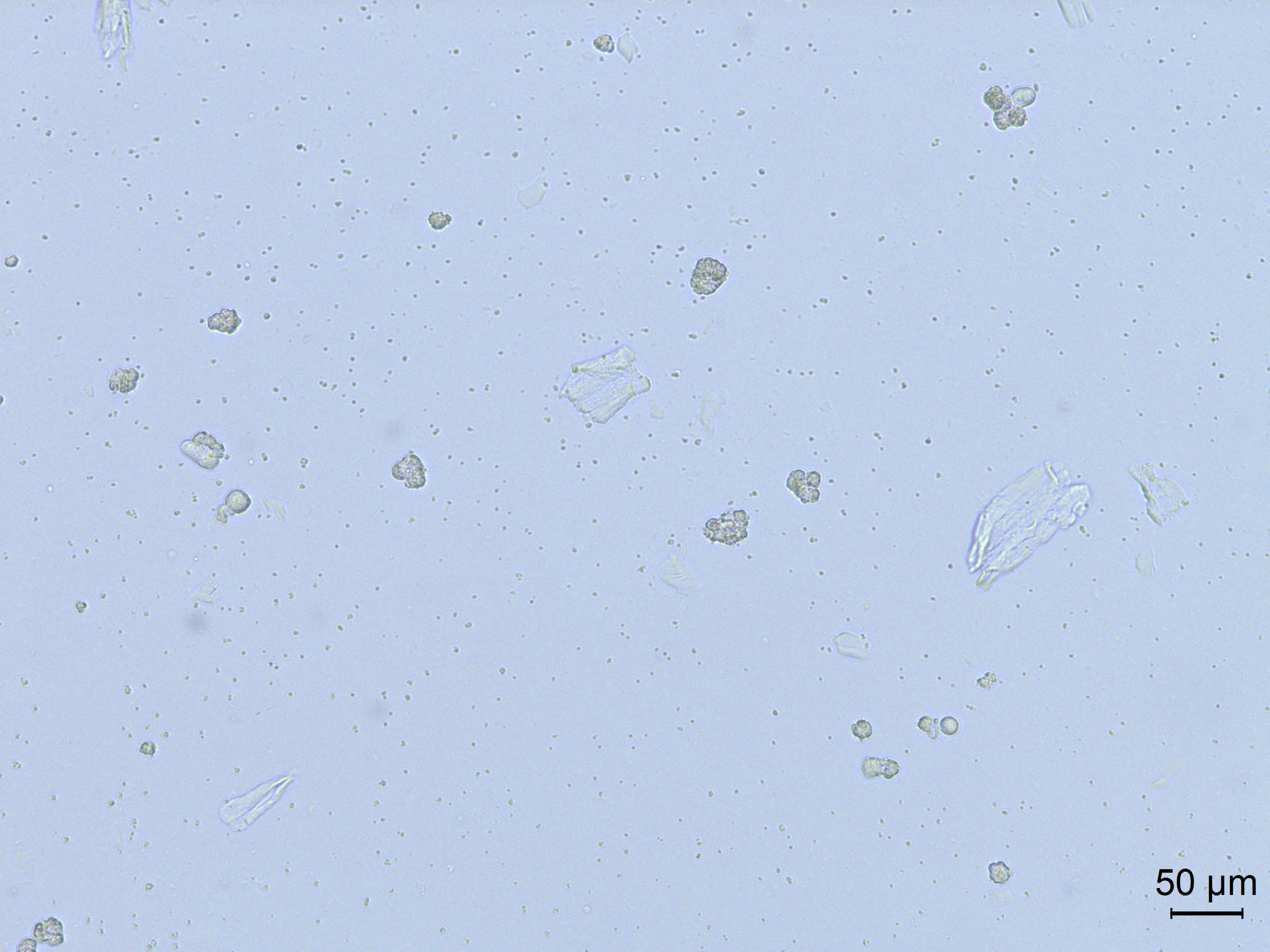

Supplement: Supplementary file 8 — Source data Fig. 6 [file 44321_2024_186_MOESM8_ESM.zip › Figure 6/6B/22Rv1_shPlexinD1-2.tif]

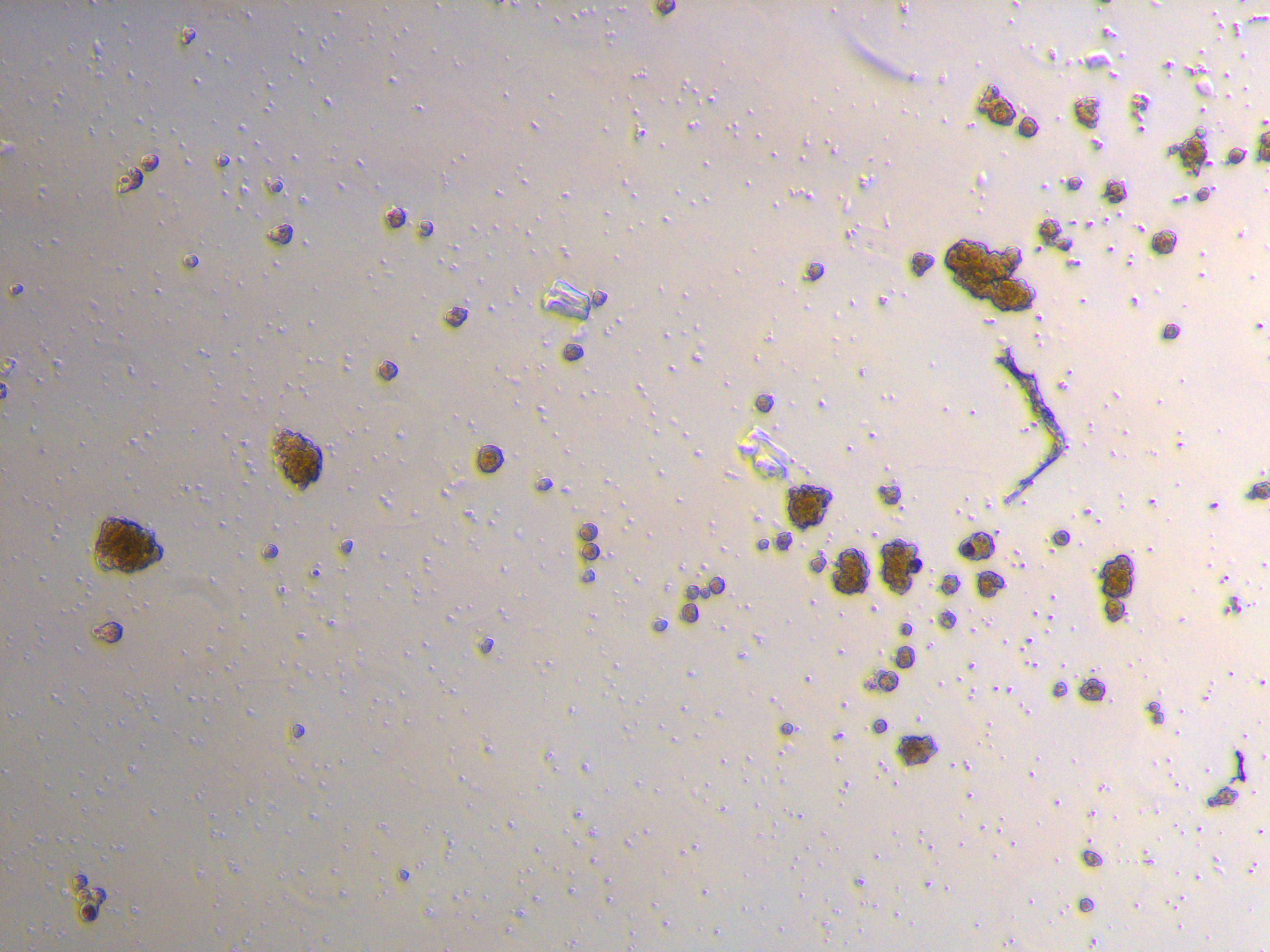

Supplement: Supplementary file 8 — Source data Fig. 6 [file 44321_2024_186_MOESM8_ESM.zip › Figure 6/6B/C4-2BENZR_shCon.tif]

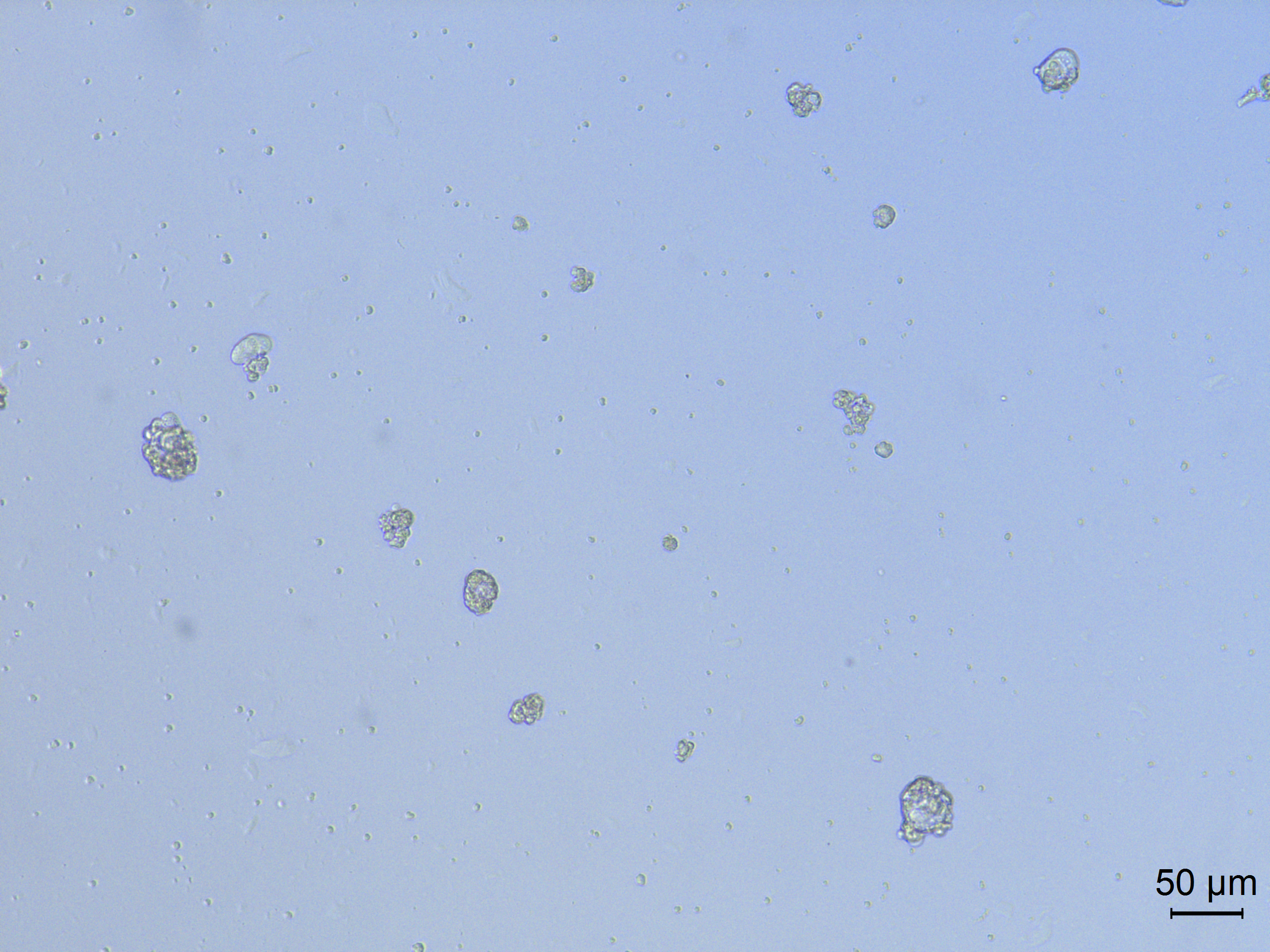

Supplement: Supplementary file 8 — Source data Fig. 6 [file 44321_2024_186_MOESM8_ESM.zip › Figure 6/6B/22Rv1_shPlexinD1-1.tif]

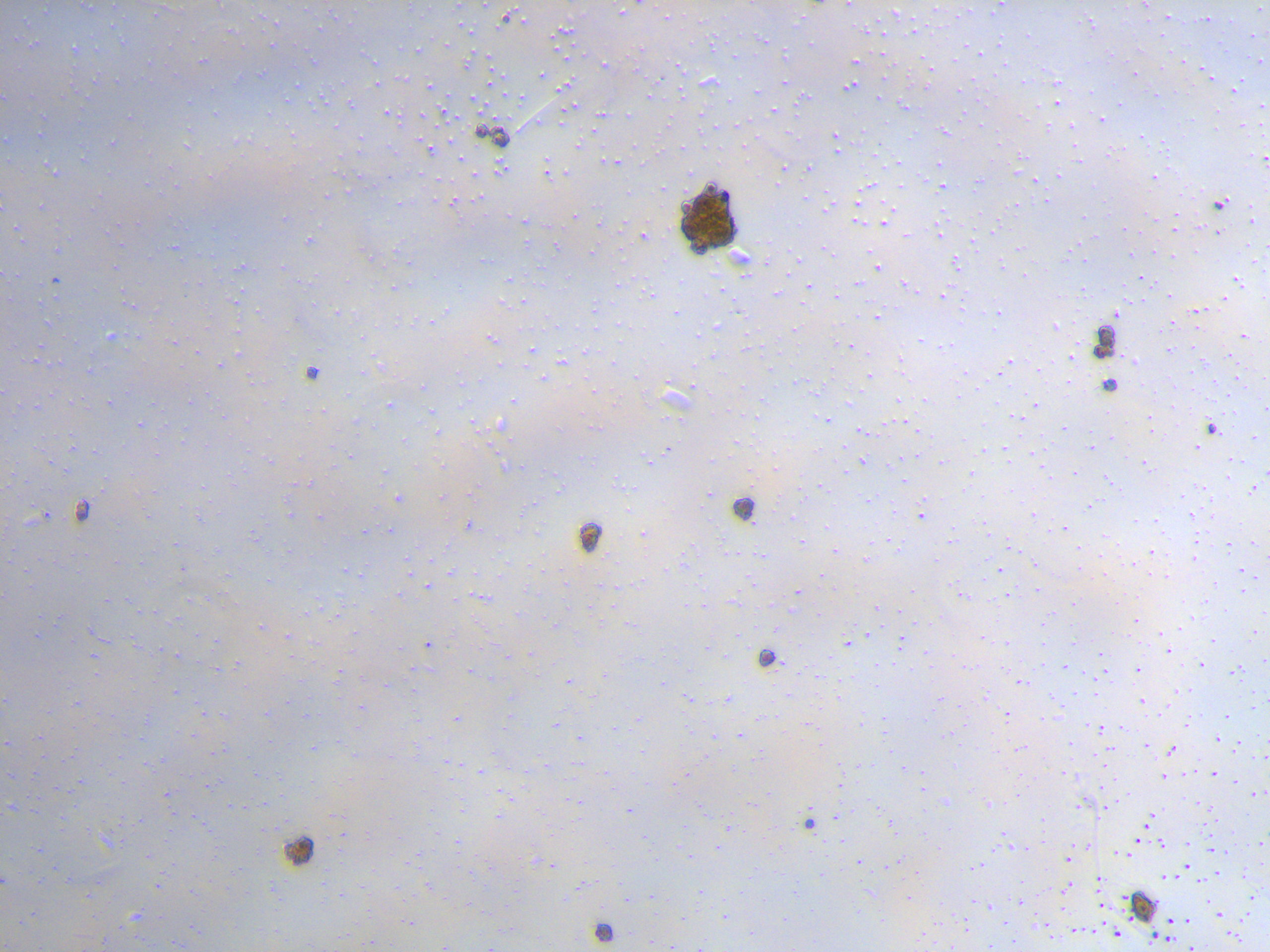

Supplement: Supplementary file 8 — Source data Fig. 6 [file 44321_2024_186_MOESM8_ESM.zip › Figure 6/6B/C4-2BENZR_shPlexinD1-2.tif]

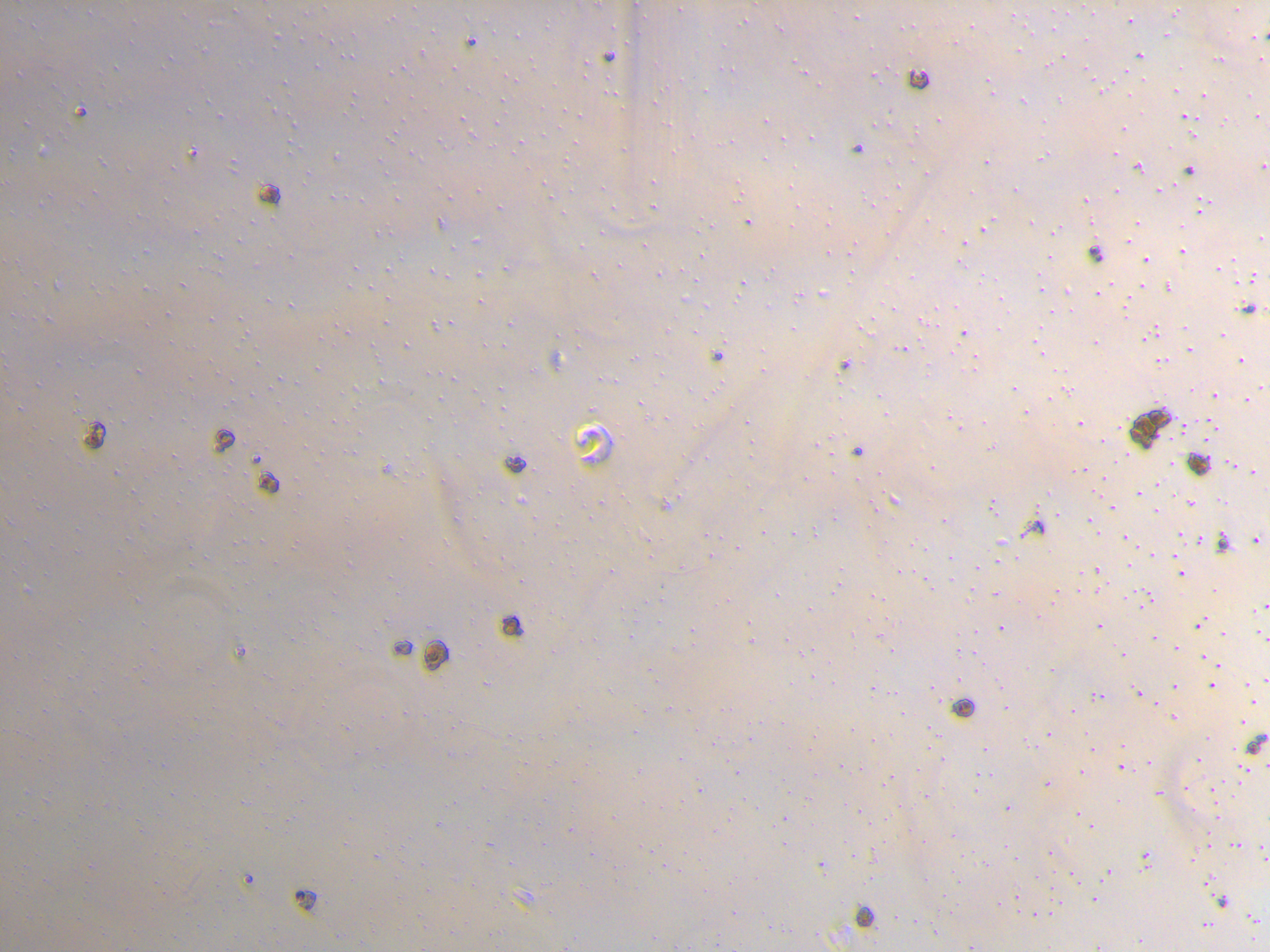

Supplement: Supplementary file 8 — Source data Fig. 6 [file 44321_2024_186_MOESM8_ESM.zip › Figure 6/6B/C4-2BENZR_shPlexinD1-1.tif]

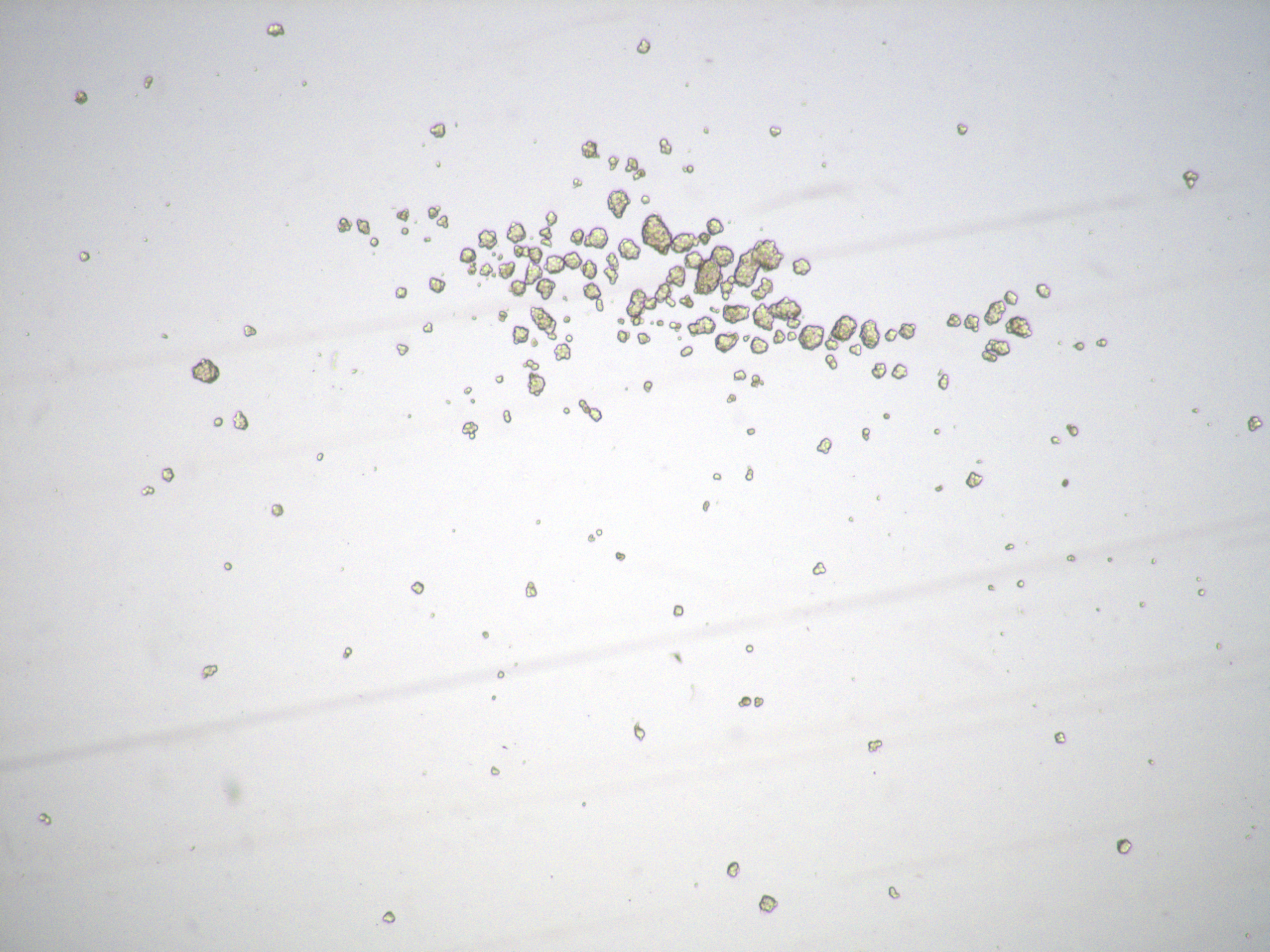

Supplement: Supplementary file 8 — Source data Fig. 6 [file 44321_2024_186_MOESM8_ESM.zip › Figure 6/6B/LNCaP_PlexinD1.tif]
