# Supplementary figures and images for: PlexinD1 is a driver and a therapeutic target in advanced prostate cancer (part 2 of 2)
Source: EMBO Mol Med. 2025 Jan 2;17(2):336–64. doi: 10.1038/s44321-024-00186-z (PMC11822115; doi:10.1038/s44321-024-00186-z)

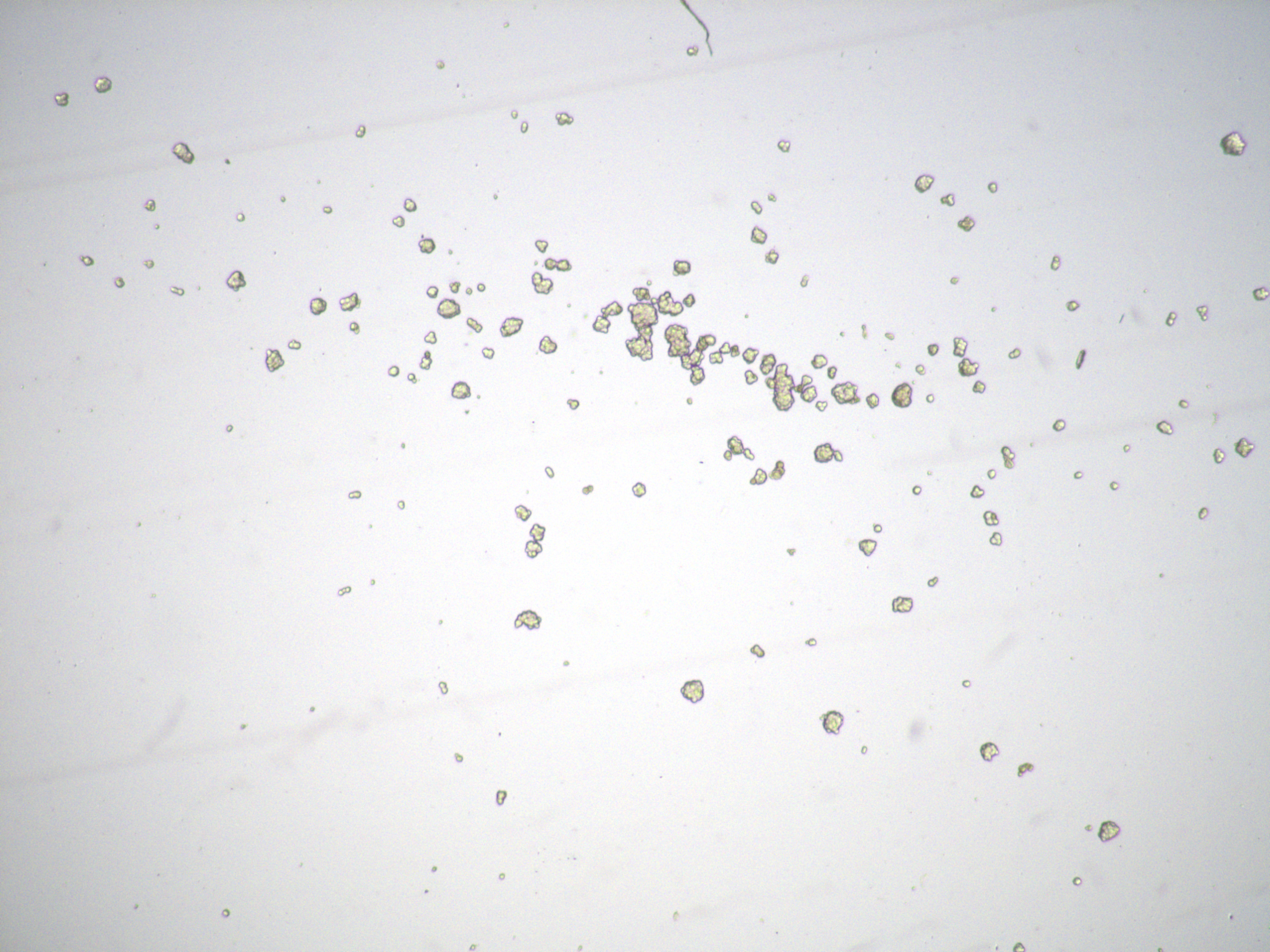

Supplement: Supplementary file 8 — Source data Fig. 6 [file 44321_2024_186_MOESM8_ESM.zip › Figure 6/6B/LNCaP_Vector.tif]

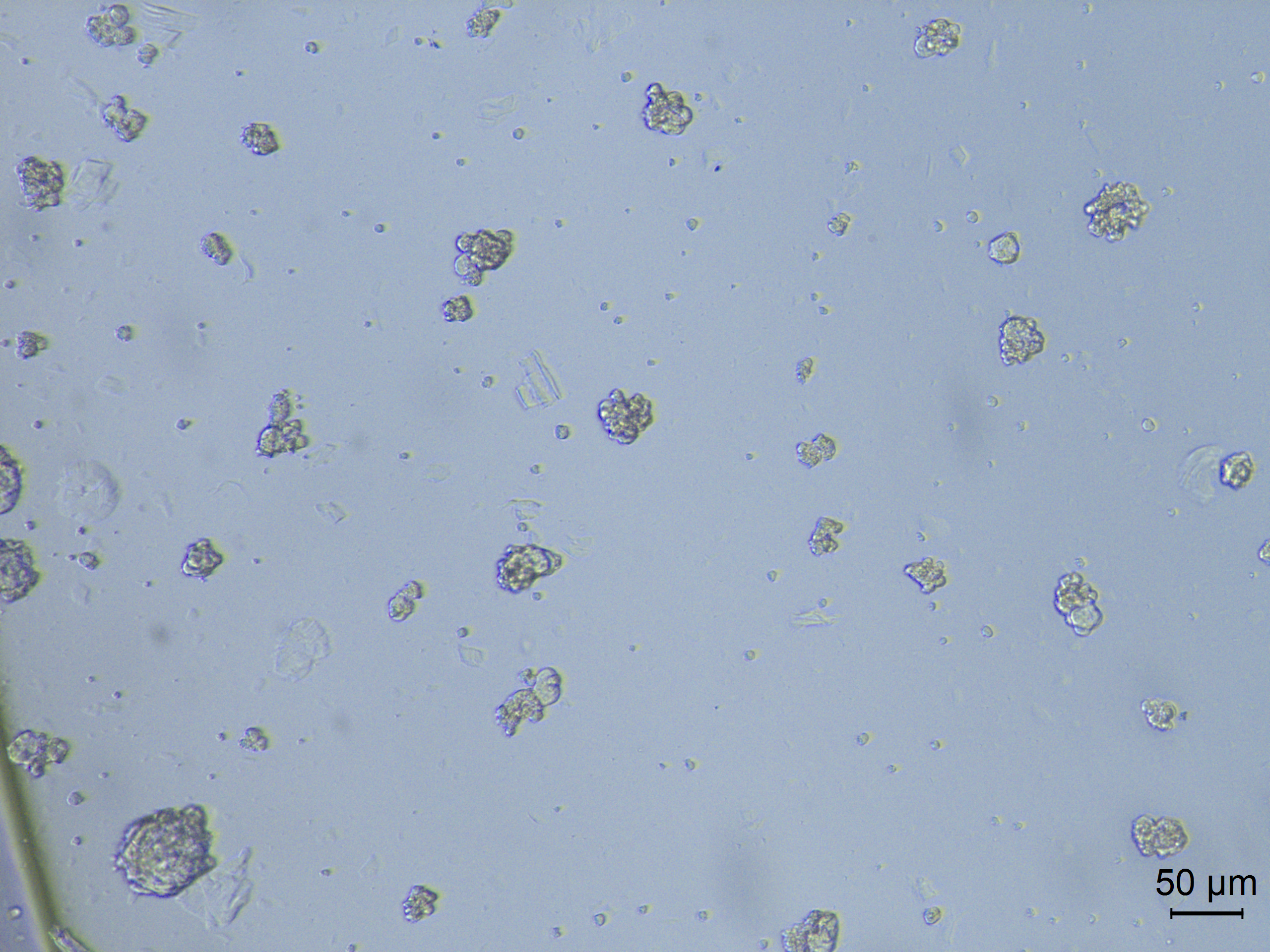

Supplement: Supplementary file 8 — Source data Fig. 6 [file 44321_2024_186_MOESM8_ESM.zip › Figure 6/6B/22Rv1_shCon.tif]

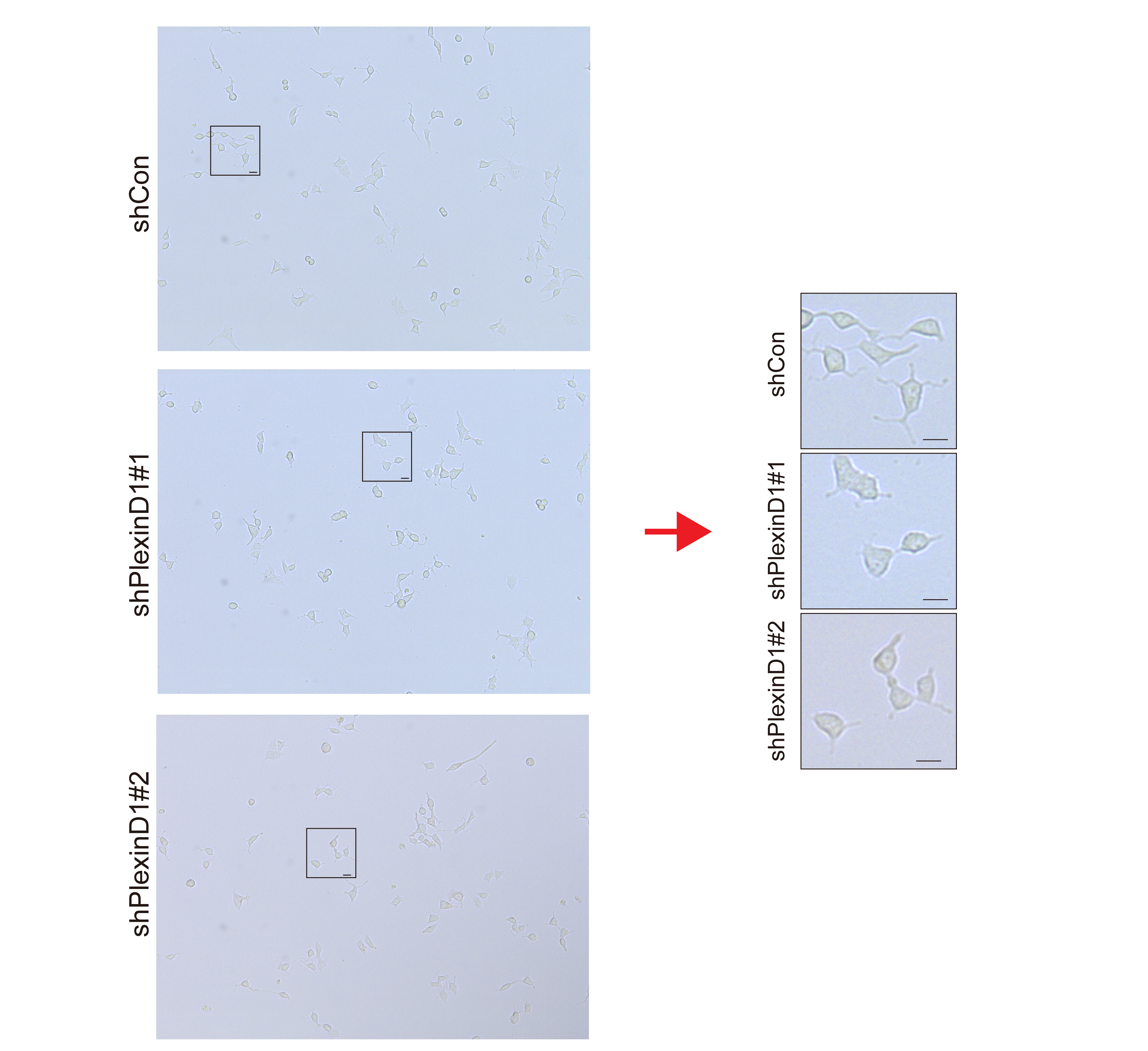

Supplement: Supplementary file 8 — Source data Fig. 6 [file 44321_2024_186_MOESM8_ESM.zip › Figure 6/6E/README.tif]

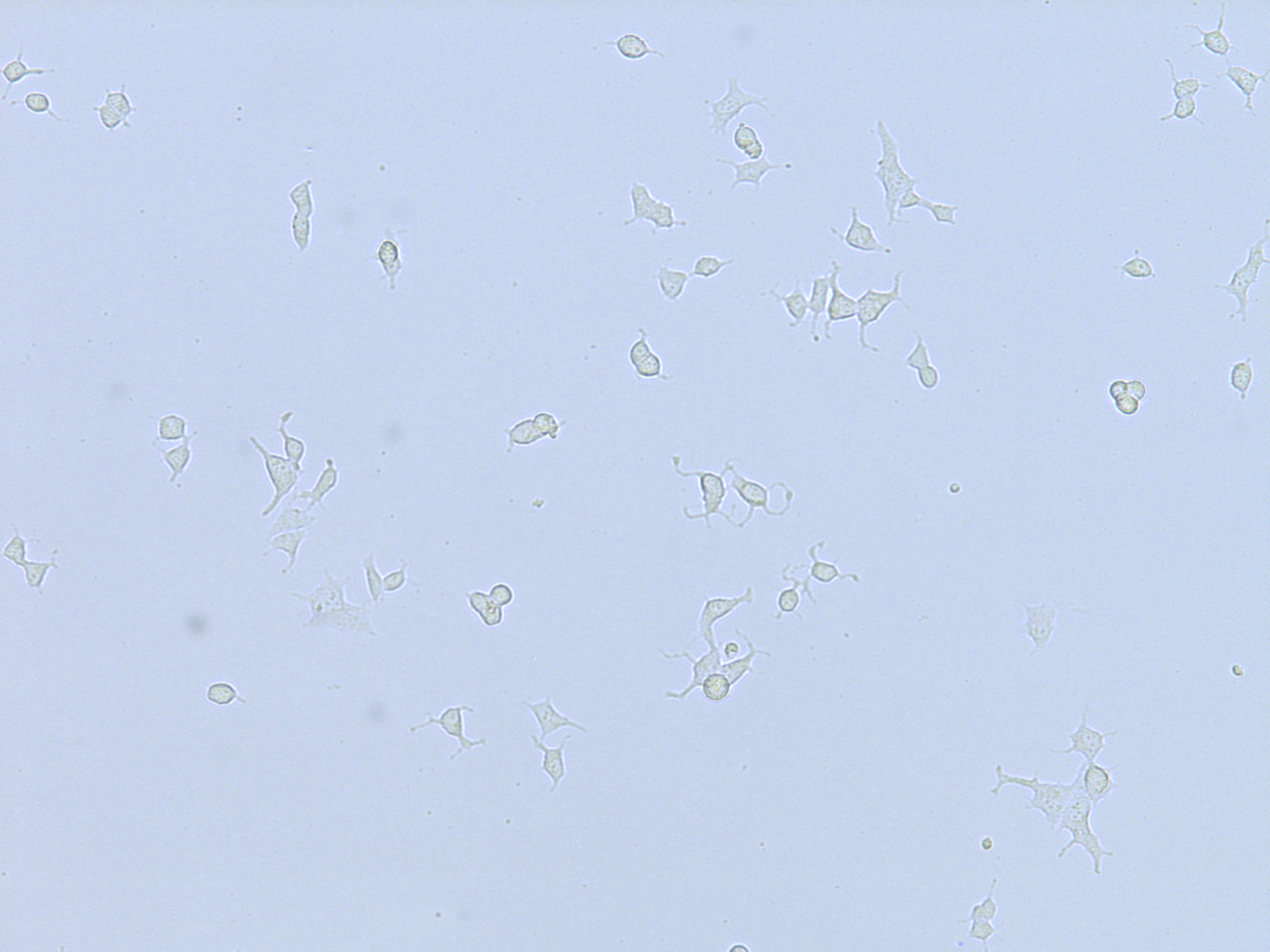

Supplement: Supplementary file 8 — Source data Fig. 6 [file 44321_2024_186_MOESM8_ESM.zip › Figure 6/6E/shPlexinD1-1.tif]

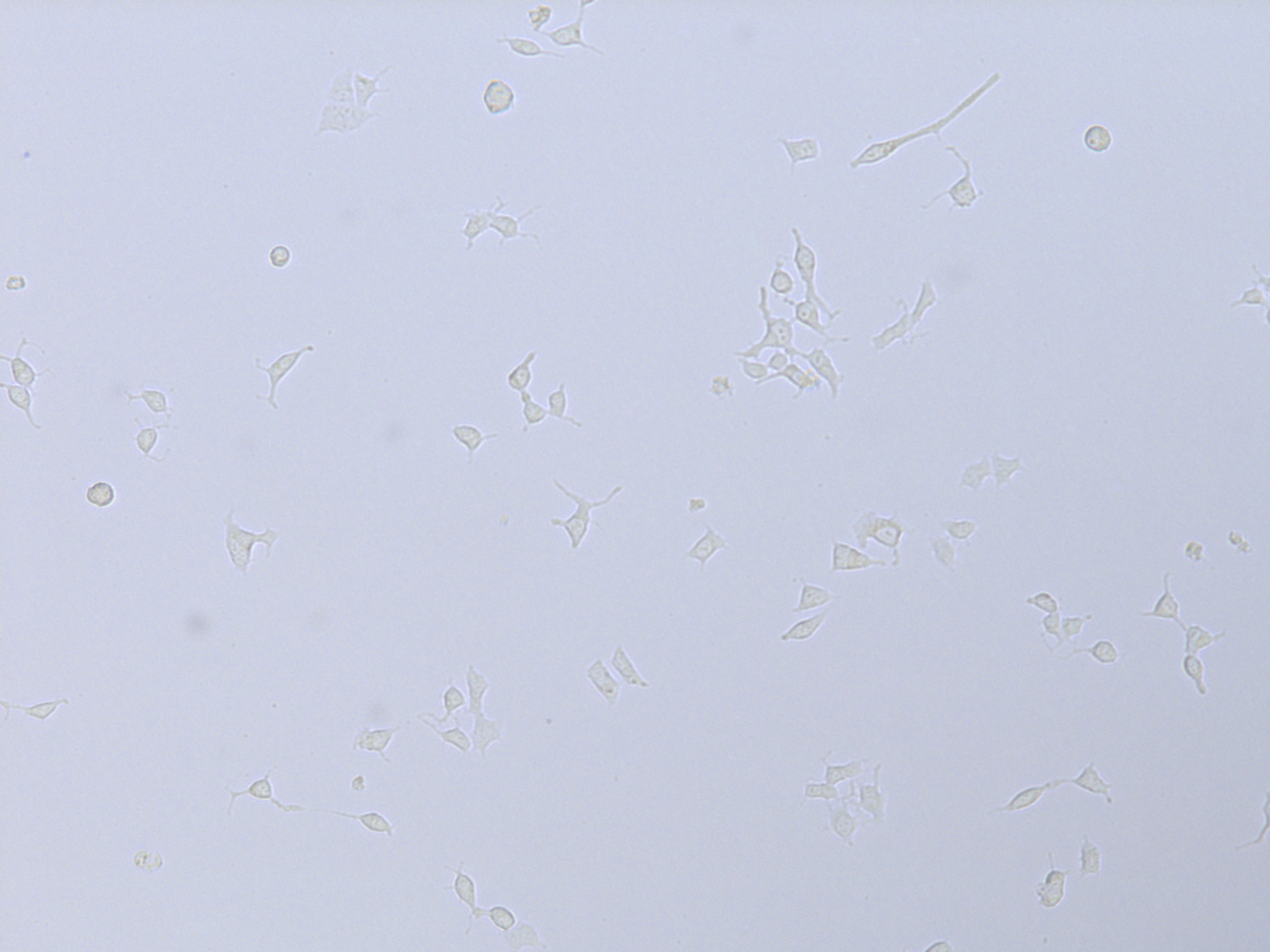

Supplement: Supplementary file 8 — Source data Fig. 6 [file 44321_2024_186_MOESM8_ESM.zip › Figure 6/6E/shPlexinD1-2.tif]

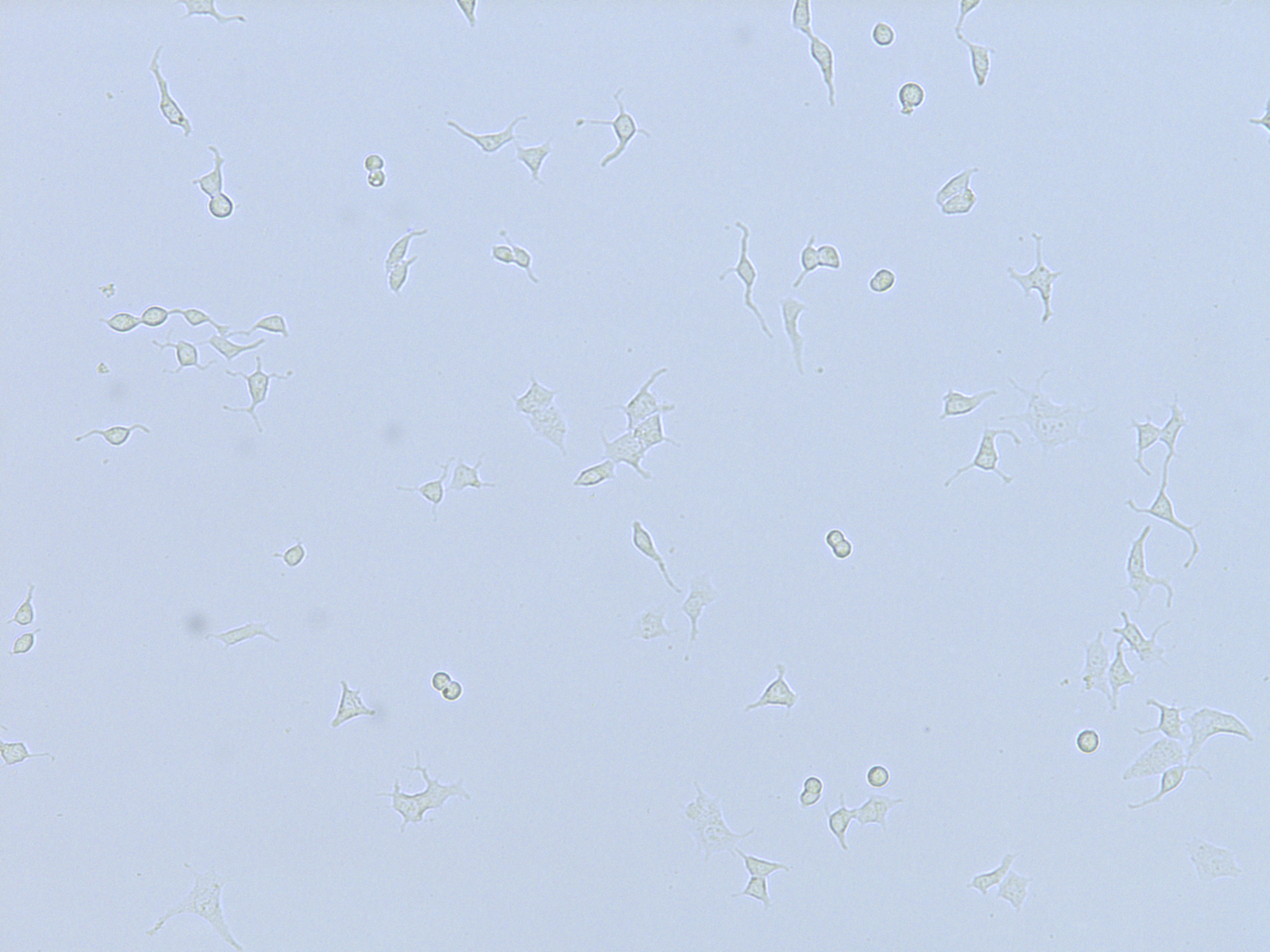

Supplement: Supplementary file 8 — Source data Fig. 6 [file 44321_2024_186_MOESM8_ESM.zip › Figure 6/6E/shC.tif]

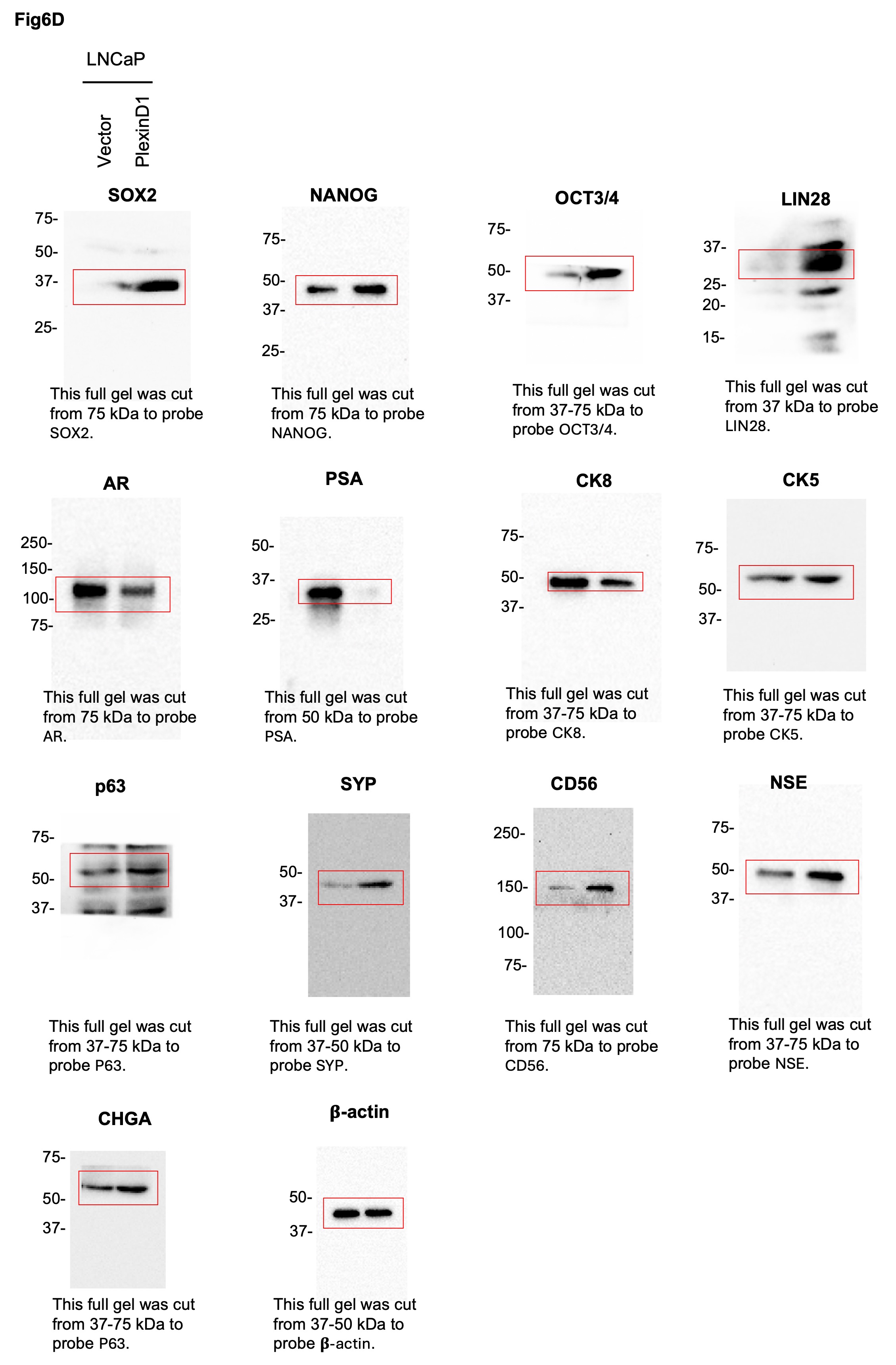

Supplement: Supplementary file 8 — Source data Fig. 6 [file 44321_2024_186_MOESM8_ESM.zip › Figure 6/6D/WB-6D-LNCaP.jpg]

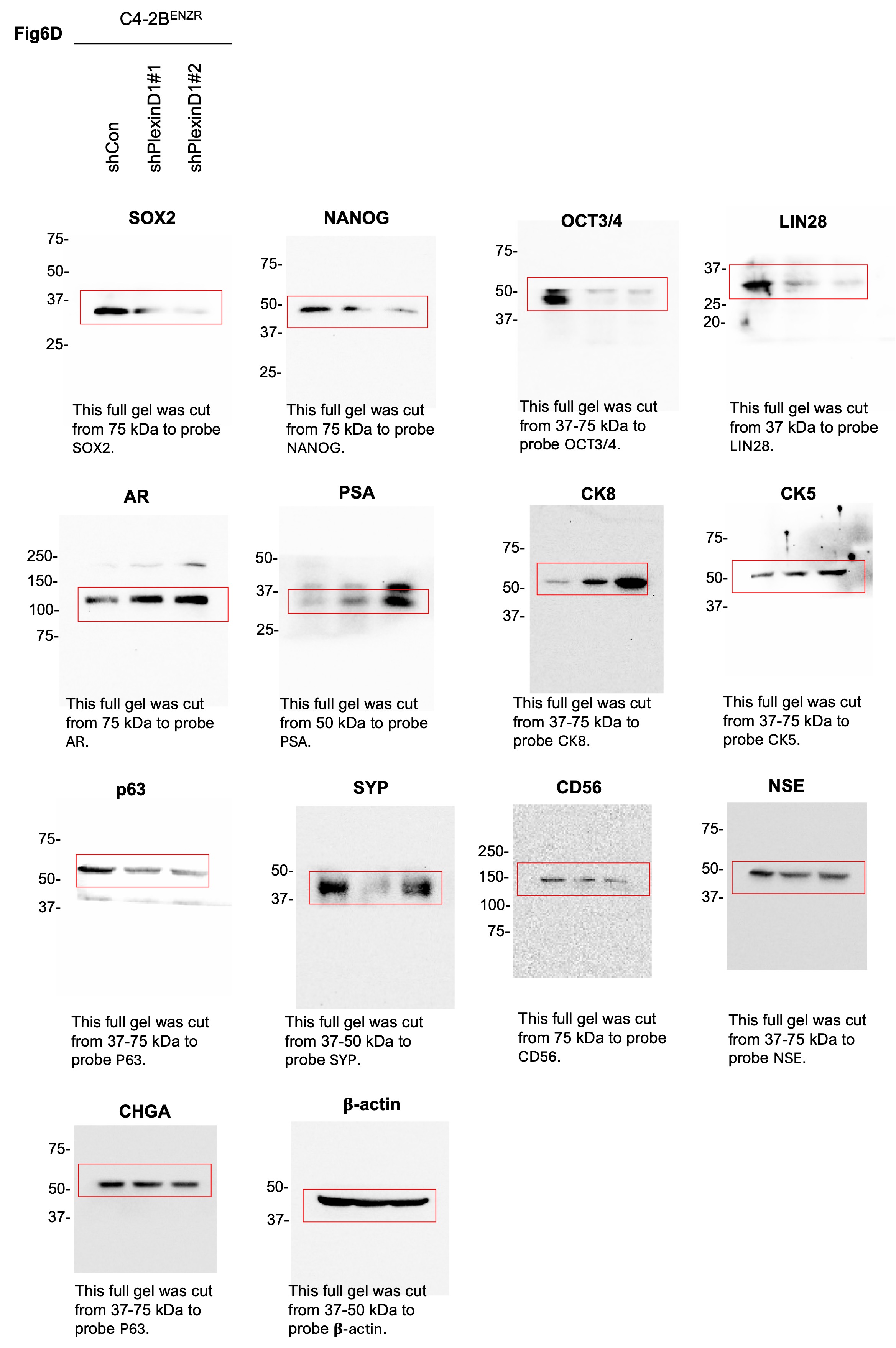

Supplement: Supplementary file 8 — Source data Fig. 6 [file 44321_2024_186_MOESM8_ESM.zip › Figure 6/6D/WB-6D C4-2BENZR.jpg]

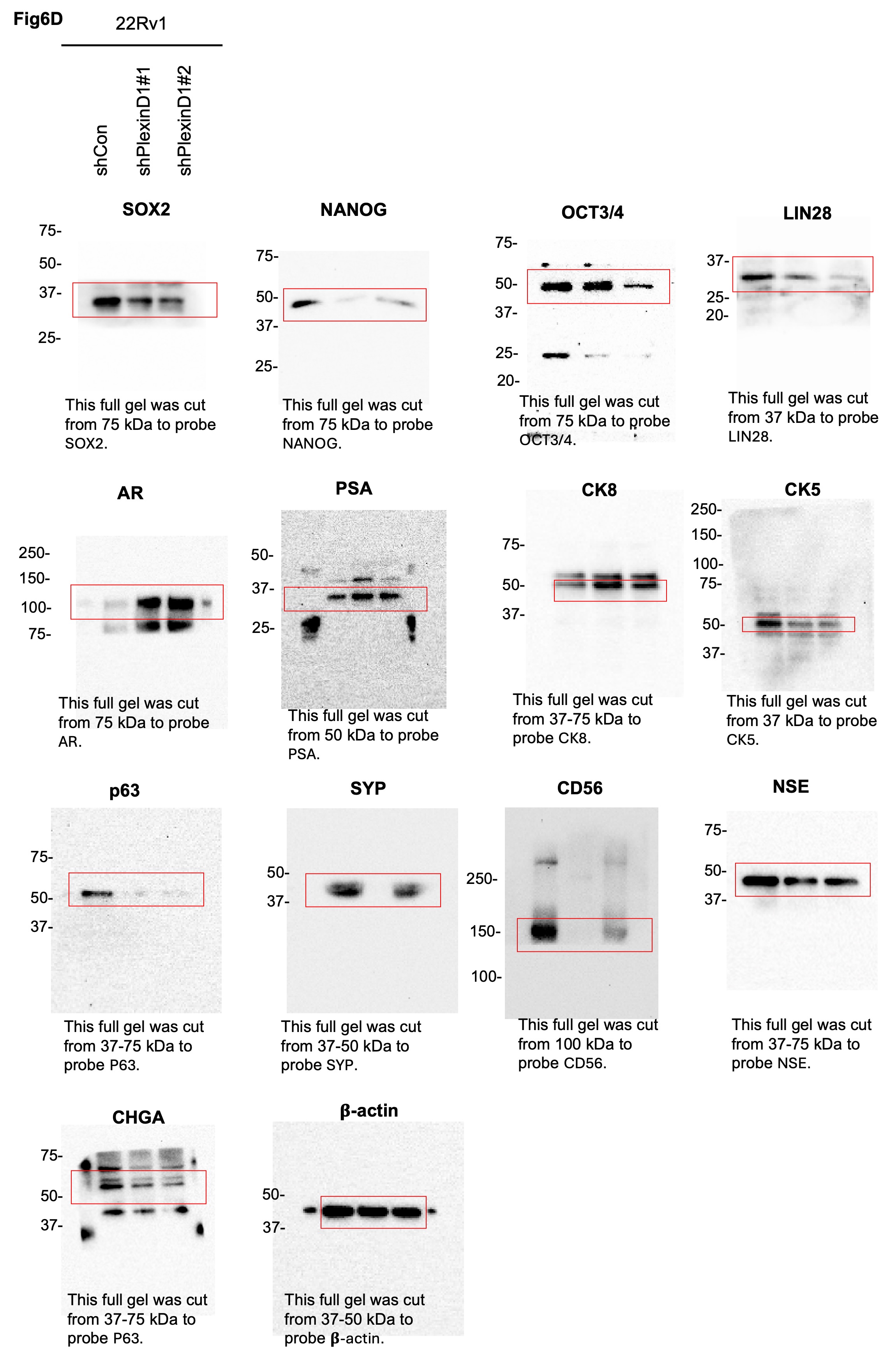

Supplement: Supplementary file 8 — Source data Fig. 6 [file 44321_2024_186_MOESM8_ESM.zip › Figure 6/6D/WB-6D 22Rv1.jpg]

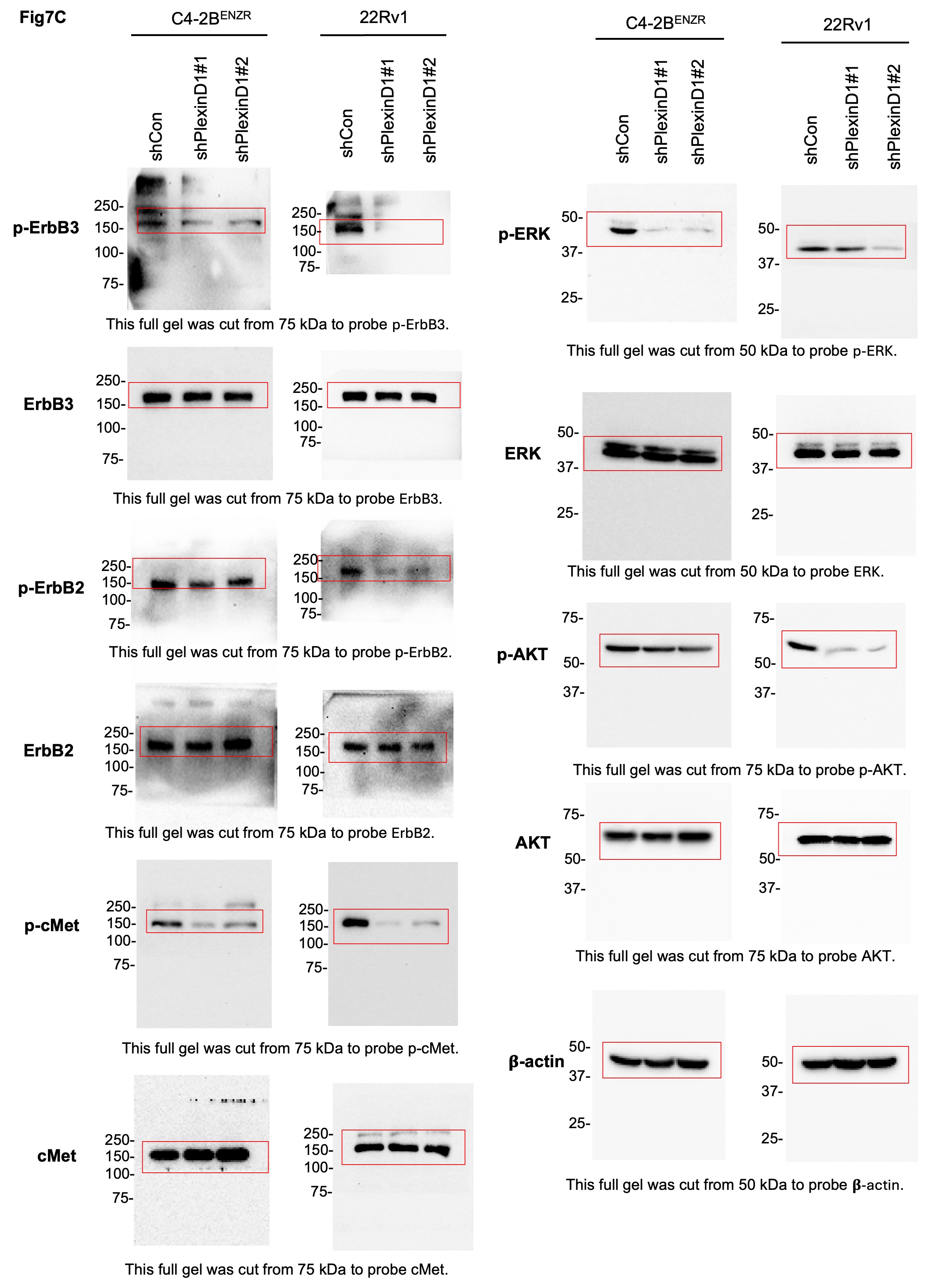

Supplement: Supplementary file 9 — Source data Fig. 7 [file 44321_2024_186_MOESM9_ESM.zip › Figure 7/7C/WB-7C.jpg]

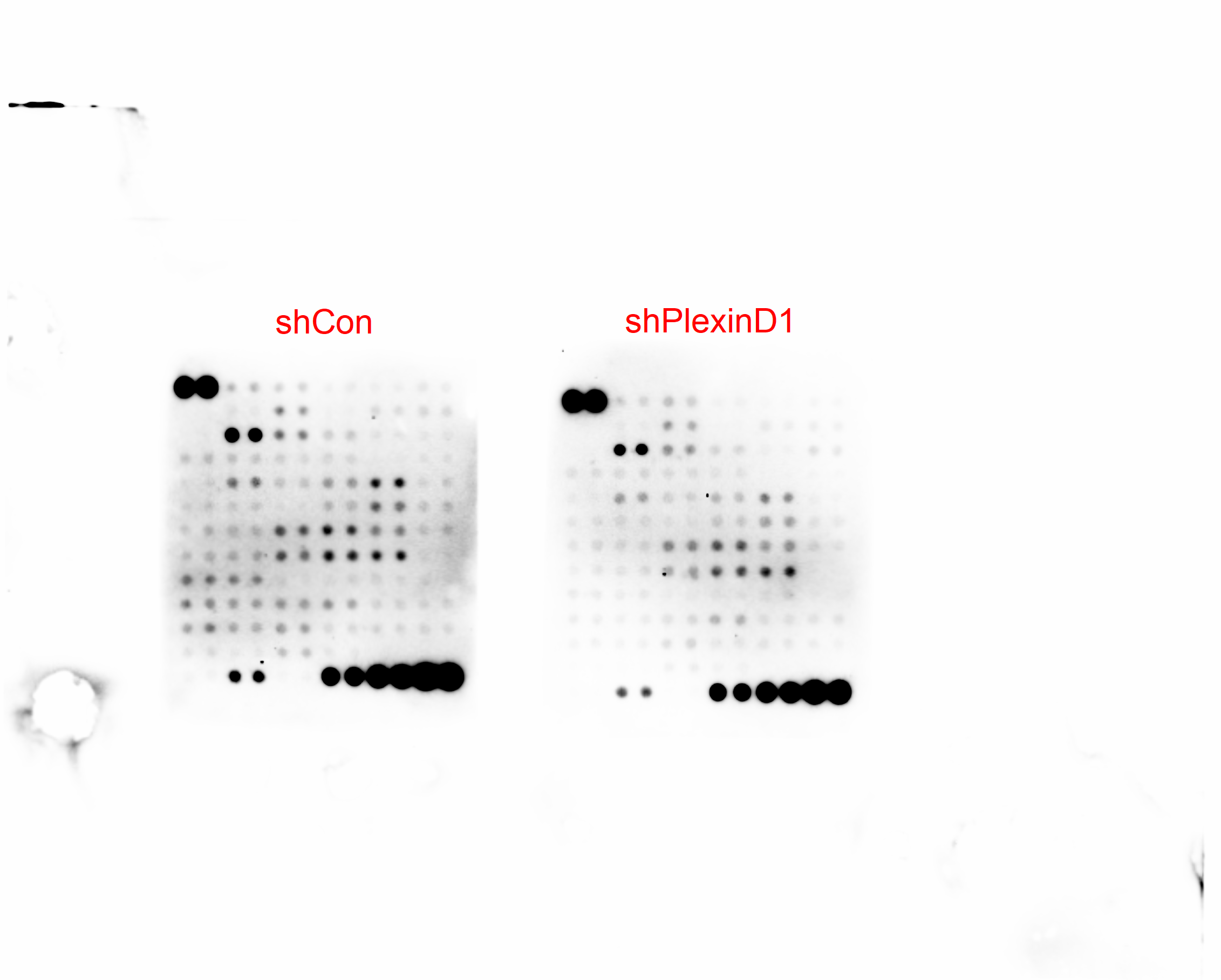

Supplement: Supplementary file 9 — Source data Fig. 7 [file 44321_2024_186_MOESM9_ESM.zip › Figure 7/7B/RTK array.tif]

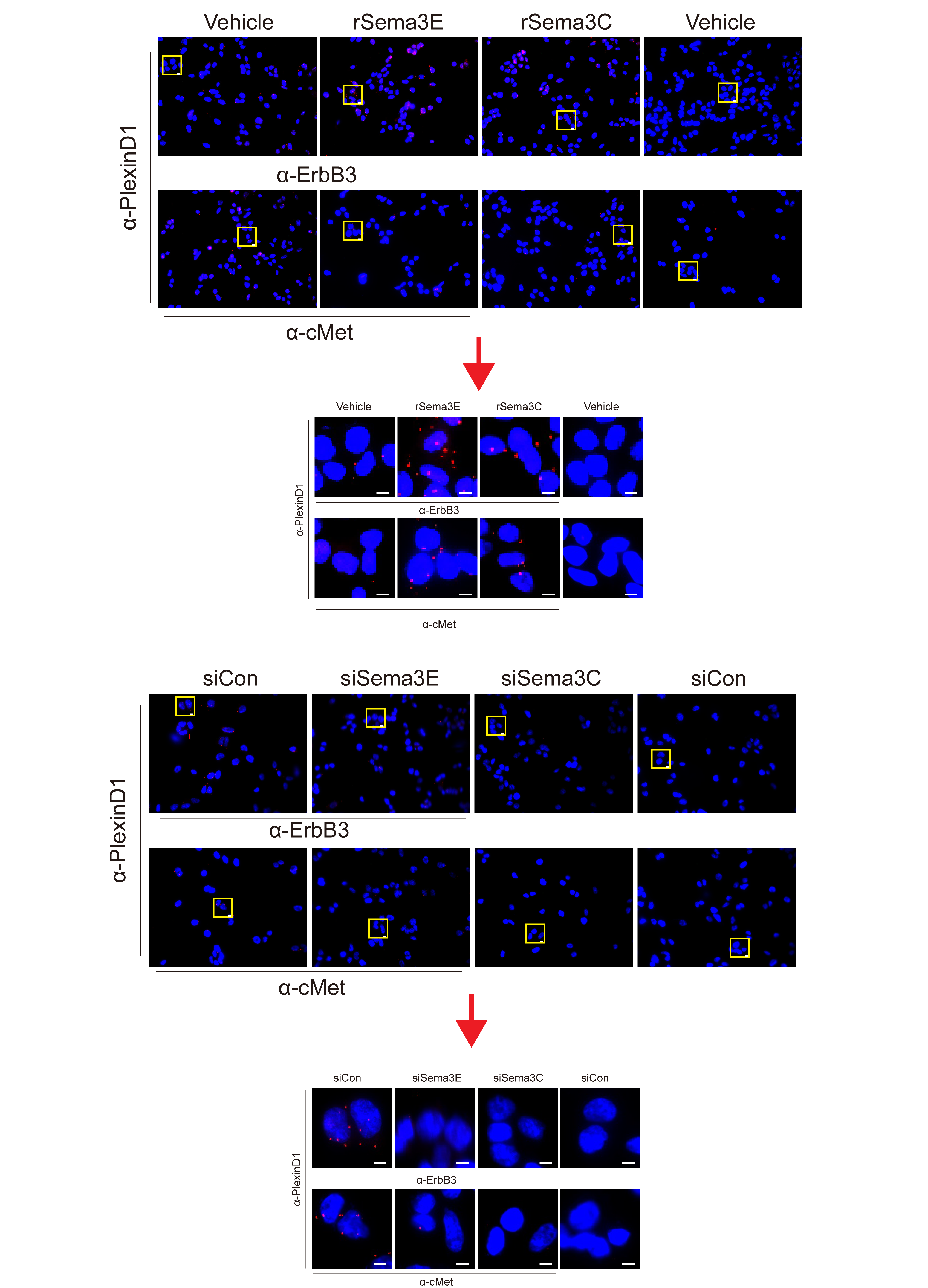

Supplement: Supplementary file 9 — Source data Fig. 7 [file 44321_2024_186_MOESM9_ESM.zip › Figure 7/7E/README.tif]

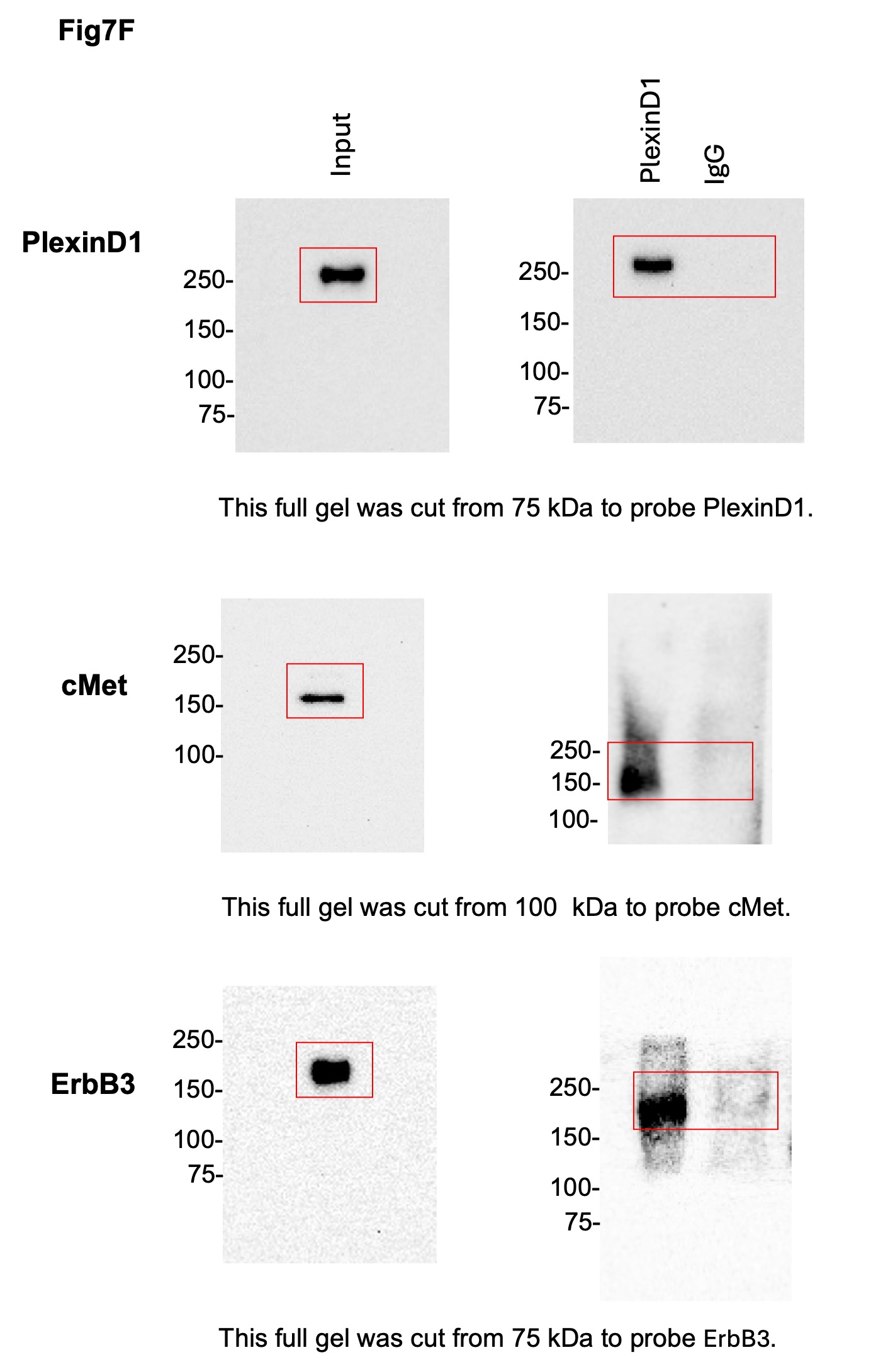

Supplement: Supplementary file 9 — Source data Fig. 7 [file 44321_2024_186_MOESM9_ESM.zip › Figure 7/7F/WB-7F.jpg]

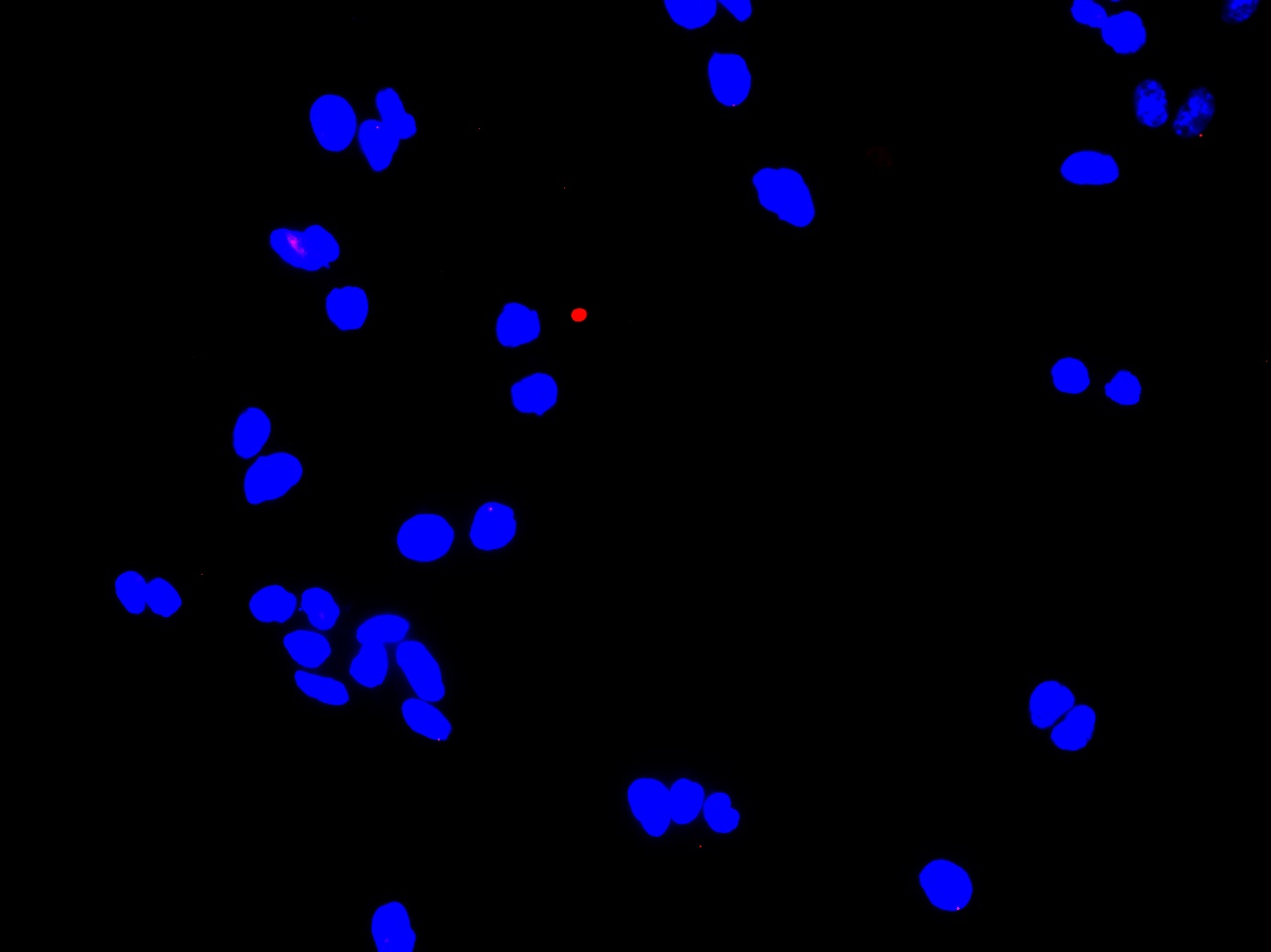

Supplement: Supplementary file 9 — Source data Fig. 7 [file 44321_2024_186_MOESM9_ESM.zip › Figure 7/7E/recombinant protein/PlexinD1-cMet/Vehicle_neg.jpg]

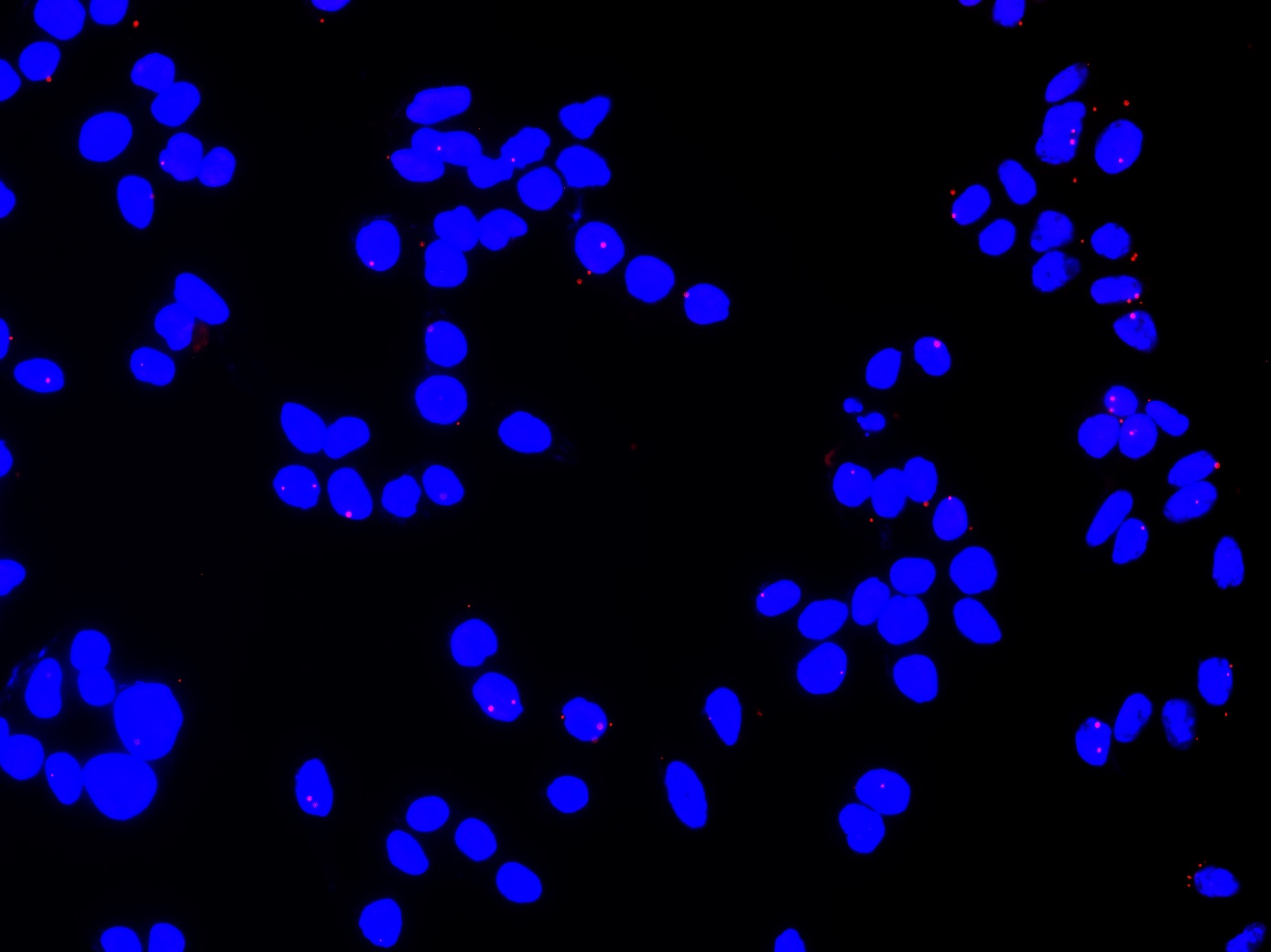

Supplement: Supplementary file 9 — Source data Fig. 7 [file 44321_2024_186_MOESM9_ESM.zip › Figure 7/7E/recombinant protein/PlexinD1-cMet/rSema3C.jpg]

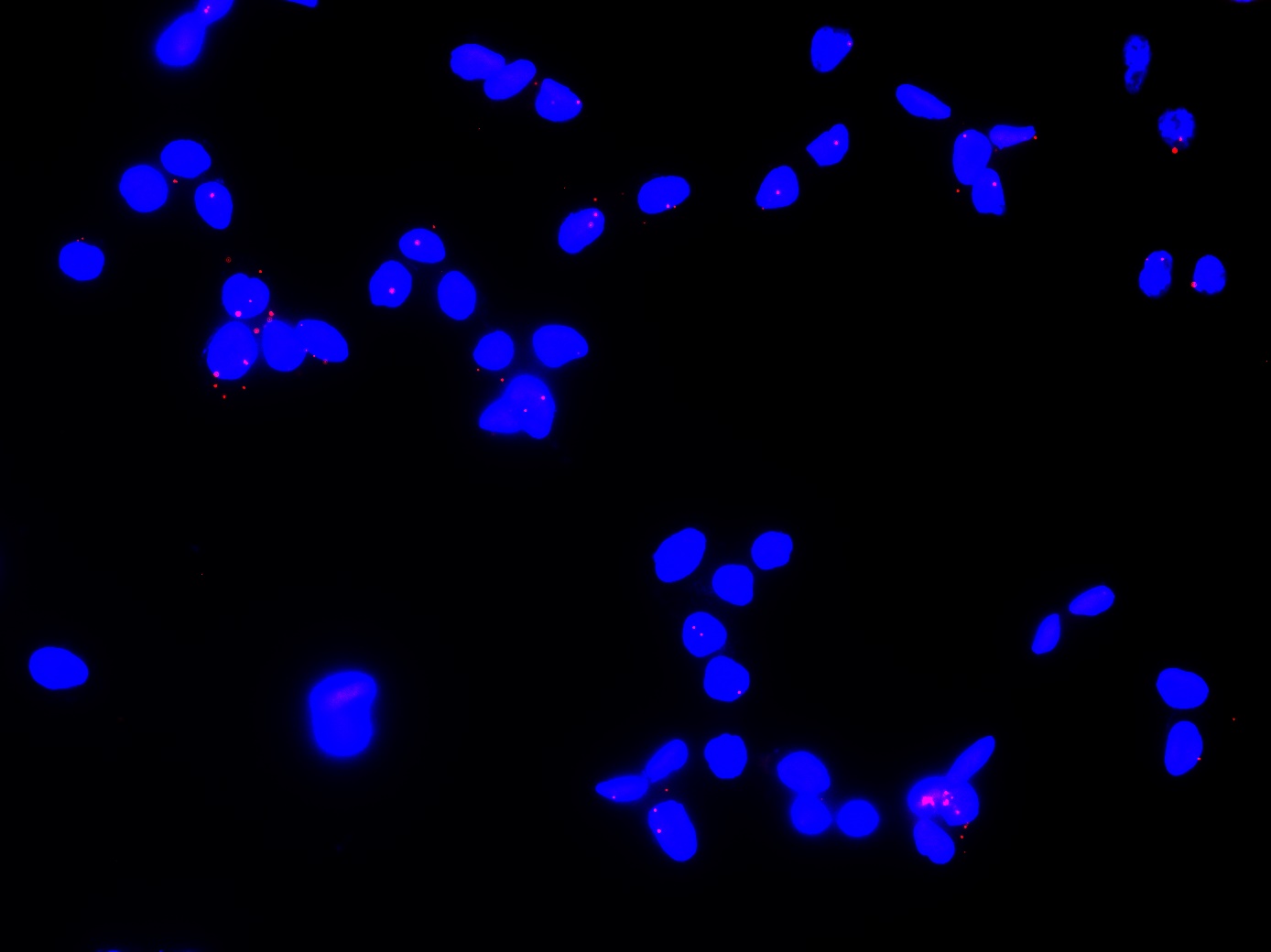

Supplement: Supplementary file 9 — Source data Fig. 7 [file 44321_2024_186_MOESM9_ESM.zip › Figure 7/7E/recombinant protein/PlexinD1-cMet/rSema3E.jpg]

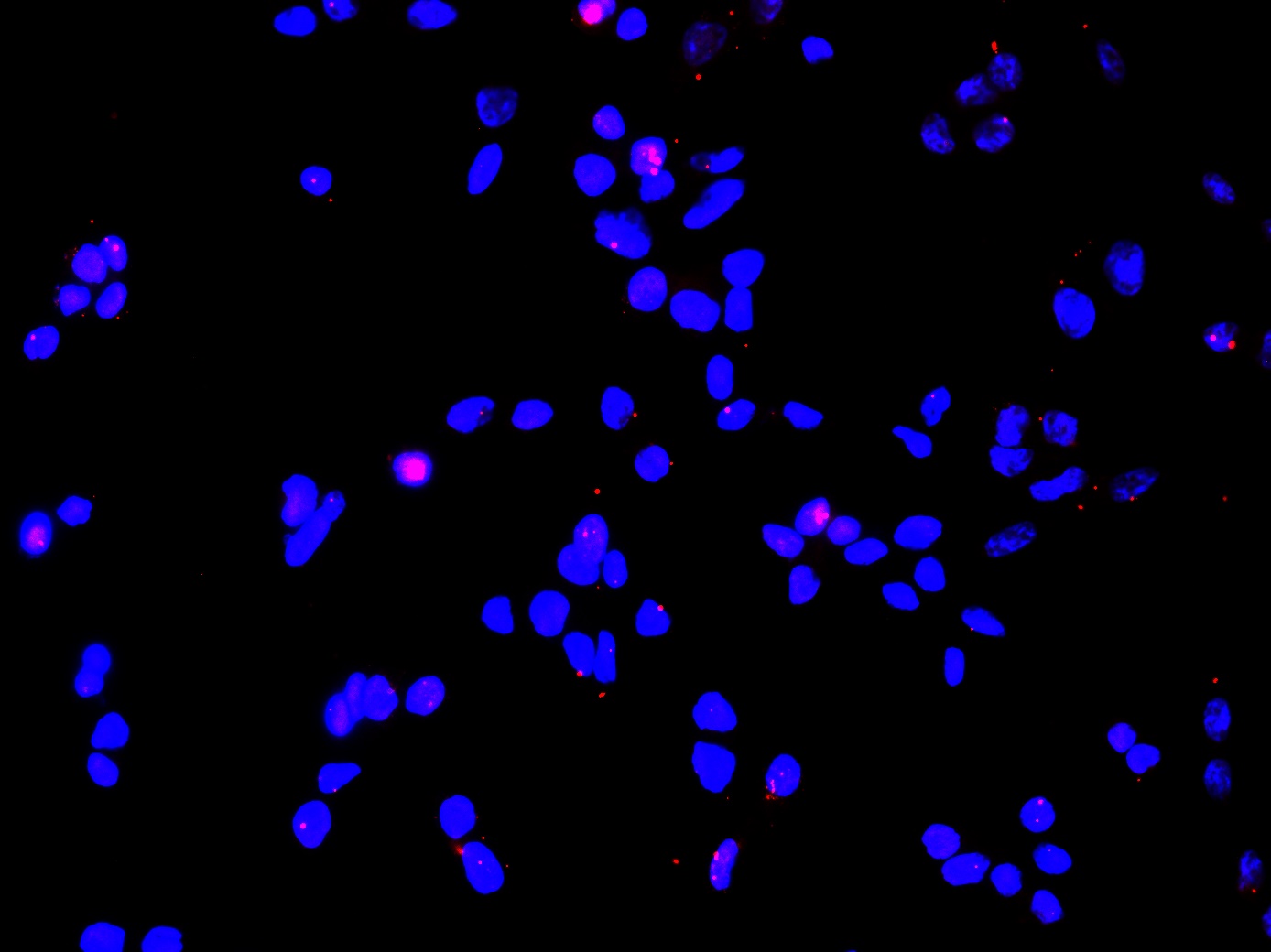

Supplement: Supplementary file 9 — Source data Fig. 7 [file 44321_2024_186_MOESM9_ESM.zip › Figure 7/7E/recombinant protein/PlexinD1-cMet/Vehicle.jpg]

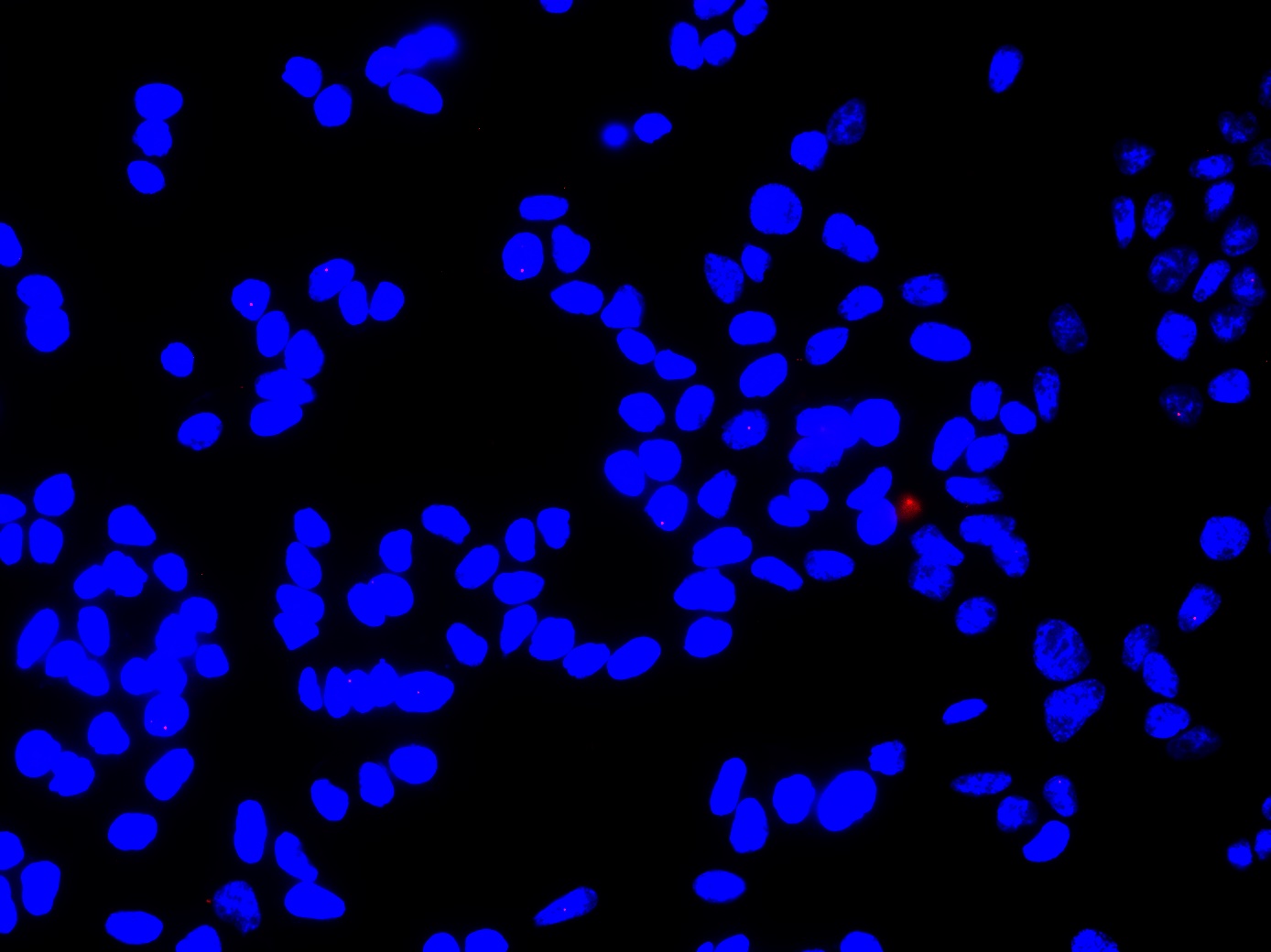

Supplement: Supplementary file 9 — Source data Fig. 7 [file 44321_2024_186_MOESM9_ESM.zip › Figure 7/7E/recombinant protein/PlexinD1-aErbB3/Vehicle_neg.jpg]

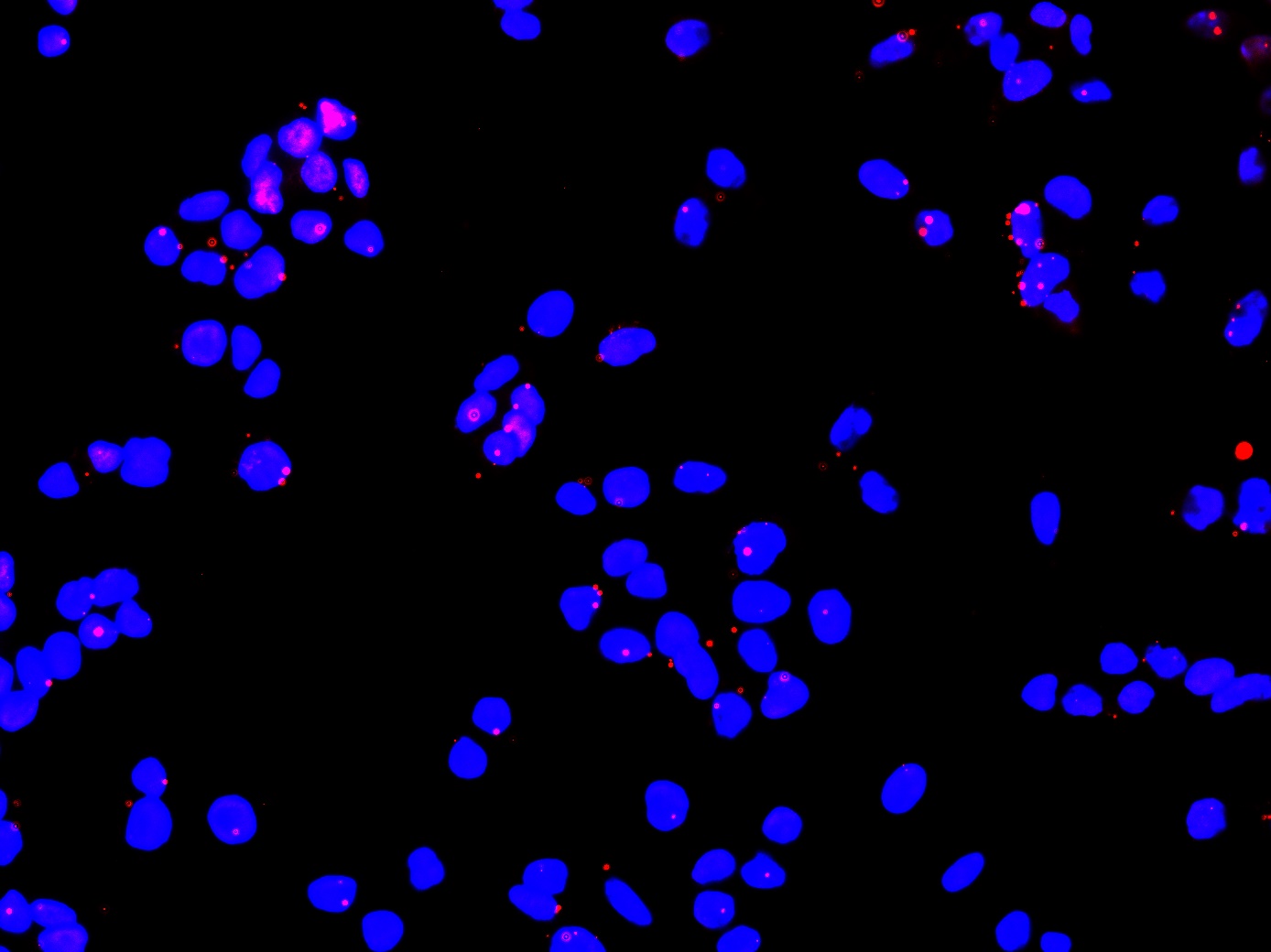

Supplement: Supplementary file 9 — Source data Fig. 7 [file 44321_2024_186_MOESM9_ESM.zip › Figure 7/7E/recombinant protein/PlexinD1-aErbB3/rSema3C.jpg]

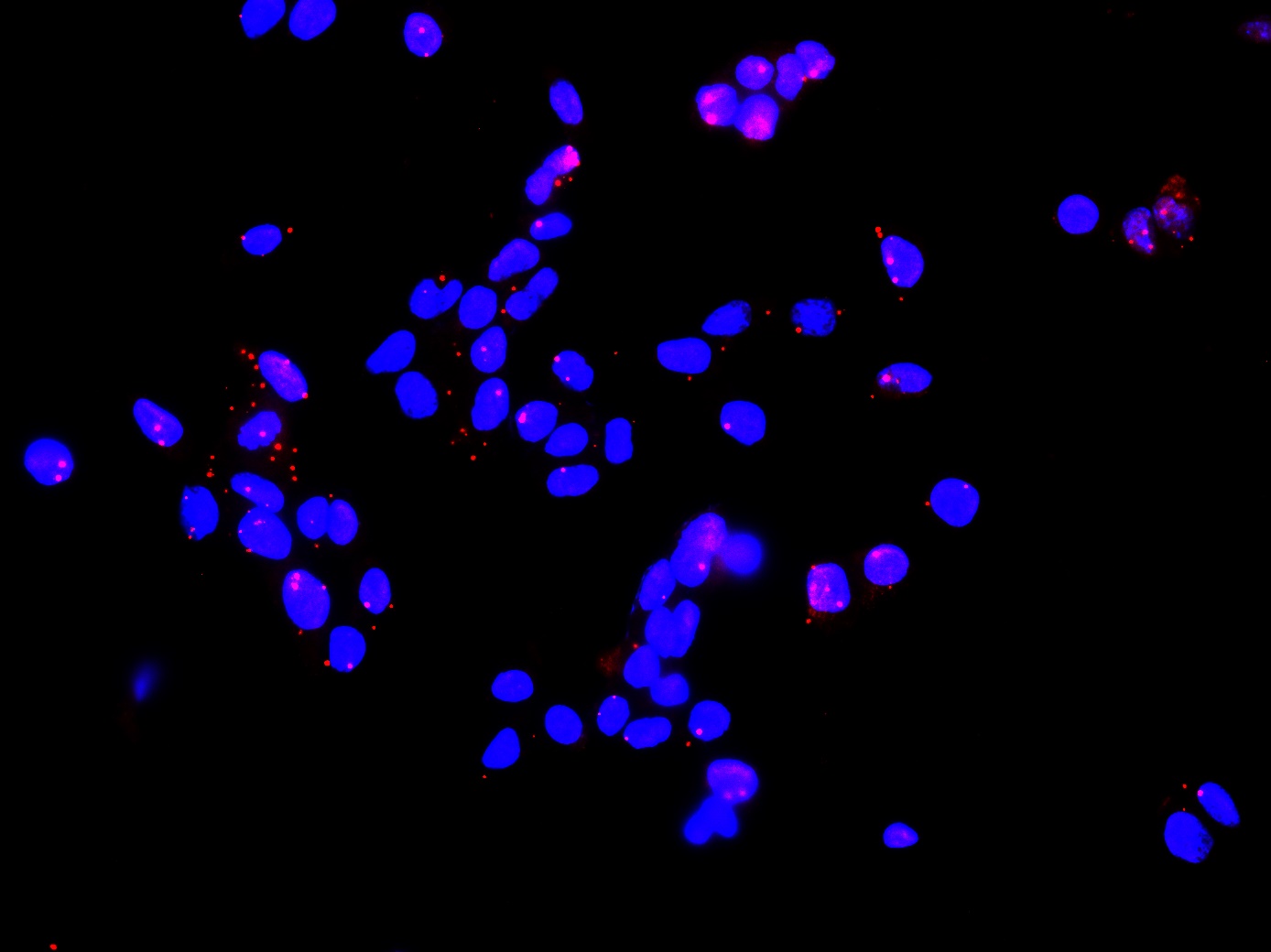

Supplement: Supplementary file 9 — Source data Fig. 7 [file 44321_2024_186_MOESM9_ESM.zip › Figure 7/7E/recombinant protein/PlexinD1-aErbB3/rSema3E.jpg]

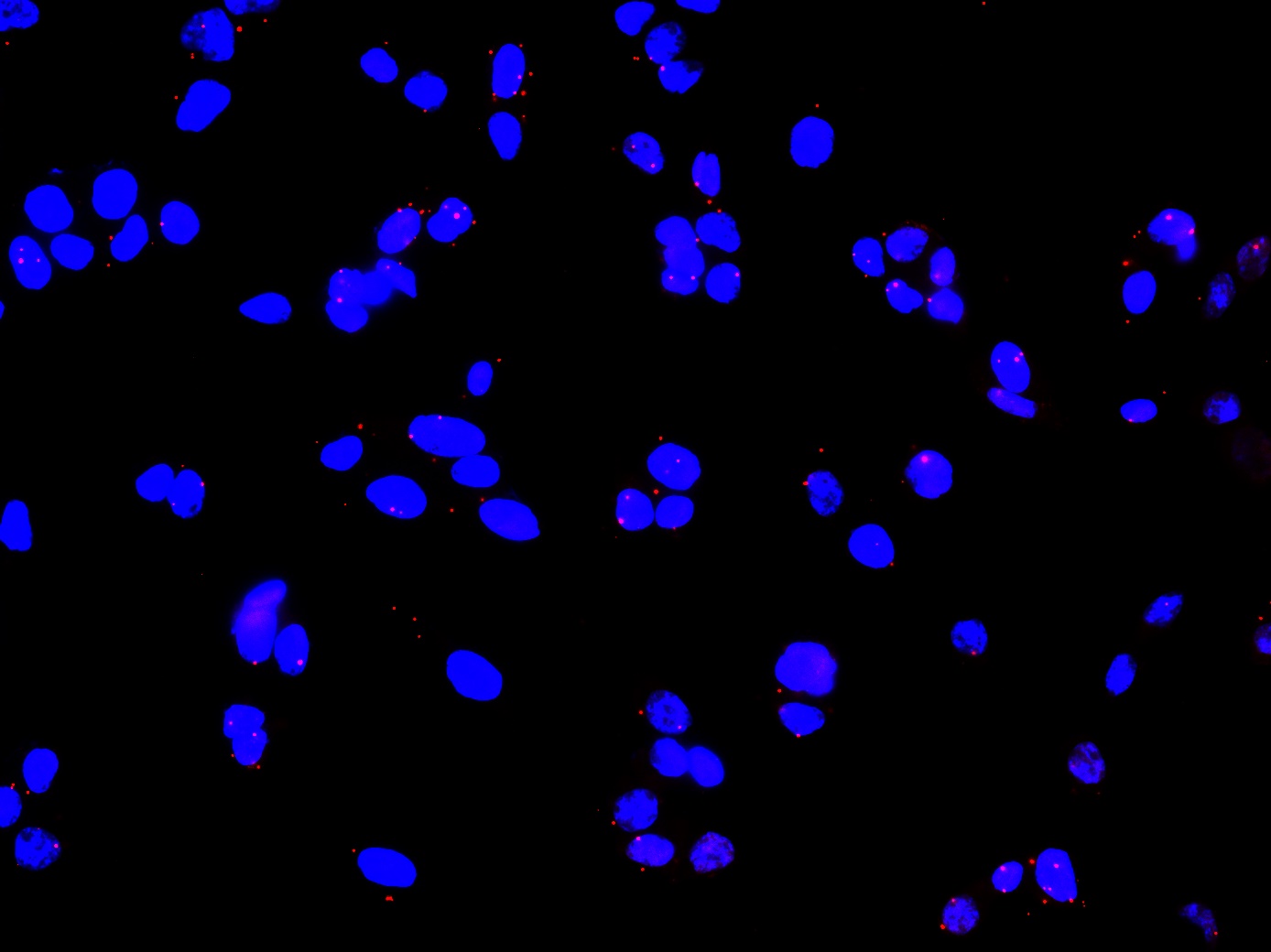

Supplement: Supplementary file 9 — Source data Fig. 7 [file 44321_2024_186_MOESM9_ESM.zip › Figure 7/7E/recombinant protein/PlexinD1-aErbB3/Vehicle.jpg]

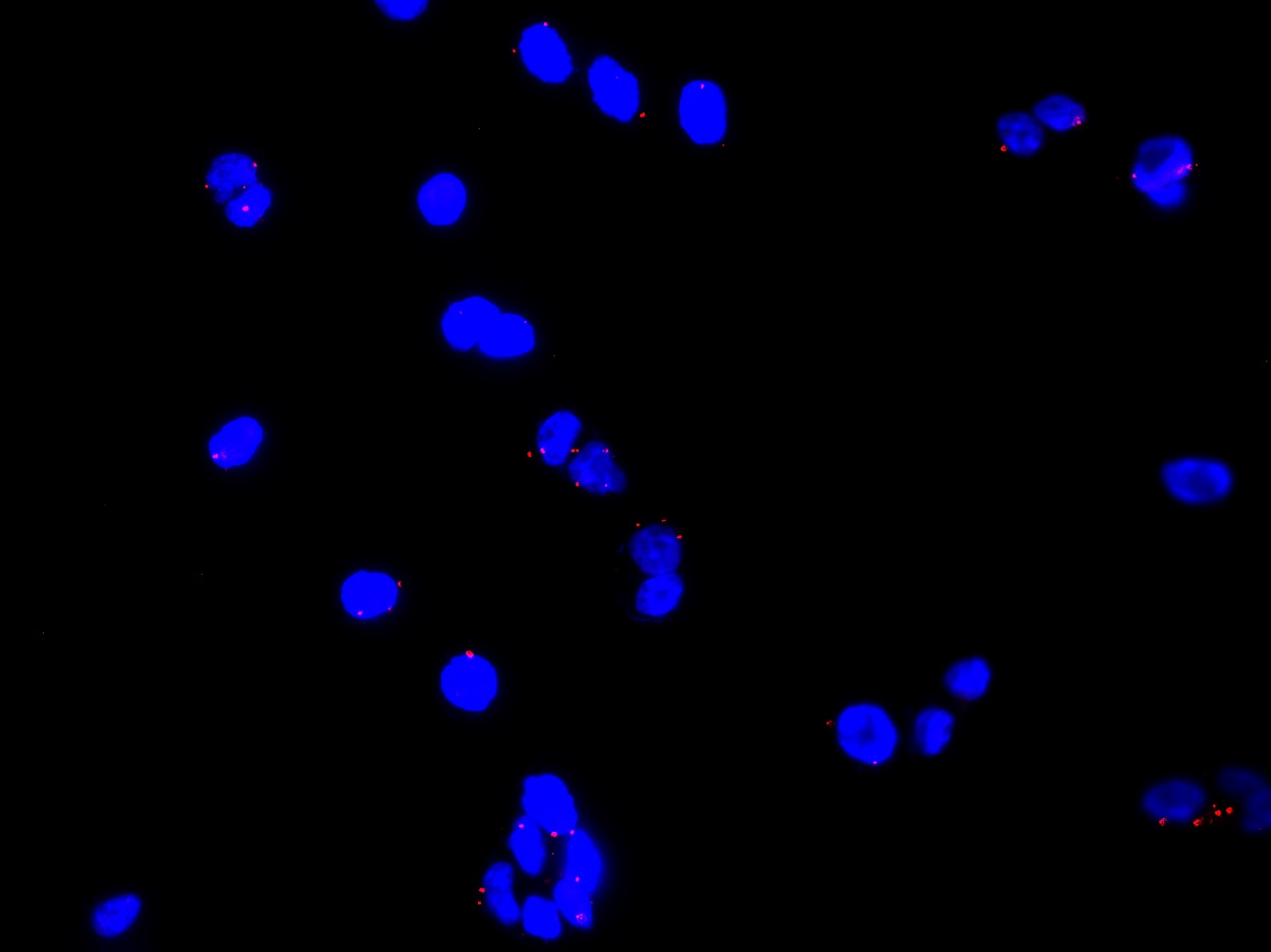

Supplement: Supplementary file 9 — Source data Fig. 7 [file 44321_2024_186_MOESM9_ESM.zip › Figure 7/7E/siRNA/PlexinD1-cMet/siCon.jpg]

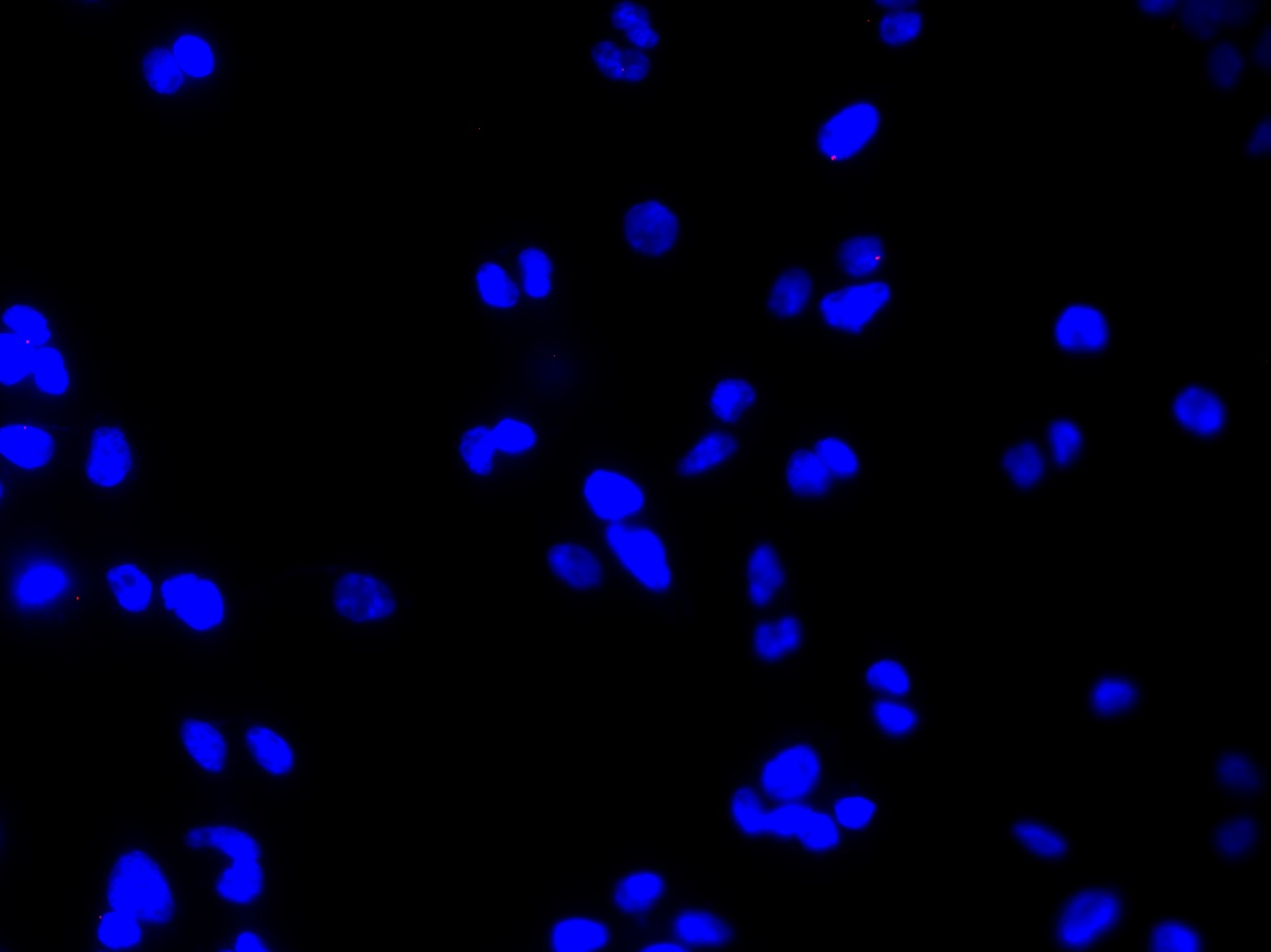

Supplement: Supplementary file 9 — Source data Fig. 7 [file 44321_2024_186_MOESM9_ESM.zip › Figure 7/7E/siRNA/PlexinD1-cMet/siCon_neg.jpg]

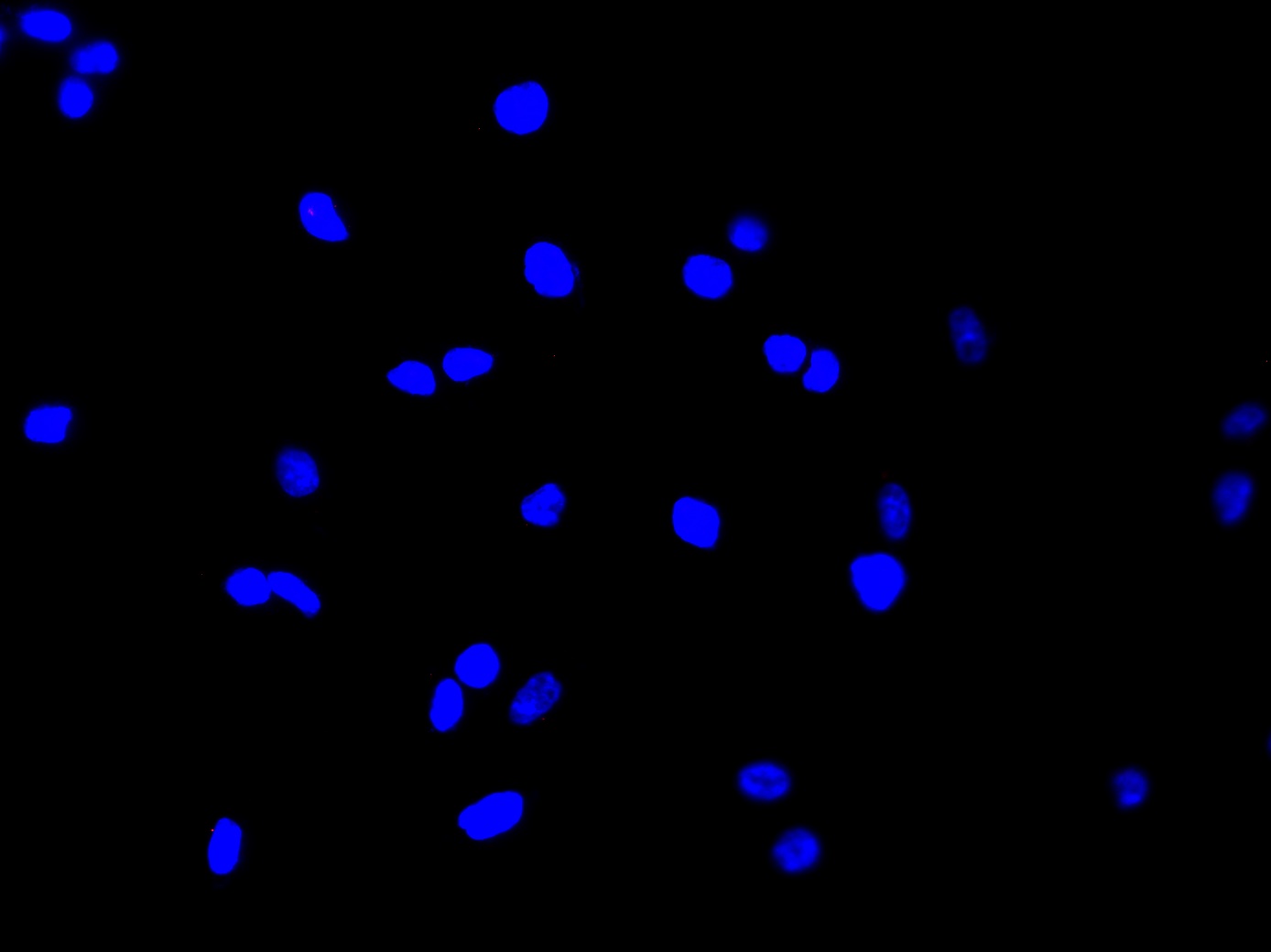

Supplement: Supplementary file 9 — Source data Fig. 7 [file 44321_2024_186_MOESM9_ESM.zip › Figure 7/7E/siRNA/PlexinD1-cMet/siSema3C.jpg]

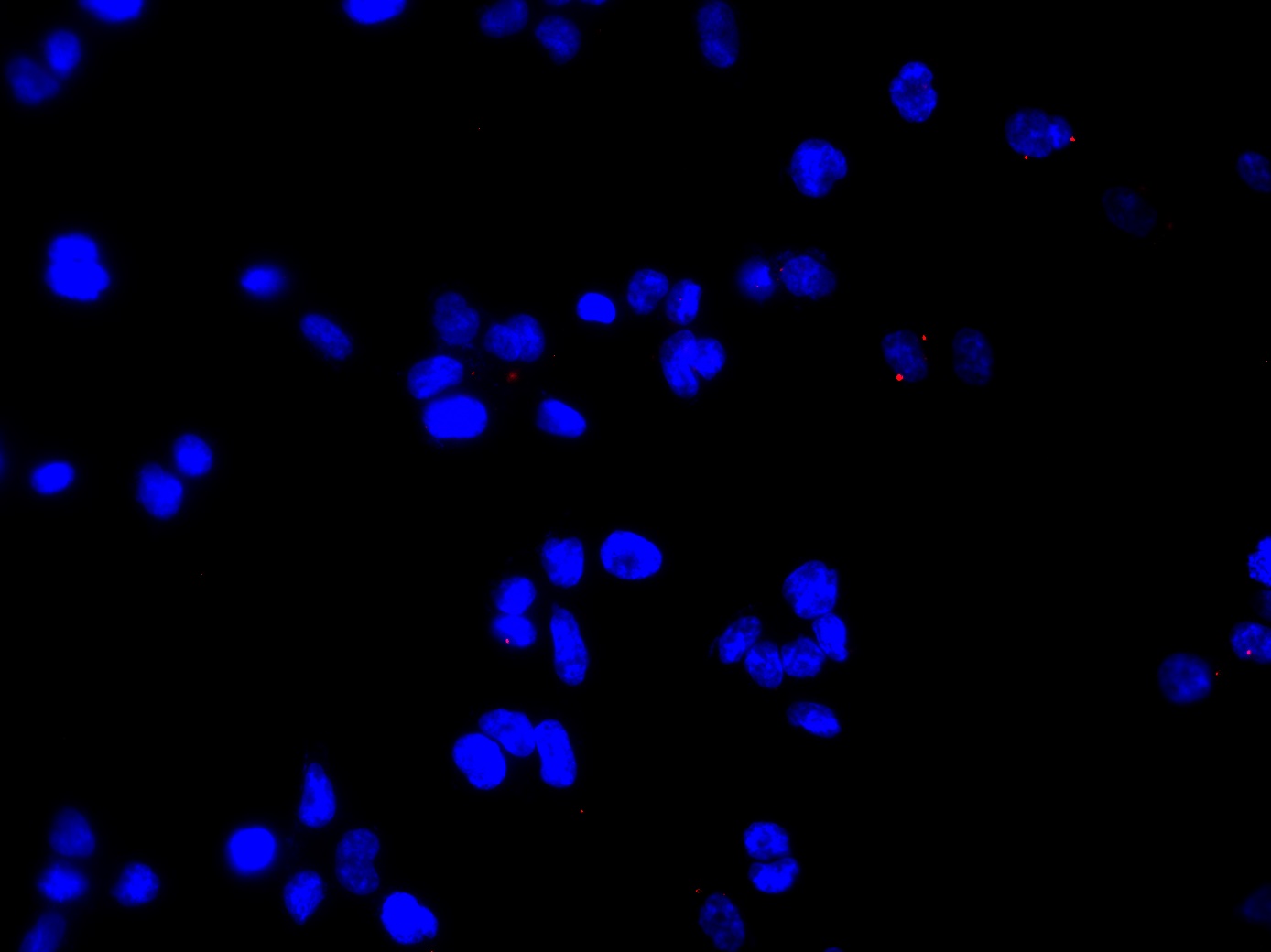

Supplement: Supplementary file 9 — Source data Fig. 7 [file 44321_2024_186_MOESM9_ESM.zip › Figure 7/7E/siRNA/PlexinD1-cMet/siSema3E.jpg]

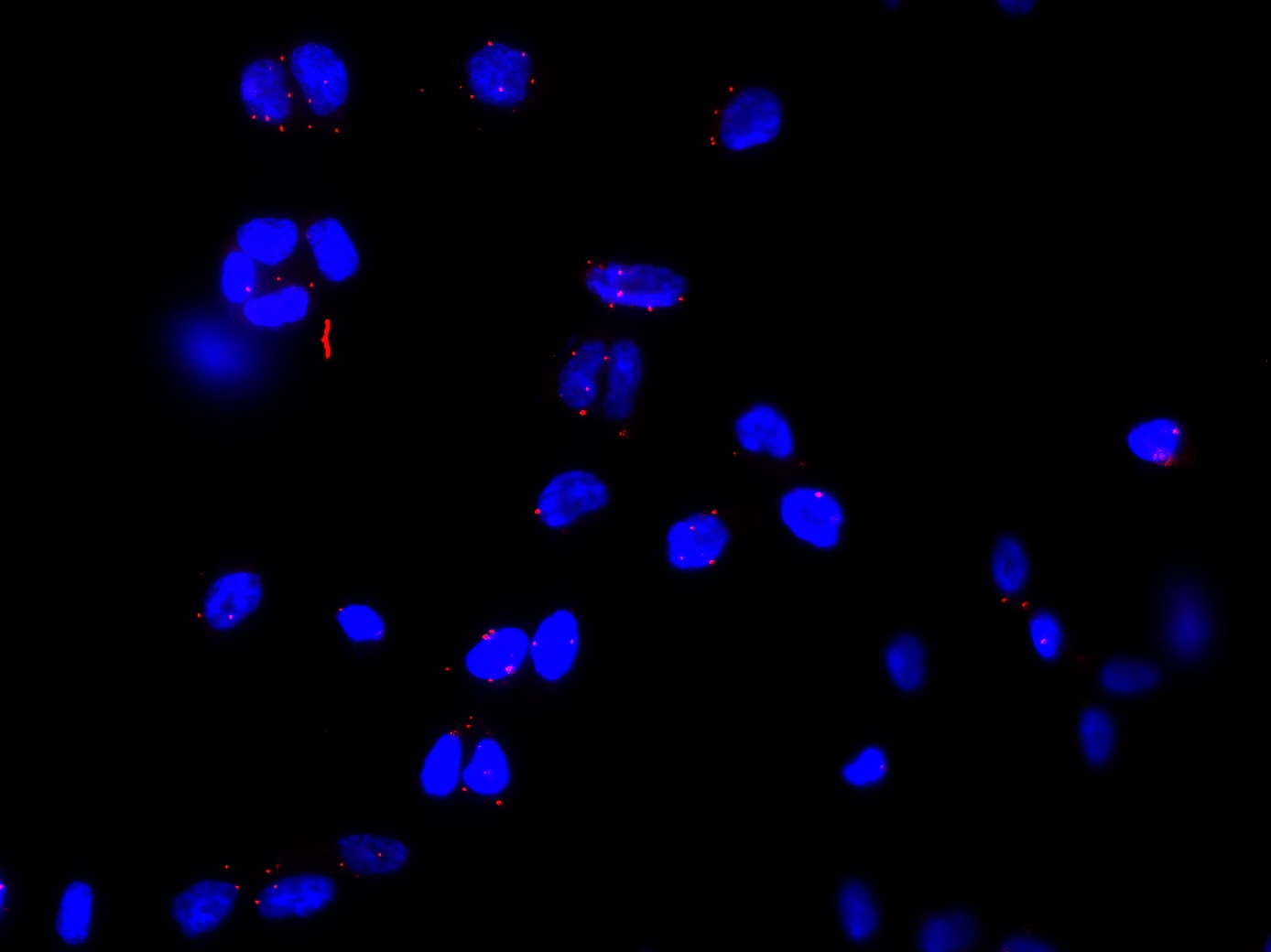

Supplement: Supplementary file 9 — Source data Fig. 7 [file 44321_2024_186_MOESM9_ESM.zip › Figure 7/7E/siRNA/PlexinD1-aErbB3/siCon.jpg]

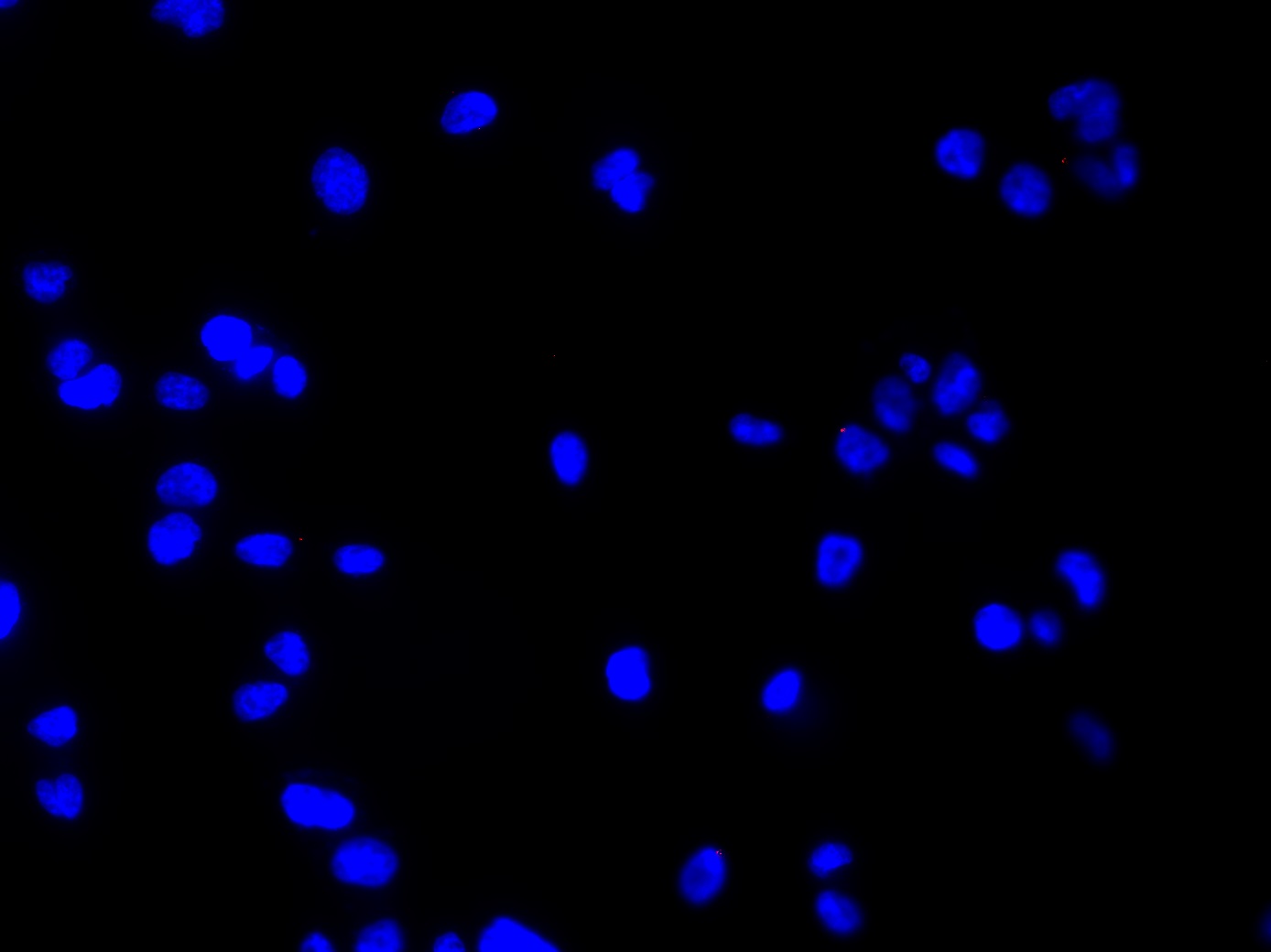

Supplement: Supplementary file 9 — Source data Fig. 7 [file 44321_2024_186_MOESM9_ESM.zip › Figure 7/7E/siRNA/PlexinD1-aErbB3/siCon_neg.jpg]

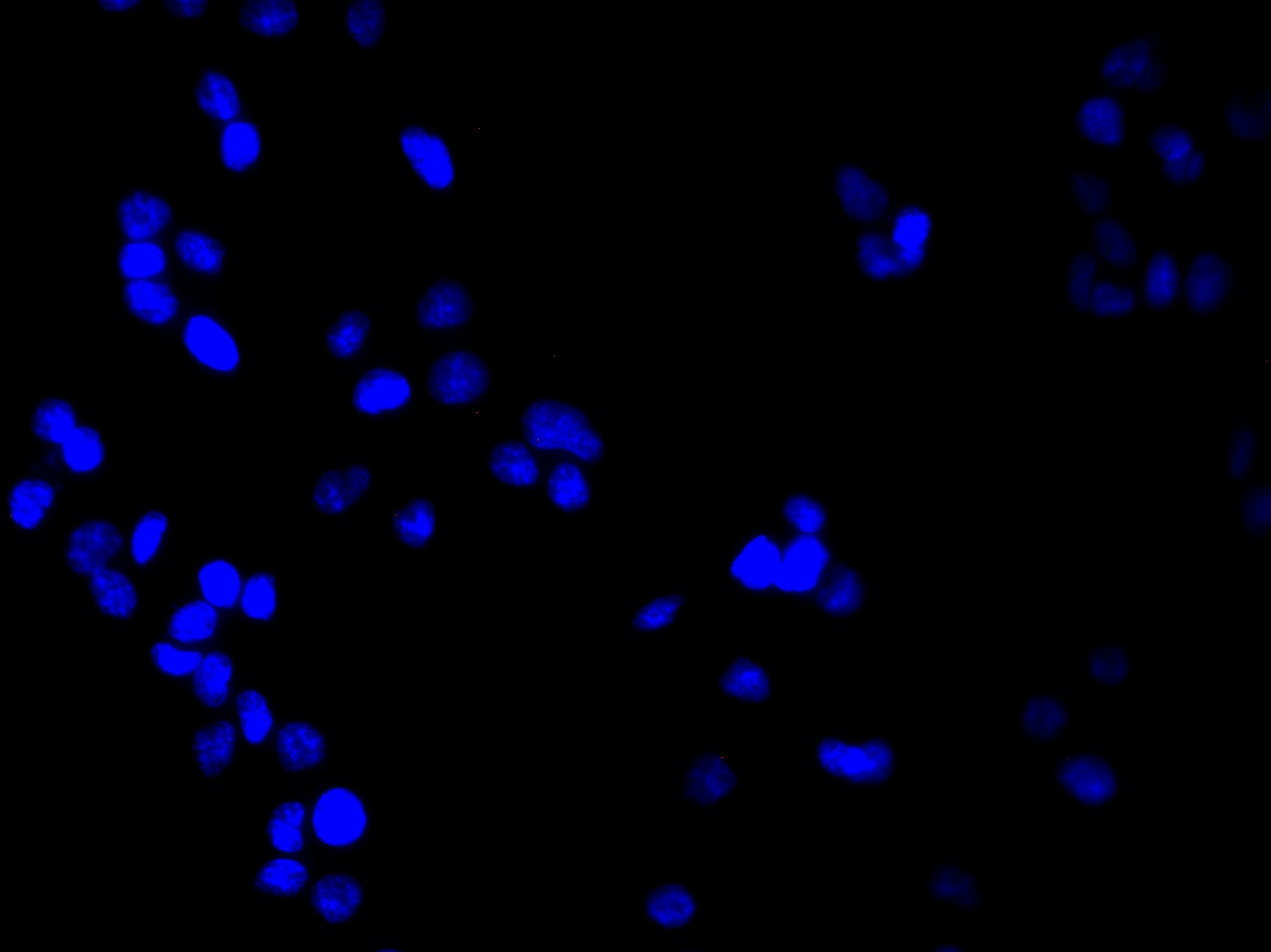

Supplement: Supplementary file 9 — Source data Fig. 7 [file 44321_2024_186_MOESM9_ESM.zip › Figure 7/7E/siRNA/PlexinD1-aErbB3/siSema3C.jpg]

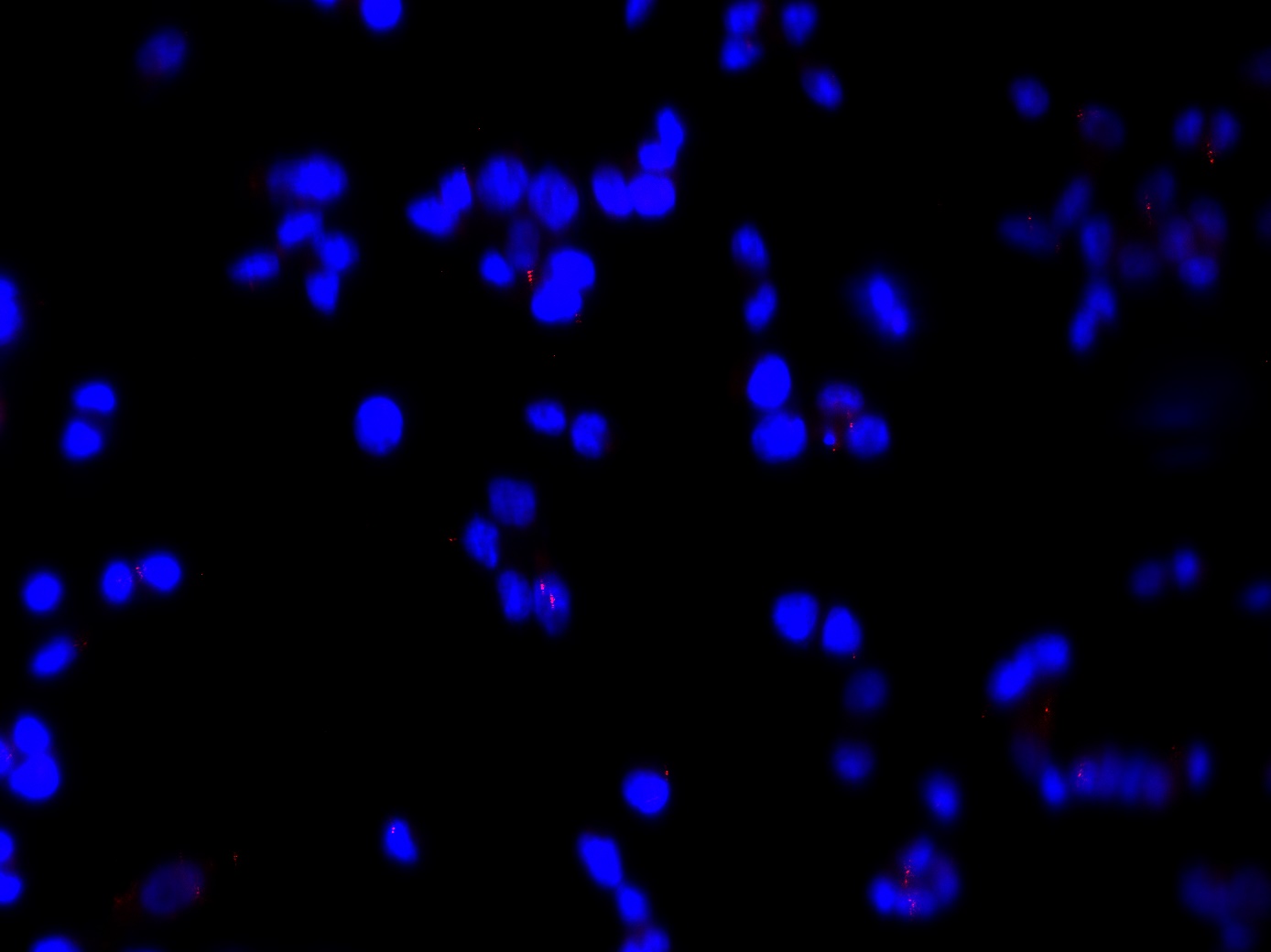

Supplement: Supplementary file 9 — Source data Fig. 7 [file 44321_2024_186_MOESM9_ESM.zip › Figure 7/7E/siRNA/PlexinD1-aErbB3/siSema3E.jpg]

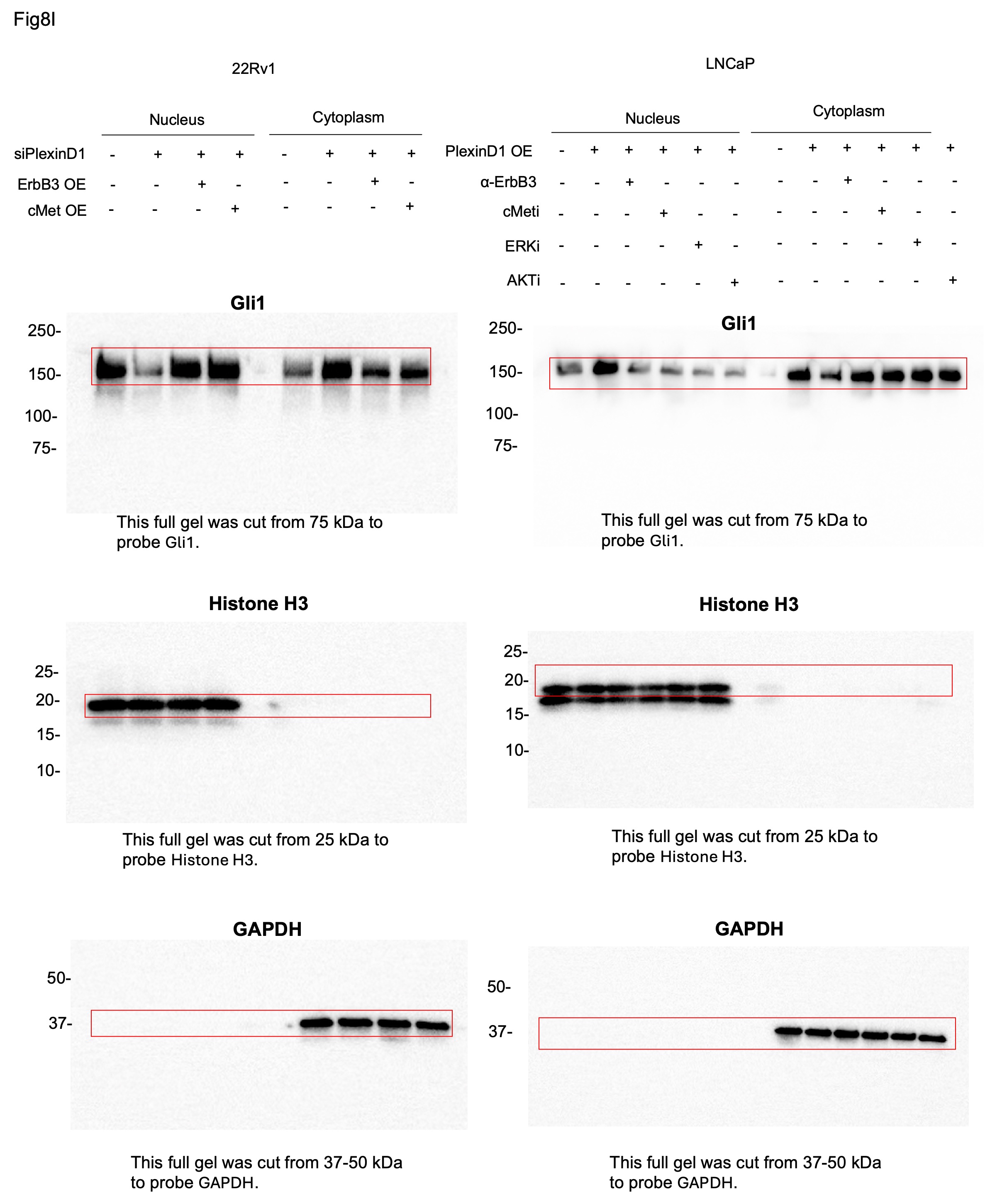

Supplement: Supplementary file 10 — Source data Fig. 8 [file 44321_2024_186_MOESM10_ESM.zip › Figure 8/8I/WB-8I.jpg]

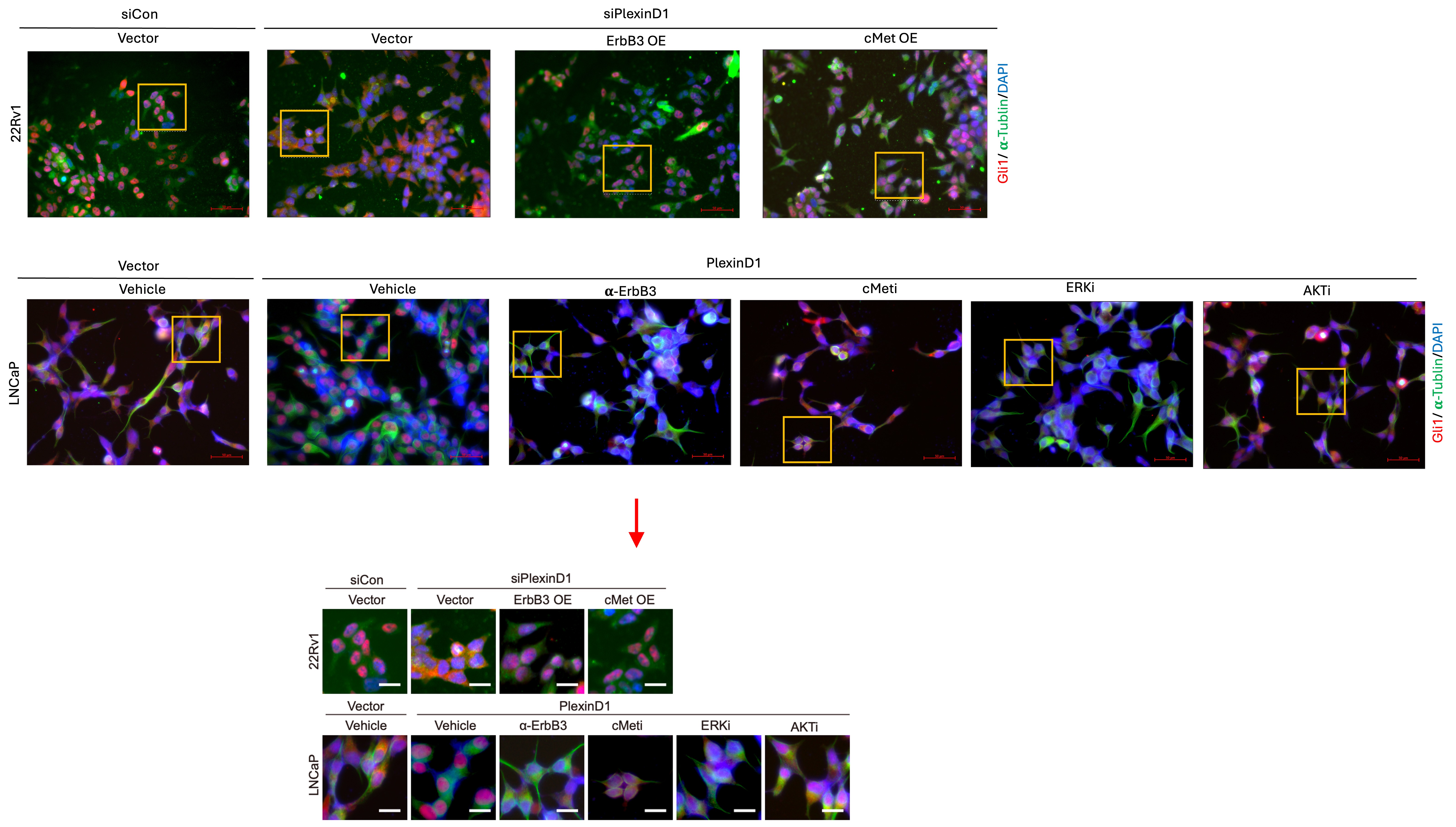

Supplement: Supplementary file 10 — Source data Fig. 8 [file 44321_2024_186_MOESM10_ESM.zip › Figure 8/8H/README.tif]

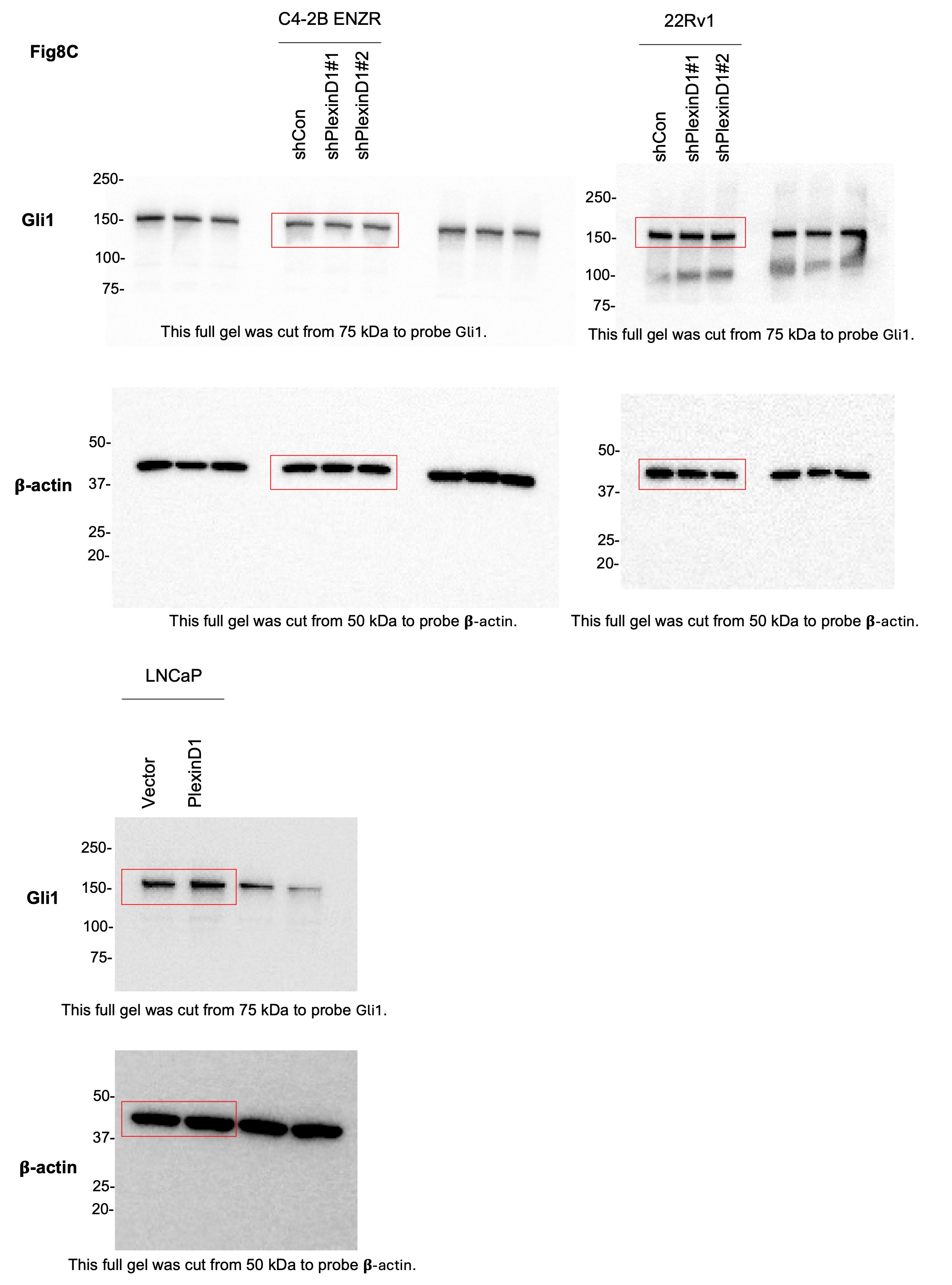

Supplement: Supplementary file 10 — Source data Fig. 8 [file 44321_2024_186_MOESM10_ESM.zip › Figure 8/8C/WB-8C.jpg]

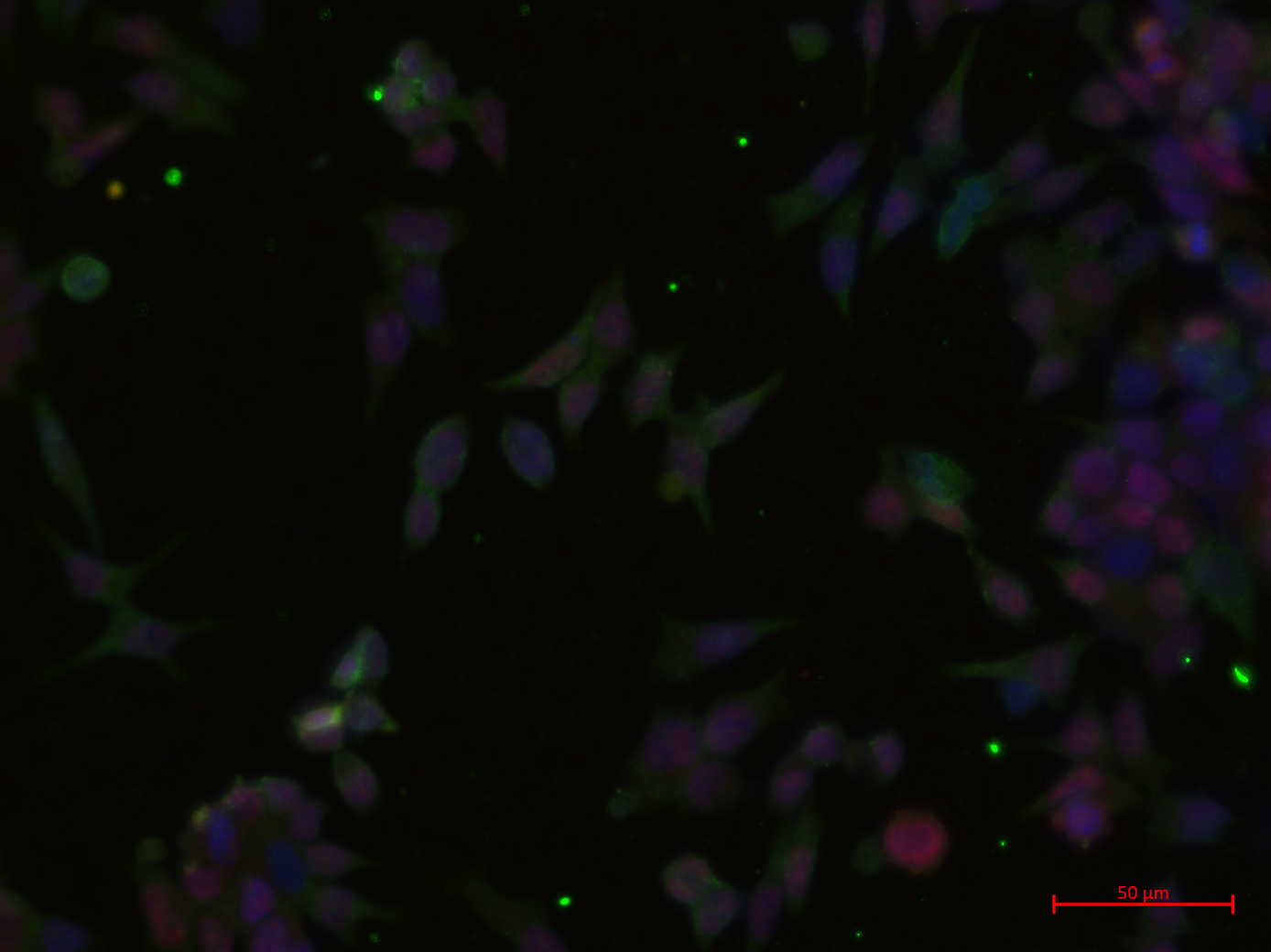

Supplement: Supplementary file 10 — Source data Fig. 8 [file 44321_2024_186_MOESM10_ESM.zip › Figure 8/8H/22Rv1/siPlexinD1-cMet OE.tif]

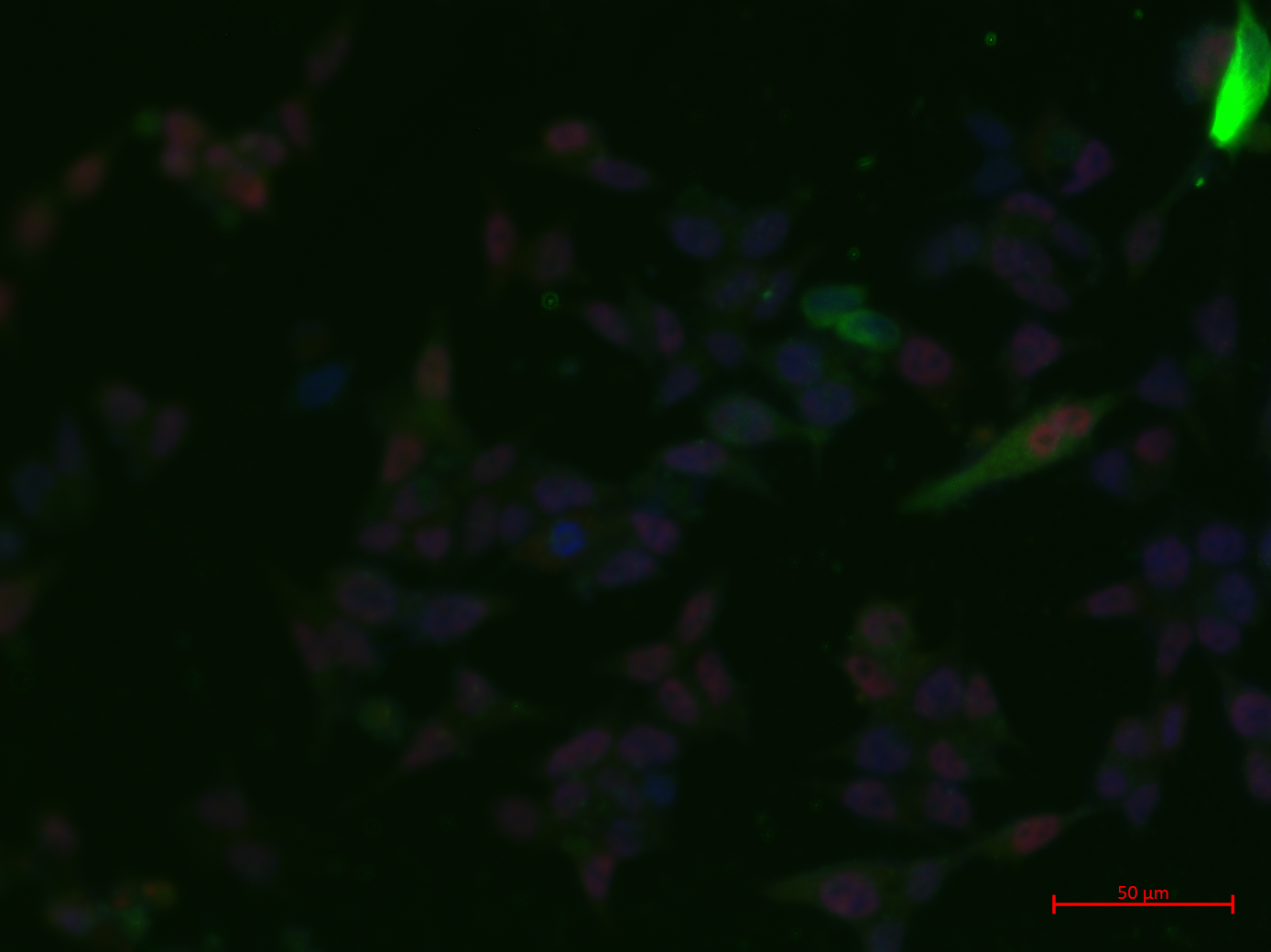

Supplement: Supplementary file 10 — Source data Fig. 8 [file 44321_2024_186_MOESM10_ESM.zip › Figure 8/8H/22Rv1/siPlexinD1-ErbB3 OE.tif]

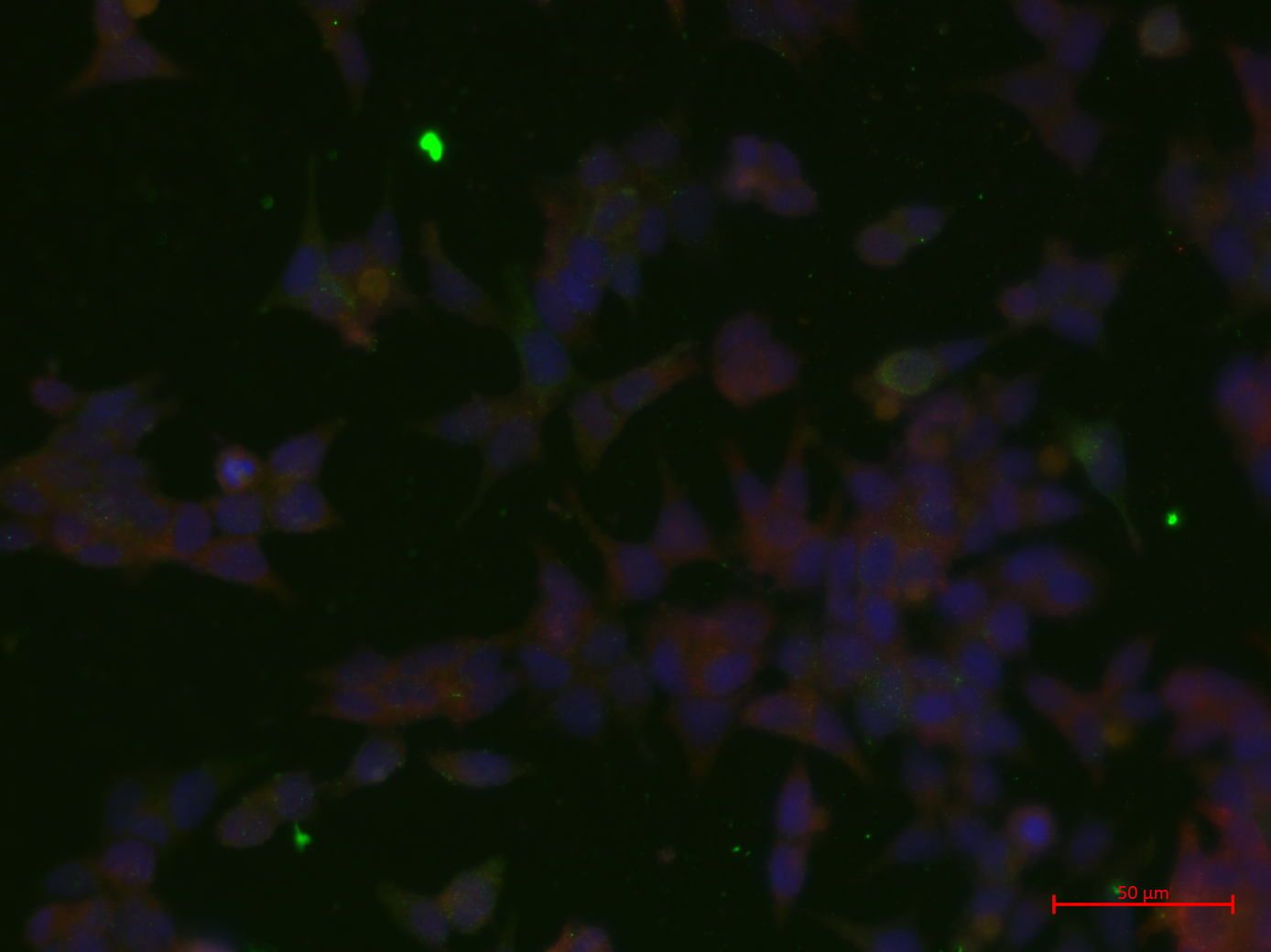

Supplement: Supplementary file 10 — Source data Fig. 8 [file 44321_2024_186_MOESM10_ESM.zip › Figure 8/8H/22Rv1/siPlexinD1-vector.tif]

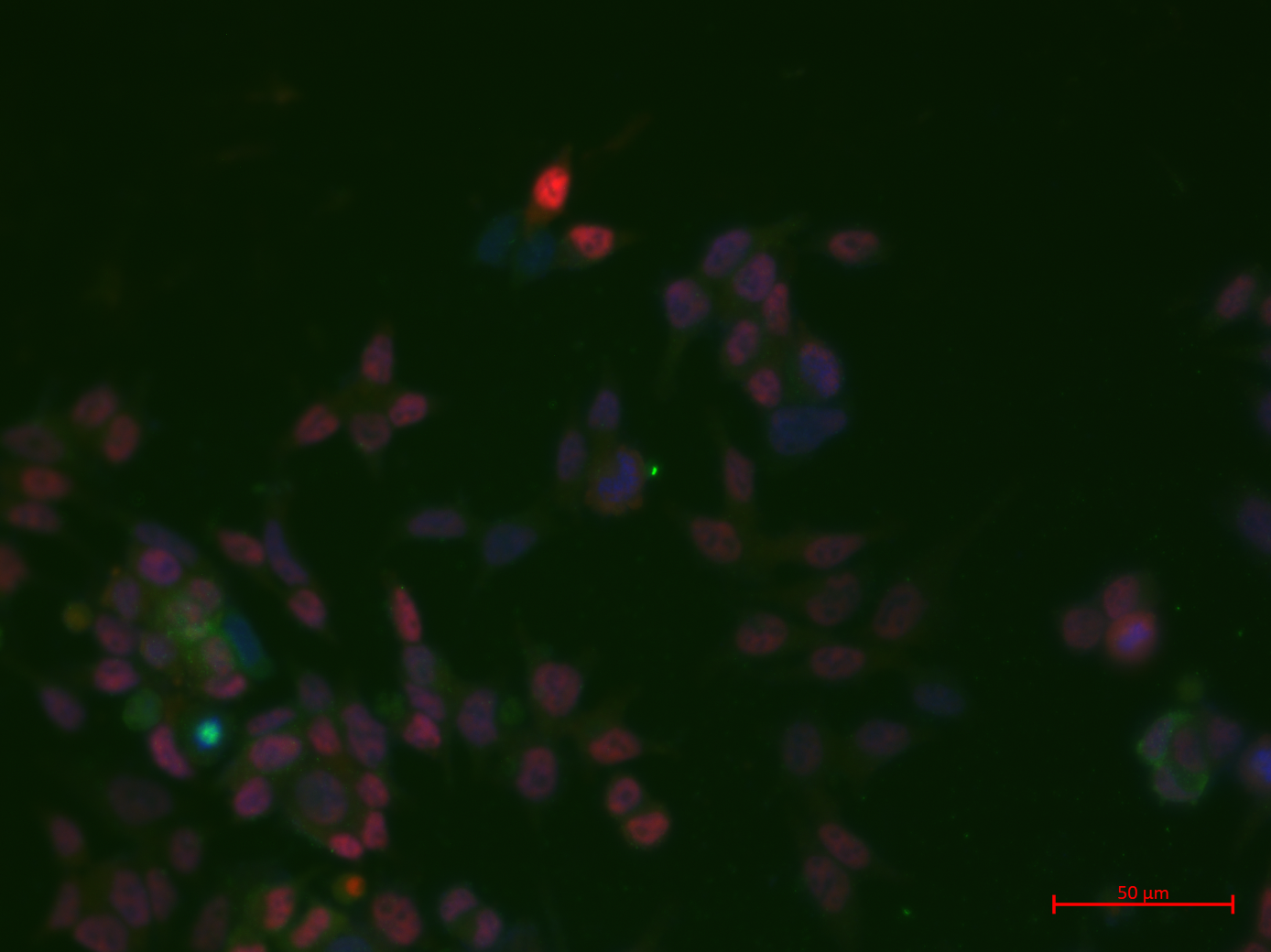

Supplement: Supplementary file 10 — Source data Fig. 8 [file 44321_2024_186_MOESM10_ESM.zip › Figure 8/8H/22Rv1/siControl.tif]

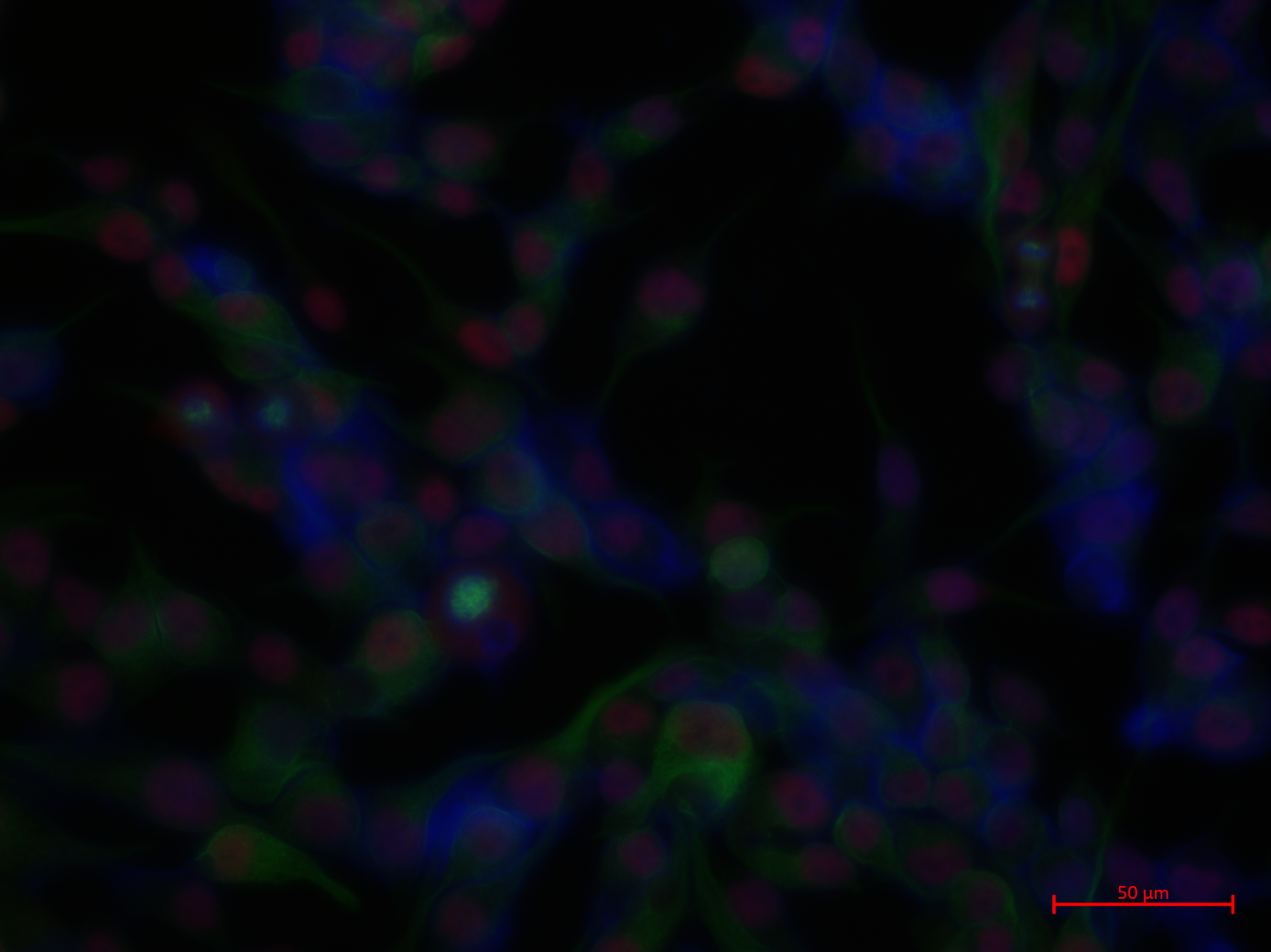

Supplement: Supplementary file 10 — Source data Fig. 8 [file 44321_2024_186_MOESM10_ESM.zip › Figure 8/8H/LNCaP/PlexinD1-vehicle.tif]

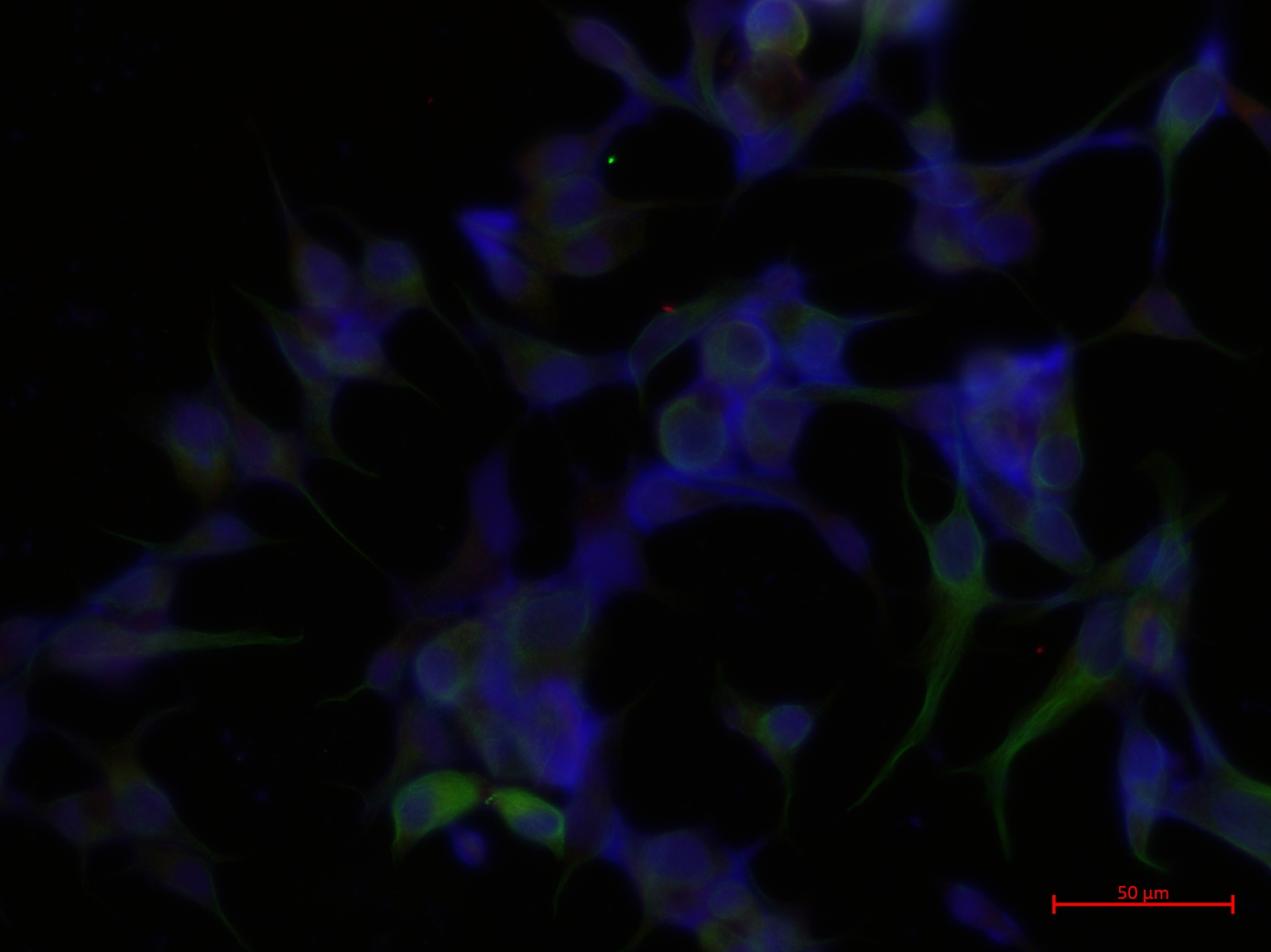

Supplement: Supplementary file 10 — Source data Fig. 8 [file 44321_2024_186_MOESM10_ESM.zip › Figure 8/8H/LNCaP/PlexinD1-ERKi.tif]

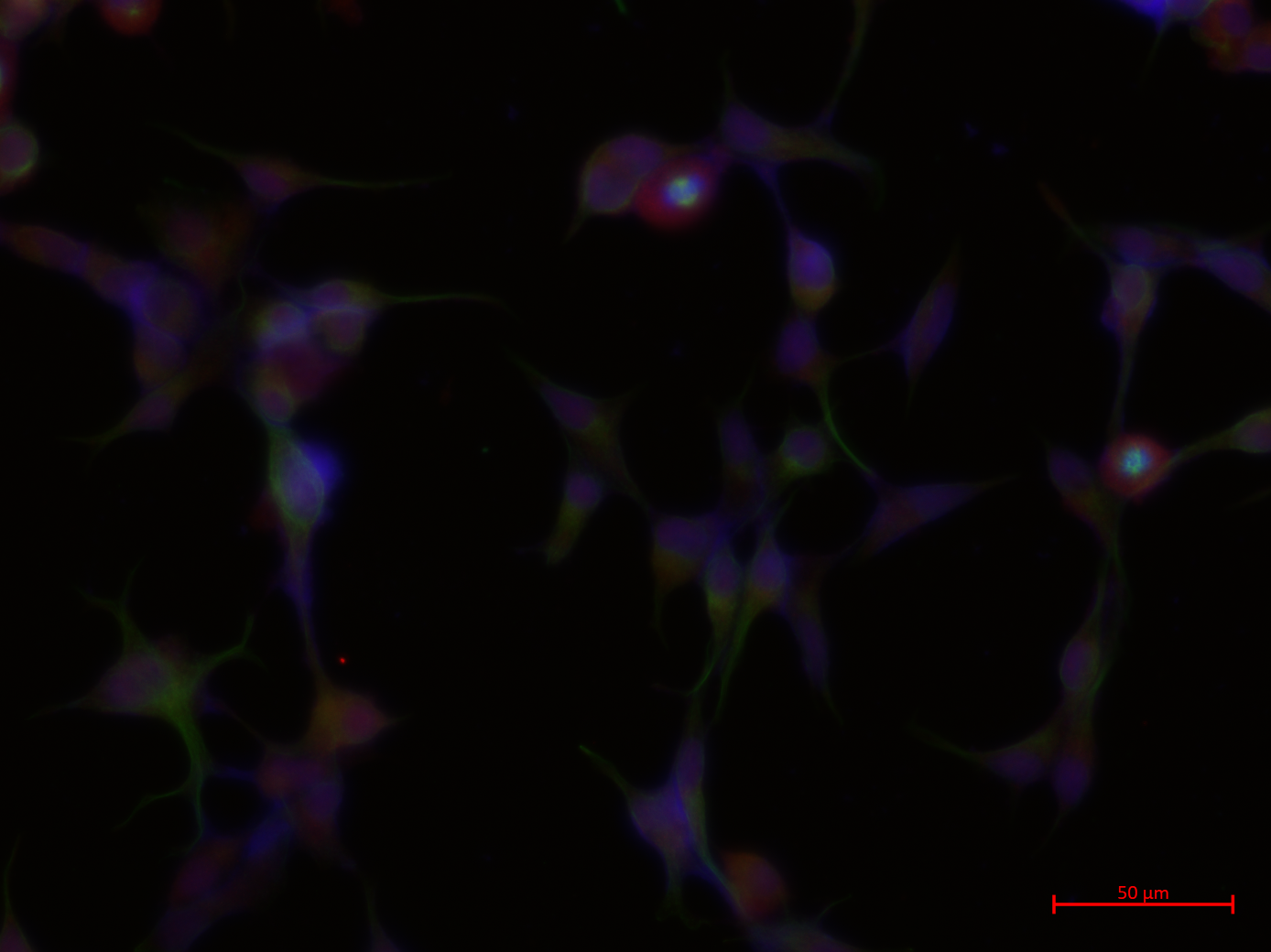

Supplement: Supplementary file 10 — Source data Fig. 8 [file 44321_2024_186_MOESM10_ESM.zip › Figure 8/8H/LNCaP/PlexinD1-AKTi.tif]

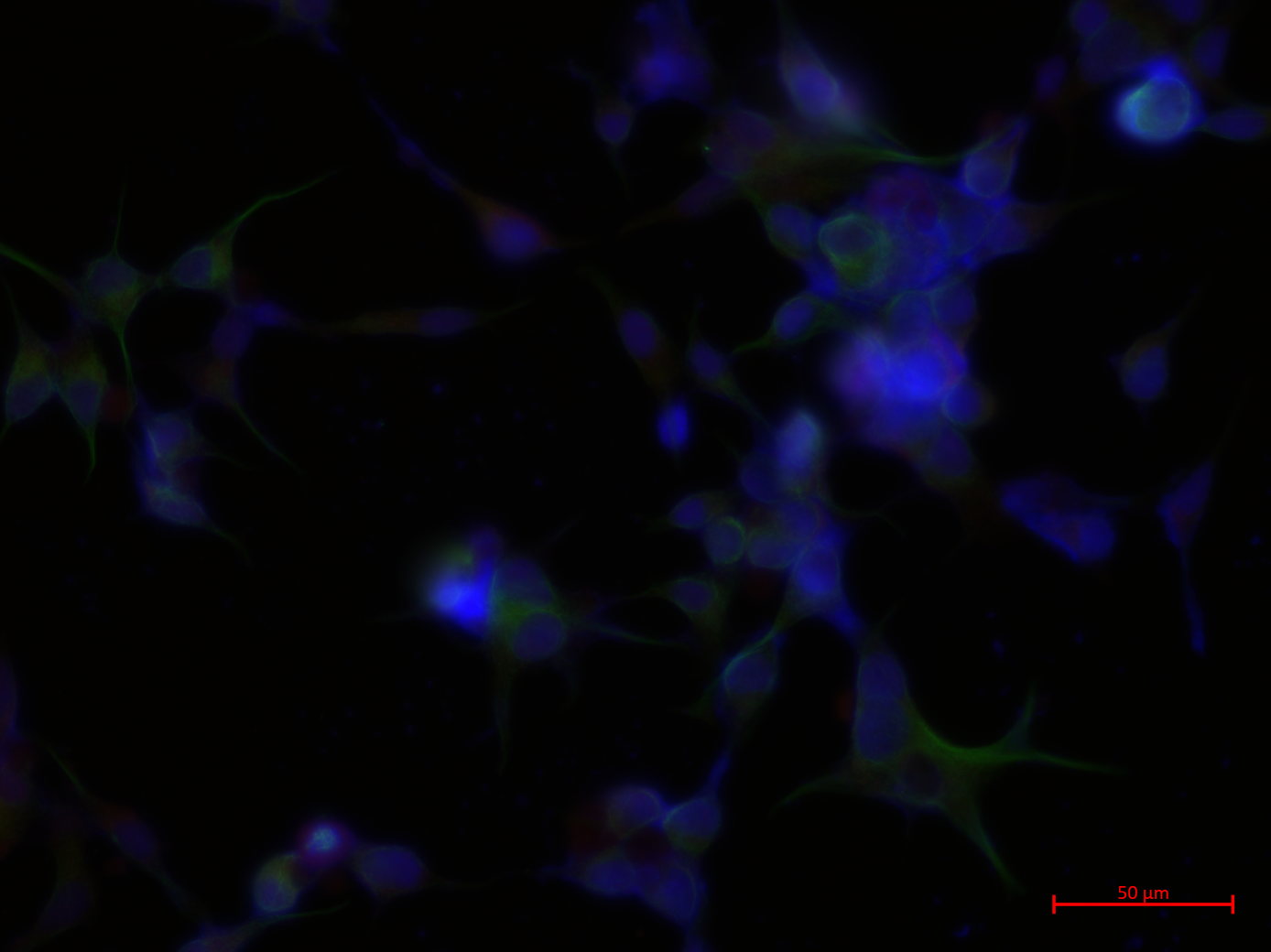

Supplement: Supplementary file 10 — Source data Fig. 8 [file 44321_2024_186_MOESM10_ESM.zip › Figure 8/8H/LNCaP/PlexinD1-aErbB3.tif]

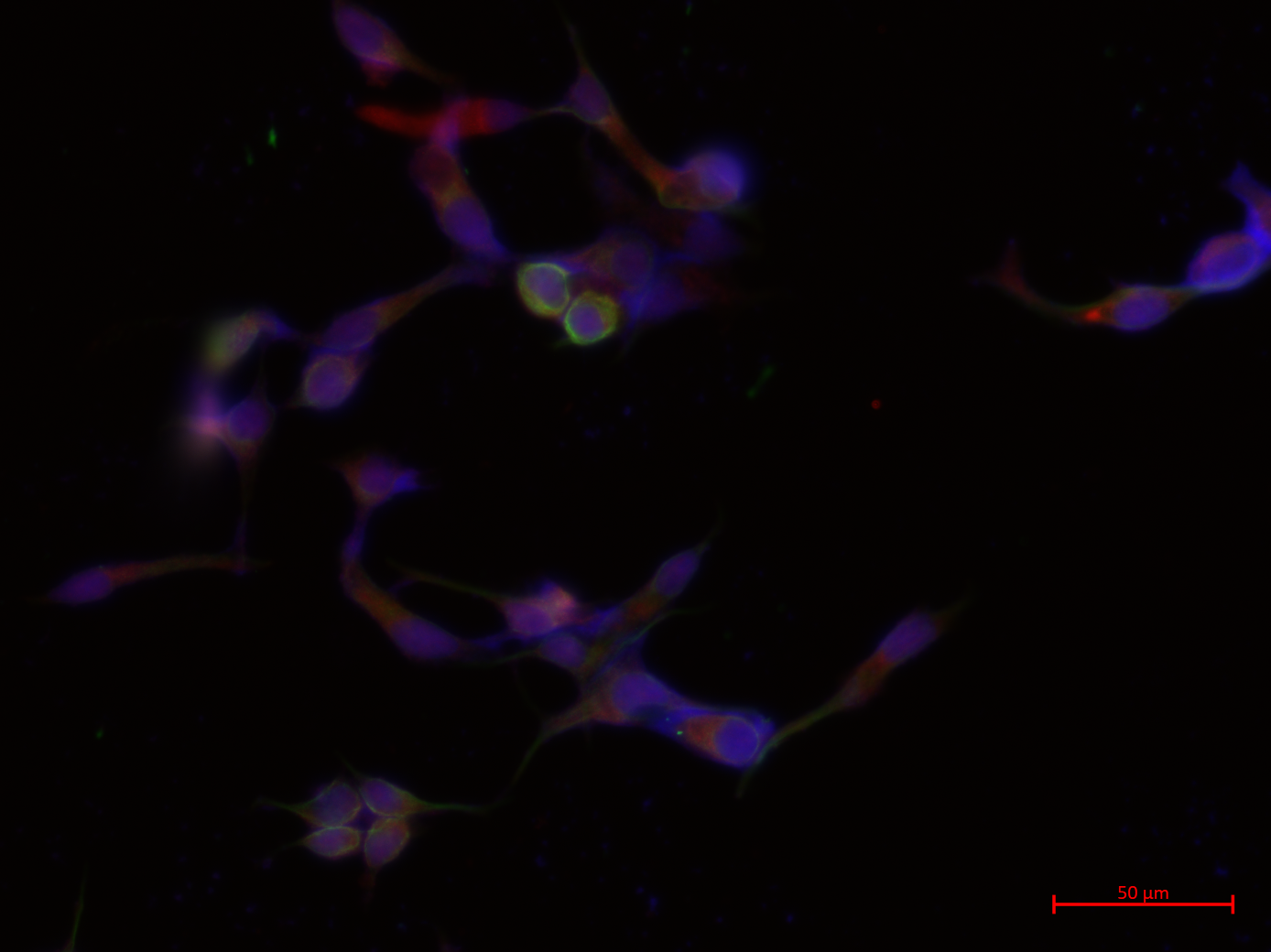

Supplement: Supplementary file 10 — Source data Fig. 8 [file 44321_2024_186_MOESM10_ESM.zip › Figure 8/8H/LNCaP/PlexinD1-cMeti.tif]

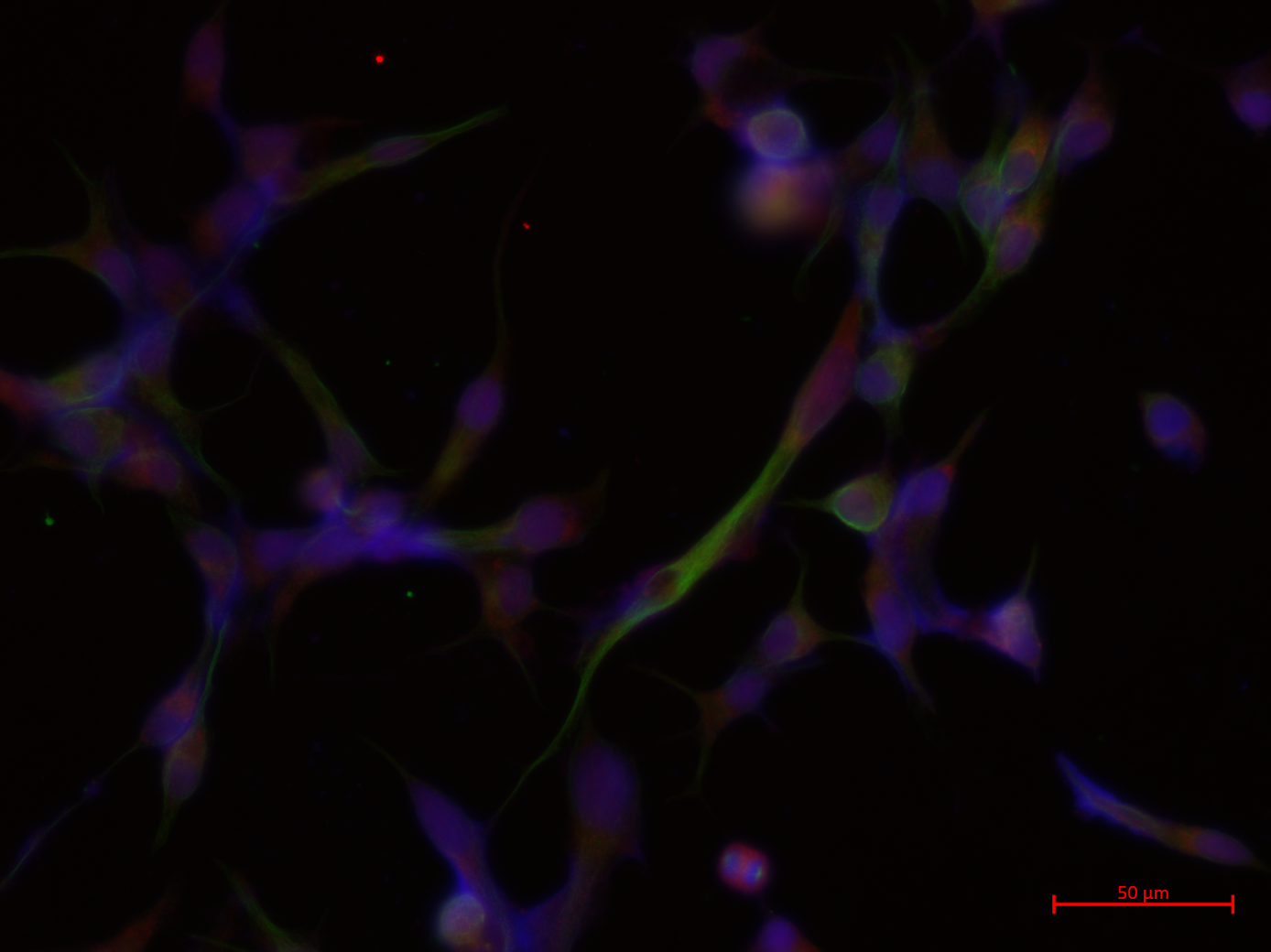

Supplement: Supplementary file 10 — Source data Fig. 8 [file 44321_2024_186_MOESM10_ESM.zip › Figure 8/8H/LNCaP/Vector-vehicle.tif]

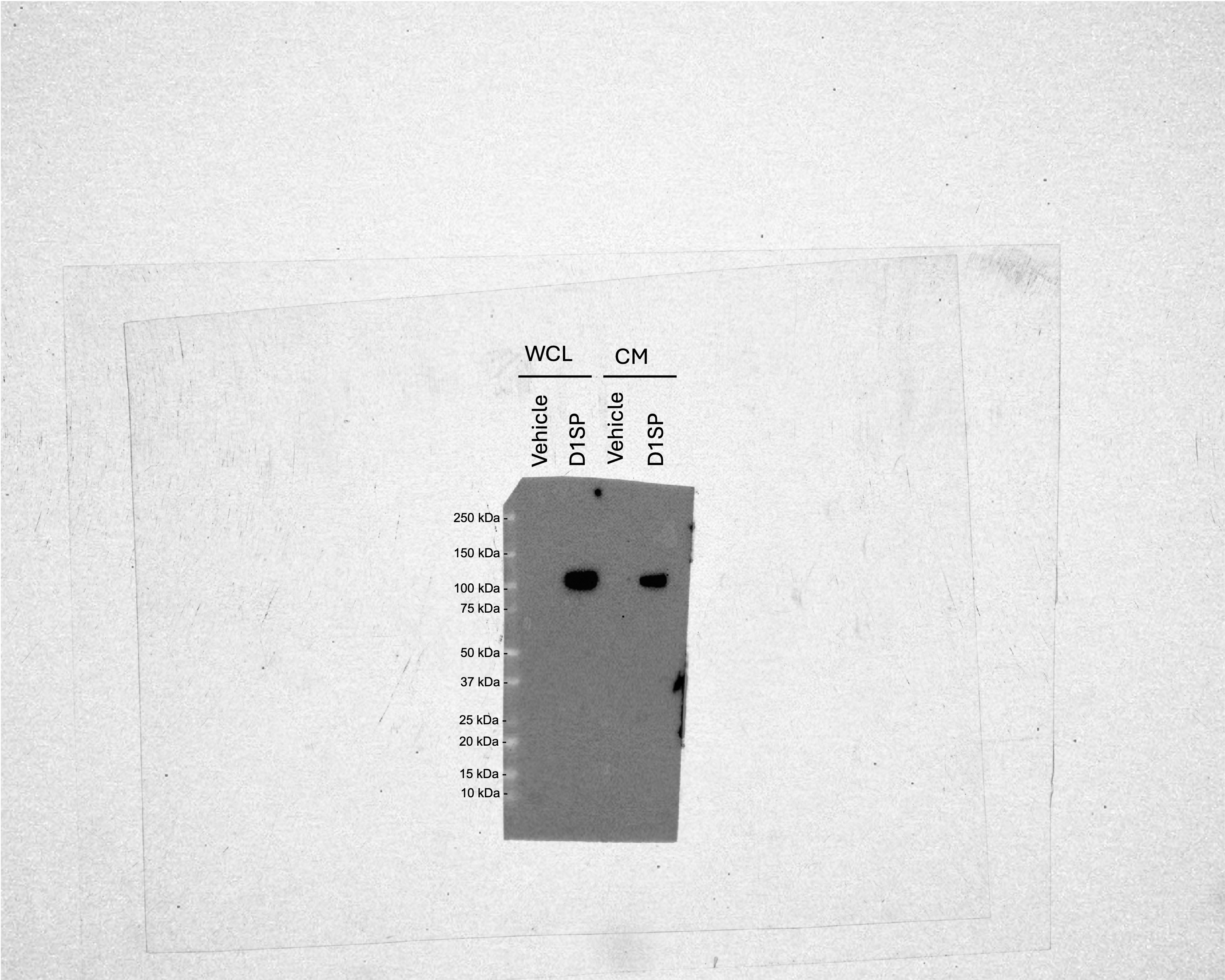

Supplement: Supplementary file 11 — Source data Fig. 9 [file 44321_2024_186_MOESM11_ESM.zip › Figure 9/9B/WB-9B.jpg]

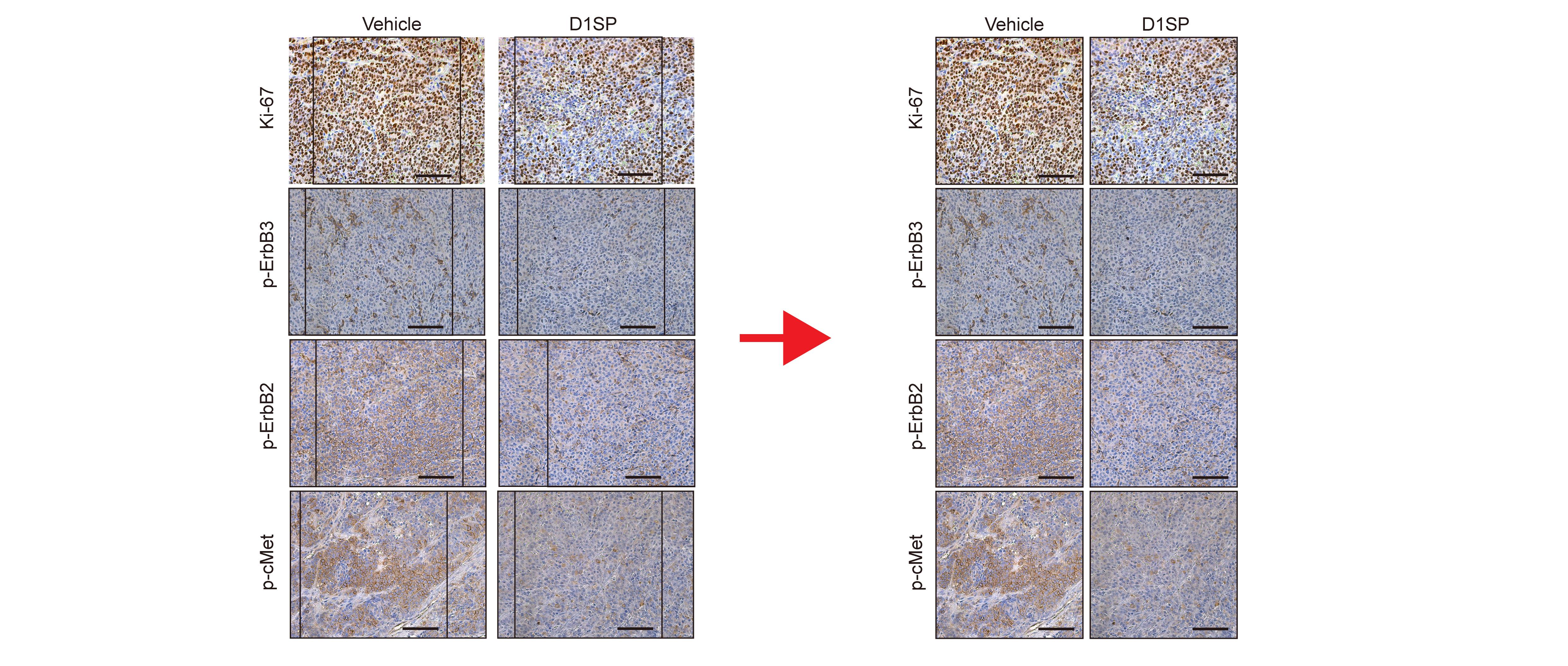

Supplement: Supplementary file 11 — Source data Fig. 9 [file 44321_2024_186_MOESM11_ESM.zip › Figure 9/9K/README.tif]

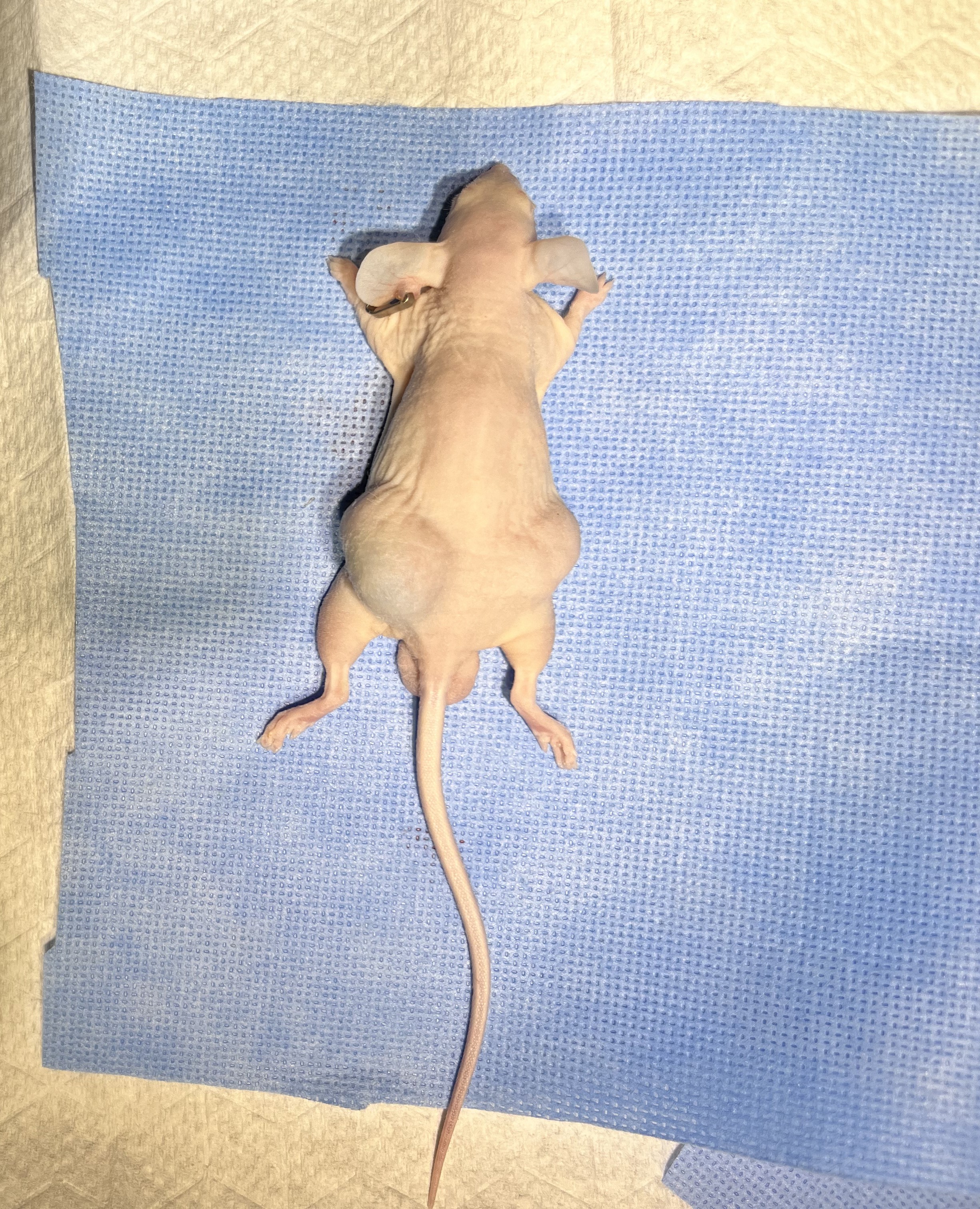

Supplement: Supplementary file 11 — Source data Fig. 9 [file 44321_2024_186_MOESM11_ESM.zip › Figure 9/9J/Mouse.jpg]

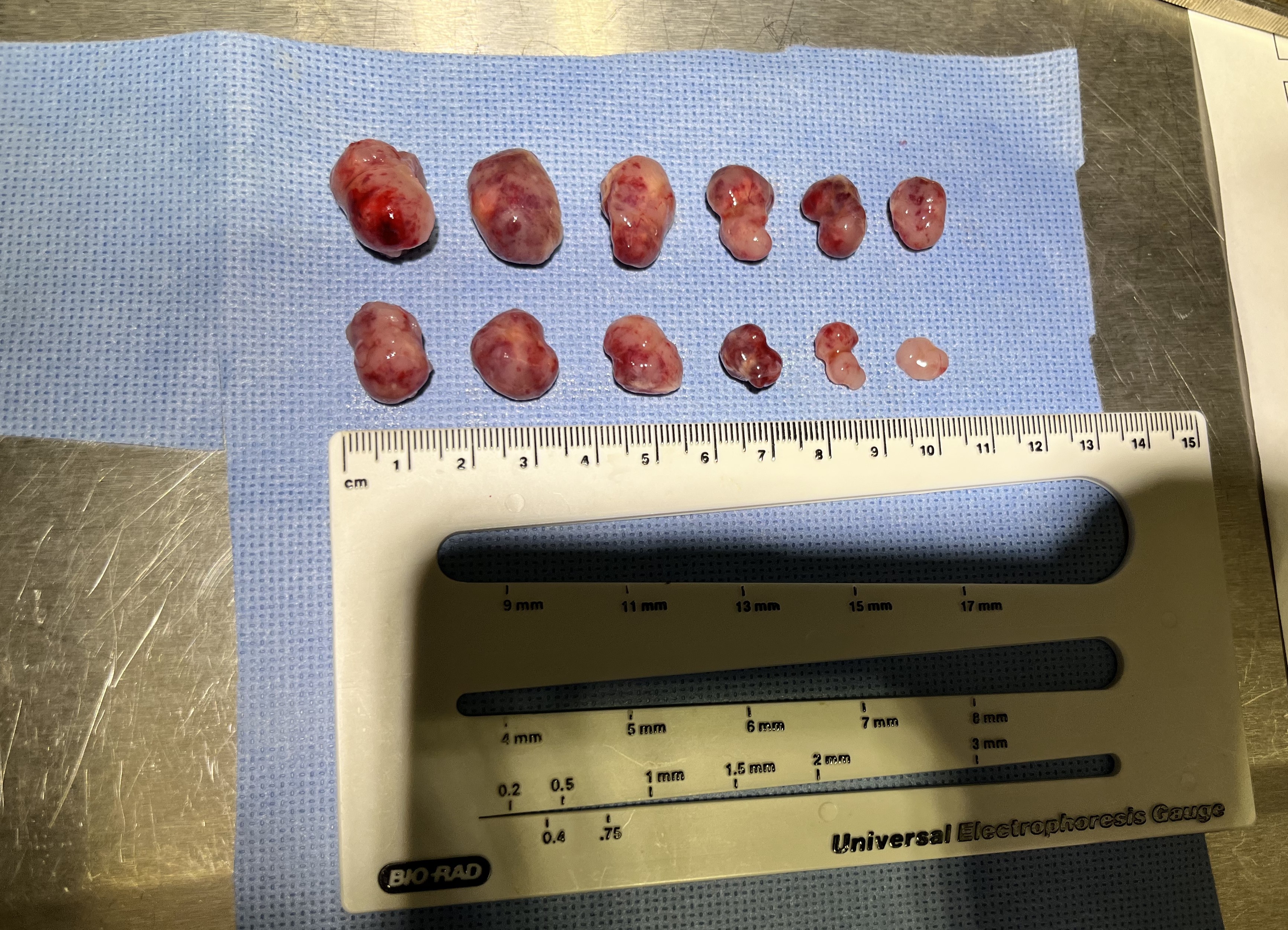

Supplement: Supplementary file 11 — Source data Fig. 9 [file 44321_2024_186_MOESM11_ESM.zip › Figure 9/9J/Tumors.jpg]

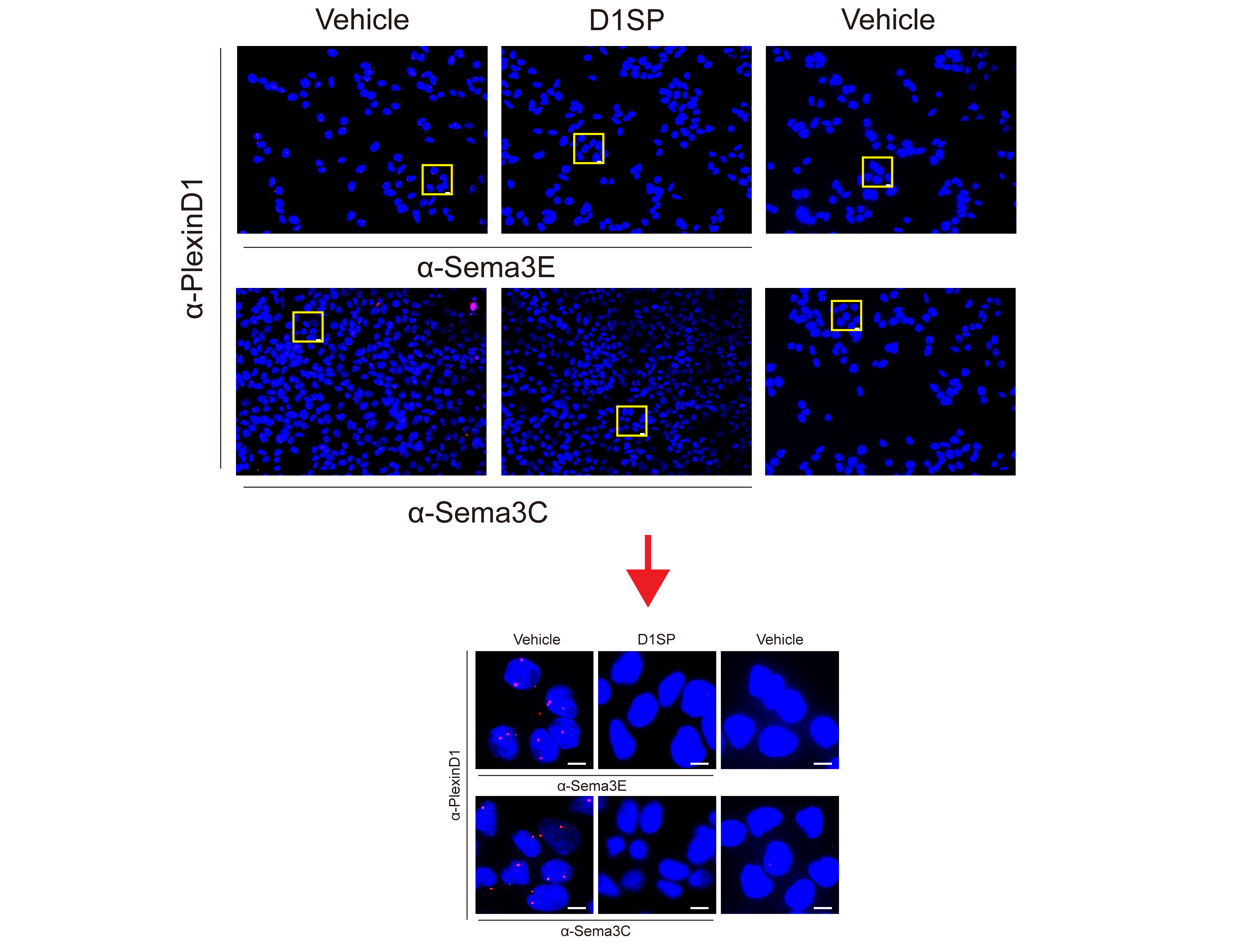

Supplement: Supplementary file 11 — Source data Fig. 9 [file 44321_2024_186_MOESM11_ESM.zip › Figure 9/9D/RAEDME.tif]

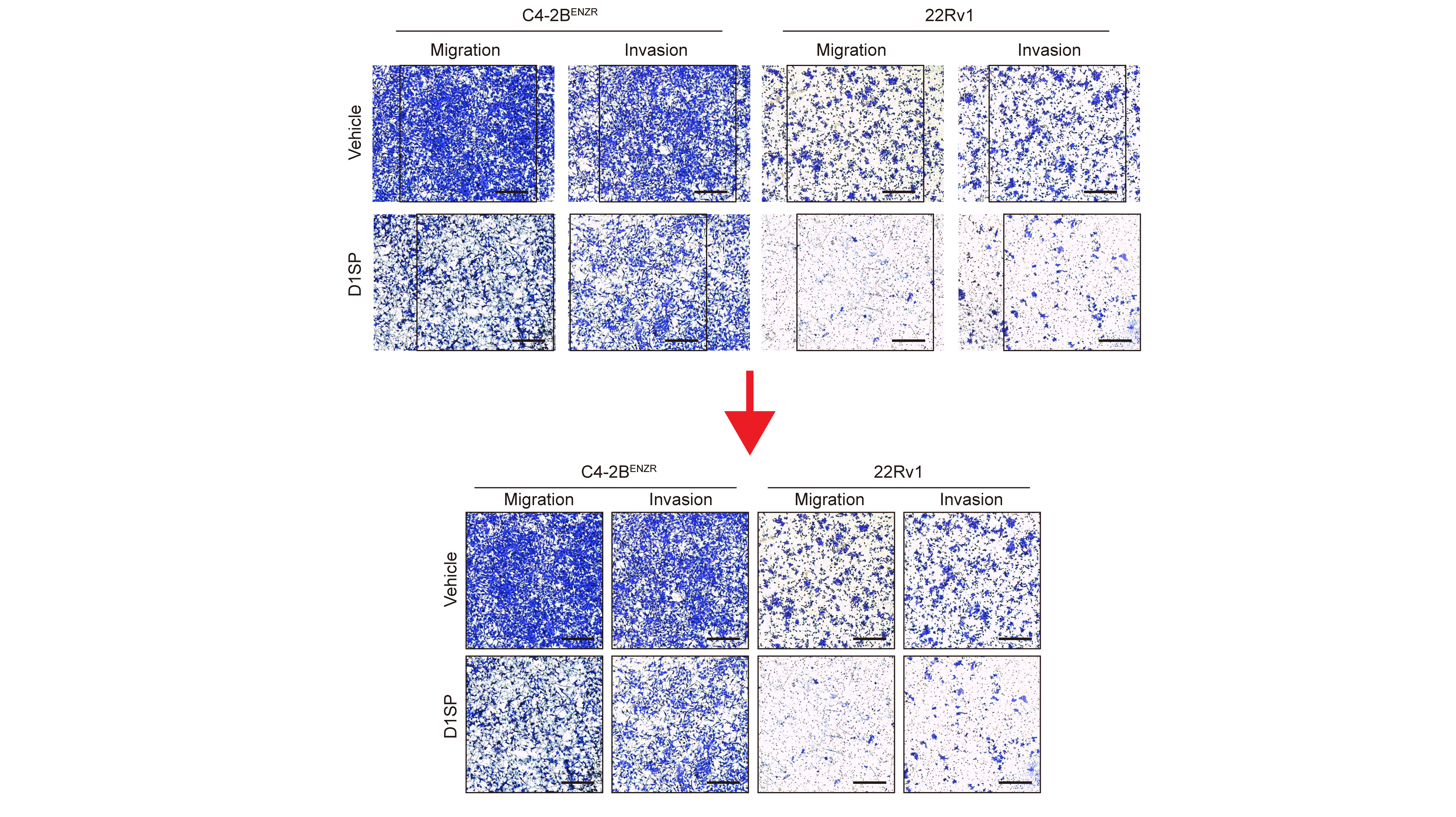

Supplement: Supplementary file 11 — Source data Fig. 9 [file 44321_2024_186_MOESM11_ESM.zip › Figure 9/9F/README.tif]

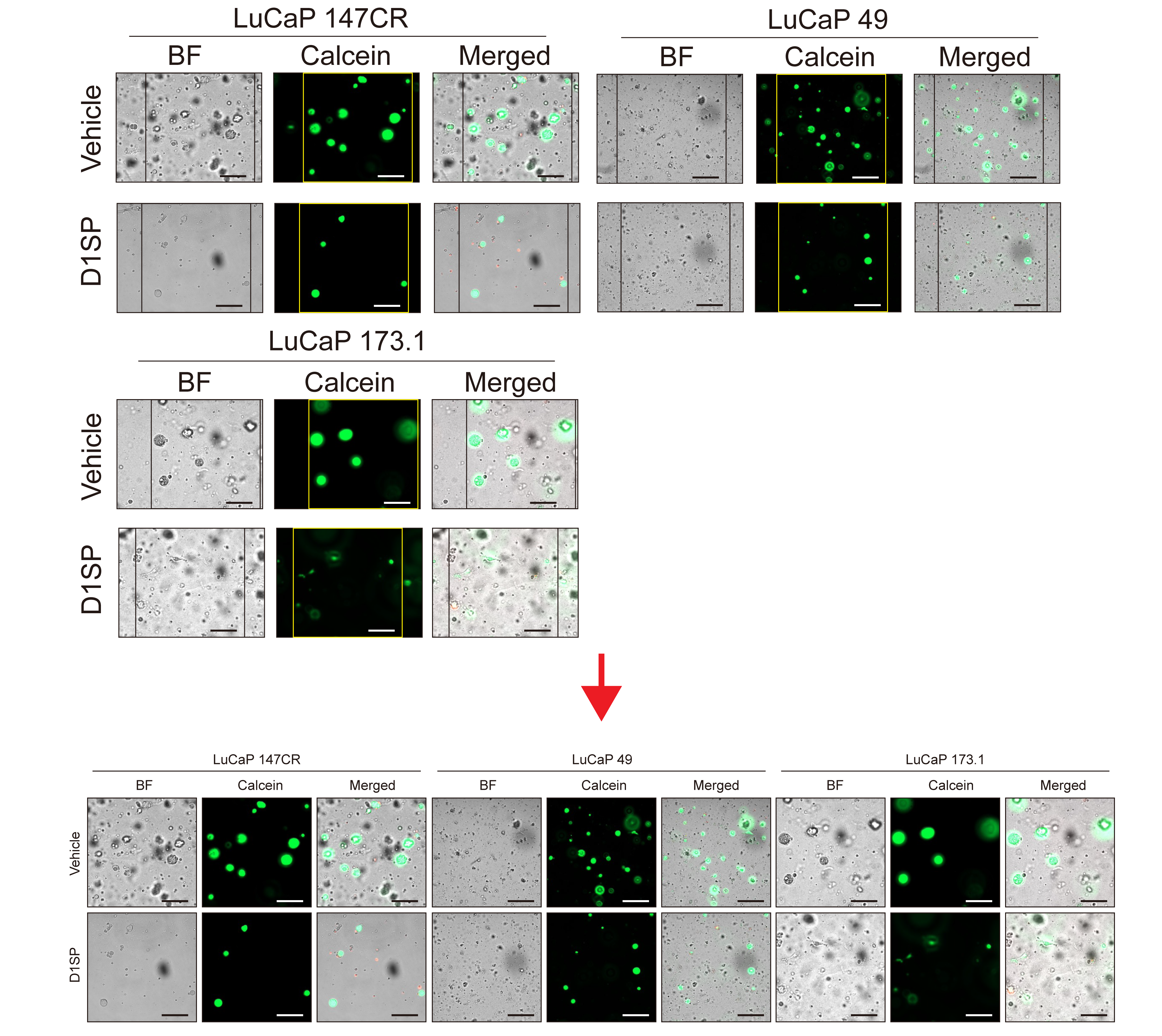

Supplement: Supplementary file 11 — Source data Fig. 9 [file 44321_2024_186_MOESM11_ESM.zip › Figure 9/9G/README.tif]

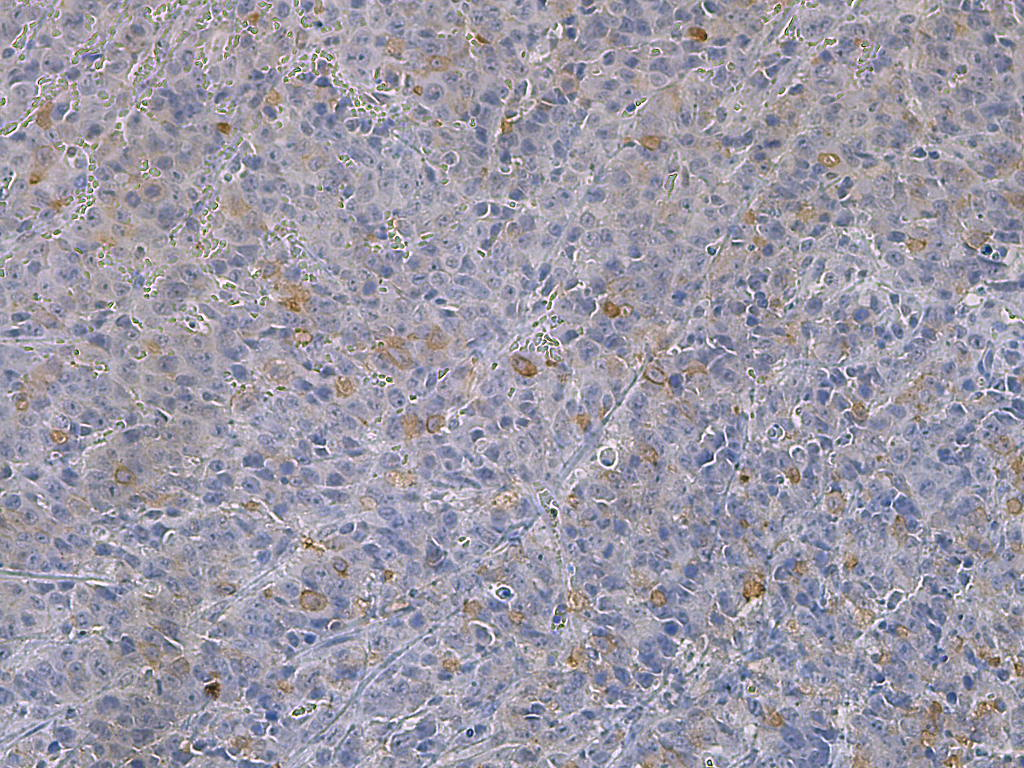

Supplement: Supplementary file 11 — Source data Fig. 9 [file 44321_2024_186_MOESM11_ESM.zip › Figure 9/9K/p-cMet/D1SP.tif]

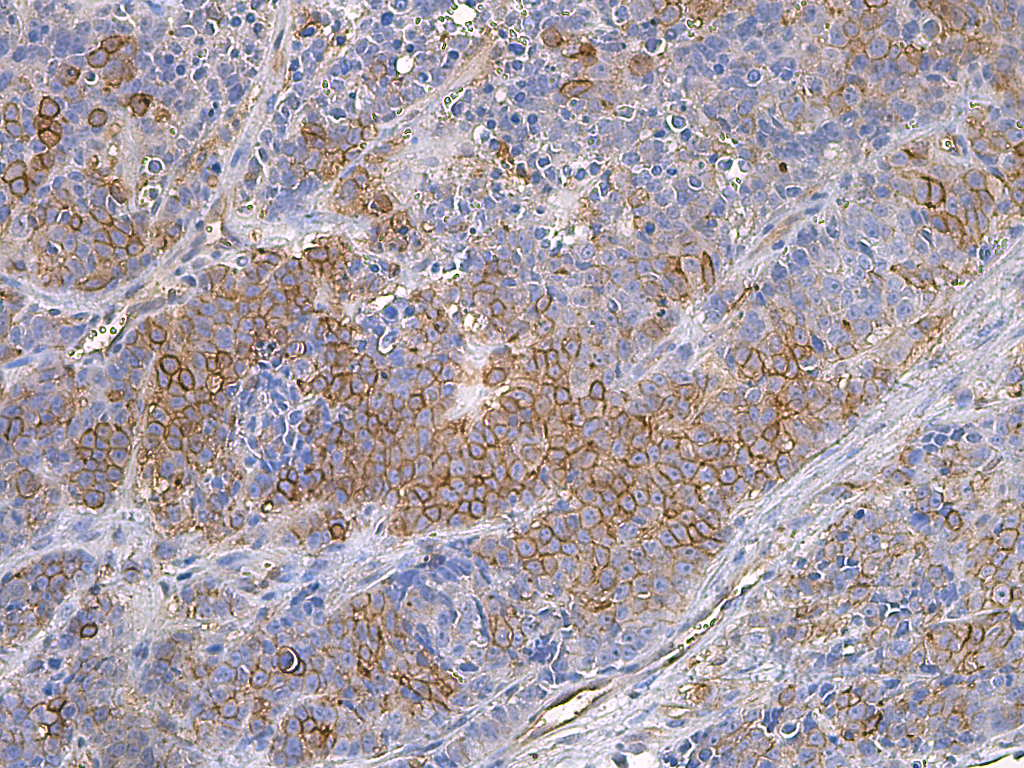

Supplement: Supplementary file 11 — Source data Fig. 9 [file 44321_2024_186_MOESM11_ESM.zip › Figure 9/9K/p-cMet/Vehicle.tif]

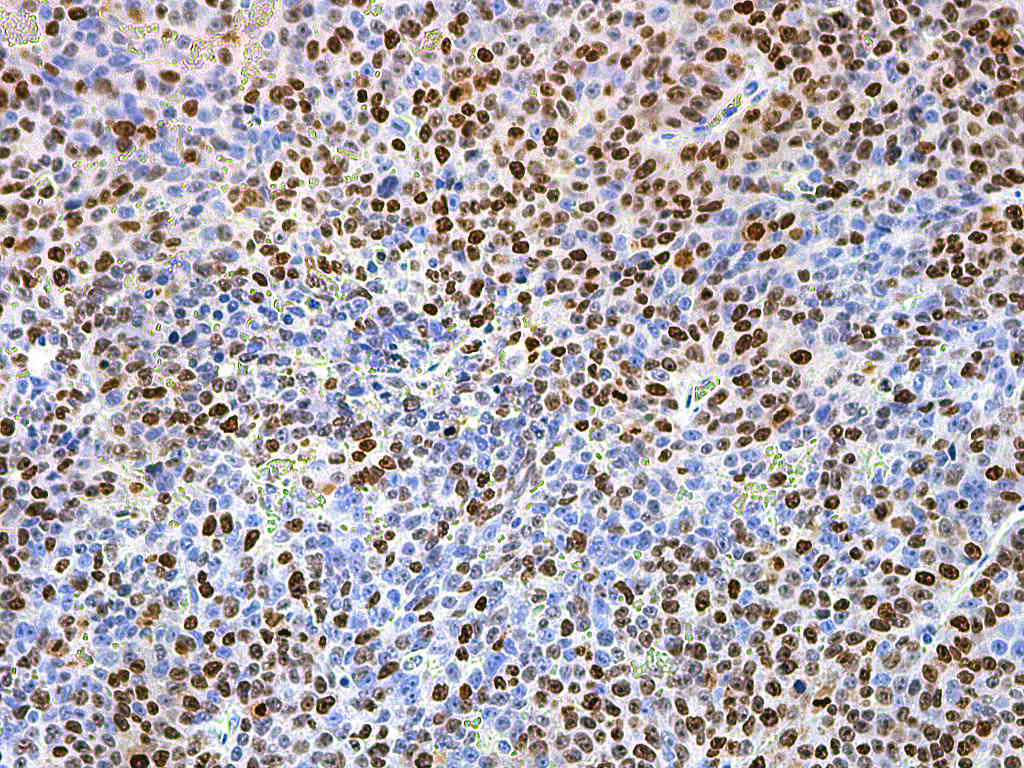

Supplement: Supplementary file 11 — Source data Fig. 9 [file 44321_2024_186_MOESM11_ESM.zip › Figure 9/9K/Ki-67/D1SP.tif]

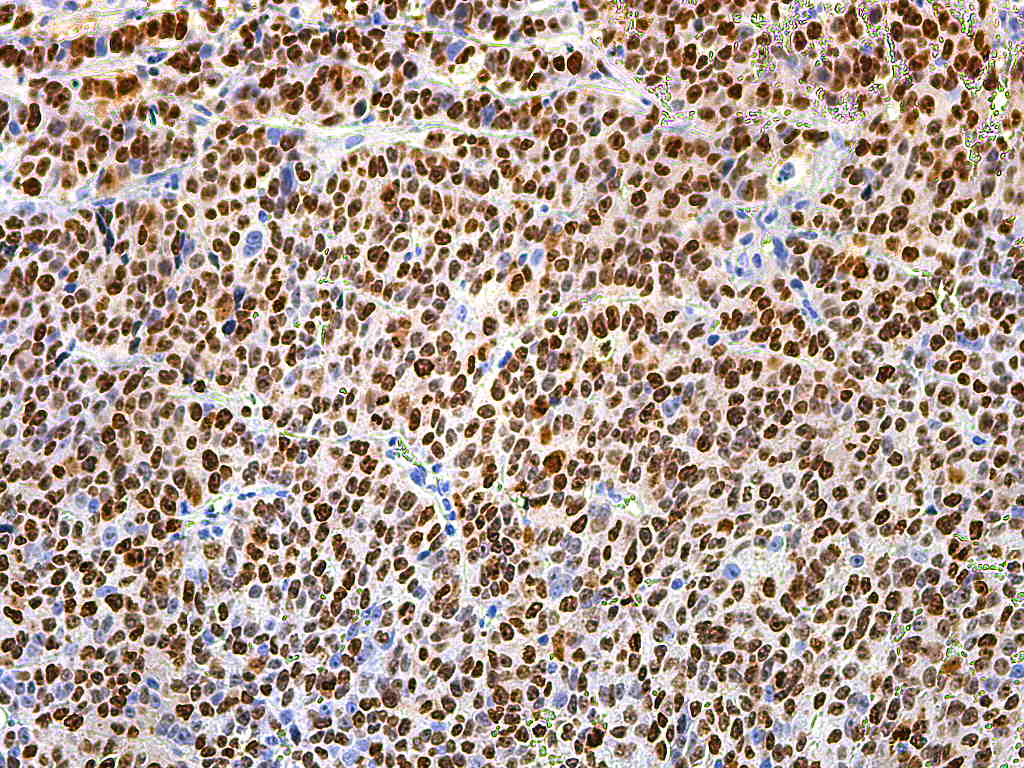

Supplement: Supplementary file 11 — Source data Fig. 9 [file 44321_2024_186_MOESM11_ESM.zip › Figure 9/9K/Ki-67/Vehicle.tif]

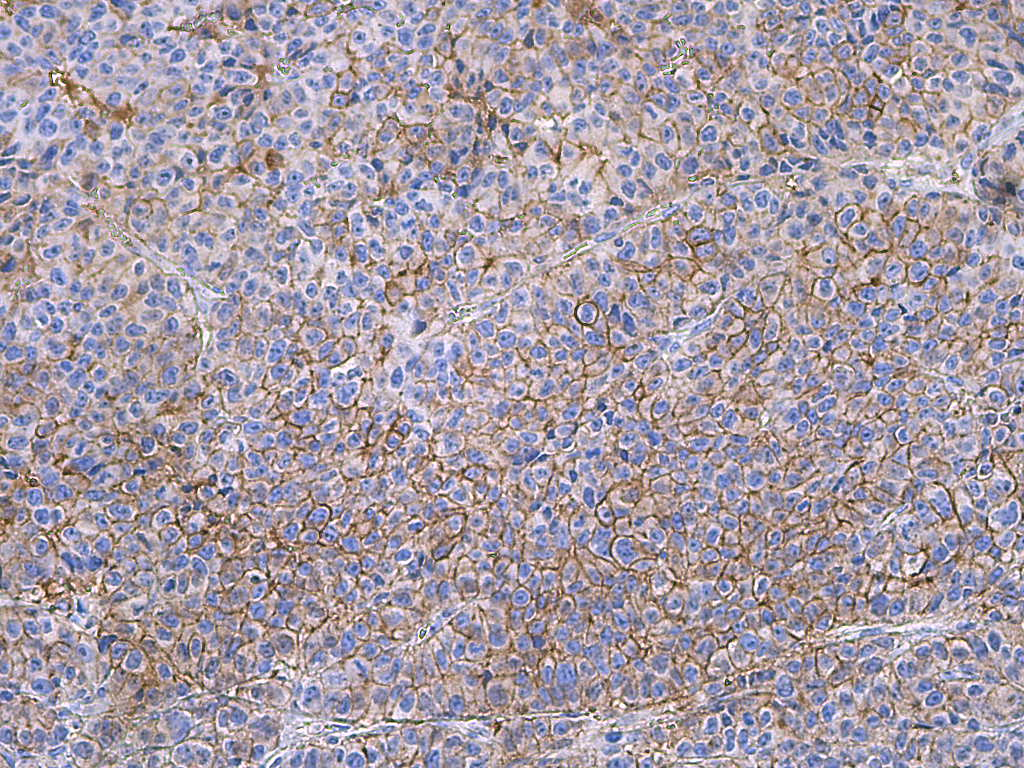

Supplement: Supplementary file 11 — Source data Fig. 9 [file 44321_2024_186_MOESM11_ESM.zip › Figure 9/9K/p-ErbB2/Vehicle_pErbB2.tif]

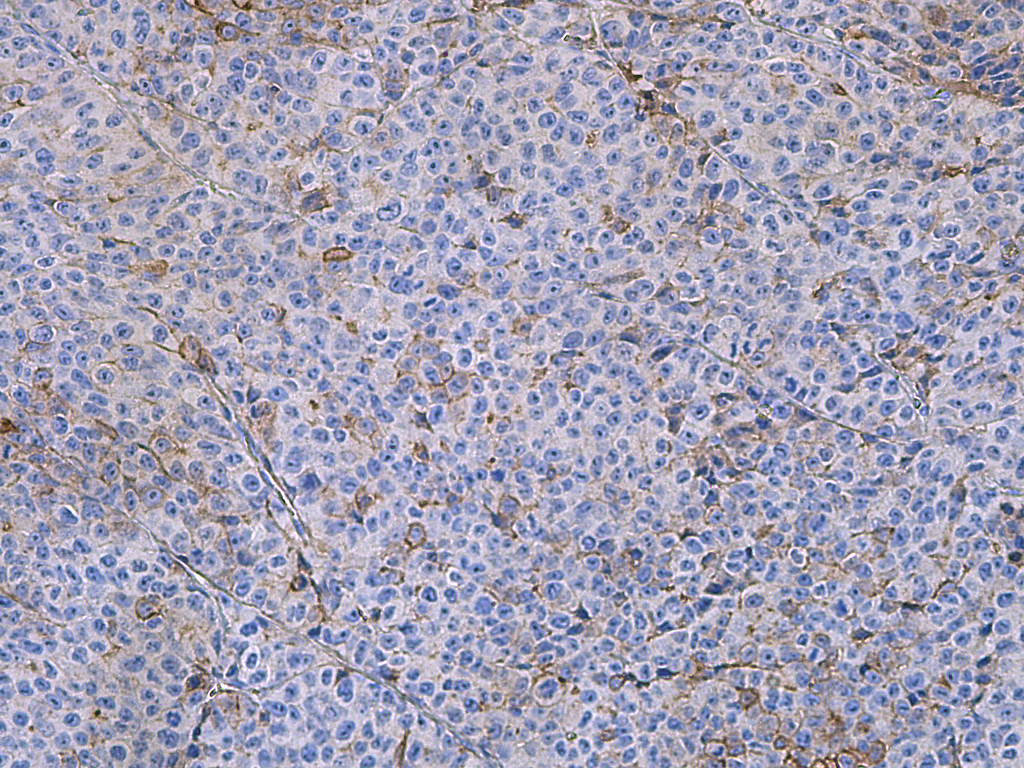

Supplement: Supplementary file 11 — Source data Fig. 9 [file 44321_2024_186_MOESM11_ESM.zip › Figure 9/9K/p-ErbB2/D1SP_pErbB2.tif]

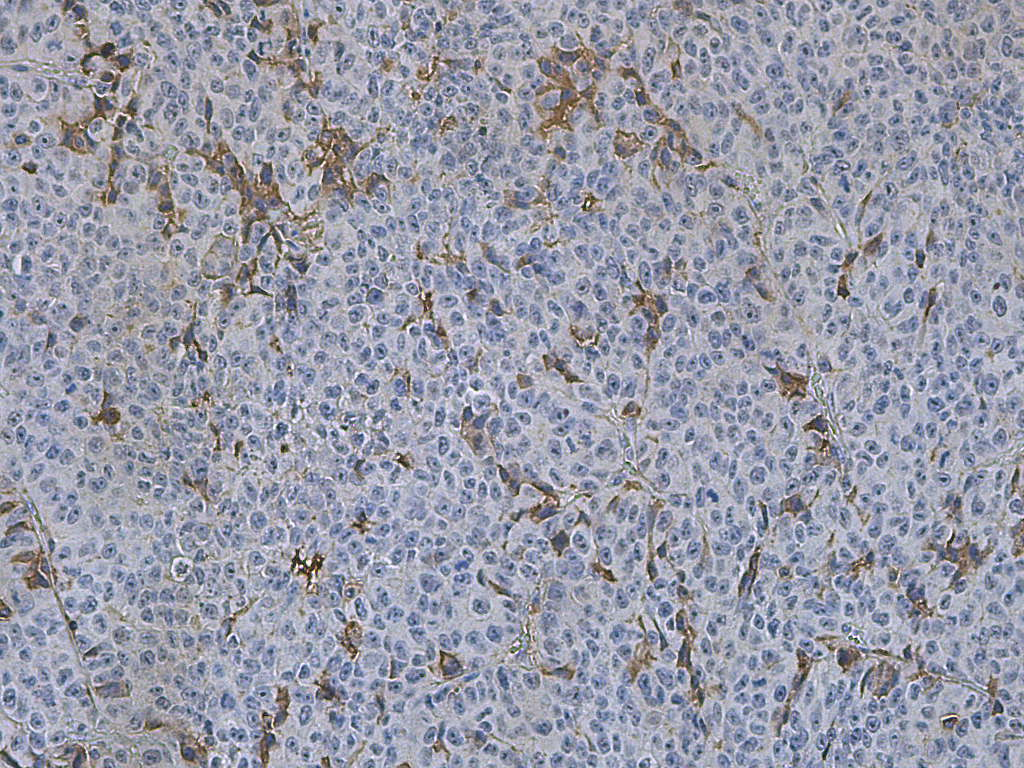

Supplement: Supplementary file 11 — Source data Fig. 9 [file 44321_2024_186_MOESM11_ESM.zip › Figure 9/9K/p-ErbB3/Vehicle-pErbB3.tif]

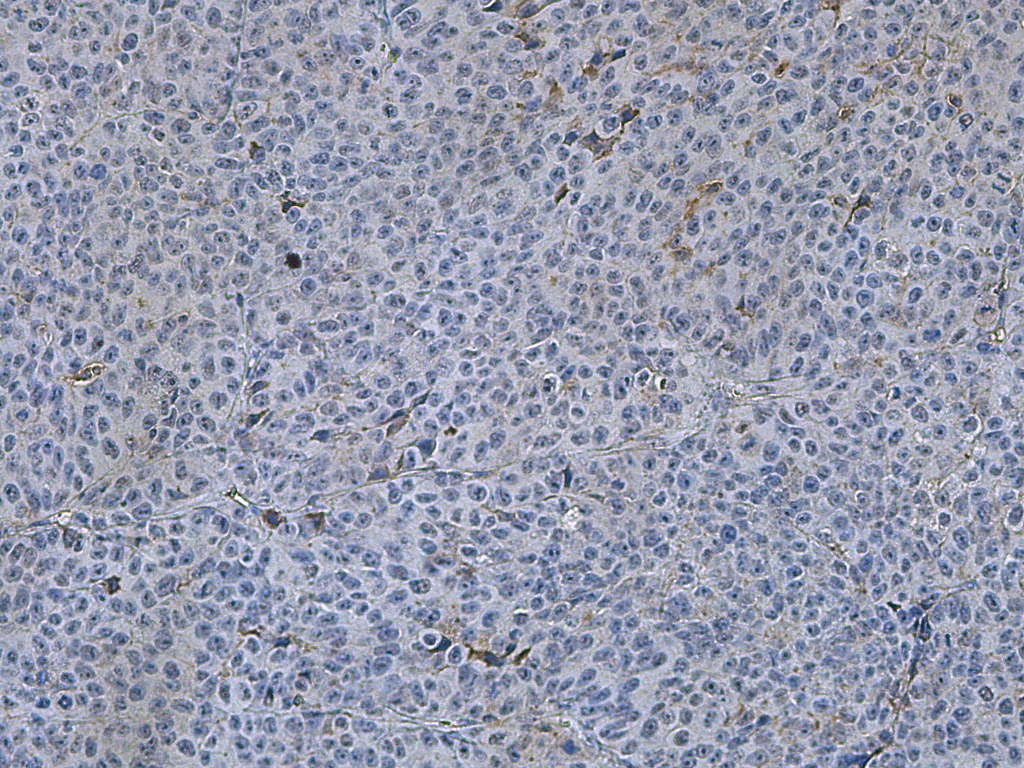

Supplement: Supplementary file 11 — Source data Fig. 9 [file 44321_2024_186_MOESM11_ESM.zip › Figure 9/9K/p-ErbB3/D1SP-pErbB3.tif]

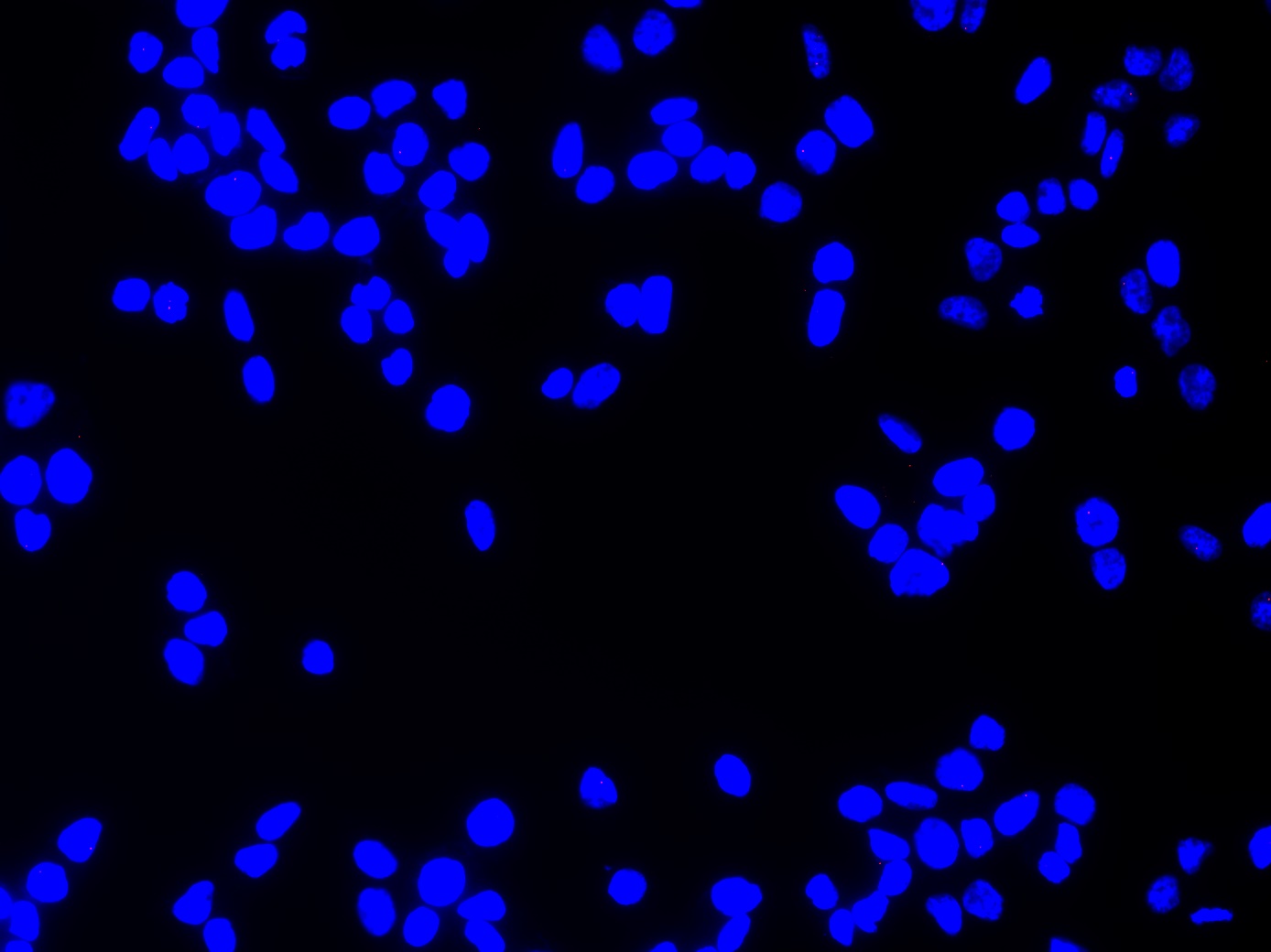

Supplement: Supplementary file 11 — Source data Fig. 9 [file 44321_2024_186_MOESM11_ESM.zip › Figure 9/9D/PlexinD1-Sema3C/Vehicle_neg.jpg]

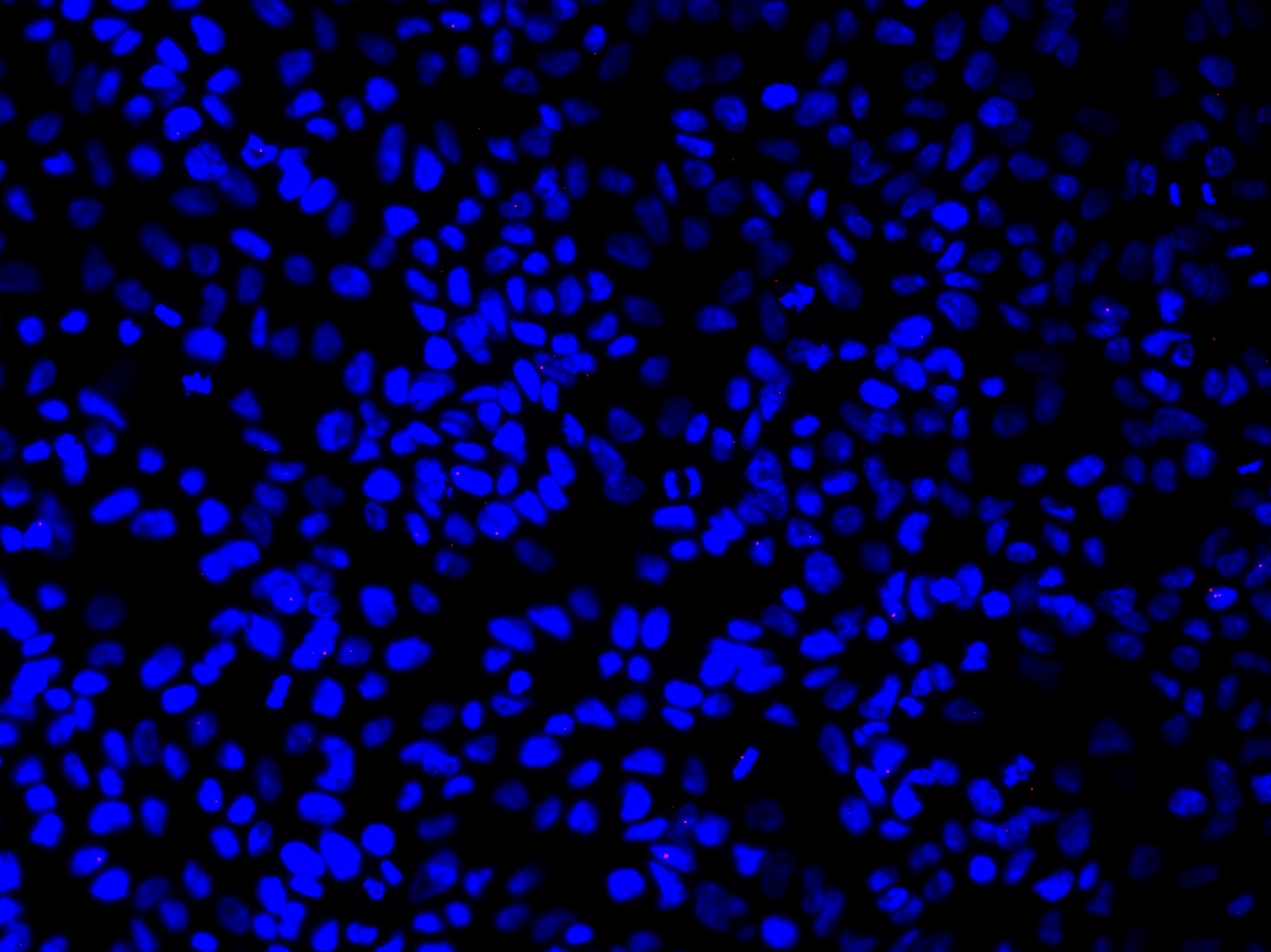

Supplement: Supplementary file 11 — Source data Fig. 9 [file 44321_2024_186_MOESM11_ESM.zip › Figure 9/9D/PlexinD1-Sema3C/D1SP.jpg]

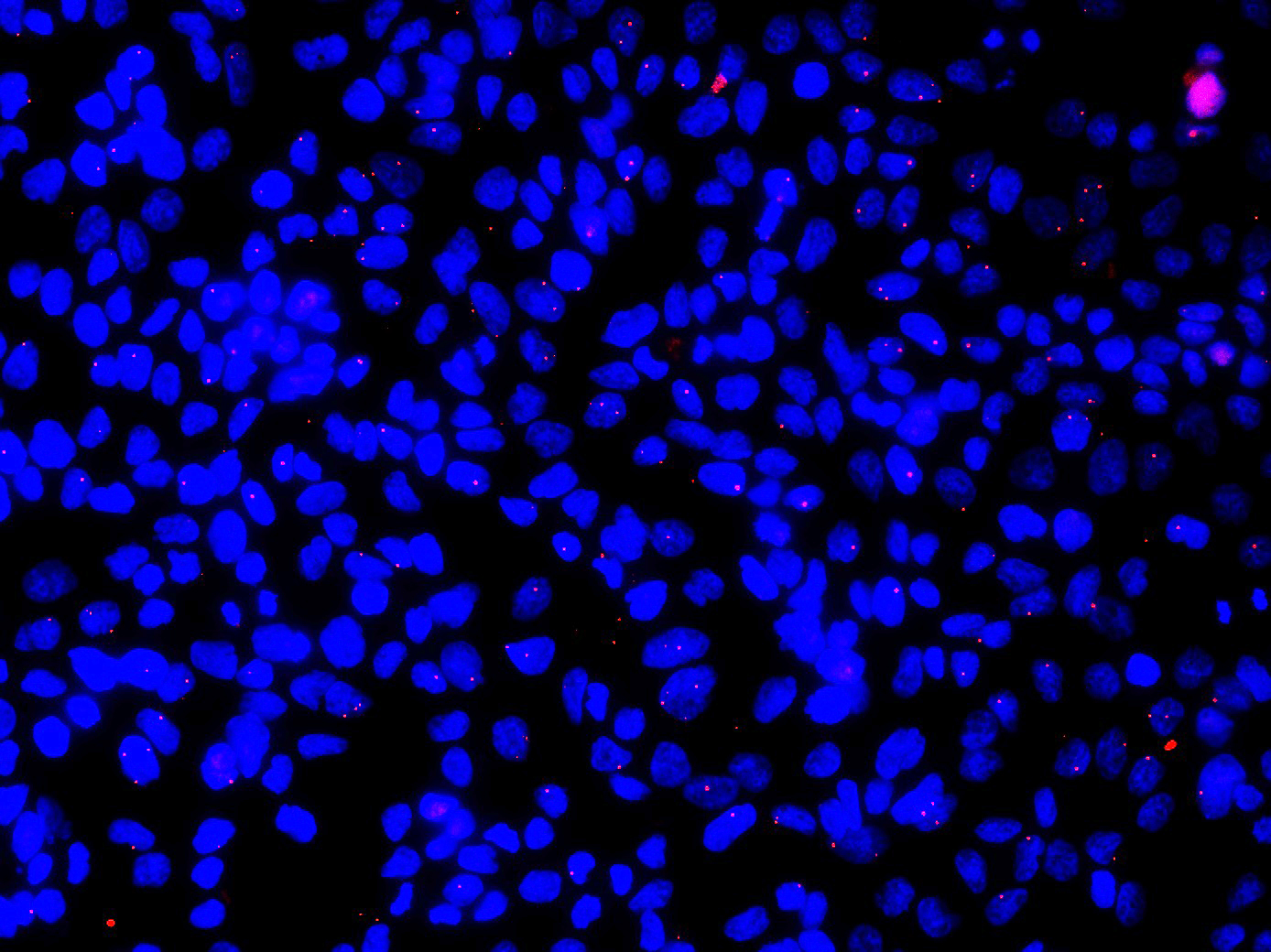

Supplement: Supplementary file 11 — Source data Fig. 9 [file 44321_2024_186_MOESM11_ESM.zip › Figure 9/9D/PlexinD1-Sema3C/Vehicle.jpg]

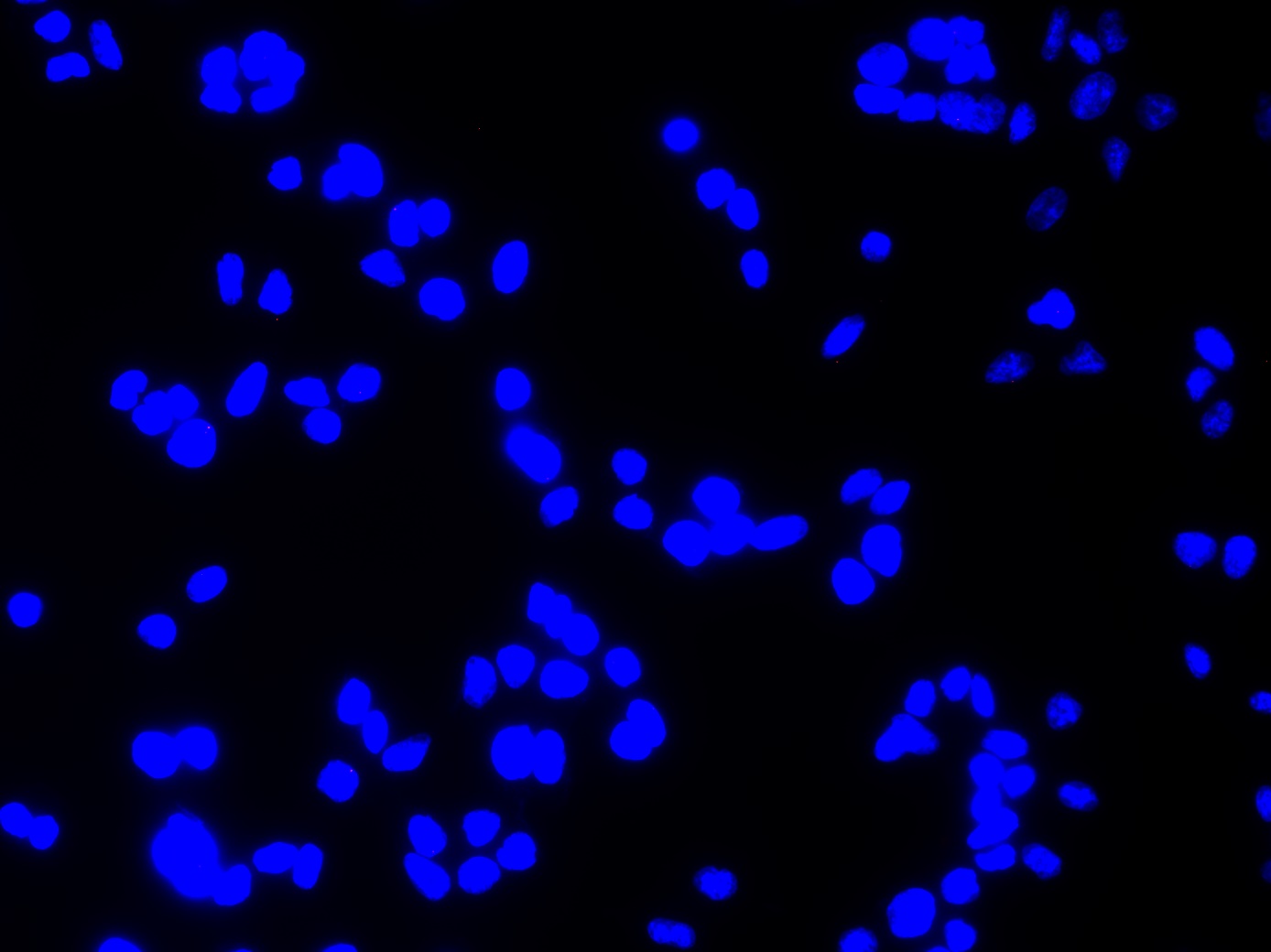

Supplement: Supplementary file 11 — Source data Fig. 9 [file 44321_2024_186_MOESM11_ESM.zip › Figure 9/9D/PlexinD1-Sema3E/Vehicle_neg.jpg]

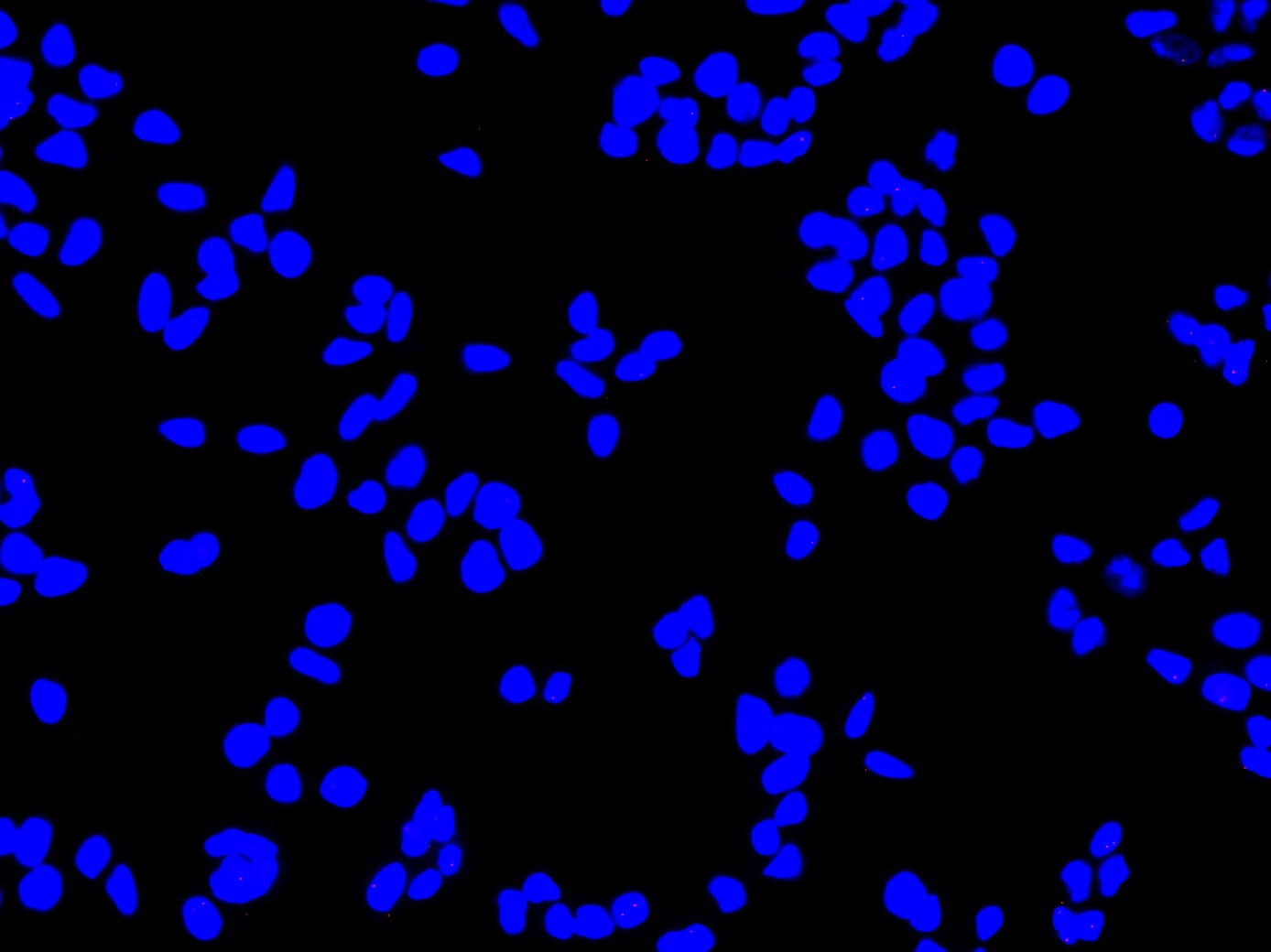

Supplement: Supplementary file 11 — Source data Fig. 9 [file 44321_2024_186_MOESM11_ESM.zip › Figure 9/9D/PlexinD1-Sema3E/D1SP.jpg]

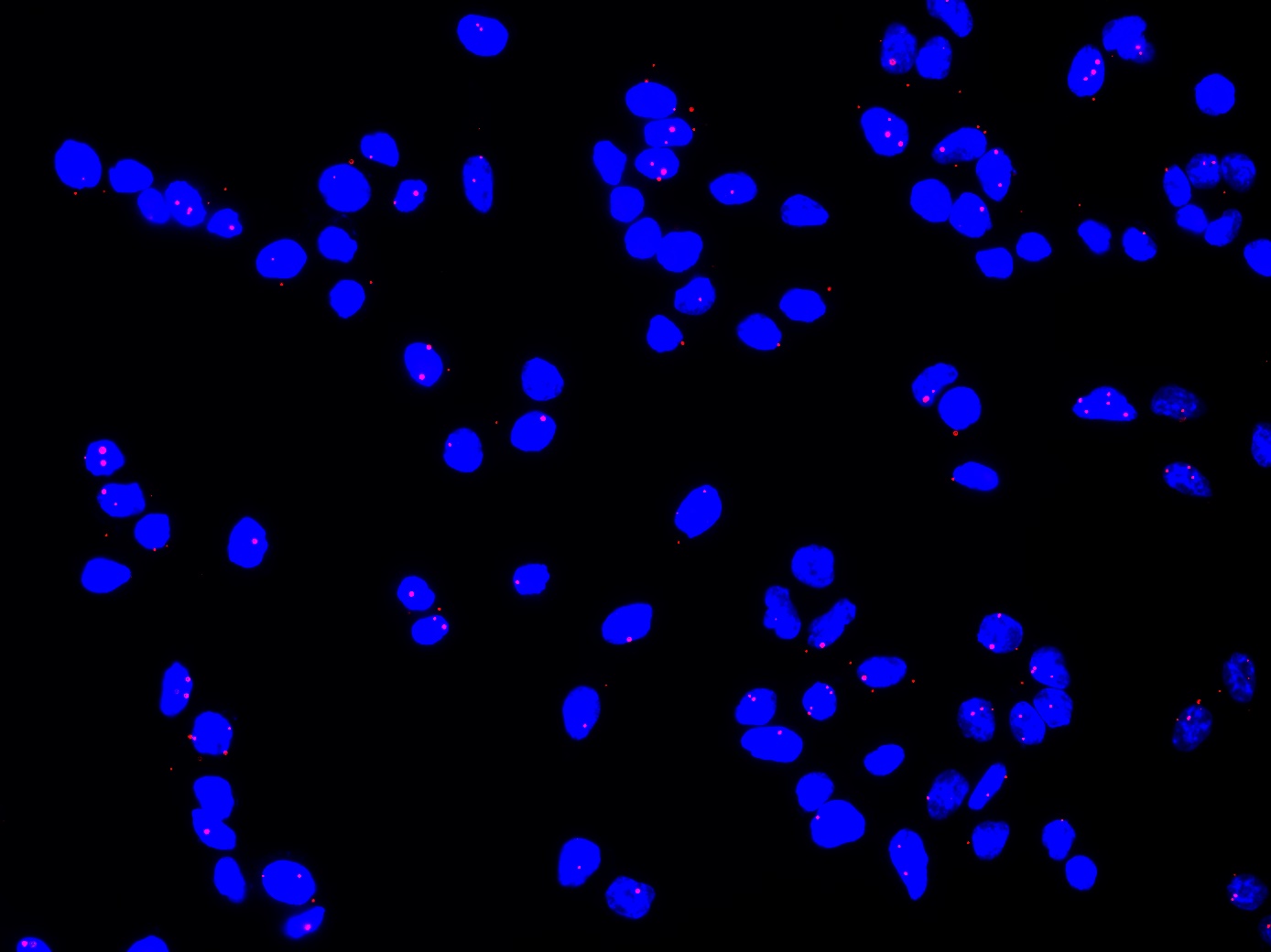

Supplement: Supplementary file 11 — Source data Fig. 9 [file 44321_2024_186_MOESM11_ESM.zip › Figure 9/9D/PlexinD1-Sema3E/Vehicle.jpg]

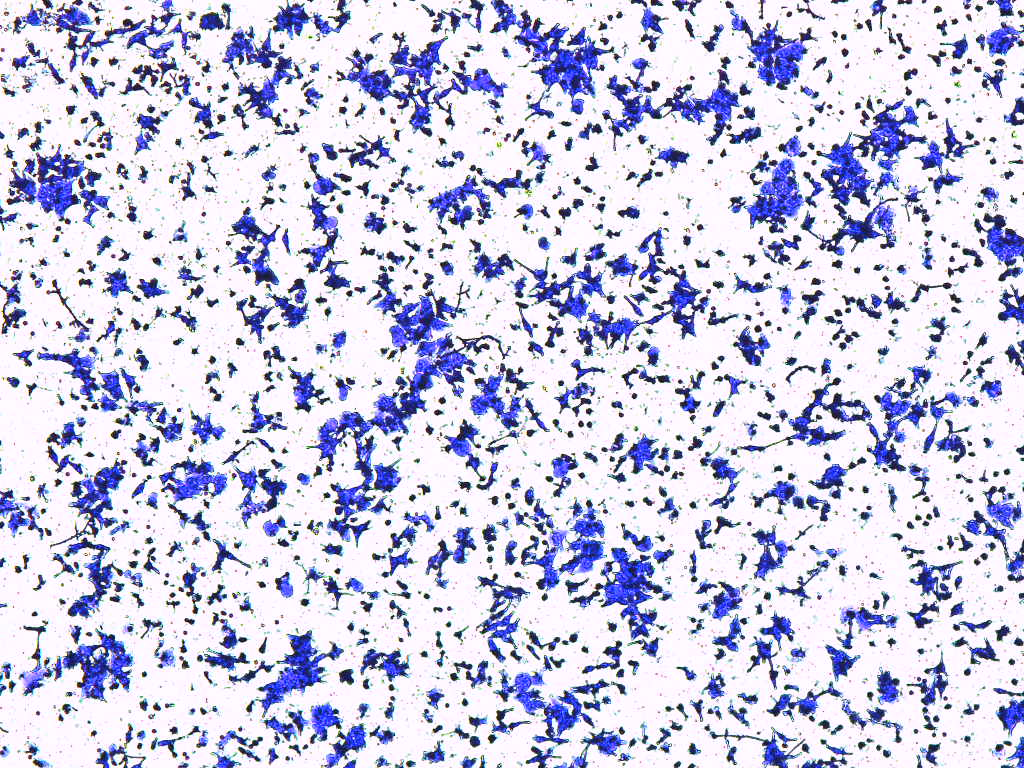

Supplement: Supplementary file 11 — Source data Fig. 9 [file 44321_2024_186_MOESM11_ESM.zip › Figure 9/9F/Invasion/22Rv1_Vehicle.tif]

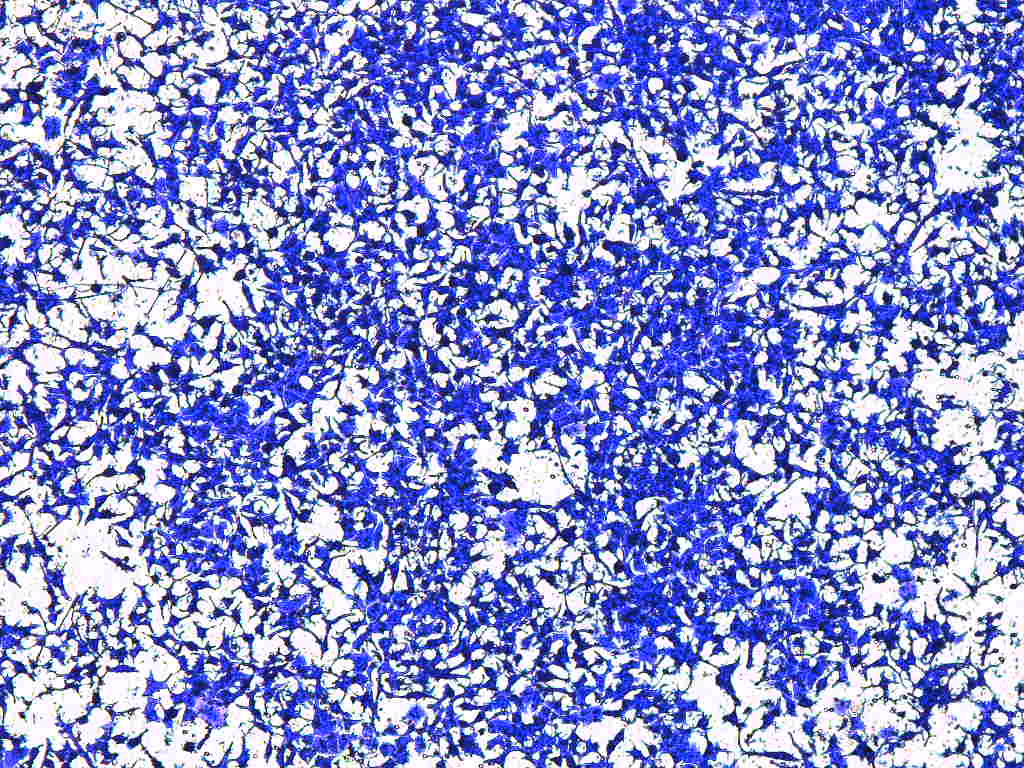

Supplement: Supplementary file 11 — Source data Fig. 9 [file 44321_2024_186_MOESM11_ESM.zip › Figure 9/9F/Invasion/C4-2BENZR_vehicle.tif]

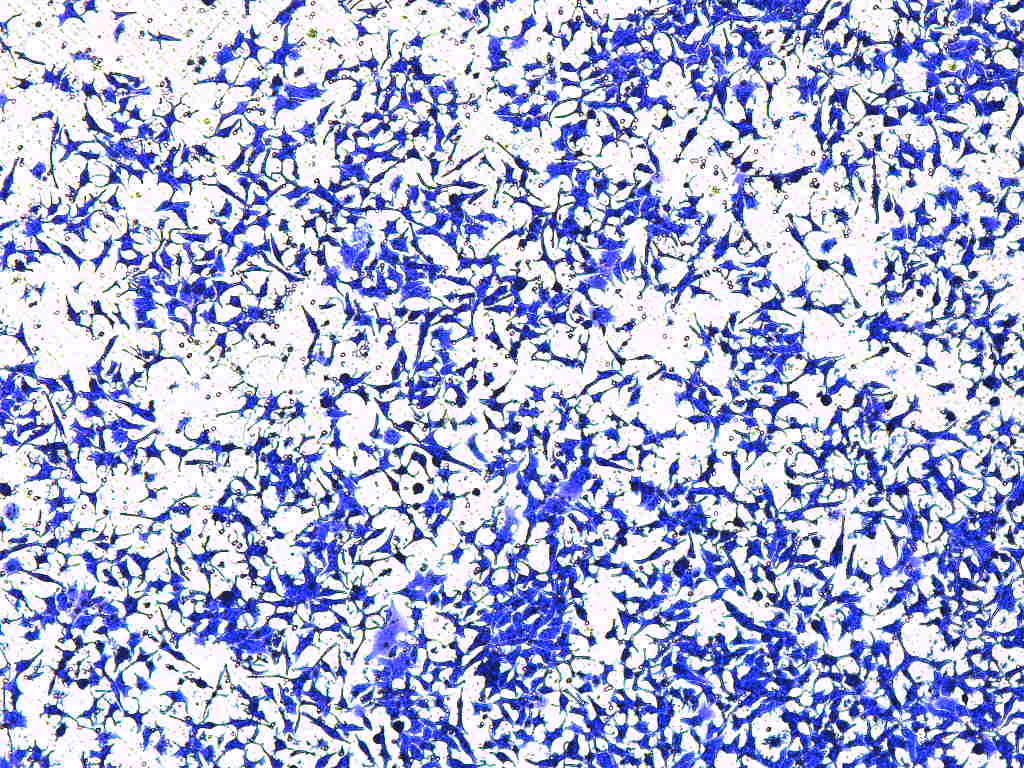

Supplement: Supplementary file 11 — Source data Fig. 9 [file 44321_2024_186_MOESM11_ESM.zip › Figure 9/9F/Invasion/C4-2BENZR_D1SP.tif]

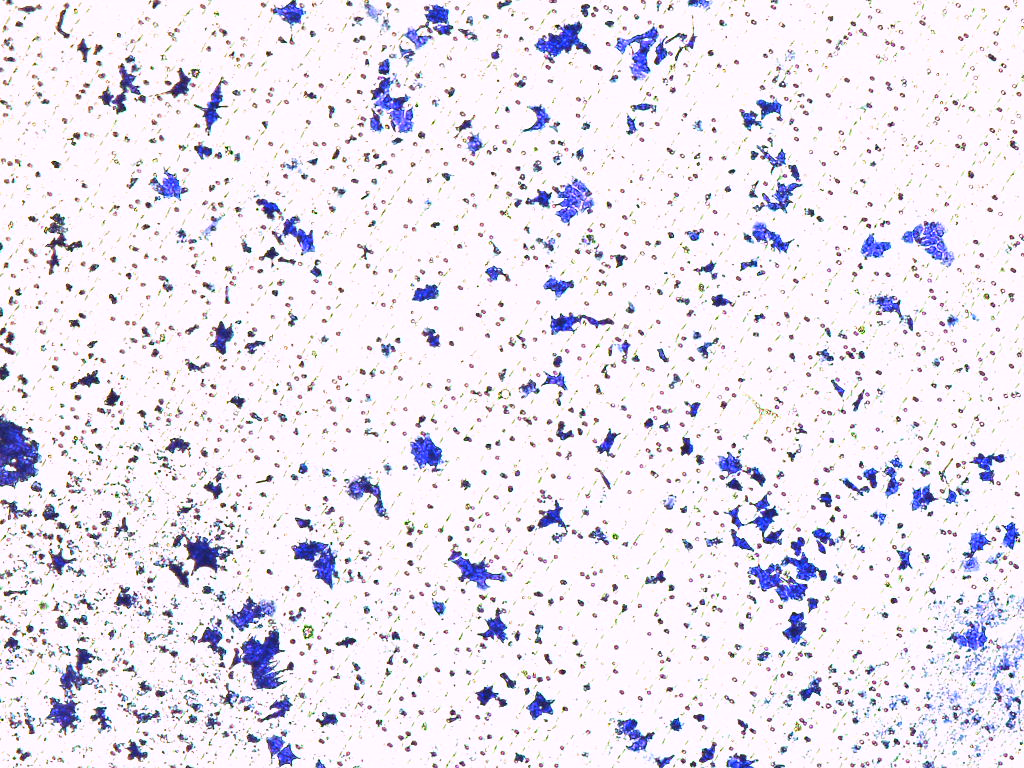

Supplement: Supplementary file 11 — Source data Fig. 9 [file 44321_2024_186_MOESM11_ESM.zip › Figure 9/9F/Invasion/22Rv1_D1SP.tif]

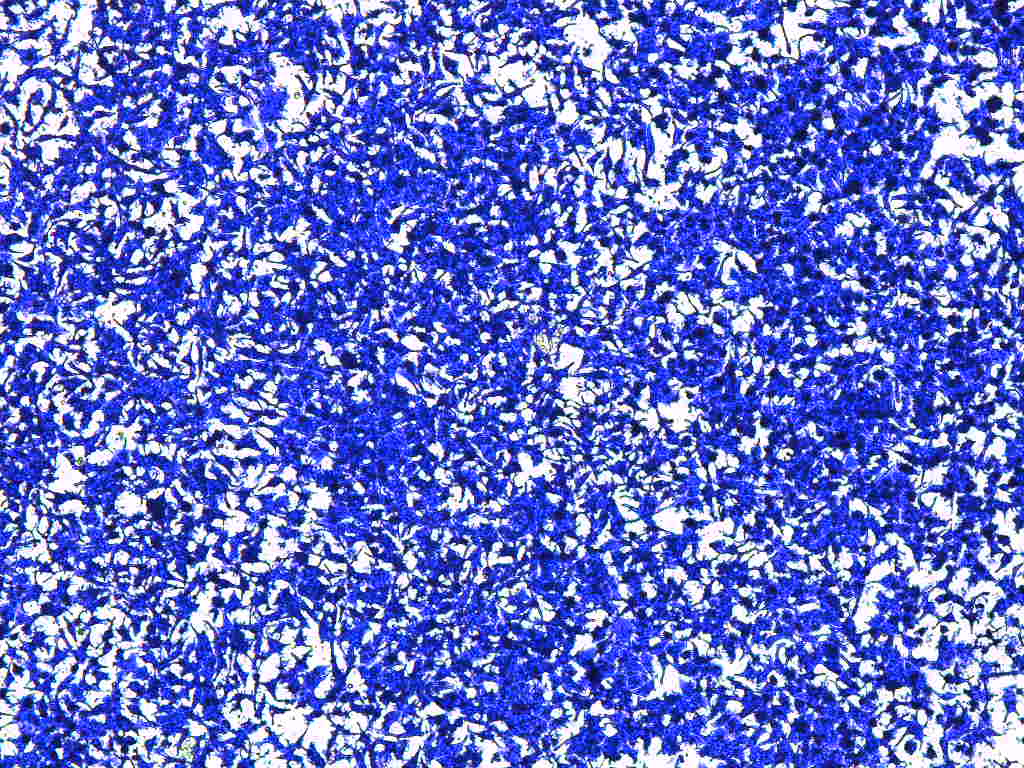

Supplement: Supplementary file 11 — Source data Fig. 9 [file 44321_2024_186_MOESM11_ESM.zip › Figure 9/9F/Migration/C4-2B ENZR_vehicle.tif]

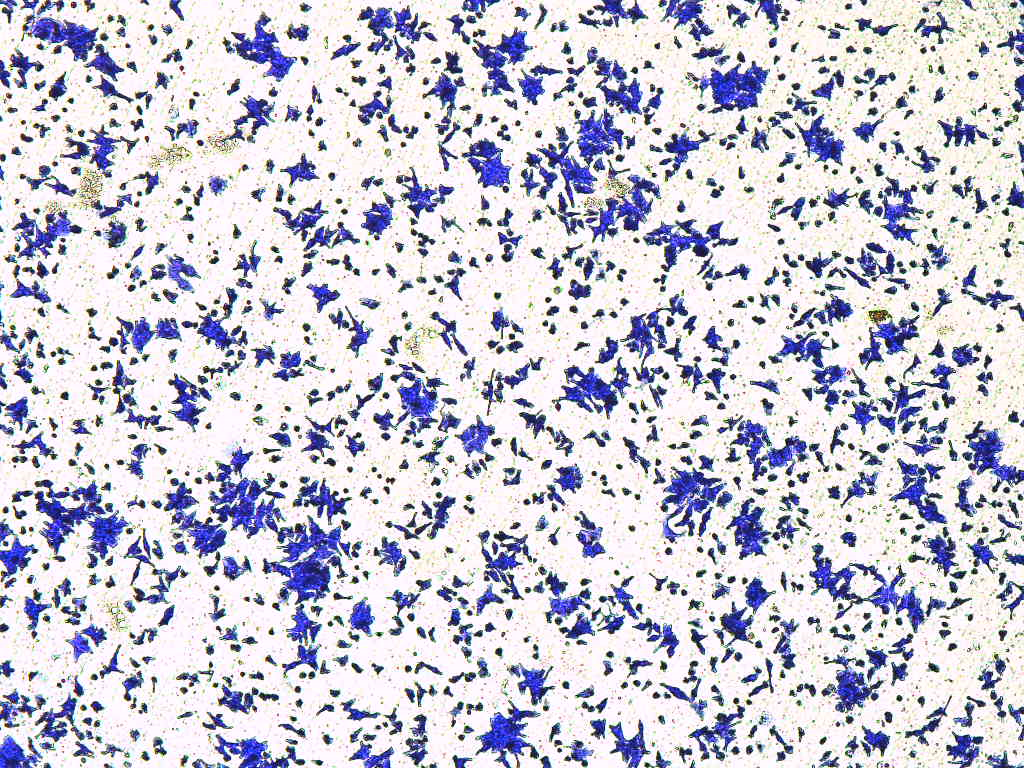

Supplement: Supplementary file 11 — Source data Fig. 9 [file 44321_2024_186_MOESM11_ESM.zip › Figure 9/9F/Migration/22Rv1_vehicle.tif]

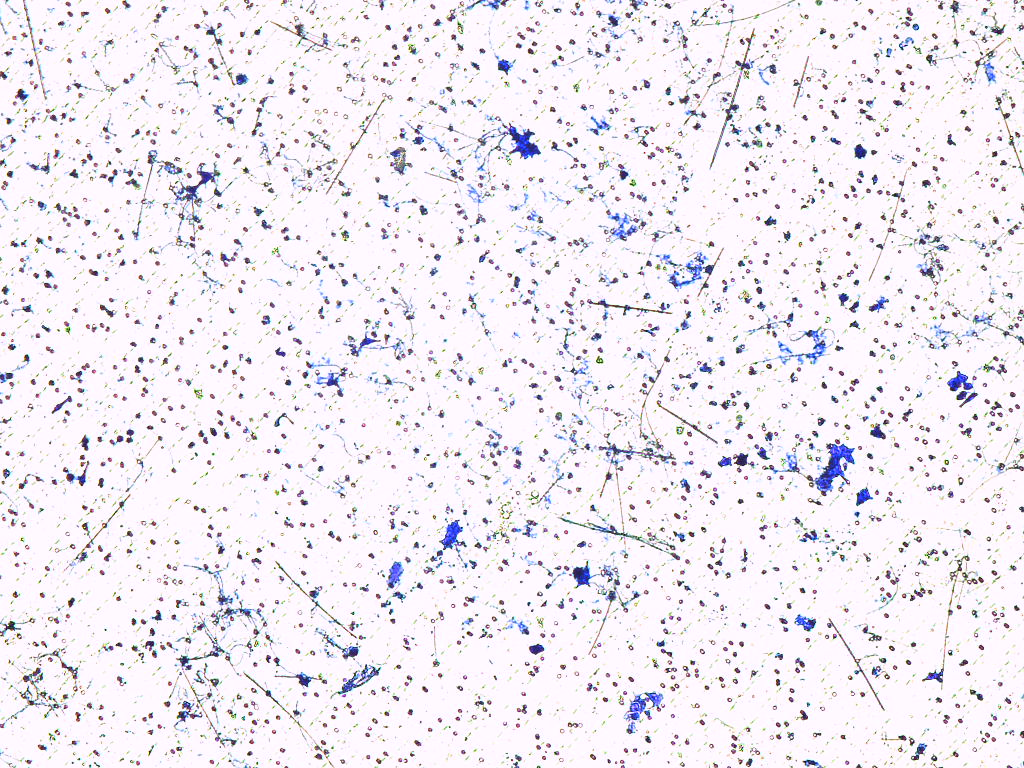

Supplement: Supplementary file 11 — Source data Fig. 9 [file 44321_2024_186_MOESM11_ESM.zip › Figure 9/9F/Migration/22Rv1_D1SP.tif]

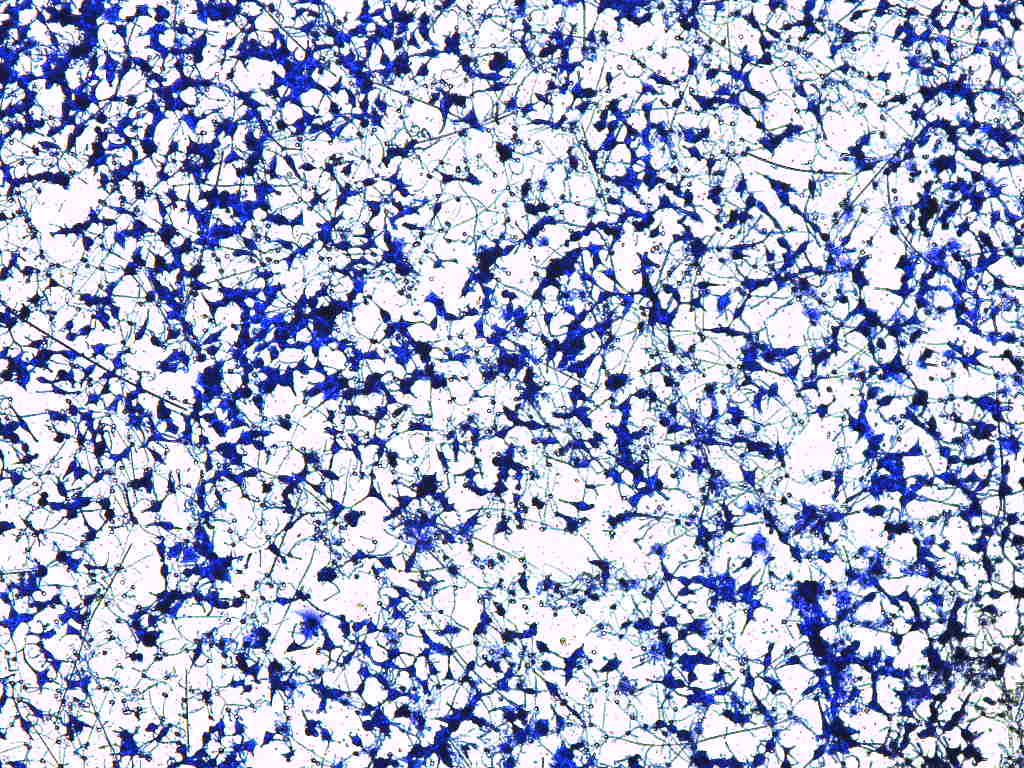

Supplement: Supplementary file 11 — Source data Fig. 9 [file 44321_2024_186_MOESM11_ESM.zip › Figure 9/9F/Migration/C4-2B ENZR_D1SP.tif]

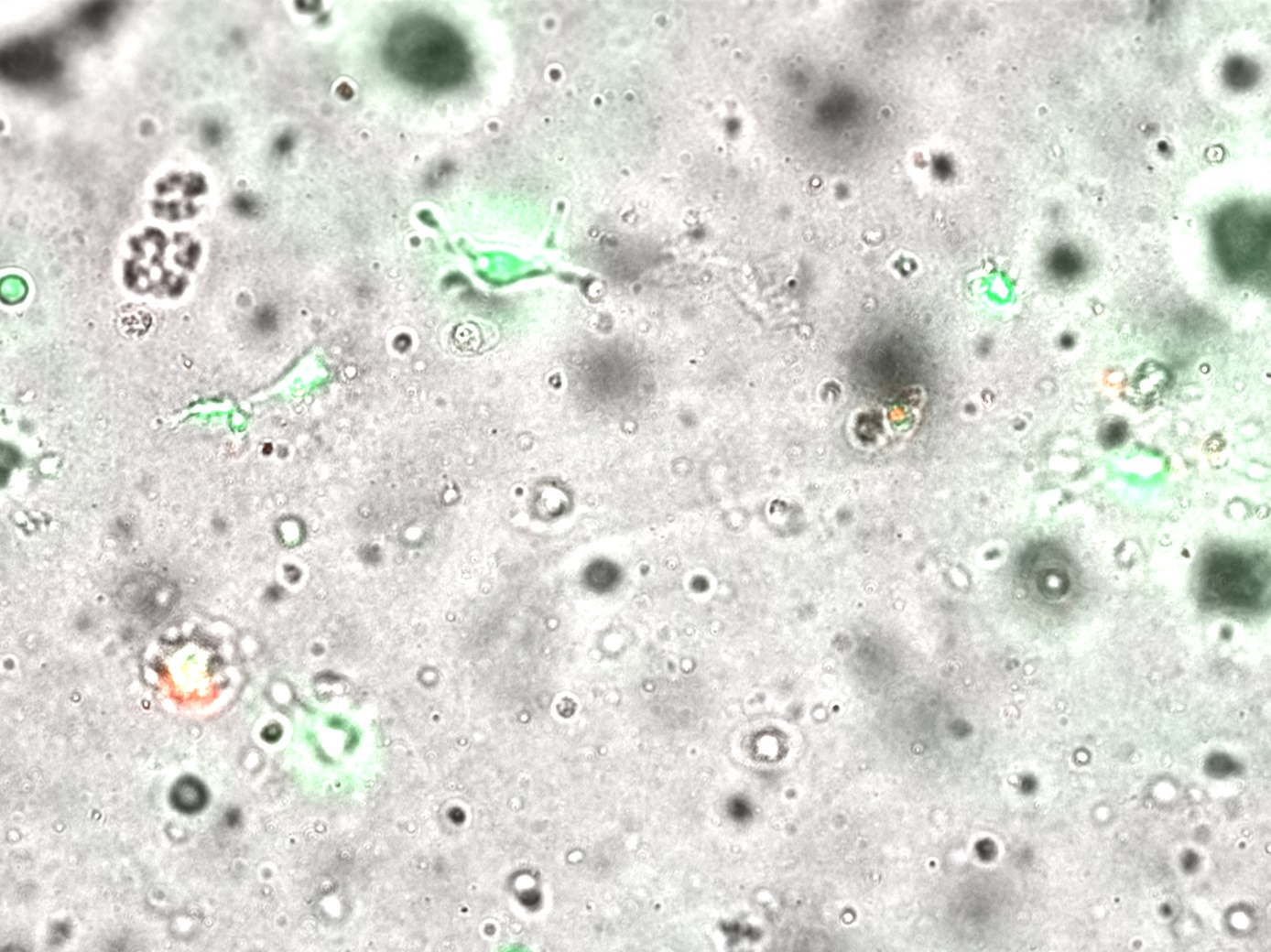

Supplement: Supplementary file 11 — Source data Fig. 9 [file 44321_2024_186_MOESM11_ESM.zip › Figure 9/9G/LuCaP 173.1/D1SP/Merged.jpg]

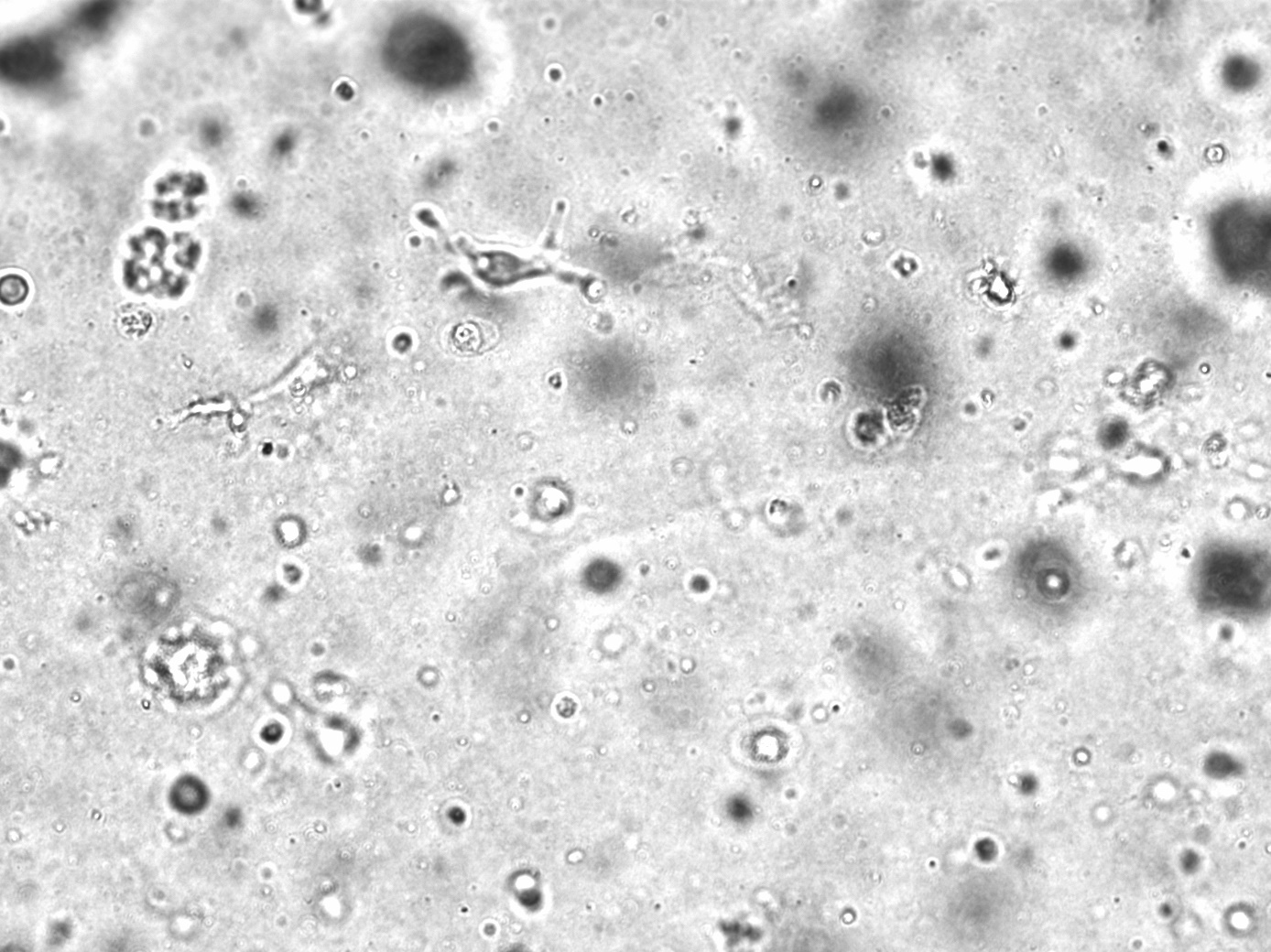

Supplement: Supplementary file 11 — Source data Fig. 9 [file 44321_2024_186_MOESM11_ESM.zip › Figure 9/9G/LuCaP 173.1/D1SP/BF.jpg]

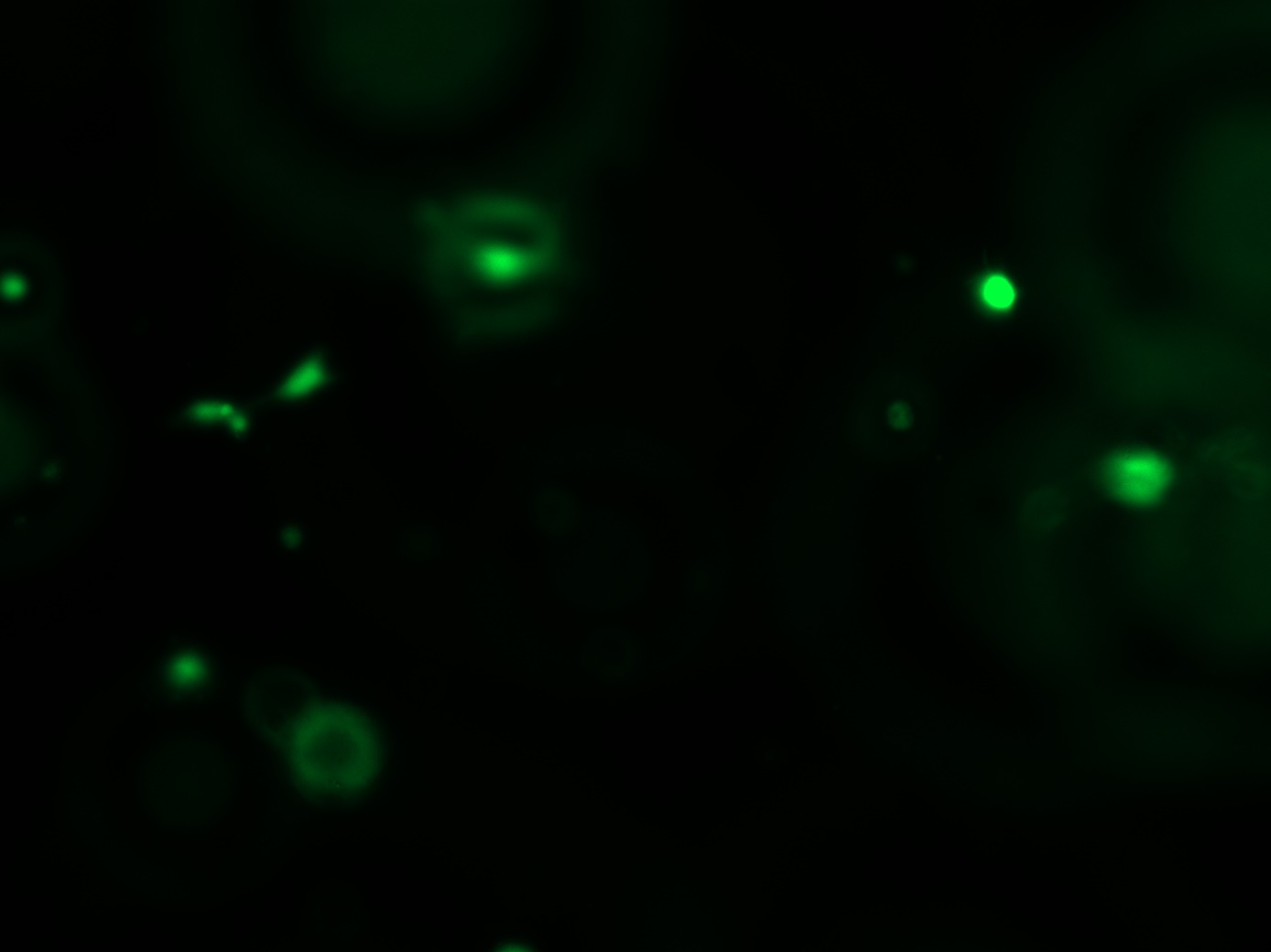

Supplement: Supplementary file 11 — Source data Fig. 9 [file 44321_2024_186_MOESM11_ESM.zip › Figure 9/9G/LuCaP 173.1/D1SP/Calcein.jpg]

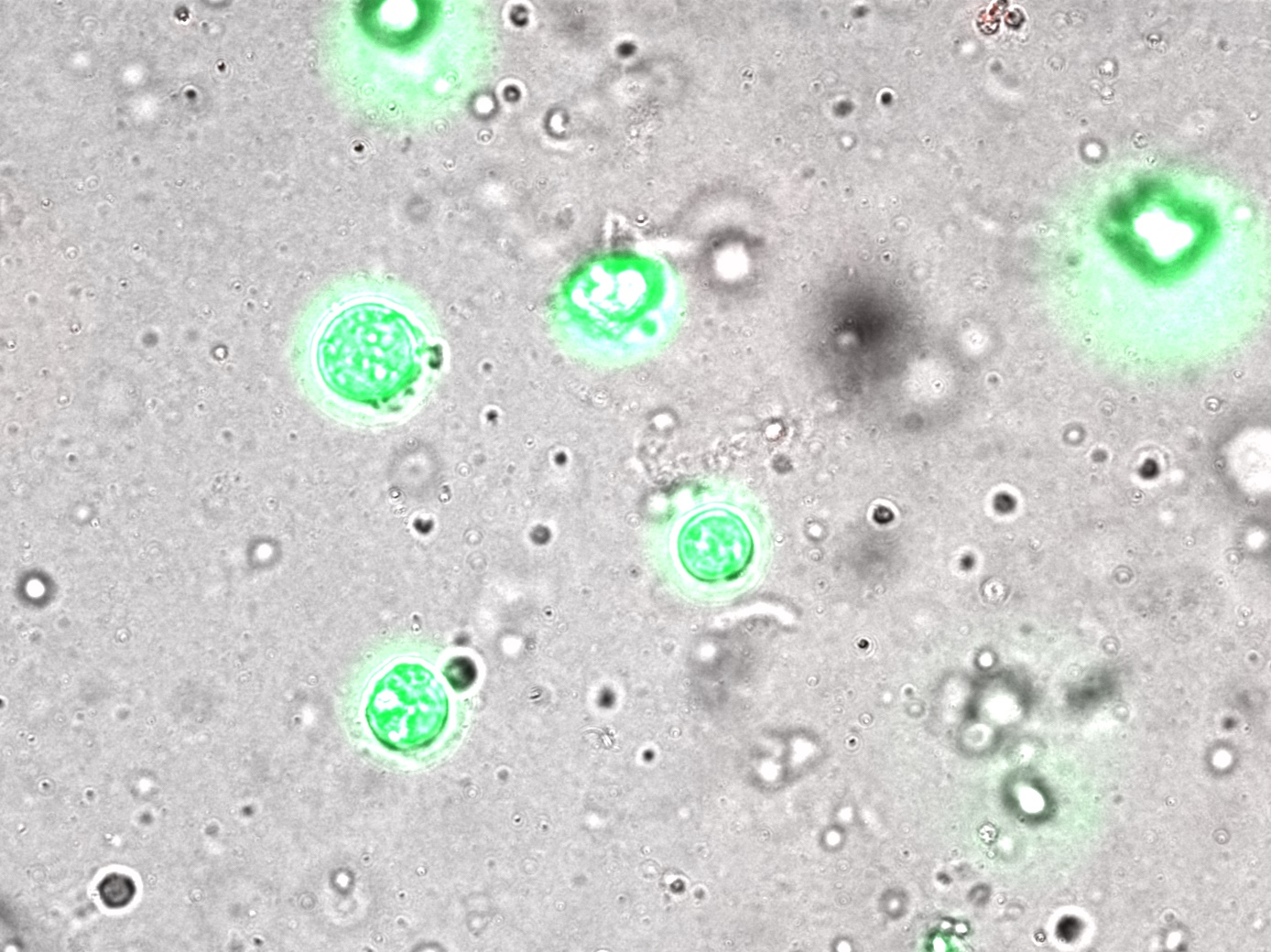

Supplement: Supplementary file 11 — Source data Fig. 9 [file 44321_2024_186_MOESM11_ESM.zip › Figure 9/9G/LuCaP 173.1/Vehicle/Merged.jpg]

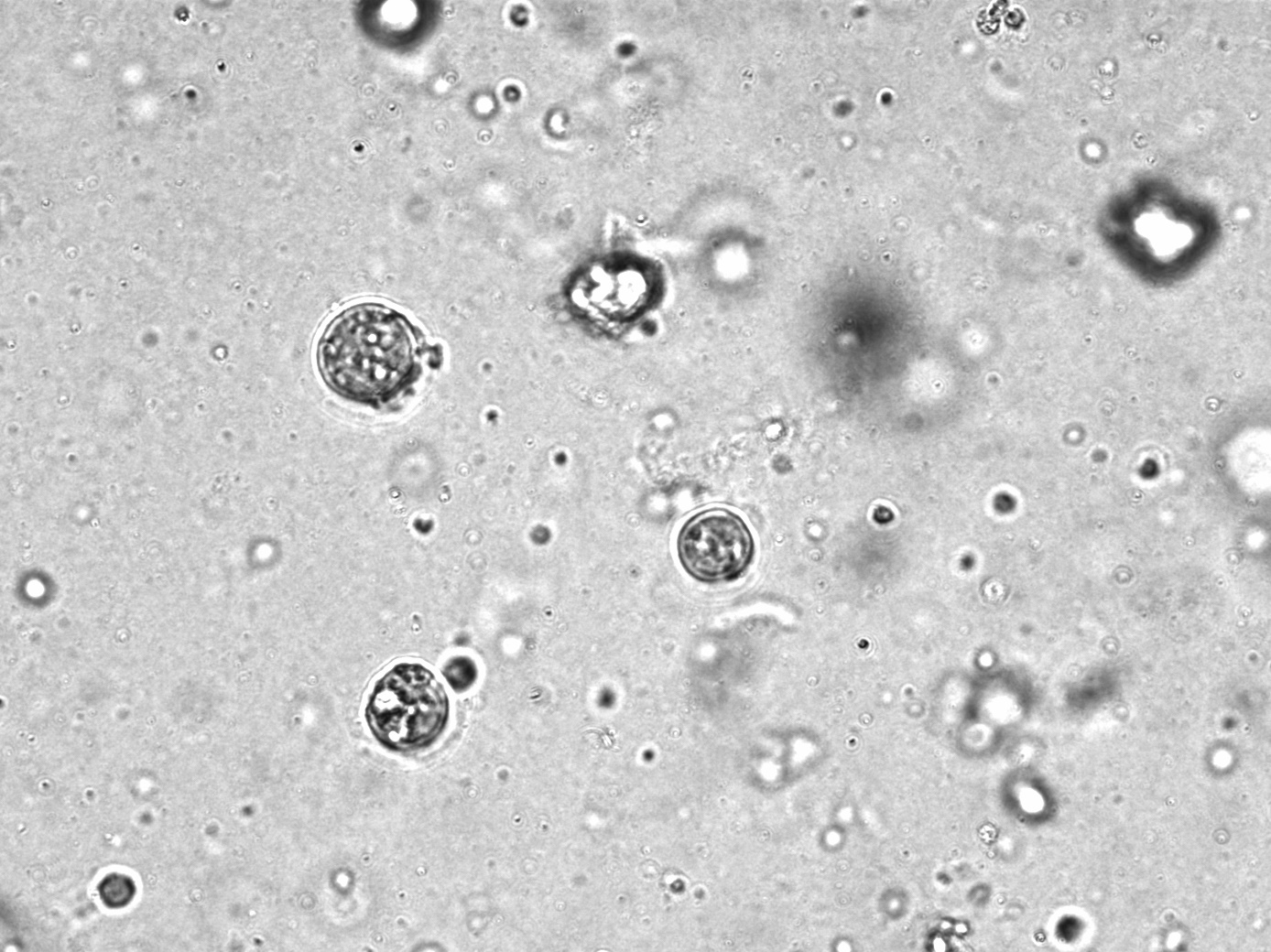

Supplement: Supplementary file 11 — Source data Fig. 9 [file 44321_2024_186_MOESM11_ESM.zip › Figure 9/9G/LuCaP 173.1/Vehicle/BF.jpg]

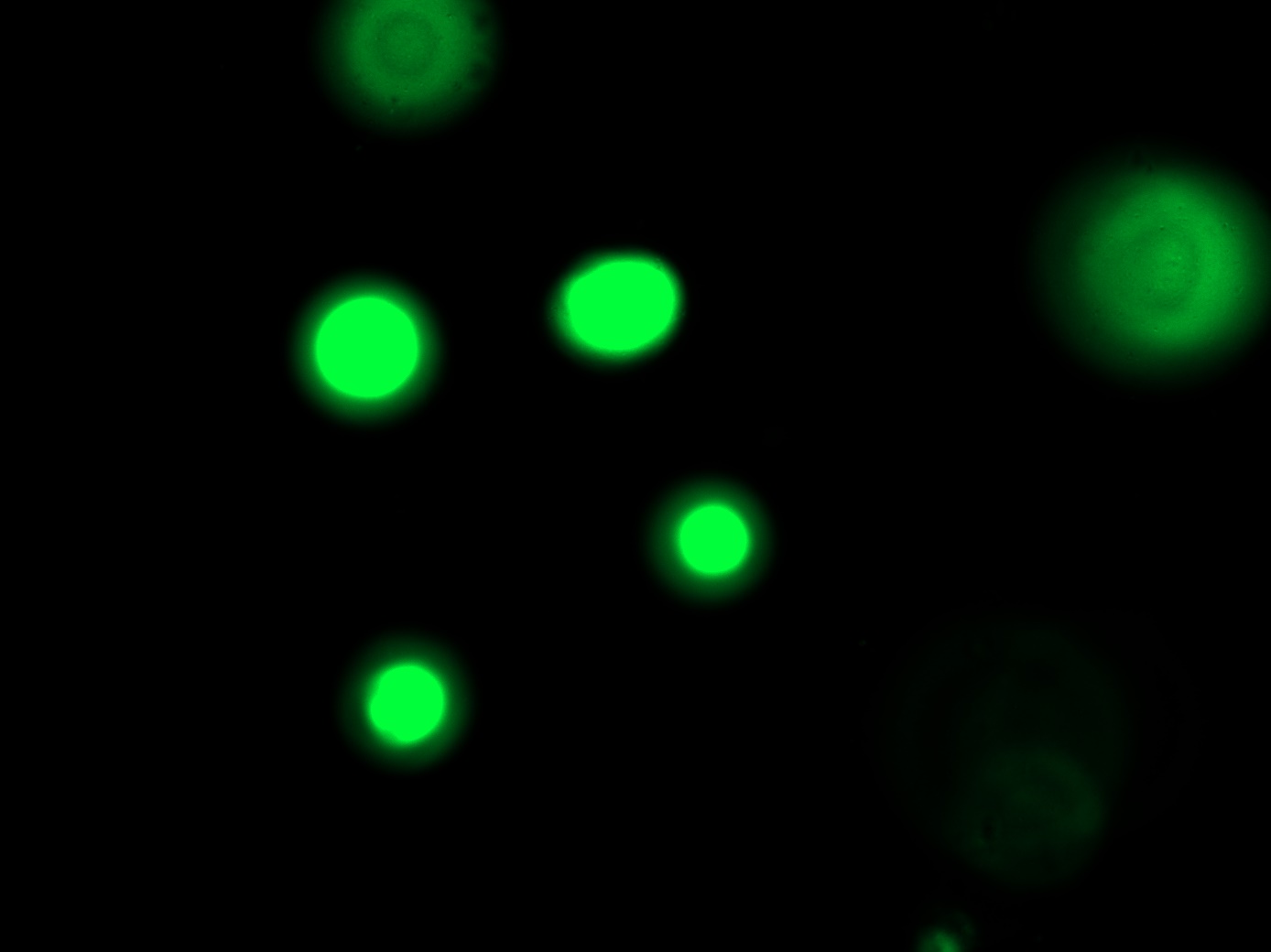

Supplement: Supplementary file 11 — Source data Fig. 9 [file 44321_2024_186_MOESM11_ESM.zip › Figure 9/9G/LuCaP 173.1/Vehicle/Calcein.jpg]

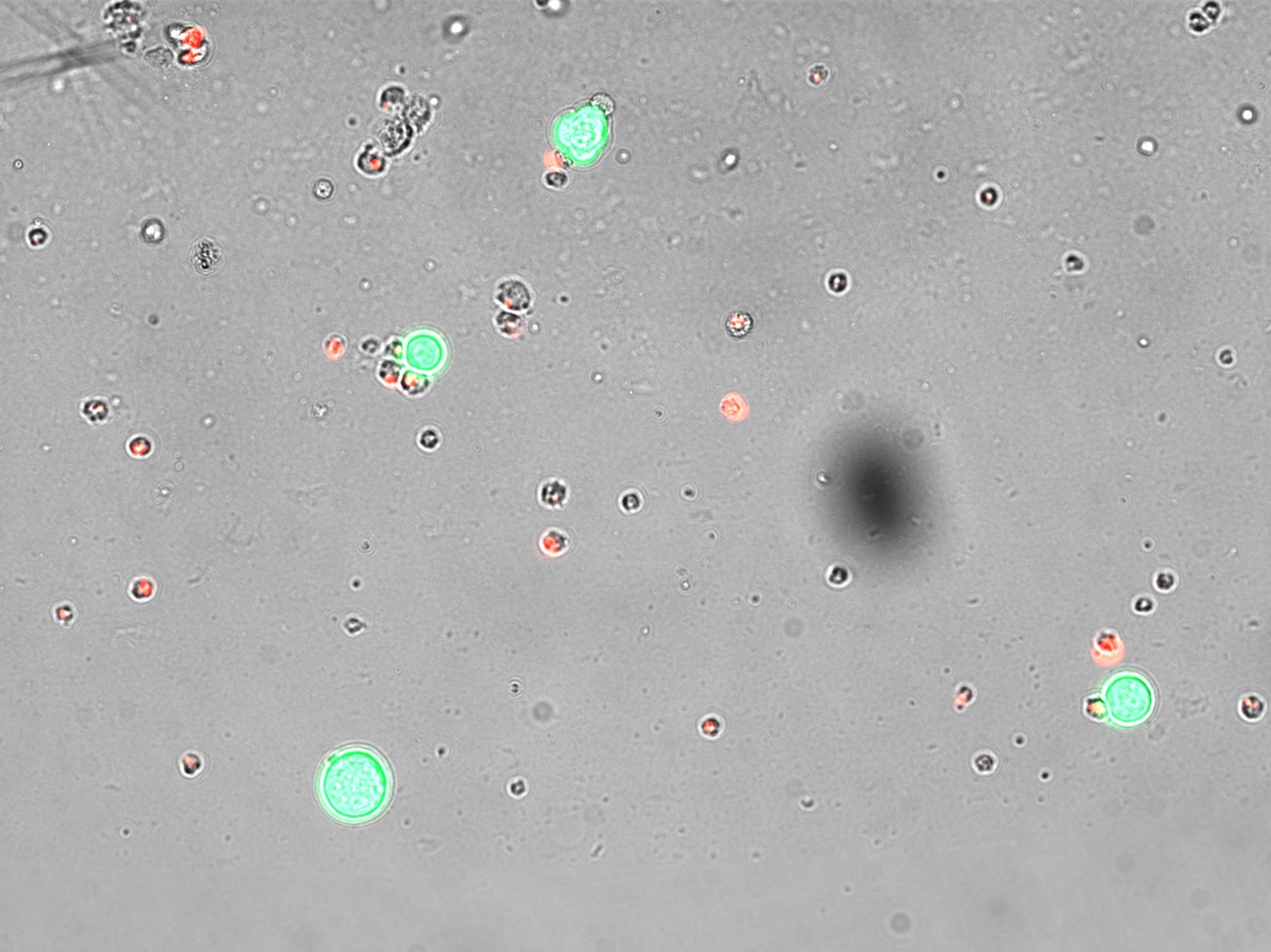

Supplement: Supplementary file 11 — Source data Fig. 9 [file 44321_2024_186_MOESM11_ESM.zip › Figure 9/9G/LuCaP 147CR/D1SP/Merged.jpg]

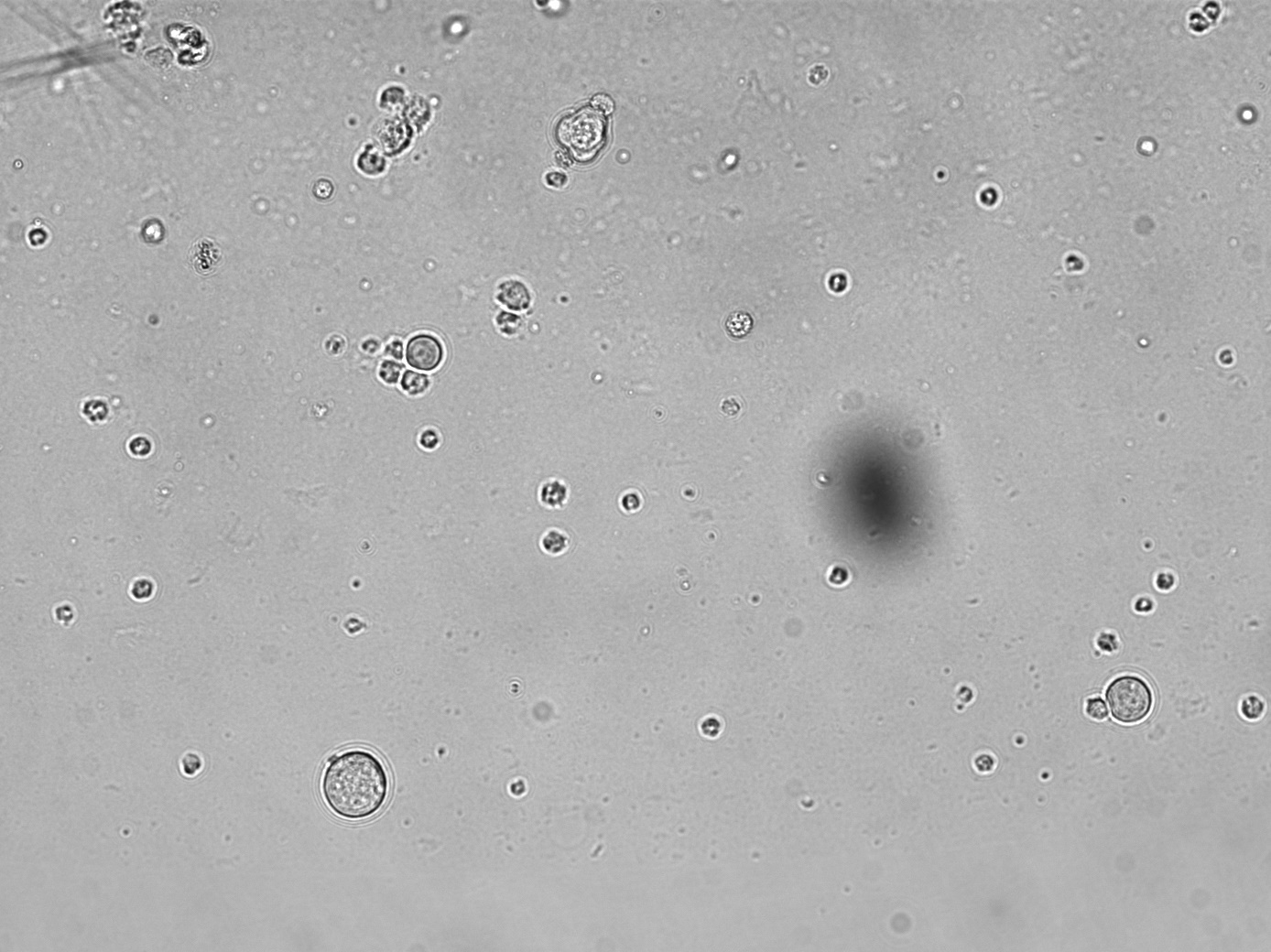

Supplement: Supplementary file 11 — Source data Fig. 9 [file 44321_2024_186_MOESM11_ESM.zip › Figure 9/9G/LuCaP 147CR/D1SP/BF.jpg]

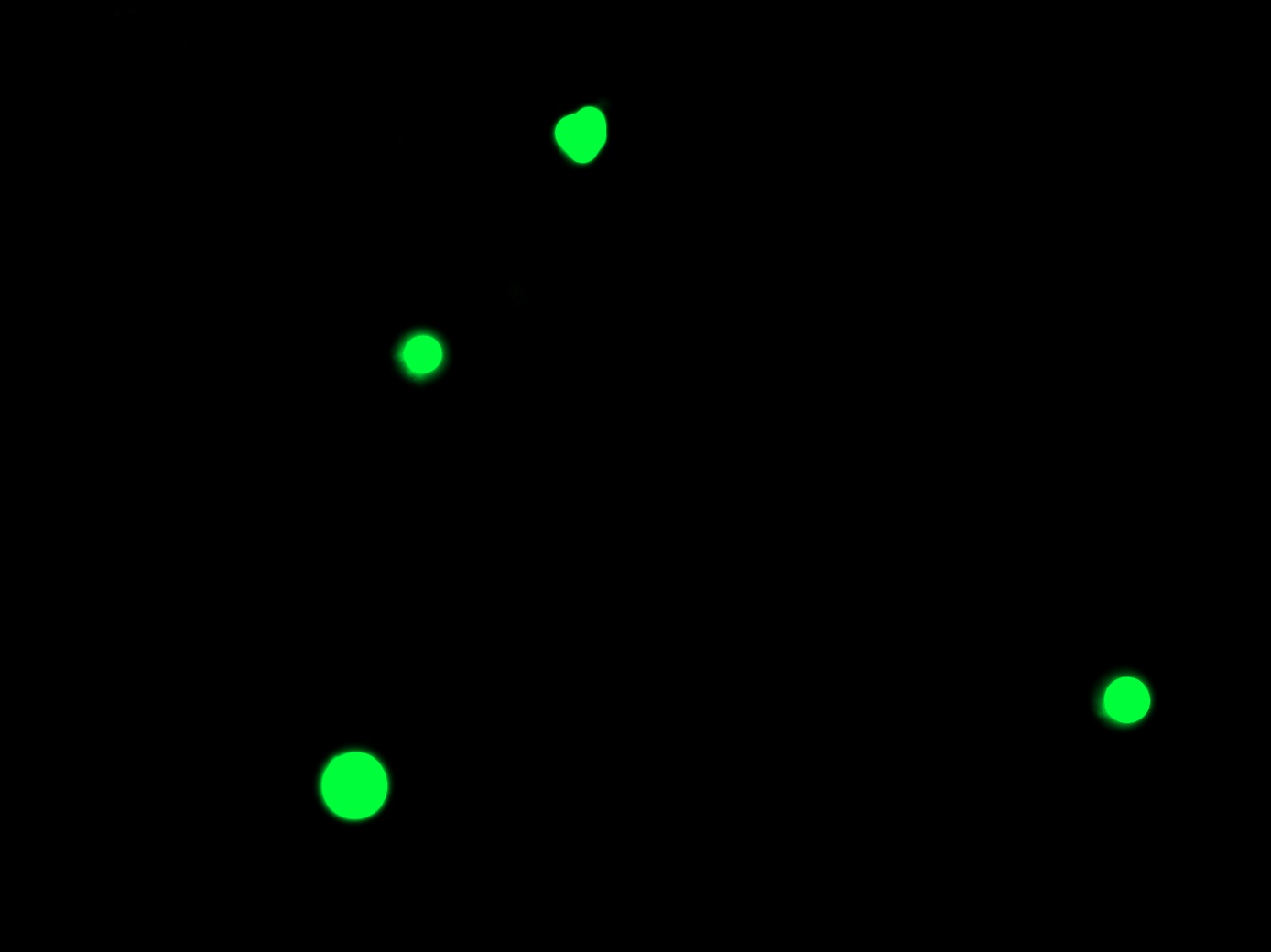

Supplement: Supplementary file 11 — Source data Fig. 9 [file 44321_2024_186_MOESM11_ESM.zip › Figure 9/9G/LuCaP 147CR/D1SP/Calcein.jpg]

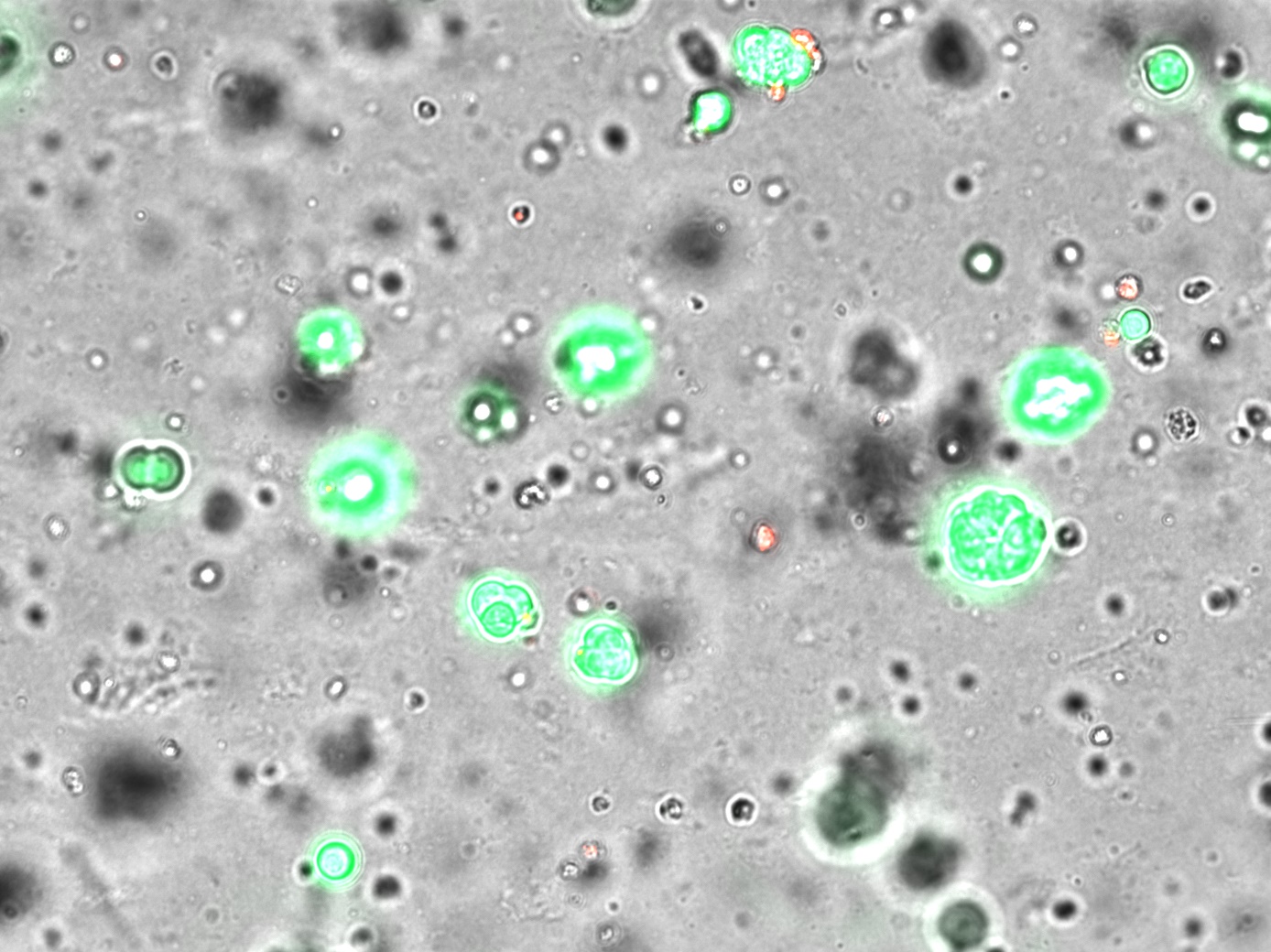

Supplement: Supplementary file 11 — Source data Fig. 9 [file 44321_2024_186_MOESM11_ESM.zip › Figure 9/9G/LuCaP 147CR/Vehicle/Merged.jpg]

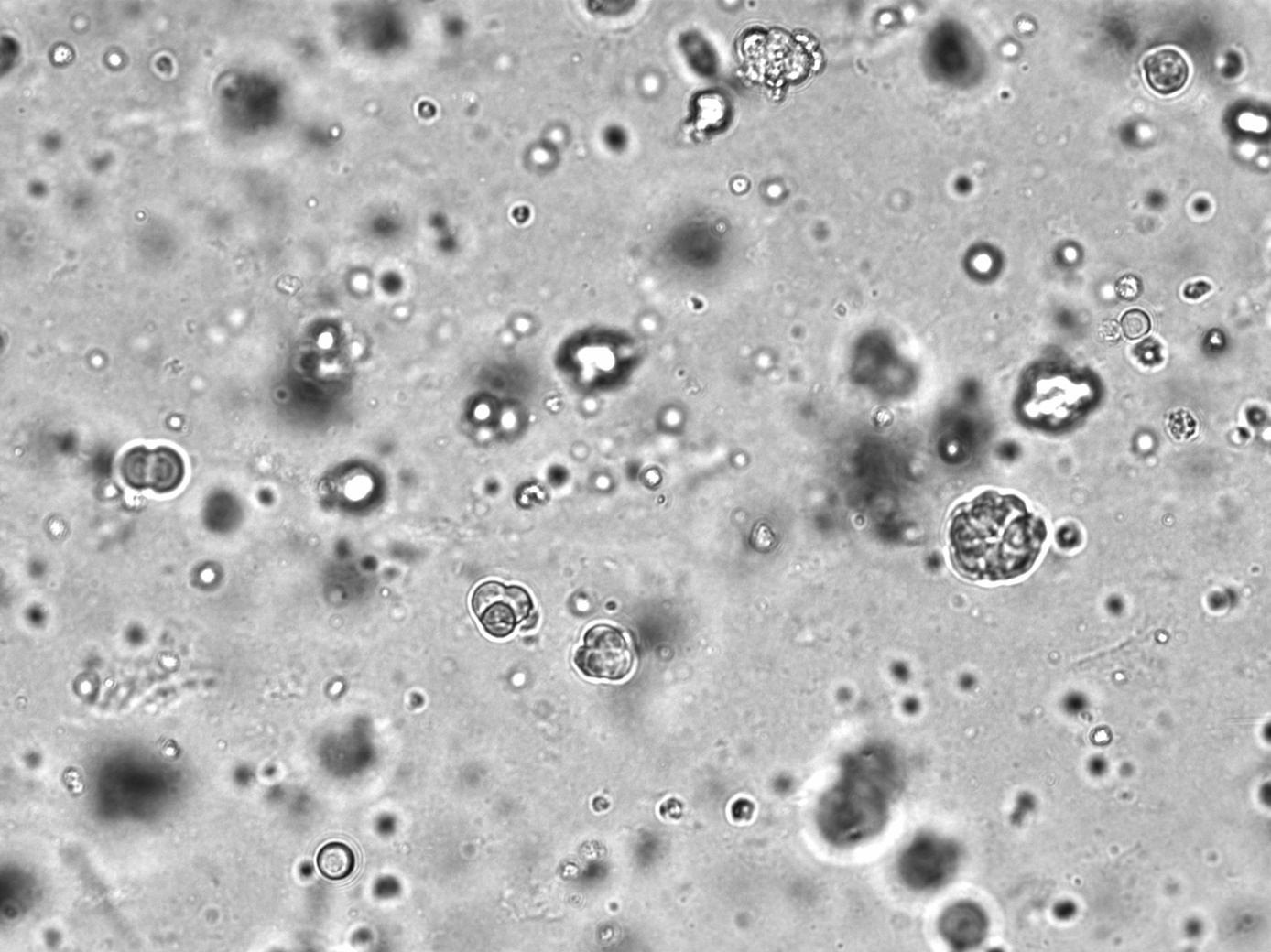

Supplement: Supplementary file 11 — Source data Fig. 9 [file 44321_2024_186_MOESM11_ESM.zip › Figure 9/9G/LuCaP 147CR/Vehicle/BF.jpg]

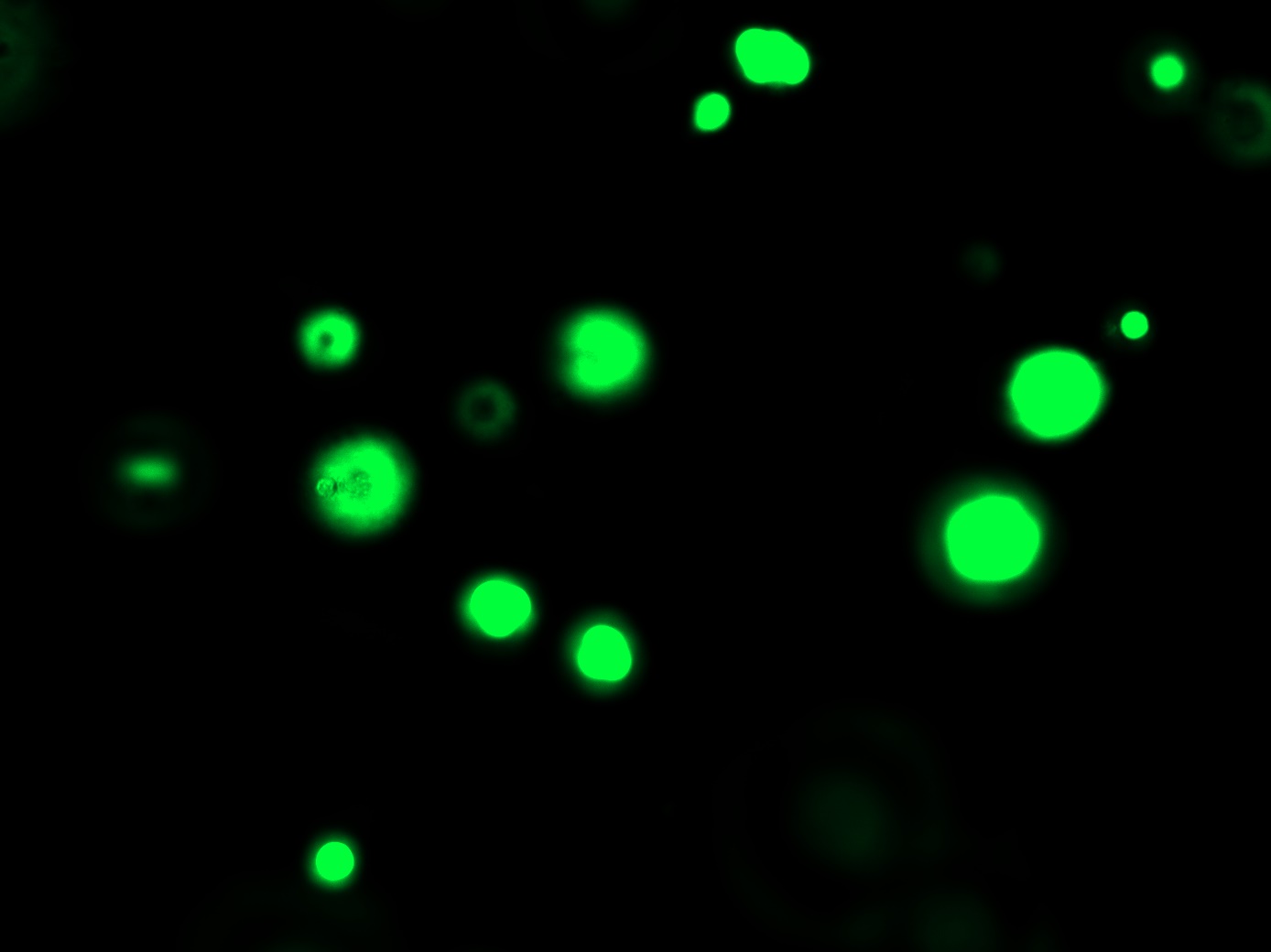

Supplement: Supplementary file 11 — Source data Fig. 9 [file 44321_2024_186_MOESM11_ESM.zip › Figure 9/9G/LuCaP 147CR/Vehicle/Calcein.jpg]

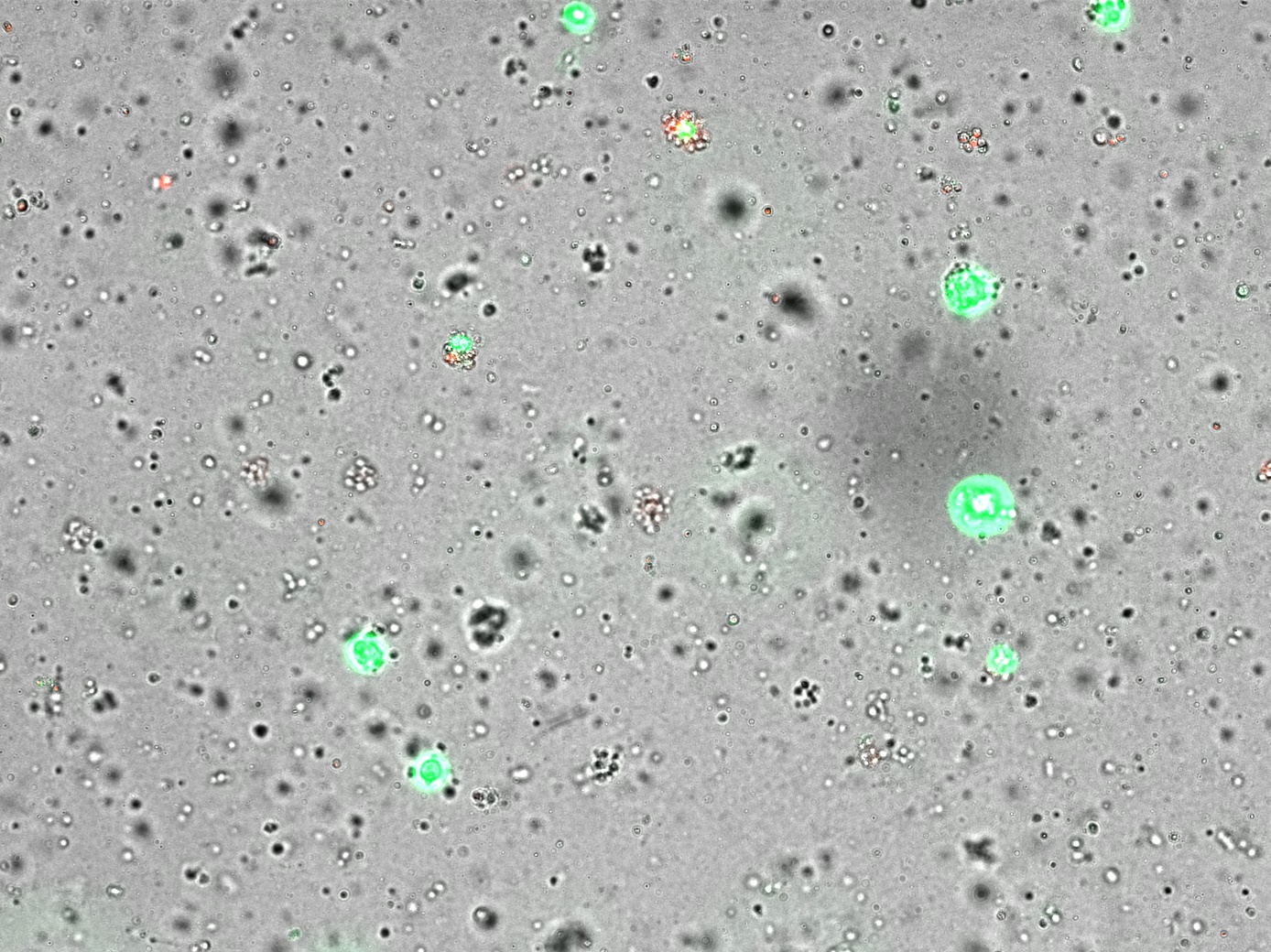

Supplement: Supplementary file 11 — Source data Fig. 9 [file 44321_2024_186_MOESM11_ESM.zip › Figure 9/9G/LuCaP 49/D1SP/Merged.jpg]

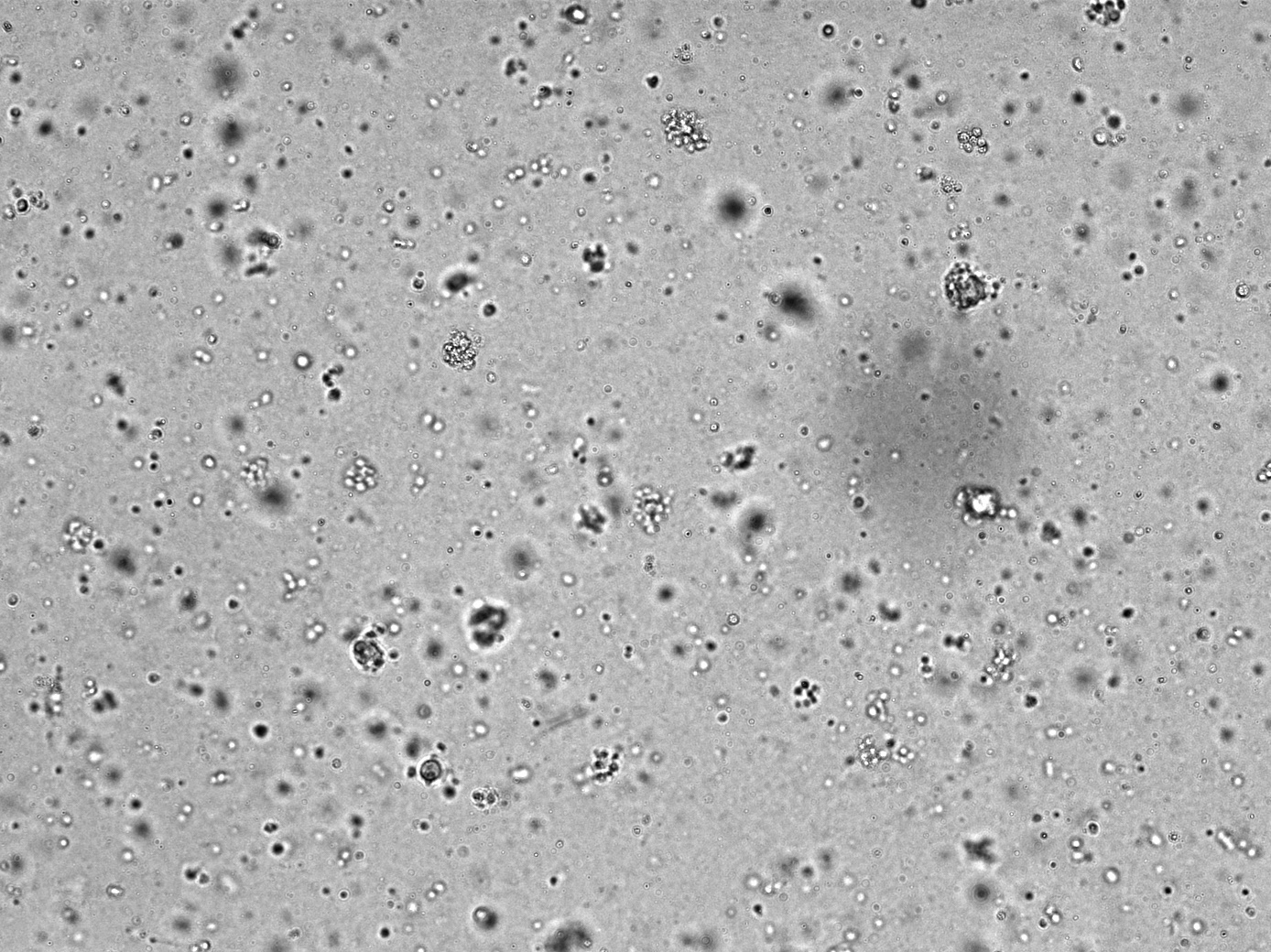

Supplement: Supplementary file 11 — Source data Fig. 9 [file 44321_2024_186_MOESM11_ESM.zip › Figure 9/9G/LuCaP 49/D1SP/BF.jpg]

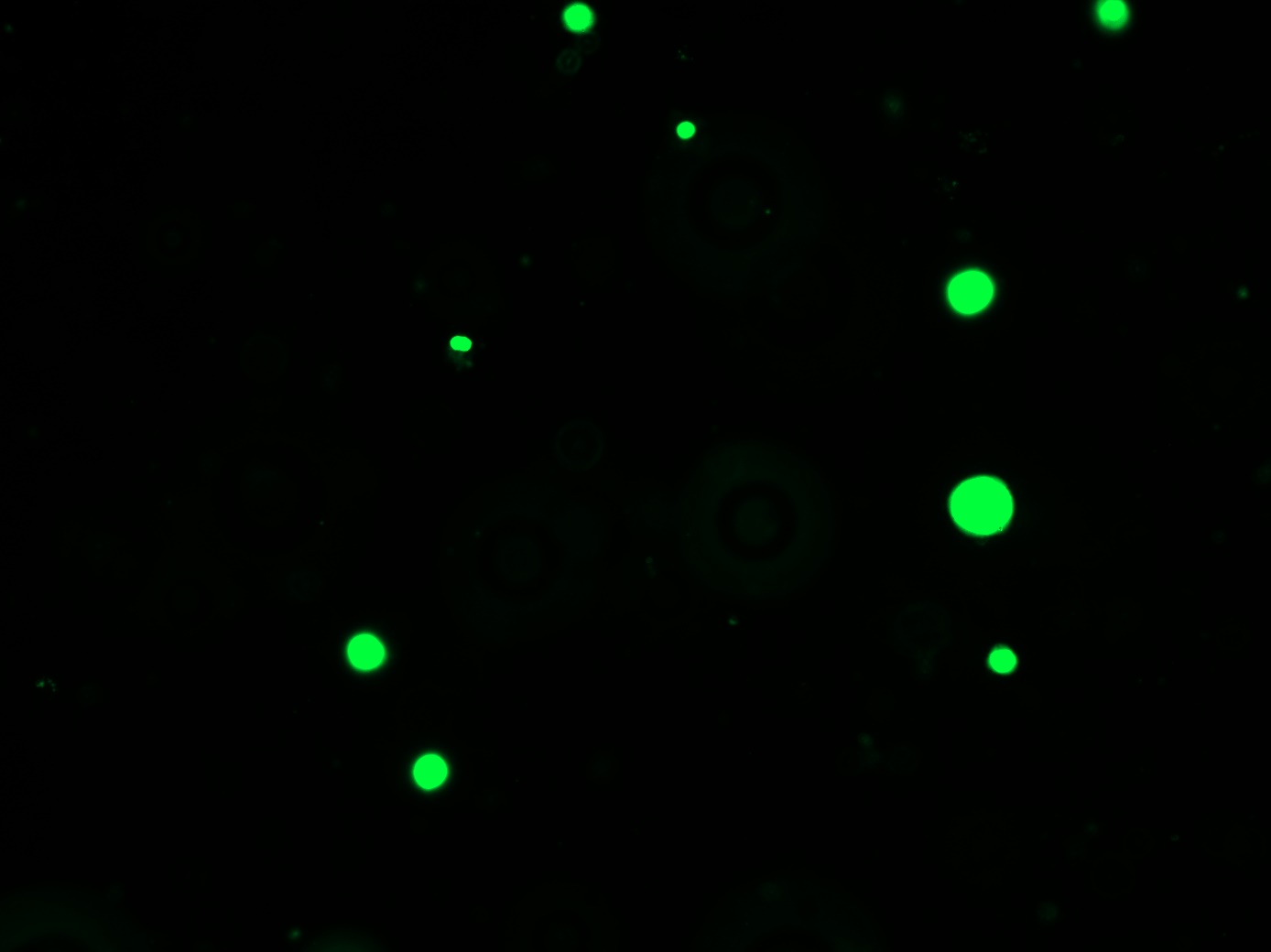

Supplement: Supplementary file 11 — Source data Fig. 9 [file 44321_2024_186_MOESM11_ESM.zip › Figure 9/9G/LuCaP 49/D1SP/Calcein.jpg]

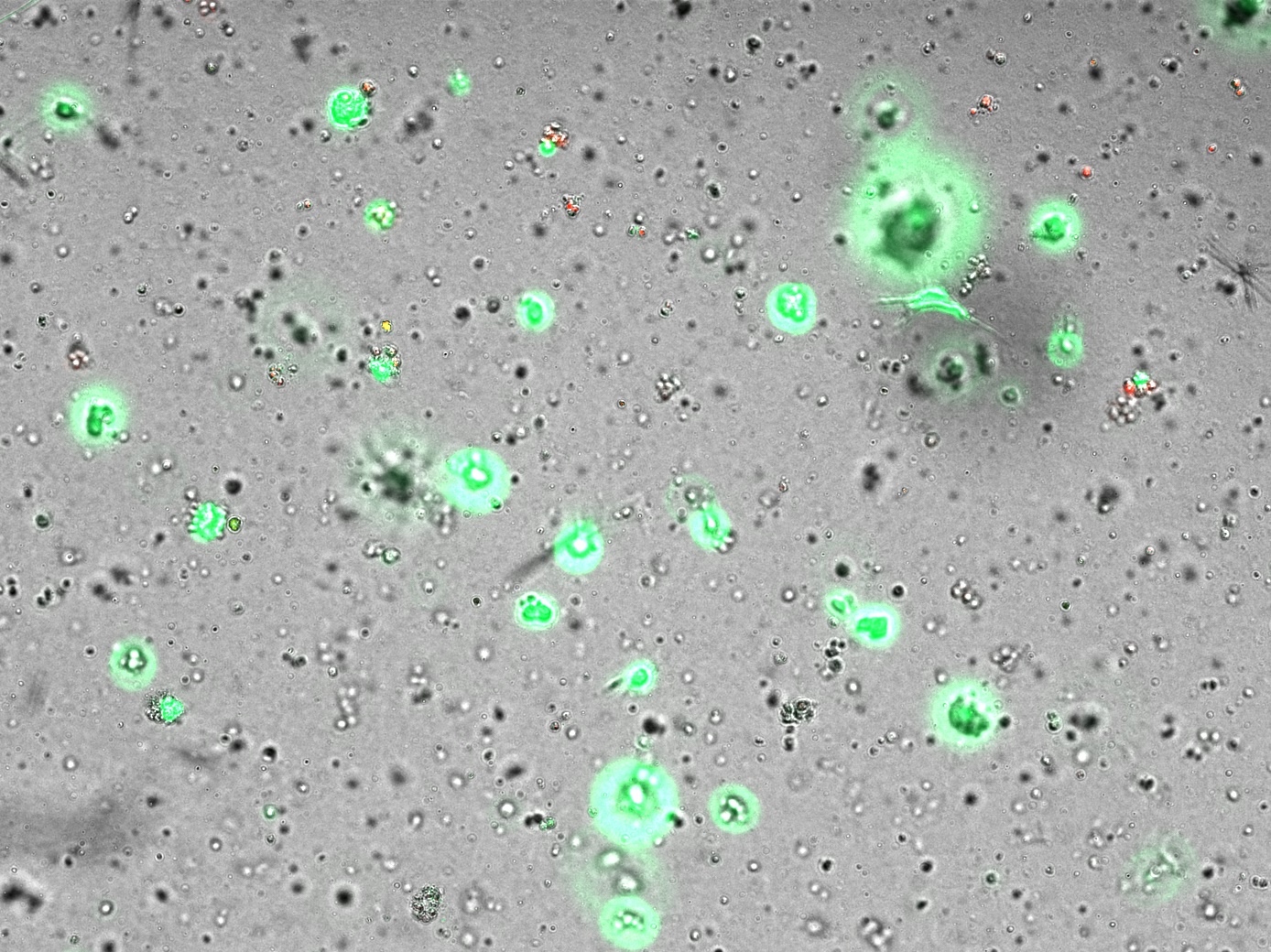

Supplement: Supplementary file 11 — Source data Fig. 9 [file 44321_2024_186_MOESM11_ESM.zip › Figure 9/9G/LuCaP 49/Vehicle/Merged.jpg]

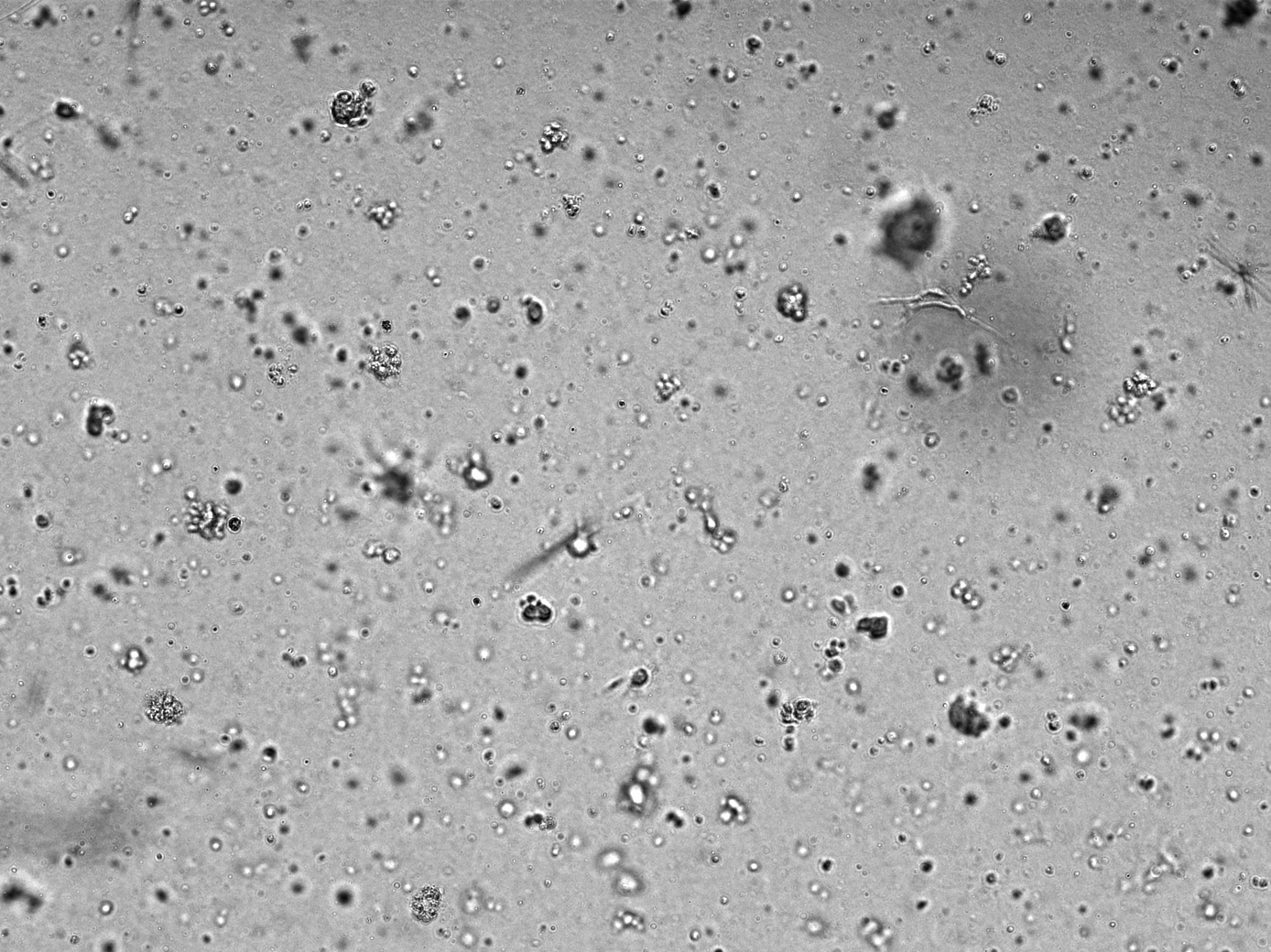

Supplement: Supplementary file 11 — Source data Fig. 9 [file 44321_2024_186_MOESM11_ESM.zip › Figure 9/9G/LuCaP 49/Vehicle/BF.jpg]

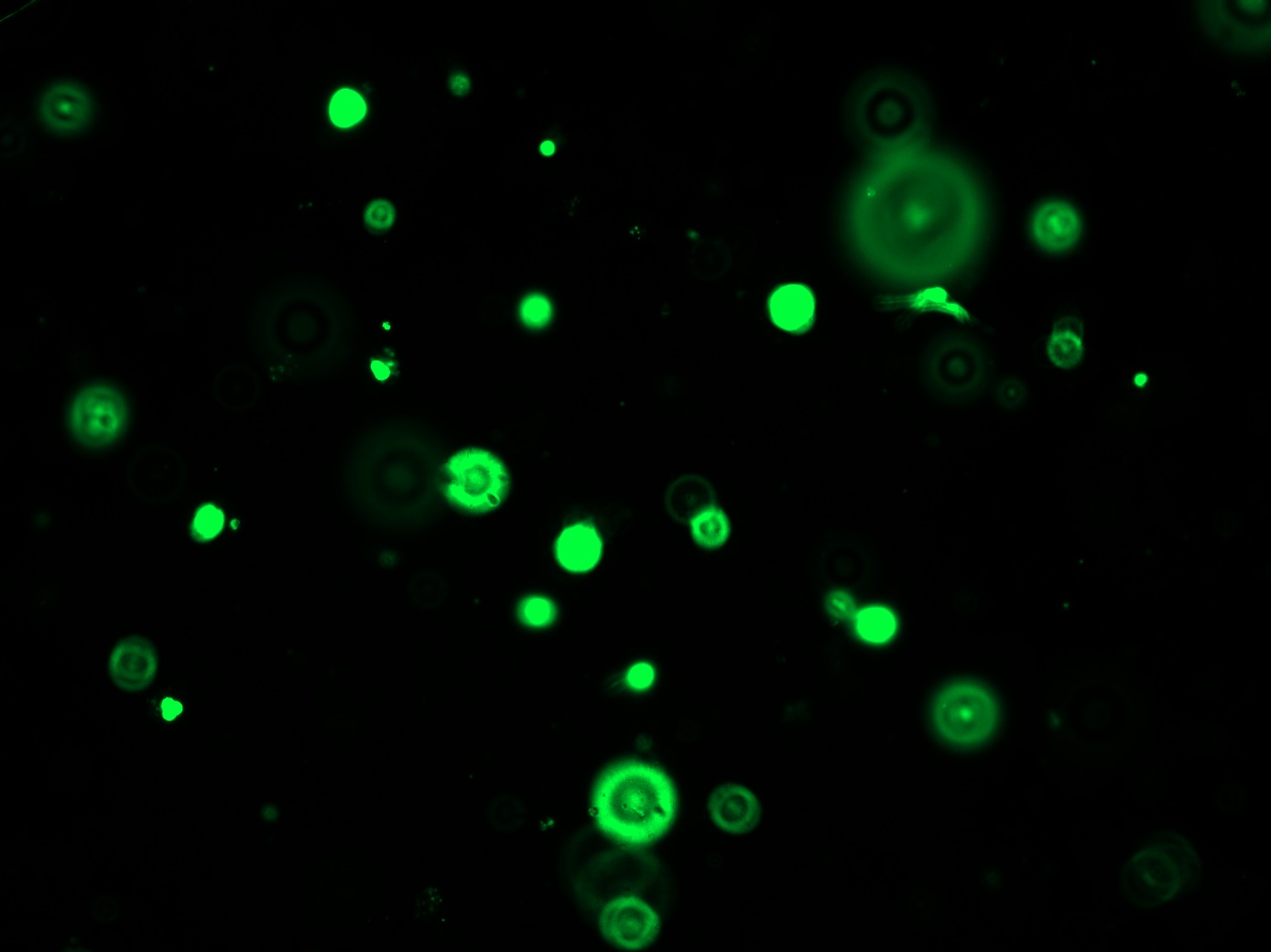

Supplement: Supplementary file 11 — Source data Fig. 9 [file 44321_2024_186_MOESM11_ESM.zip › Figure 9/9G/LuCaP 49/Vehicle/Calcein.jpg]
